# Mediating Effects of Immunophenotypes on the Causal Relationship of Gut Microbiota and Their Metabolic Pathways With Osteonecrosis: A Mendelian Randomization Analysis

**DOI:** 10.1155/mi/9323113

**Published:** 2025-10-28

**Authors:** Minlong Wang, Yongjiu Meng, Qinrong Shen, Hongming Meng

**Affiliations:** Department of Orthopedics, Shaoxing Hospital of Traditional Chinese Medicine, Shaoxing TCM Hospital Affiliated to Zhejiang Chinese Medical University, Shaoxing, Zhejiang, China

**Keywords:** Bayesian weighted Mendelian randomization, gut microbiota, immunophenotypes, Mendelian randomization, osteonecrosis

## Abstract

**Background:**

This study examined the potential causal relationship between gut microbiota and osteonecrosis and analyzed the mediating effects of immunophenotypes to establish evidence-based causal associations.

**Methods:**

Bayesian weighted Mendelian randomization and the inverse-variance weighted method were used to evaluate the causal relationships between osteonecrosis and 412 gut microbial species and their metabolic pathways, as well as between osteonecrosis and 731 immune cell signatures. Mediation analysis was conducted to assess the role of immune cells in mediating the relationship between gut microbiota and osteonecrosis.

**Results:**

A total of 16 gut microbial species and their metabolic pathways and 23 immunophenotypes were identified as causal factors in susceptibility to osteonecrosis (*p* < 0.05). The superpathway of sulfate assimilation and cysteine biosynthesis of the gut microbiota demonstrated a protective effect against osteonecrosis. The absolute count of CD33^−^ HLA DR + Myeloid cell exhibited a mediation effect of 4.15%, while T cell %lymphocyte demonstrated a mediation effect of 12.16% in this protective mechanism.

**Conclusion:**

The study findings highlighted the role of the superpathway of sulfate assimilation and cysteine biosynthesis of the gut microbiota in exerting a protective effect against osteonecrosis. This protective effect is substantially mediated by a decrease in the absolute count of CD33^−^ HLA DR + Myeloid cell and an increase in T cell % lymphocyte.

## 1. Introduction

Osteonecrosis represents a condition characterized by insufficient nutrient supply to critical bone regions, resulting in bone tissue death; this condition predominantly affects the femoral head and leads to secondary hip osteoarthritis. This multifactorial disease is classified into two main categories: traumatic and nontraumatic [1]. Osteonecrosis typically occurs in middle-aged individuals and frequently results in severe functional impairment due to the low mechanical strength of the necrotic bone tissue [2]. In nontraumatic cases, glucocorticoid use is the predominant causative factor of osteonecrosis. In addition, an increased incidence of jaw osteonecrosis is associated with several osteoporosis-related drugs, including denosumab and bisphosphonates [1, 3]. However, there is currently no highly effective treatment for osteonecrosis.

The human intestine is a complex microscopic ecosystem. The colon of a 70 kg adult contains ~3.8 × 10^13^ bacteria [4]. These microorganisms facilitate digestion and substantially influence the immune system and bone metabolism of the host [5, 6]. According to recent research, alterations in gut microbiota composition may contribute to osteoarthritis development [6, 7]. Zheng et al. [8] showed that alcohol consumption and hormonal factors can modify the composition of the gut microbiota and its metabolic pathways and thus significantly impact the occurrence of osteonecrosis of the femoral head (ONFH). Chen et al. [9] revealed that the gut microbiota can protect the femoral head through bacterial extracellular vesicles, while the loss of *Lactobacillus animalis* and its extracellular vesicles was associated with glucocorticoid-induced ONFH. However, substantial knowledge gaps remain regarding the influence of alterations in the composition of the gut microbiota and their metabolic pathways on osteonecrosis. Current research indicates that the gut microbiota affects the immune system [10, 11]. Moreover, recent studies have also identified complex causal relationships between various immunophenotypes and osteonecrosis [12, 13]. Nevertheless, the role of immunophenotypes in mediating the effect of the gut microbiota on osteonecrosis remains poorly understood.

Mendelian randomization (MR) analysis has gained increasing prominence in recent years for investigating causal relationships between exposure and outcome [14–16]. MR represents an alternative approach that utilizes instrumental variables (IVs) strongly associated with exposure to identify potential causal relationships between exposure and outcome [17]. Because confounding factors typically show no correlation with genetic variants, and variants are randomly assigned during meiosis, MR studies are identical to randomized controlled trials [18]. In the present study, we used a two-sample, mediating MR analysis to examine the causal relationship between the gut microbiota and osteonecrosis as well as to analyze the mediating effect of immunophenotypes; the findings of this study could offer novel insights into the pathogenesis and treatment of osteonecrosis.

## 2. Methods

### 2.1. Experimental Design

**Figure 1:**
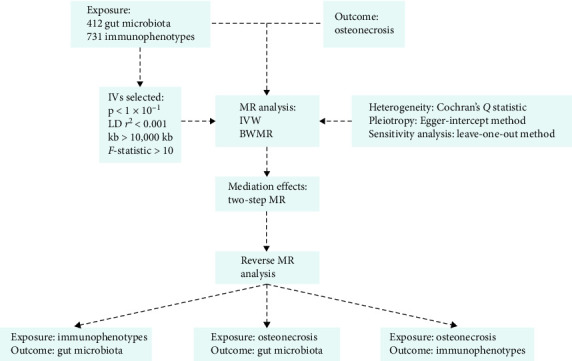
Study flowchart.

This study utilized a two-sample, mediating MR analysis to investigate the causal relationship between the gut microbiota and osteonecrosis, as well as the role of immune cells in this process (Figure 1). IVs were selected based on the following three essential criteria: (1) IVs demonstrate direct correlation with exposure; (2) IVs show no correlation with confounding variables; and (3) IVs influence outcomes exclusively through the exposure [14]. This study adheres to the reporting guidelines of STROBE-MR (Supplement 1).

### 2.2. Data Sources

The genome-wide association study (GWAS) datasets utilized in this investigation were obtained from public sources and published literature. The GWAS dataset for osteonecrosis was acquired from the FinnGen consortium (https://www.finngen.fi/en), which includes 1543 cases and 391,037 controls (phenocode: M13_OSTEONECROSIS) [19]. Lopera-Maya et al. [20] analyzed the metagenomic sequencing data from 7738 northern Dutch volunteers; the authors identified 207 microbial taxa and 205 metabolic pathways, characterized microbial composition and function, and established the gut microbiota GWAS datasets. The GWAS datasets for immune cells were obtained from the GWAS Catalog (GCST0001391 to GCST0002121), which includes 731 immunophenotypes [21].

### 2.3. Selection of IVs

A series of parameters was established to select appropriate IVs. The significance levels were set at 1 × 10^−5^ by referring to multiple studies [12, 13]. Single nucleotide polymorphisms (SNPs) were clustered to reduce linkage disequilibrium by using PLINK software (window size = 10,000 kb, r2 < 0.001). To minimize bias caused by IVs, the statistical strength *F* value was calculated for each SNP (F = (*N* − 2) × R2/(1 − R2)). *F* > 10 indicates minimal instrument bias [22]. Therefore, the IVs in this study were required to meet *F* > 10. These criteria adhered to previous study recommendations and simultaneously ensured the reliability and accuracy of the IVs. The IVs of the gut microbiota, immune cells, and osteonecrosis were screened using the abovementioned criteria.

### 2.4. Sensitivity Analysis

We specifically included methods to assess pleiotropy and heterogeneity. The heterogeneity of IVs was evaluated using Cochran's Q statistic (*p* < 0.05 indicated heterogeneity) [23]. The Egger-intercept method was utilized to assess horizontal pleiotropy (*p* < 0.05 indicated pleiotropy) [24]. Finally, the results were subjected to a sensitivity analysis by using the leave-one-out method.

### 2.5. Statistical Analysis

The “TwoSampleMR” package (version 0.5.7) and the “Bayesian Weighted Mendelian Randomization (BWMR)” package in R software (version 4.3.0) were used for statistical analyses. Causality was assessed using BWMR [25] and the inverse-variance weighted (IVW) method. MR analyses were performed with gut microbiota and immunophenotypes as exposures and osteonecrosis as the outcome to explore their causal relationships.

To evaluate how immunophenotypes mediate the effects of the gut microbiota on osteonecrosis, we performed the two-step MR mediation analysis. First, we evaluated the causal relationship between the gut microbiota and immunophenotypes (denoted as β1); we then identified the causal relationship between immunophenotypes and osteonecrosis (denoted as β2) and finally identified the causal relationship between the gut microbiota and osteonecrosis (denoted as β3). β1, β2, and β3 were all obtained through the above IVW. The mediation effect (β12 = β1*⁣*^*∗*^β2) was subsequently calculated.

Lastly, reverse MR analyses were conducted with immunophenotypes as the exposure and gut microbiota as the outcome, and with osteonecrosis as the exposure and gut microbiota and immunophenotypes as the outcomes to validate the absence of reverse causation.

## 3. Results

### 3.1. The Causal Relationship Between the Gut Microbiota and Osteonecrosis

**Figure 2:**
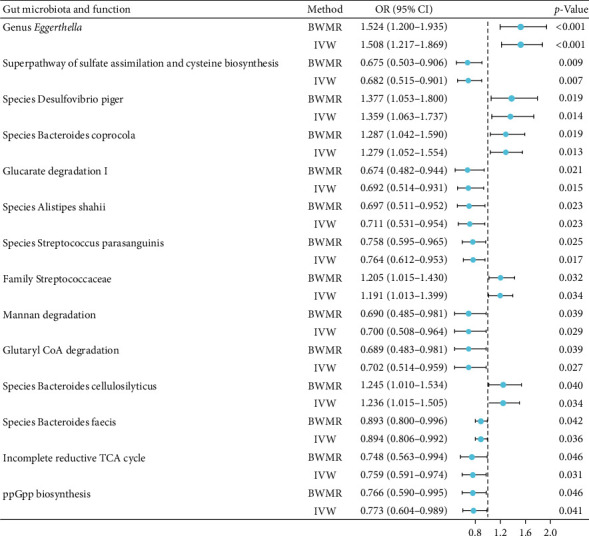
Forest diagram of the causal relationship between the gut microbiota and osteonecrosis. CI, confidence interval; OR, odds ratio.

The causal relationship between the gut microbiota and osteonecrosis was analyzed by BWMR and IVW. The findings revealed that 14 gut microbial species and their metabolic pathways showed causal associations with osteonecrosis. Specifically, genus *Eggerthella* (OR (95% CI) = 1.524 (1.200–1.935), *p* < 0.001); species *Desulfovibrio piger* (OR (95% CI) = 1.377 (1.053–1.800), *p*=0.019), *Bacteroides coprocola* (OR (95% CI) = 1.287 (1.042–1.590), *p*=0.019), and *Bacteroides cellulosilyticus* (OR (95% CI) = 1.245 (1.010–1.534), *p*=0.040); and family *Streptococcaceae* (OR (95% CI) = 1.205 (1.015–1.430), *p*=0.032) were identified as the risk factors for osteonecrosis. The elevated abundance of these gut microbial species and their metabolic pathways contributes to osteonecrosis development. Conversely, several protective factors against osteonecrosis were identified as follows: the superpathway of sulfate assimilation and cysteine biosynthesis (OR (95% CI) = 0.675 (0.503–0.906), *p*=0.009); glucarate degradation I (OR (95% CI) = 0.674 (0.482–0.944), *p*=0.021); species *Alistipes shahii* (OR (95% CI) = 0.697 (0.511–0.952), *p*=0.023), *Streptococcus parasanguinis* (OR (95% CI) = 0.758 (0.595–0.965), *p*=0.025), and *Bacteroides faecis* (OR (95% CI) = 0.893 (0.800–0.996), *p*=0.042); mannan degradation (OR (95% CI) = 0.690 (0.485–0.981), *p*=0.039); glutaryl-CoA degradation (OR (95% CI) = 0.689 (0.483–0.981), *p*=0.039); incomplete reductive TCA cycle (OR (95% CI) = 0.748 (0.563–0.994), *p*=0.046); and ppGpp biosynthesis (OR (95% CI) = 0.766 (0.590–0.995), *p*=0.046) (Figure 2). A reduction in the levels of these gut microbial species and their metabolic pathways is associated with the onset of osteonecrosis. The study findings indicated no significant pleiotropy or heterogeneity of these IVs (Supplement 2). Additionally, the leave-one-out analysis confirmed the reliability of the data. Scatterplots and leave-one-out plots are shown in Supplement 3.

### 3.2. The Causal Relationship Between Immune Cells and Osteonecrosis

**Figure 3:**
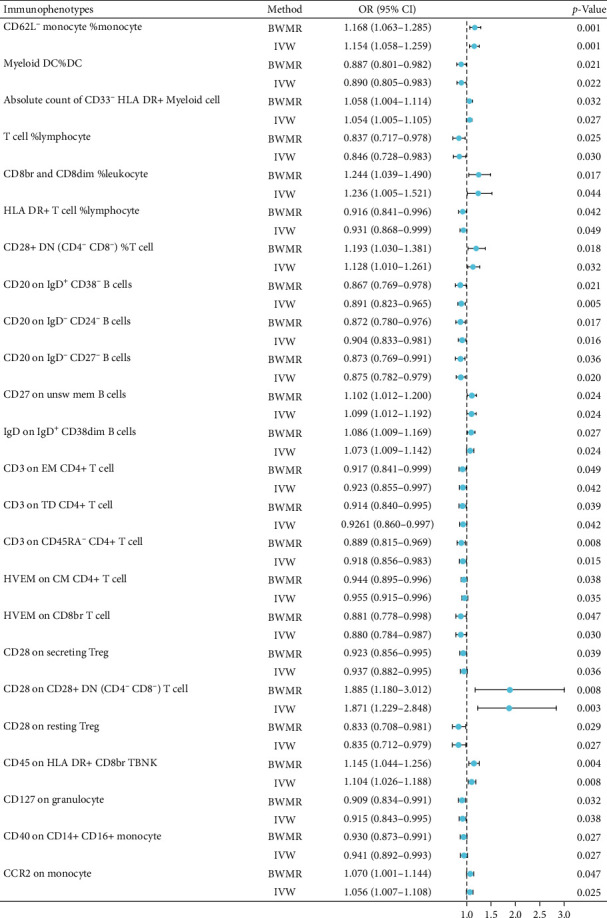
Forest diagram of the causal relationship between immunophenotypes and osteonecrosis.

By using the IVW and BWMR methods, we found significant causal associations between osteonecrosis and various immunophenotypes. The findings indicated that osteonecrosis development correlated with elevated levels of specific immune markers, including CD62L- monocyte %monocyte, absolute count of CD33^−^ HLA DR + Myeloid cell, CD8br and CD8dim %leukocyte, CD28+ DN (CD4-CD8- %T cell, CD27 on unsw mem B cells, IgD on IgD+ CD38dim B cells, CD28 on CD28+ DN (CD4-CD8-) T cell, CD45 on HLA DR + CD8br TBNK, and CCR2 on monocyte. Conversely, osteonecrosis was associated with decreased levels of Myeloid DC %DC, T cell %lymphocyte, CD20 on IgD+ CD38- B cells, CD20 on IgD- CD24- B cells, CD20 on IgD- CD27- B cells, CD3 on EM CD4+ T cell, CD3 on TD CD4+ T cell, CD3 on CD45RA- CD4+ T cell, HVEM on CM CD4+ T cell, HVEM on CD8br T cell, CD28 on secreting Treg, CD28 on resting Treg, CD127 on granulocyte, and CD40 on CD14+ CD16+ monocyte (Figure 3). The analysis revealed no significant pleiotropy or heterogeneity in the IVs of these immunophenotypes, except for HLA DR + T cell %lymphocyte (Supplement 4). Consequently, the protective role of HLA DR + T cell %lymphocyte against osteonecrosis requires further validation. The leave-one-out analysis confirmed the reliability of the data. The scatterplots and leave-one-out plots are shown in Supplement 5.

### 3.3. MR Analysis Between the Gut Microbiota, Immunophenotypes, and Osteonecrosis

This investigation examined the mediating role of immunophenotypes in the causative relationship between osteonecrosis and both the superpathway of sulfate assimilation and cysteine synthesis of the gut microbiota and the genus *Eggerthella*.

**Figure 4:**
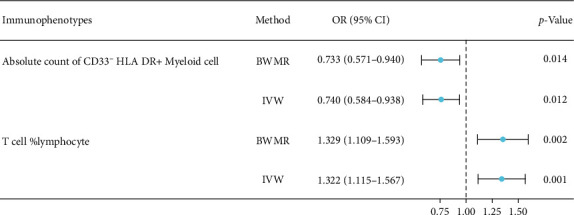
Forest diagram of the causal relationship between the superpathway of sulfate assimilation and cysteine biosynthesis of the gut microbiota and immunophenotypes.

Two-sample MR analysis was used to investigate the causal relationship between the gut microbiota and immune cells. The analysis revealed no causal relationship between the aforementioned 24 immunophenotypes and the genus *Eggerthella*. However, the superpathway of sulfate assimilation and cysteine biosynthesis of the gut microbiota demonstrated causal relationships with both the absolute count of CD33^−^ HLA DR + Myeloid cell and T cell %lymphocyte (the percentage of T cells in lymphocytes), respectively. Specifically, the enhancement of the superpathway of sulfate assimilation and cysteine biosynthesis significantly decreased CD33^−^ HLA DR + Myeloid cell absolute count, while T cell %lymphocyte showed a significant increase (Figure 4). The study findings indicated no substantial pleiotropy or heterogeneity of these IVs (Supplement 6). The leave-one-out method confirmed the reliability of the data. The scatterplots and leave-one-out plots are shown in Supplement 7.

Additional analysis revealed that in the protective mechanism of the superpathway of sulfate assimilation and cysteine biosynthesis against osteonecrosis, the absolute count of CD33^−^ HLA DR + Myeloid cell exhibited a mediation effect of 4.15%, while T cell %lymphocyte demonstrated a mediation effect of 12.16% (Table 1).

### 3.4. Reverse MR Test

**Figure 5:**
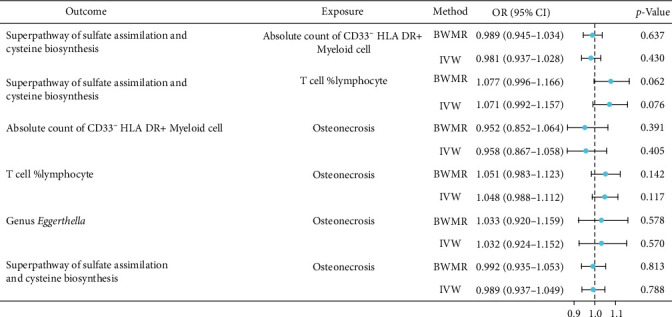
The results of reverse MR.

Two-sample MR analysis was conducted to investigate the causal relationship between the superpathway of sulfate assimilation and cysteine biosynthesis of the gut microbiota and two immunophenotypes: the absolute count of CD33^−^ HLA DR + Myeloid cell and T cell %lymphocyte. The analysis revealed no significant causal associations between these variables. Additionally, the same methodological approach was applied to examine potential causal relationships between osteonecrosis and both the superpathway of sulfate assimilation and cysteine biosynthesis of the gut microbiota as well as the absolute count of CD33^−^ HLA DR + Myeloid cell and T cell %lymphocyte. The results indicated no significant causal effects of osteonecrosis on these parameters (Figure 5). Moreover, subsequent analyses demonstrated that osteonecrosis exhibited no causal relationship with the previously identified 14 gut microbial species and 24 immune cell types (Supplement 8).

## 4. Discussion

This study employed a two-sample, mediating MR analysis to investigate the causal relationship between 412 gut microbial species and osteonecrosis, and the mediation effect of 731 immunophenotypes. The findings revealed that 16 gut microbial species and their metabolic pathways, including genus *Eggerthella* and the superpathway of sulfate assimilation and cysteine biosynthesis of the gut microbiota, and 23 immunophenotypes, such as the absolute count of CD33^−^ HLA DR + Myeloid cell and T cell %lymphocyte, demonstrated a causal relationship with osteonecrosis. A reverse MR analysis indicated that osteonecrosis had no significant causal effect on these factors. Furthermore, the results of mediation analysis demonstrated that the absolute count of CD33^−^ HLA DR + Myeloid cell and T cell %lymphocyte mediated the protective effect of the superpathway of sulfate assimilation and cysteine biosynthesis of the gut microbiota on osteonecrosis. Specifically, the absolute count of CD33^−^ HLA DR + Myeloid cell exhibited a mediation effect of 4.15%, while T cell %lymphocyte showed a mediation effect of 12.16%.

As shown in previous research, genus *Eggerthella* activates Th17 cells in the intestine, which secrete IL-17, subsequently inducing the RANKL/RANK/OPG system and promoting osteoclastogenesis and bone resorption [26–29]. Current literature increasingly suggests that osteonecrosis correlates with extended osteoclast longevity and direct apoptosis of bone marrow stem cells and osteoblasts, as observed in steroid-induced femoral head necrosis [30, 31]. Although specific research on the role of *Eggerthella* in osteonecrosis remains limited, our study indicates that an increase in the abundance of *Eggerthella* contributes to osteonecrosis formation. Additionally, sulfur metabolism plays a critical role in osteonecrosis development. According to recent research, the exhaled breath of patients with medication-related osteonecrosis of the jaw contains elevated concentrations of hydrogen sulfide and methyl mercaptan compared to reference values [32]. Kniha et al. [33] demonstrated increased sulfur levels in rats with osteonecrosis. Yang et al. [34] found significantly lower levels of sulfate in the urine of patients with femoral head necrosis compared to those in healthy individuals; this finding indicated a close relationship between cysteine and methionine metabolism disorders and ONFH. Studies have also observed positive therapeutic effects using a calcium sulfate-calcium phosphate bone graft substitute for ONFH treatment [35]. Moreover, research indicates significantly reduced glutathione (GSH) content in steroid-induced ONFH, while an increased GSH content helps prevent and delay bone necrosis [36–38]. Notably, previous studies have demonstrated that enhancing gut microbiota cysteine synthesis significantly increases GSH biosynthesis levels [39], which is consistent with our findings. Our results indicate that the superpathway of sulfate assimilation and cysteine biosynthesis of the gut microbiota serves as a protective factor against osteonecrosis. These gut microbiota and their metabolic pathways may therefore represent promising targets for future osteonecrosis treatment and prevention.

The immune response plays a crucial role in the development and progression of osteonecrosis and represents a defining characteristic of the disease [40, 41]. Our findings demonstrate that multiple immune cells and immunophenotypes exhibit causal relationships with osteonecrosis. Chen et al. [42] found that T cell subset imbalance contributes to the pathophysiological process of ONFH. Additionally, Li et al. [13] demonstrated that T cell %lymphocyte exerts a protective effect during osteonecrosis occurrence, which is consistent with our findings. T cells influence bone metabolism through osteoprotegerin and RANKL secretion or by modulating the local inflammatory microenvironment. Helper T cells and cytotoxic T lymphocytes contribute to osteonecrosis progression, while regulatory T cells (Treg) mitigate its advancement [43]. Our results indicate that CD28 on resting Treg and CD28 on secreting Treg function as protective factors, suggesting that the protective effect of Treg cells is mediated by CD28. Furthermore, our research revealed the varying effects of different B cell immune phenotypes on osteonecrosis. Alcohol abuse activates TLR4-related signaling pathways, leading to systemic inflammation and increased TNF-α production. The TNF-α family regulates peripheral B cell activity and bone marrow B cell development, promoting ONFH [43–45]. Additional studies demonstrate that pre-B cells, plasma cells, and immature B cells inhibit osteoclast differentiation, while activated B cells release RANKL, promoting osteoclast formation [46, 47].

These effects likely manifest through immunophenotypes, including CD20 on IgD+ CD38- B cells, CD20 on IgD-CD24- B cells, CD20 on IgD-CD27- B cells, CD27 on unsw mem B cells, and IgD on IgD+ CD38dim B cells. Thus, distinct B cell subtypes regulate bone metabolism by influencing the formation of osteoblasts and osteoclasts. Previous research also indicates that myeloid dendritic cells protect against osteonecrosis by regulating immune responses and activating T cells [12, 48, 49], as confirmed by our findings regarding Myeloid DC %DC. However, research on CD33^−^ HLA DR + Myeloid cell in osteonecrosis remains limited. Our findings suggest that these cells can trigger osteonecrosis occurrence. Additionally, our results demonstrate that in the protective mechanism of the superpathway of sulfate assimilation and cysteine biosynthesis of the gut microbiota against osteonecrosis, the absolute count of CD33^−^ HLA DR + Myeloid cell and T cell %lymphocyte exhibited mediation effects of 4.15% and 12.16%, respectively. In summary, immune cells and related immunophenotypes significantly influence osteonecrosis, offering important perspectives for mechanistic studies and treatment approaches.

This study employed MR analysis to investigate causal relationships between gut microbiota, immune cells, and osteonecrosis. The investigation of these relationships through a traditional approach requires substantial time and financial investment. In contrast, MR analysis presents an efficient alternative. Because confounding factors typically show no correlation with genetic variants, and variants are randomly assigned during meiosis, MR studies are identical to randomized controlled trials. However, this study has several limitations. First, the threshold of IVs was set at 1 × 10^−5^ to ensure sufficient IVs. Although this threshold has been used in numerous studies, it is less stringent than the conventional threshold of *p* < 5 × 10^−8^. Additionally, the findings were not adjusted for multiple comparisons, which may increase the risk of Type I errors, as the purpose of this exploratory analysis was to identify potential positive results. Thus, IV estimates may provide causal effects only under certain assumptions. Second, the absence of individual case information prevented population stratification analysis. Finally, the data originated from European populations, necessitating further research to confirm universality across other populations. Additional studies are required to elucidate the detailed mechanisms through which the gut microbiota influence osteonecrosis development and progression and the mediating role of immunophenotypes. For example, we can explore how the superpathway of sulfate assimilation and cysteine biosynthesis of the gut microbiota affects the local immune environment of bones and joints, causing dysregulation of immune cell expression, thereby causing functional imbalance of local bone tissue microvessels, osteoblasts, osteoclasts, bone marrow stem cells, and chondrocytes, which ultimately leads to osteonecrosis.

## 5. Conclusions

In conclusion, this study established causal relationships between the gut microbiota, immunophenotypes, and osteonecrosis. The research highlighted the protective effect of the superpathway of sulfate assimilation and cysteine biosynthesis of the gut microbiota against osteonecrosis. The absolute count of CD33^−^ HLA DR + Myeloid cell and T cell %lymphocyte demonstrated mediation effects of 4.15% and 12.16%, respectively, in this protective mechanism. These findings provide valuable insights for developing osteonecrosis prevention and treatment strategies.

## Figures and Tables

**Table 1:** Mediation effect of the absolute count of CD33^−^ HLA DR + Myeloid cell and the percentage of T cells in lymphocytes.

Immunophenotypes	Path a	Path b	Total effect	Indirect effect	Proportion mediated (%)
	β1	β2	β3	β12	
The absolute count of CD33^−^ HLA DR + Myeloid cell	−0.300	0.053	−0.383	−0.016	4.15
The percentage of T cells in lymphocytes	0.279	−0.167	−0.383	−0.046	12.16

## Data Availability

The datasets generated and/or analyzed during the current study are available in the FinnGen repository (https://www.finngen.fi/en) and GWAS Catalog (https://www.ebi.ac.uk/gwas/downloads/summary-statistics).

## Nomenclature

MR: Mendelian randomization
GWAS: Genome-wide association study
ONFH: Osteonecrosis of the femoral head
IVs: Instrumental variables
SNPs: Single nucleotide polymorphisms
BWMR: Bayesian weighted Mendelian randomization
IVW: Inverse variance weighted
OR: Odds ratio
CI: Confidence interval
Treg: Regulatory T cells
GSH: Glutathione.

## Supporting Information

Supporting Information Supplement 1: The STROBE-MR checklist of recommended items to address in reports of Mendelian randomization studies. Supplement 2: The significant pleiotropy or heterogeneity of IVs in the MR analysis using gut microbiota as the exposure and osteonecrosis as the outcome. Supplement 3: The scatterplots and leave-one-out plots in the MR analysis using gut microbiota as the exposure and osteonecrosis as the outcome. Supplement 4: The significant pleiotropy or heterogeneity of IVs in the MR analysis using immune cells as the exposure and osteonecrosis as the outcome. Supplement 5: The scatterplots and leave-one-out plots in the MR analysis using immune cells as the exposure and osteonecrosis as the outcome. Supplement 6: The significant pleiotropy or heterogeneity of IVs in the MR analysis using gut microbiota as the exposure and immune cells as the outcome. Supplement 7: The scatterplots and leave-one-out plots in the MR analysis using gut microbiota as the exposure and immune cells as the outcome. Supplement 8: The results of the MR analysis using osteonecrosis as the exposure and gut microbiota and immune cells as the outcomes.

## References

[1] Motta F., Timilsina S., Gershwin M. E., Selmi C. (2022). Steroid-Induced Osteonecrosis. *Journal of Translational Autoimmunity*.

[2] Lafforgue P. (2006). Pathophysiology and Natural History of Avascular Necrosis of Bone. *Joint Bone Spine*.

[3] Mucke T., Krestan C. R., Mitchell D. A., Kirschke J. S., Wutzl A. (2016). Bisphosphonate and Medication-Related Osteonecrosis of the Jaw: A Review. *Seminars in Musculoskeletal Radiology*.

[4] Sender R., Fuchs S., Milo R. (2016). Revised Estimates for the Number of Human and Bacteria Cells in the Body. *PLOS Biology*.

[5] Hofman D., Kudla U., Miqdady M., Nguyen T. V. H., Moran-Ramos S., Vandenplas Y. (2022). Faecal Microbiota in Infants and Young Children With Functional Gastrointestinal Disorders: A Systematic Review. *Nutrients*.

[6] Di J., Xi Y., Wu Y. (2024). Gut Microbiota Metabolic Pathways: Key Players in Knee Osteoarthritis Development. *Experimental Gerontology*.

[7] Yu X.-H., Yang Y.-Q., Cao R.-R., Bo L., Lei S.-F. (2021). The Causal Role of Gut Microbiota in Development of Osteoarthritis. *Osteoarthritis and Cartilage*.

[8] Zheng Q.-Y., Tao Y., Geng L., Ren P., Ni M., Zhang G.-Q. (2024). Non-Traumatic Osteonecrosis of the Femoral Head Induced by Steroid and Alcohol Exposure is Associated With Intestinal Flora Alterations and Metabolomic Profiles. *Journal of Orthopaedic Surgery and Research*.

[9] Chen C.-Y., Rao S.-S., Yue T. (2022). Glucocorticoid-Induced Loss of Beneficial Gut Bacterial Extracellular Vesicles is Associated With the Pathogenesis of Osteonecrosis. *Science Advances*.

[10] Kim A.-R., Jeon S.-G., Kim H.-R. (2024). Preventive and Therapeutic Effects of Lactiplantibacillus Plantarum HD02 and MD159 Through Mast Cell Degranulation Inhibition in Mouse Models of Atopic Dermatitis. *Nutrients*.

[11] Acevedo-Román A., Pagán-Zayas N., Velázquez-Rivera L. I., Torres-Ventura A. C., Godoy-Vitorino F. (2024). Insights Into Gut Dysbiosis: Inflammatory Diseases, Obesity, and Restoration Approaches. *International Journal of Molecular Sciences*.

[12] Wang C., Zhu Y., Pan D. (2024). Identifying the Causal Relationship Between Immune Factors and Osteonecrosis: A Two-Sample Mendelian Randomization Study. *Scientific Reports*.

[13] Li W., Xu J.-W., Chai J.-L. (2024). Complex Causal Association Between Genetically Predicted 731 Immunocyte Phenotype and Osteonecrosis: A Bidirectional Two-Sample Mendelian Randomization Analysis. *International Journal of Surgery*.

[14] Li Y., Wan S., Liu J., Huang Y., Jiang L. (2024). Causal Relationship Between Dietary Intake and IgA Nephropathy: A Mendelian Randomization Study. *Frontiers in Nutrition*.

[15] Dan J., Lu H. M., Zhou X., Wang H. Y., Wang J. H. (2024). Association of Autoimmune Diseases With the Occurrence of Osteoarthritis: A Gene Expression and Mendelian Randomization Study. *Frontiers in Medicine*.

[16] Liu J., Jiang J., Li Y. (2024). Effects of FGF21 Overexpression in Osteoporosis and Bone Mineral Density: A Two-Sample, Mediating Mendelian Analysis. *Frontiers in Endocrinology*.

[17] Guo J., Yang P., Wang J. H. (2024). Blood Metabolites, Neurocognition and Psychiatric Disorders: A Mendelian Randomization Analysis to Investigate Causal Pathways. *Translational Psychiatry*.

[18] Swanson S. A., Tiemeier H., Ikram M. A., Hernán M. A. (2017). Nature as a Trialist?: Deconstructing the Analogy Between Mendelian Randomization and Randomized Trials. *Epidemiology*.

[19] Kurki M. I., Karjalainen J., Palta P. (2023). FinnGen Provides Genetic Insights From a Well-Phenotyped Isolated Population. *Nature*.

[20] Lopera-Maya E. A., Kurilshikov A., Van Der Graaf A. (2022). Effect of Host Genetics on the Gut Microbiome in 7,738 Participants of the Dutch Microbiome Project. *Nature Genetics*.

[21] Orru V., Steri M., Sidore C. (2020). Complex Genetic Signatures in Immune Cells Underlie Autoimmunity and Inform Therapy. *Nature Genetics*.

[22] Miao J., Xu Y. (2025). Causal Effects of Smoking, Alcohol, Coffee, and Tea Intake on Gynecologic Cancers: A Mendelian Randomization Study. *International Journal of Women’s Health*.

[23] Sun K., Ming Y., Wu Y. (2023). The Genetic Causal Association Between Educational Attainment and Risk of 12 Common Musculoskeletal Disorders: A Two-Sample Mendelian Randomization. *Orthopaedic Surgery*.

[24] Li W., Chai J.-L., Li Z. (2023). No Evidence of Genetic Causality Between Diabetes and Osteonecrosis: A Bidirectional Two-Sample Mendelian Randomization Analysis. *Journal of Orthopaedic Surgery and Research*.

[25] Zhao J., Ming J., Hu X., Chen G., Liu J., Yang C. (2020). Bayesian Weighted Mendelian Randomization for Causal Inference Based on Summary Statistics. *Bioinformatics*.

[26] Wu D., Cline-Smith A., Shashkova E., Perla A., Katyal A., Aurora R. (2021). T-Cell Mediated Inflammation in Postmenopausal Osteoporosis. *Frontiers in Immunology*.

[27] Ono T., Hayashi M., Sasaki F., Nakashima T. (2020). RANKL Biology: Bone Metabolism, the Immune System, and Beyond. *Inflammation and Regeneration*.

[28] Qiao X., Li X., Wang Z. (2024). Gut Microbial Community and Fecal Metabolomic Signatures in Different Types of Osteoporosis Animal Models. *Aging (Albany NY)*.

[29] Alexander M., Ang Q. Y., Nayak R. R. (2022). Human Gut Bacterial Metabolism Drives Th17 Activation and Colitis. *Cell Host & Microbe*.

[30] Wang L., You X., Zhang L., Zhang C., Zou W. (2022). Mechanical Regulation of Bone Remodeling. *Bone Research*.

[31] Xu H., Wang L., Zhu X., Zhang H., Chen H., Zhang H. (2024). Jintiange Capsule Ameliorates Glucocorticoid-Induced Osteonecrosis of the Femoral Head in Rats by Regulating the Activity and Differentiation of BMSCs. *Journal of Traditional and Complementary Medicine*.

[32] Choi Y., Kim J., Rhee Y., Park J. H., Nam W., Park W. (2024). The Assessment of Halitosis With a New Screening Tool in Medication-Related Osteonecrosis of the Jaw. *Clinical Oral Investigations*.

[33] Kniha K., Buhl E. M., Al-Sibai F. (2023). Results of Thermal Osteonecrosis for Implant Removal on Electron Microscopy, Implant Stability, and Radiographic Parameters - A Rat Study. *Head & Face Medicine*.

[34] Yang G., Zhao G., Zhang J. (2019). Global Urinary Metabolic Profiling of the Osteonecrosis of the Femoral Head Based on UPLC–QTOF/MS. *Metabolomics*.

[35] Sionek A., Czwojdzinski A., Kowalczewski J. (2018). Hip Osteonecroses Treated With Calcium Sulfate-Calcium Phosphate Bone Graft Substitute Have Different Results According to the Cause of Osteonecrosis: Alcohol Abuse or Corticosteroid-Induced. *International Orthopaedics*.

[36] Sun F., Zhou J. L., Liu Z. L., Jiang Z. W., Peng H. (2022). Dexamethasone Induces Ferroptosis Via P53/SLC7A11/GPX4 Pathway in Glucocorticoid-Induced Osteonecrosis of the Femoral Head. *Biochemical and Biophysical Research Communications*.

[37] Li G.-Y., Feng Y., Cheng T. S., Yin J.-M., Zhang C.-Q. (2013). Edaravone, a Novel Free Radical Scavenger, Prevents Steroid-Induced Osteonecrosis in Rabbits. *Rheumatology*.

[38] Li J., Ge Z., Fan L., Wang K. (2017). Protective Effects of Molecular Hydrogen on Steroid-Induced Osteonecrosis in Rabbits Via Reducing Oxidative Stress and Apoptosis. *BMC Musculoskeletal Disorders*.

[39] Jun-Xian W., Jing Z., Hui W., Zhi-Min L. (2017). Effect of Strengthening the Cysteine Synthetic Pathway on Glutathione Biosynthesis in *Escherichia coli*. *Journal of East China University of Science and Technology*.

[40] Leuti A., Fazio D., Fava M., Piccoli A., Oddi S., Maccarrone M. (2020). Bioactive Lipids, Inflammation and Chronic Diseases. *Advanced Drug Delivery Reviews*.

[41] Lu Y., Pei Y., Gao Y. M., Zhao F. F., Wang L., Zhang Y. (2024). Unraveling the Genetic Basis of the Causal Association Between Inflammatory Cytokines and Osteonecrosis. *Frontiers in Endocrinology*.

[42] Chen C., Zhao X., Luo Y. (2022). Imbalanced T-Cell Subsets May Facilitate the Occurrence of Osteonecrosis of the Femoral Head. *Journal of Inflammation Research*.

[43] Meng C., Qi B., Luo H. (2024). Exploring the Genetic Association Between Immune Cells and Susceptibility to Osteonecrosis Using Large-Scale Population Data. *Heliyon*.

[44] Nanes M. S. (2003). Tumor Necrosis Factor-α: Molecular and Cellular Mechanisms in Skeletal Pathology. *Gene*.

[45] James J. A., Harley J. B. (1998). B-Cell Epitope Spreading in Autoimmunity. *Immunological Reviews*.

[46] Weitzmann M. N. (2014). T-Cells and B-Cells in Osteoporosis. *Current Opinion in Endocrinology, Diabetes & Obesity*.

[47] Toni R., Di Conza G., Barbaro F. (2020). Microtopography of Immune Cells in Osteoporosis and Bone Lesions by Endocrine Disruptors. *Frontiers in Immunology*.

[48] Banchereau J., Briere F., Caux C. (2000). Immunobiology of Dendritic Cells. *Annual Review of Immunology*.

[49] Steinman R. M., Hawiger D., Nussenzweig M. C. (2003). Tolerogenic Dendritic Cells. *Annual Review of Immunology*.

