# Supplementary figures and images for: Mediating Effects of Immunophenotypes on the Causal Relationship of Gut Microbiota and Their Metabolic Pathways With Osteonecrosis: A Mendelian Randomization Analysis
Source: Mediators Inflamm. 2025 Oct 28;2025:9323113. doi: 10.1155/mi/9323113 (PMC12585845; doi:10.1155/mi/9323113)

# MR Test

- Inverse variance weighted
- MR Egger
- Simple mode
- Weighted median
- Weighted mode

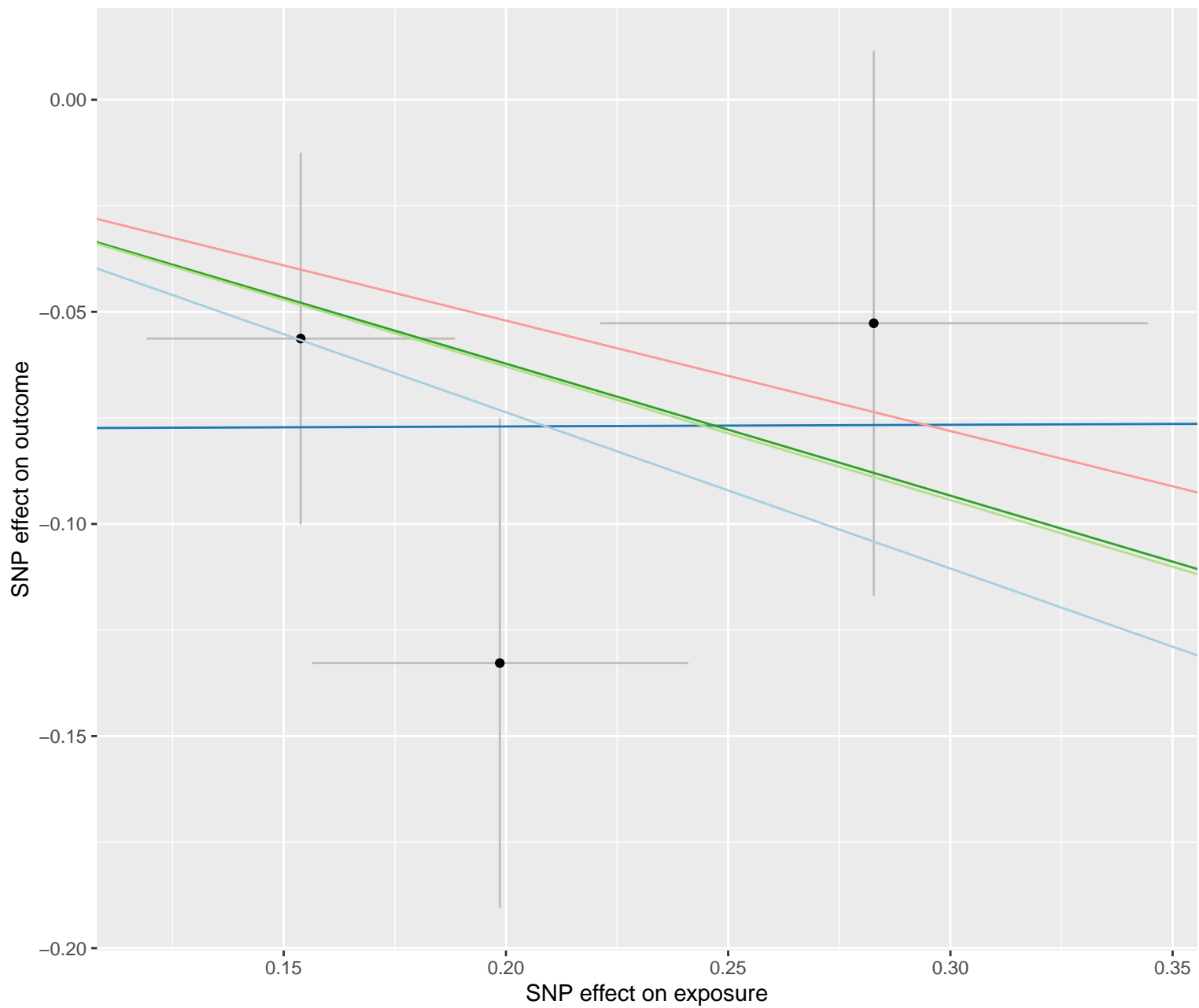

Supplement: Supporting Information — Supplement 1: The STROBE-MR checklist of recommended items to address in reports of Mendelian randomization studies. Supplement 2: The significant pleiotropy or heterogeneity of IVs in the MR analysis using gut microbiota as the exposure and osteonecrosis as the outcome. Supplement 3: The scatterplots and leave-one-out plots in the MR analysis using gut microbiota as the exposure and osteonecrosis as the outcome. Supplement 4: The significant pleiotropy or heterogeneity of IVs in the MR analysis using immune cells as the exposure and osteonecrosis as the outcome. Supplement 5: The scatterplots and leave-one-out plots in the MR analysis using immune cells as the exposure and osteonecrosis as the outcome. Supplement 6: The significant pleiotropy or heterogeneity of IVs in the MR analysis using gut microbiota as the exposure and immune cells as the outcome. Supplement 7: The scatterplots and leave-one-out plots in the MR analysis using gut microbiota as the exposure and immune cells as the outcome. Supplement 8: The results of the MR analysis using osteonecrosis as the exposure and gut microbiota and immune cells as the outcomes. [file 9323113.f1.zip › Supplement 3/GCST90027472/scatter.pdf]

rs2236141

rs4557268

rs79950843

All

-0.75

-0.50

-0.25

0.00

MR leave-one-out sensitivity analysis for  
'exposure' on 'outcome'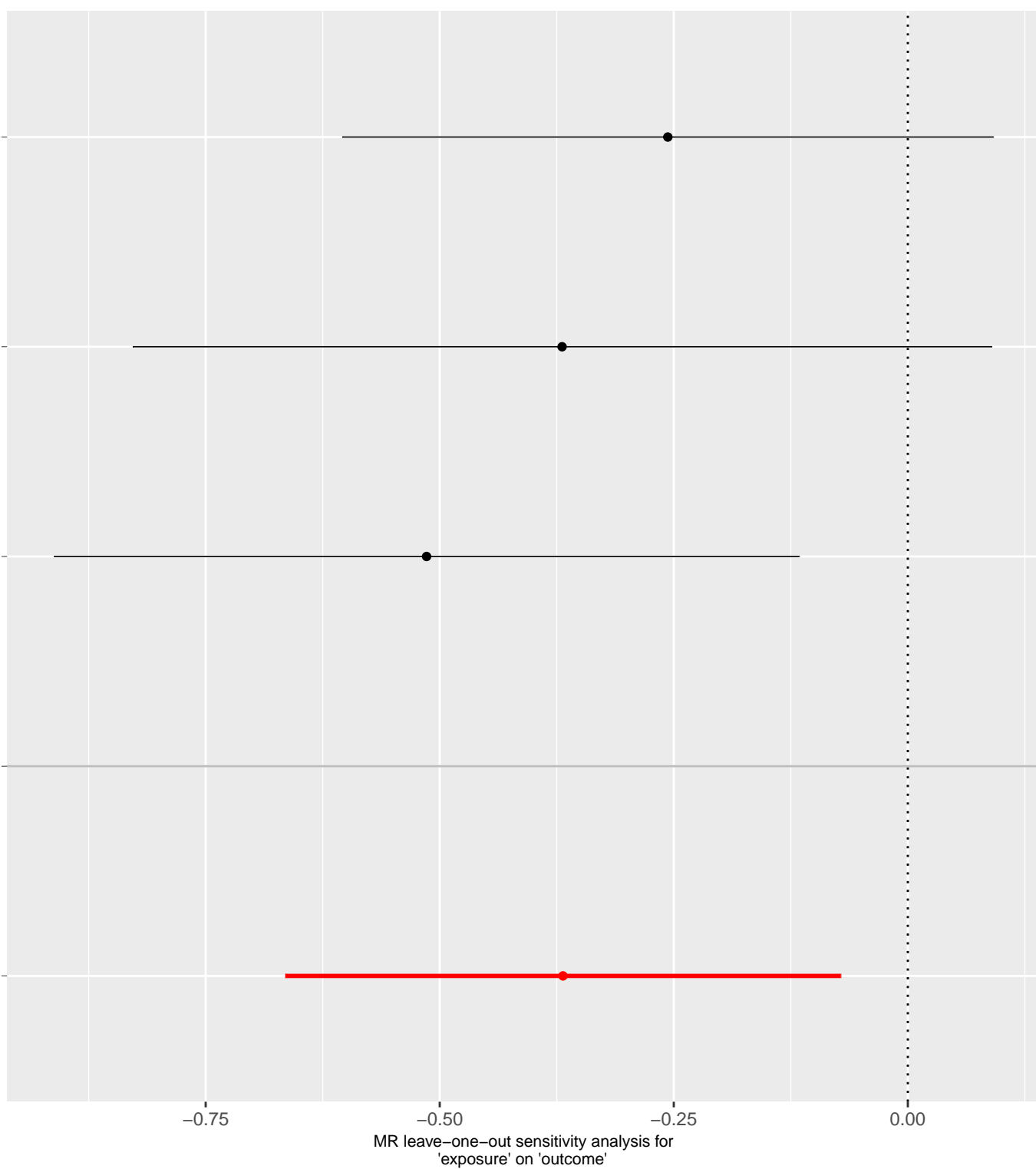

Supplement: Supporting Information — Supplement 1: The STROBE-MR checklist of recommended items to address in reports of Mendelian randomization studies. Supplement 2: The significant pleiotropy or heterogeneity of IVs in the MR analysis using gut microbiota as the exposure and osteonecrosis as the outcome. Supplement 3: The scatterplots and leave-one-out plots in the MR analysis using gut microbiota as the exposure and osteonecrosis as the outcome. Supplement 4: The significant pleiotropy or heterogeneity of IVs in the MR analysis using immune cells as the exposure and osteonecrosis as the outcome. Supplement 5: The scatterplots and leave-one-out plots in the MR analysis using immune cells as the exposure and osteonecrosis as the outcome. Supplement 6: The significant pleiotropy or heterogeneity of IVs in the MR analysis using gut microbiota as the exposure and immune cells as the outcome. Supplement 7: The scatterplots and leave-one-out plots in the MR analysis using gut microbiota as the exposure and immune cells as the outcome. Supplement 8: The results of the MR analysis using osteonecrosis as the exposure and gut microbiota and immune cells as the outcomes. [file 9323113.f1.zip › Supplement 3/GCST90027472/sensitivity-analysis.pdf]

# MR Test

- Inverse variance weighted
- MR Egger
- Simple mode
- Weighted median
- Weighted mode

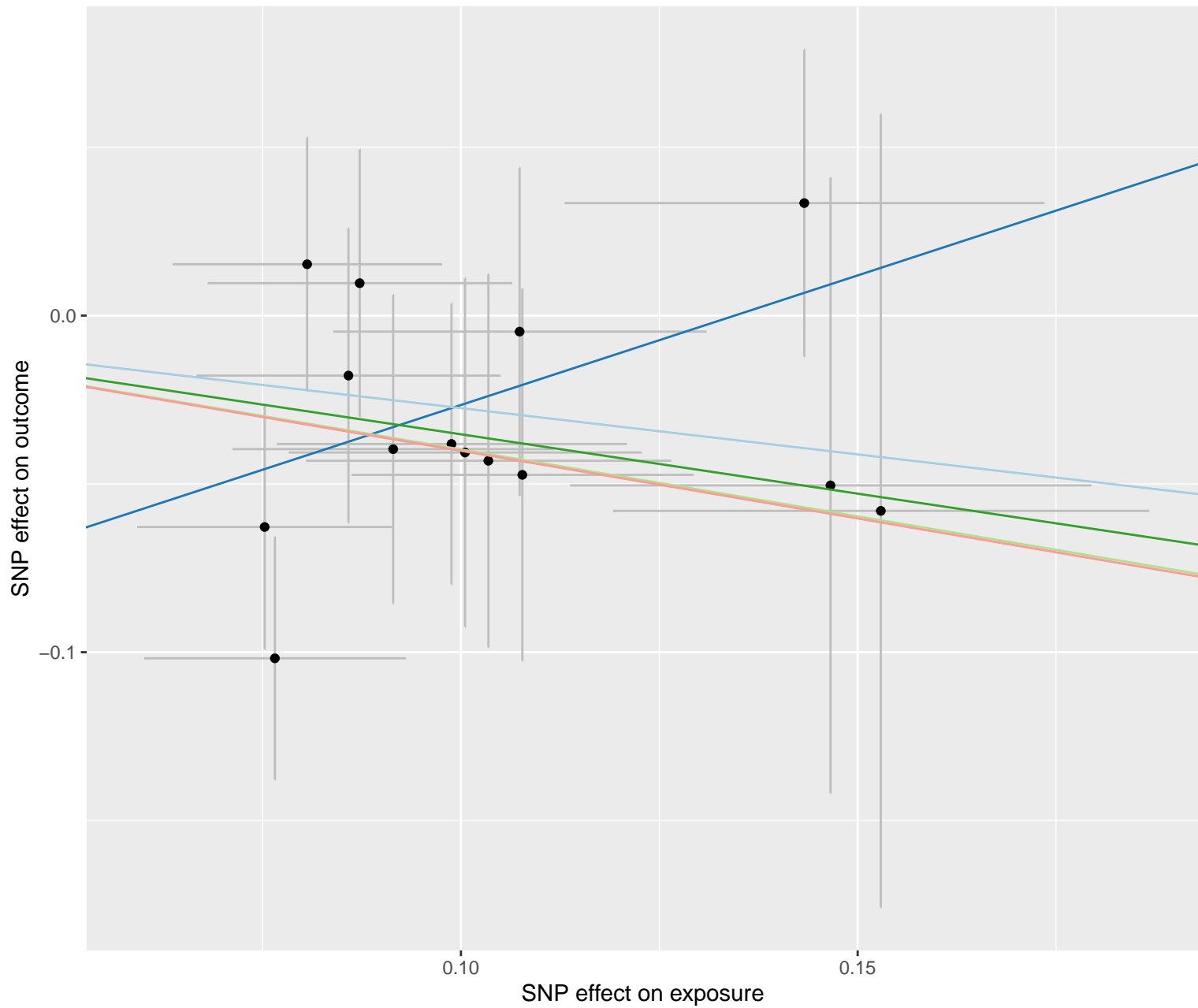

Supplement: Supporting Information — Supplement 1: The STROBE-MR checklist of recommended items to address in reports of Mendelian randomization studies. Supplement 2: The significant pleiotropy or heterogeneity of IVs in the MR analysis using gut microbiota as the exposure and osteonecrosis as the outcome. Supplement 3: The scatterplots and leave-one-out plots in the MR analysis using gut microbiota as the exposure and osteonecrosis as the outcome. Supplement 4: The significant pleiotropy or heterogeneity of IVs in the MR analysis using immune cells as the exposure and osteonecrosis as the outcome. Supplement 5: The scatterplots and leave-one-out plots in the MR analysis using immune cells as the exposure and osteonecrosis as the outcome. Supplement 6: The significant pleiotropy or heterogeneity of IVs in the MR analysis using gut microbiota as the exposure and immune cells as the outcome. Supplement 7: The scatterplots and leave-one-out plots in the MR analysis using gut microbiota as the exposure and immune cells as the outcome. Supplement 8: The results of the MR analysis using osteonecrosis as the exposure and gut microbiota and immune cells as the outcomes. [file 9323113.f1.zip › Supplement 3/GCST90027501/scatter.pdf]

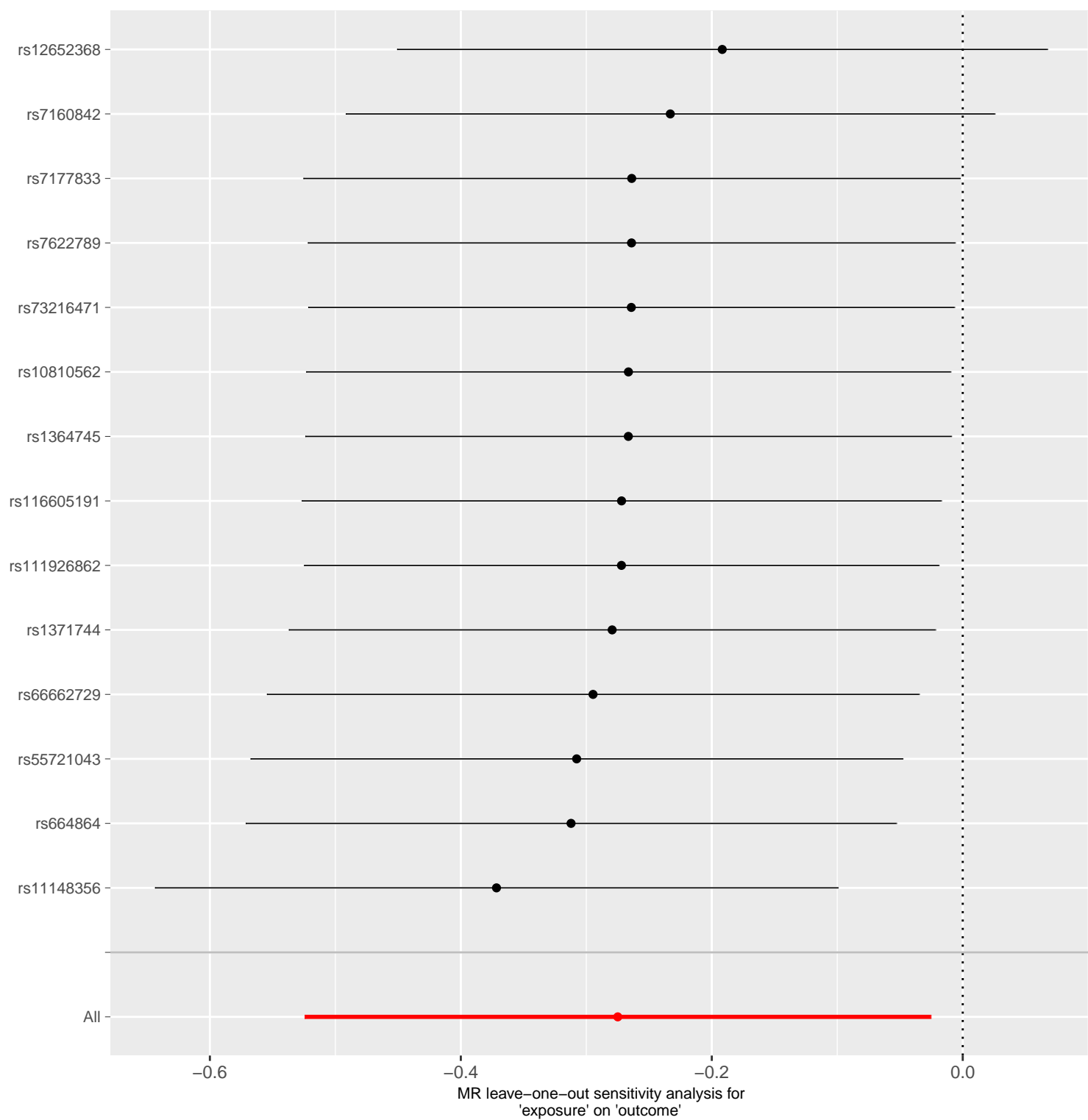

Supplement: Supporting Information — Supplement 1: The STROBE-MR checklist of recommended items to address in reports of Mendelian randomization studies. Supplement 2: The significant pleiotropy or heterogeneity of IVs in the MR analysis using gut microbiota as the exposure and osteonecrosis as the outcome. Supplement 3: The scatterplots and leave-one-out plots in the MR analysis using gut microbiota as the exposure and osteonecrosis as the outcome. Supplement 4: The significant pleiotropy or heterogeneity of IVs in the MR analysis using immune cells as the exposure and osteonecrosis as the outcome. Supplement 5: The scatterplots and leave-one-out plots in the MR analysis using immune cells as the exposure and osteonecrosis as the outcome. Supplement 6: The significant pleiotropy or heterogeneity of IVs in the MR analysis using gut microbiota as the exposure and immune cells as the outcome. Supplement 7: The scatterplots and leave-one-out plots in the MR analysis using gut microbiota as the exposure and immune cells as the outcome. Supplement 8: The results of the MR analysis using osteonecrosis as the exposure and gut microbiota and immune cells as the outcomes. [file 9323113.f1.zip › Supplement 3/GCST90027501/sensitivity-analysis.pdf]

# MR Test

- Inverse variance weighted
- MR Egger
- Simple mode
- Weighted median
- Weighted mode

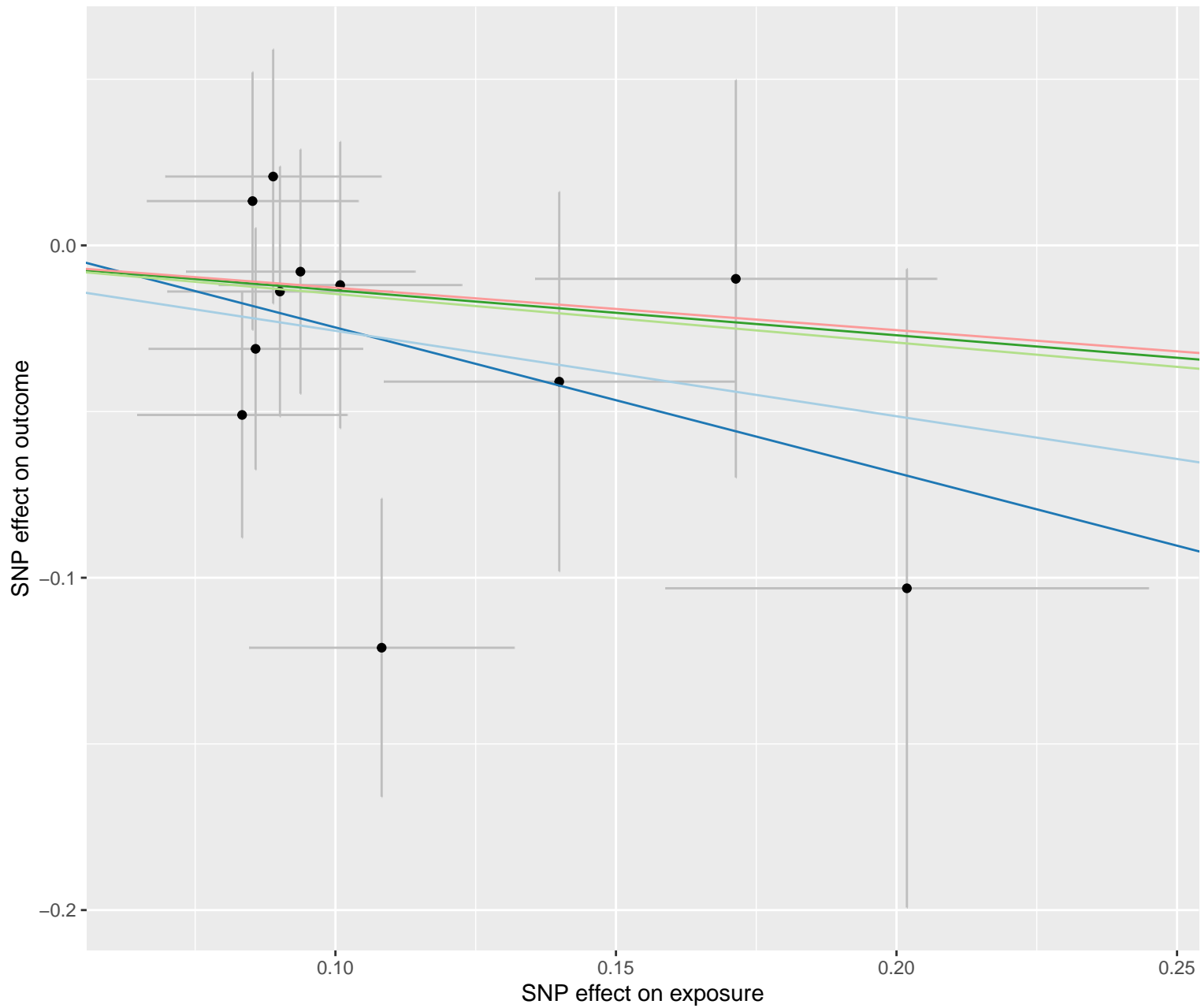

Supplement: Supporting Information — Supplement 1: The STROBE-MR checklist of recommended items to address in reports of Mendelian randomization studies. Supplement 2: The significant pleiotropy or heterogeneity of IVs in the MR analysis using gut microbiota as the exposure and osteonecrosis as the outcome. Supplement 3: The scatterplots and leave-one-out plots in the MR analysis using gut microbiota as the exposure and osteonecrosis as the outcome. Supplement 4: The significant pleiotropy or heterogeneity of IVs in the MR analysis using immune cells as the exposure and osteonecrosis as the outcome. Supplement 5: The scatterplots and leave-one-out plots in the MR analysis using immune cells as the exposure and osteonecrosis as the outcome. Supplement 6: The significant pleiotropy or heterogeneity of IVs in the MR analysis using gut microbiota as the exposure and immune cells as the outcome. Supplement 7: The scatterplots and leave-one-out plots in the MR analysis using gut microbiota as the exposure and immune cells as the outcome. Supplement 8: The results of the MR analysis using osteonecrosis as the exposure and gut microbiota and immune cells as the outcomes. [file 9323113.f1.zip › Supplement 3/GCST90027512/scatter.pdf]

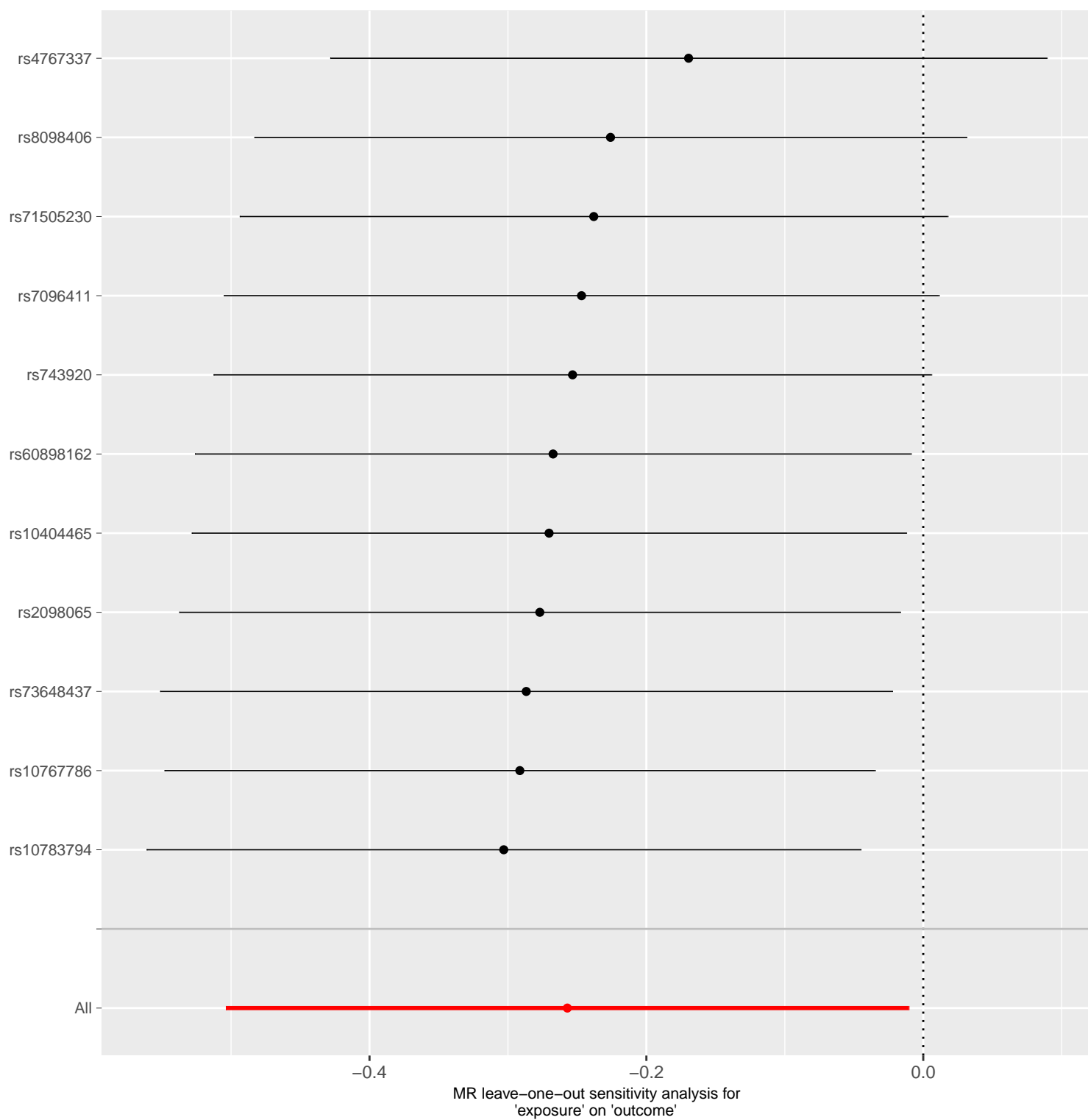

Supplement: Supporting Information — Supplement 1: The STROBE-MR checklist of recommended items to address in reports of Mendelian randomization studies. Supplement 2: The significant pleiotropy or heterogeneity of IVs in the MR analysis using gut microbiota as the exposure and osteonecrosis as the outcome. Supplement 3: The scatterplots and leave-one-out plots in the MR analysis using gut microbiota as the exposure and osteonecrosis as the outcome. Supplement 4: The significant pleiotropy or heterogeneity of IVs in the MR analysis using immune cells as the exposure and osteonecrosis as the outcome. Supplement 5: The scatterplots and leave-one-out plots in the MR analysis using immune cells as the exposure and osteonecrosis as the outcome. Supplement 6: The significant pleiotropy or heterogeneity of IVs in the MR analysis using gut microbiota as the exposure and immune cells as the outcome. Supplement 7: The scatterplots and leave-one-out plots in the MR analysis using gut microbiota as the exposure and immune cells as the outcome. Supplement 8: The results of the MR analysis using osteonecrosis as the exposure and gut microbiota and immune cells as the outcomes. [file 9323113.f1.zip › Supplement 3/GCST90027512/sensitivity-analysis.pdf]

# MR Test

- Inverse variance weighted
- MR Egger
- Simple mode
- Weighted median
- Weighted mode

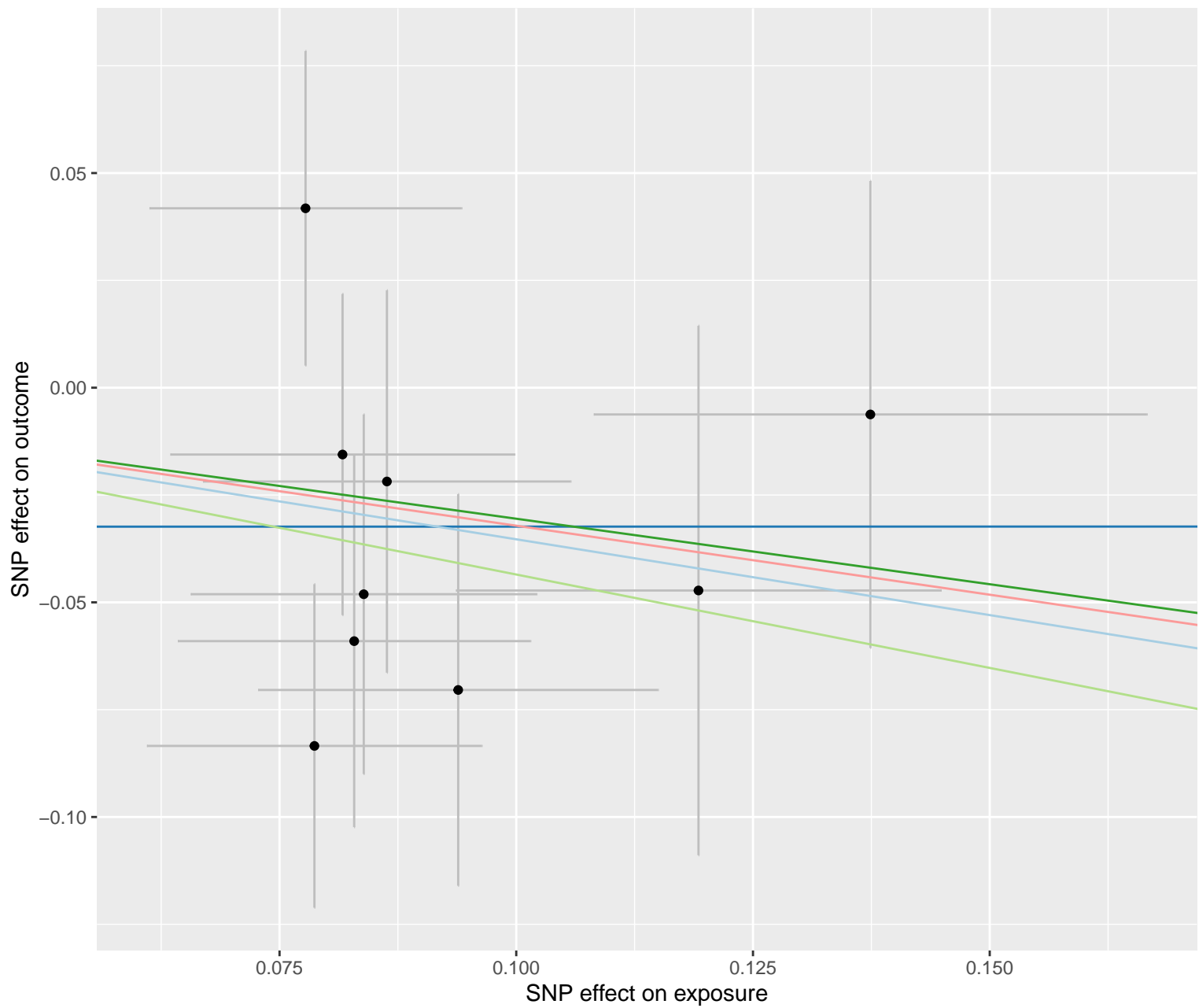

Supplement: Supporting Information — Supplement 1: The STROBE-MR checklist of recommended items to address in reports of Mendelian randomization studies. Supplement 2: The significant pleiotropy or heterogeneity of IVs in the MR analysis using gut microbiota as the exposure and osteonecrosis as the outcome. Supplement 3: The scatterplots and leave-one-out plots in the MR analysis using gut microbiota as the exposure and osteonecrosis as the outcome. Supplement 4: The significant pleiotropy or heterogeneity of IVs in the MR analysis using immune cells as the exposure and osteonecrosis as the outcome. Supplement 5: The scatterplots and leave-one-out plots in the MR analysis using immune cells as the exposure and osteonecrosis as the outcome. Supplement 6: The significant pleiotropy or heterogeneity of IVs in the MR analysis using gut microbiota as the exposure and immune cells as the outcome. Supplement 7: The scatterplots and leave-one-out plots in the MR analysis using gut microbiota as the exposure and immune cells as the outcome. Supplement 8: The results of the MR analysis using osteonecrosis as the exposure and gut microbiota and immune cells as the outcomes. [file 9323113.f1.zip › Supplement 3/GCST90027545/scatter.pdf]

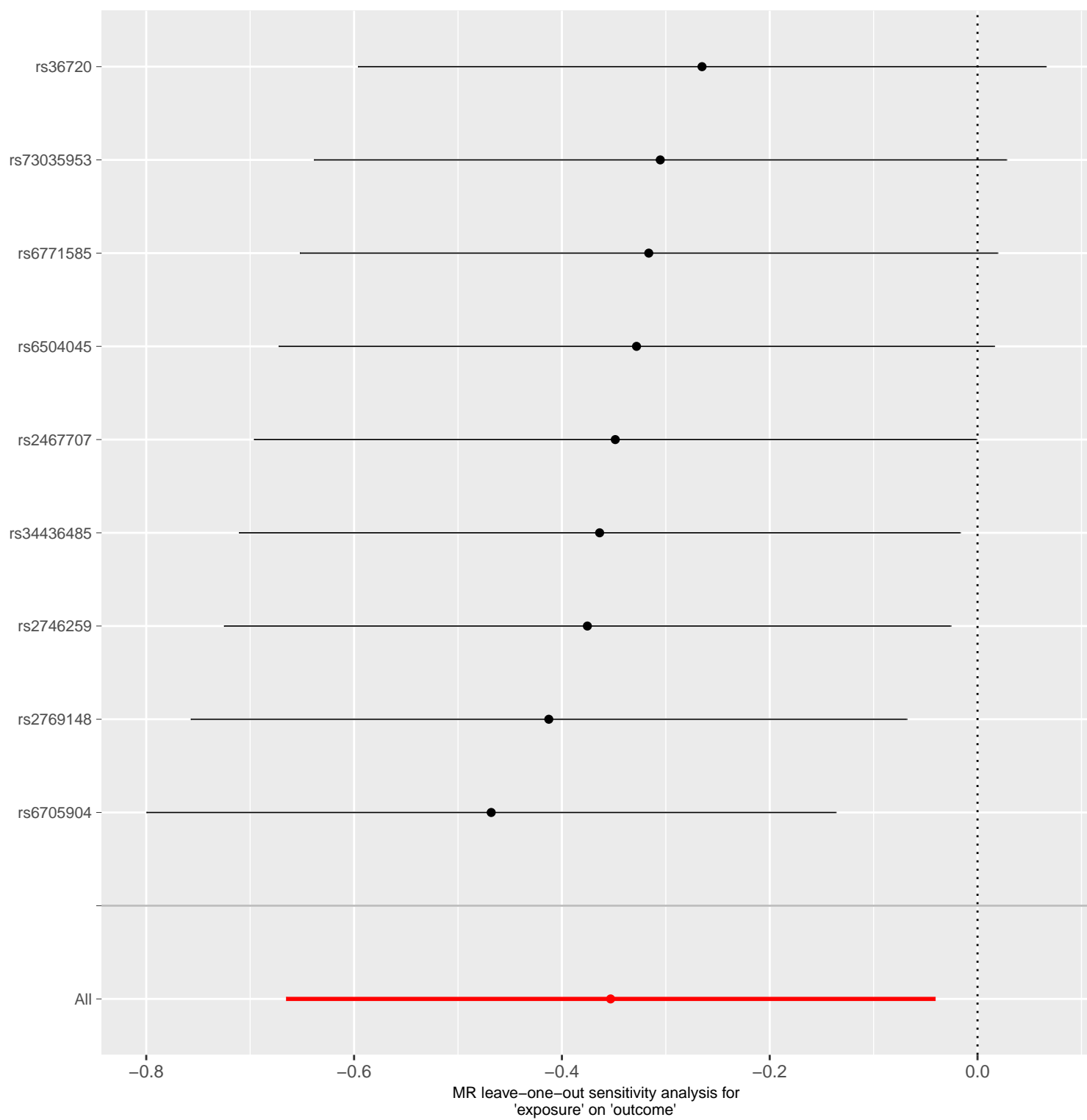

Supplement: Supporting Information — Supplement 1: The STROBE-MR checklist of recommended items to address in reports of Mendelian randomization studies. Supplement 2: The significant pleiotropy or heterogeneity of IVs in the MR analysis using gut microbiota as the exposure and osteonecrosis as the outcome. Supplement 3: The scatterplots and leave-one-out plots in the MR analysis using gut microbiota as the exposure and osteonecrosis as the outcome. Supplement 4: The significant pleiotropy or heterogeneity of IVs in the MR analysis using immune cells as the exposure and osteonecrosis as the outcome. Supplement 5: The scatterplots and leave-one-out plots in the MR analysis using immune cells as the exposure and osteonecrosis as the outcome. Supplement 6: The significant pleiotropy or heterogeneity of IVs in the MR analysis using gut microbiota as the exposure and immune cells as the outcome. Supplement 7: The scatterplots and leave-one-out plots in the MR analysis using gut microbiota as the exposure and immune cells as the outcome. Supplement 8: The results of the MR analysis using osteonecrosis as the exposure and gut microbiota and immune cells as the outcomes. [file 9323113.f1.zip › Supplement 3/GCST90027545/sensitivity-analysis.pdf]

# MR Test

- Inverse variance weighted
- MR Egger
- Simple mode
- Weighted median
- Weighted mode

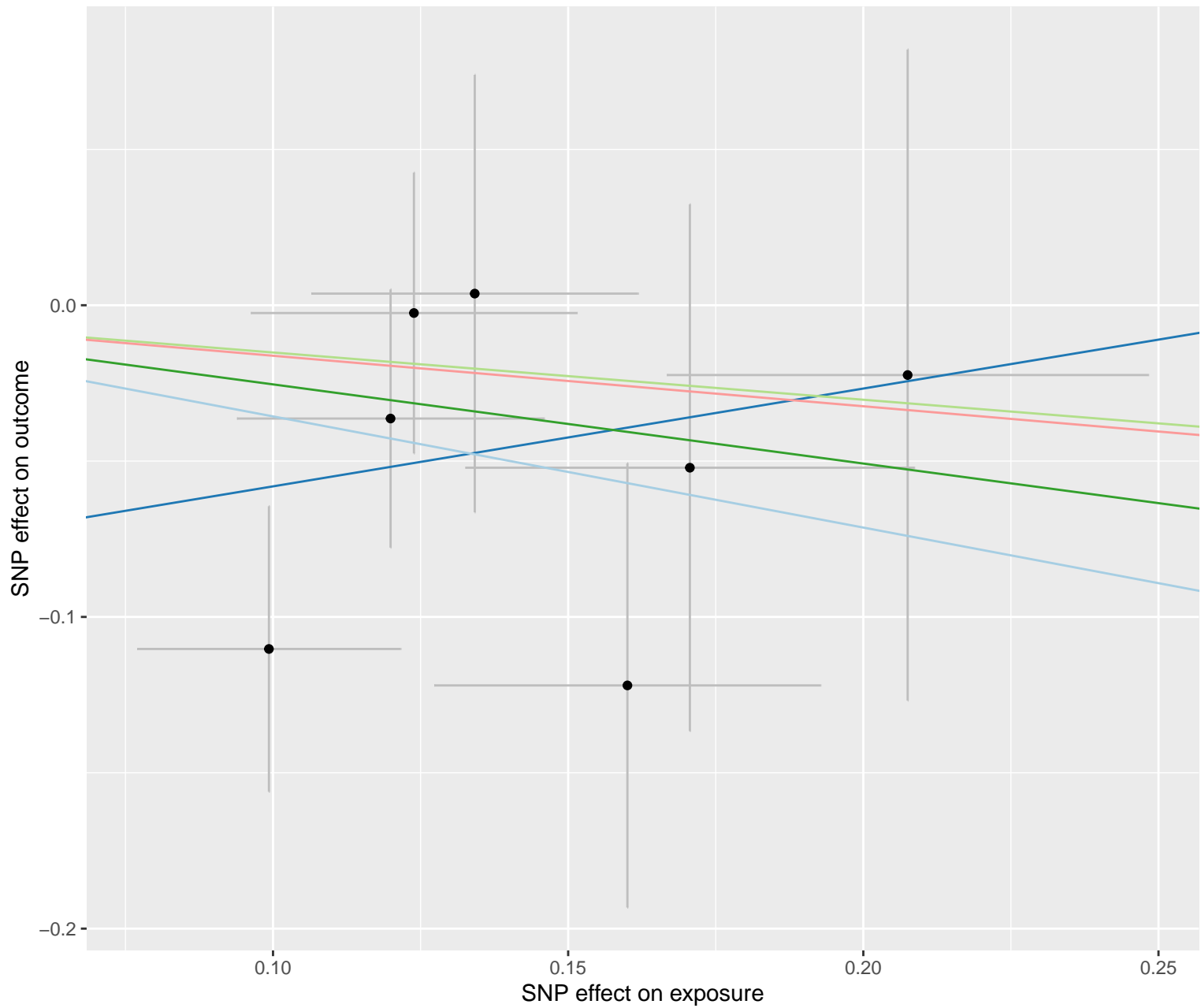

Supplement: Supporting Information — Supplement 1: The STROBE-MR checklist of recommended items to address in reports of Mendelian randomization studies. Supplement 2: The significant pleiotropy or heterogeneity of IVs in the MR analysis using gut microbiota as the exposure and osteonecrosis as the outcome. Supplement 3: The scatterplots and leave-one-out plots in the MR analysis using gut microbiota as the exposure and osteonecrosis as the outcome. Supplement 4: The significant pleiotropy or heterogeneity of IVs in the MR analysis using immune cells as the exposure and osteonecrosis as the outcome. Supplement 5: The scatterplots and leave-one-out plots in the MR analysis using immune cells as the exposure and osteonecrosis as the outcome. Supplement 6: The significant pleiotropy or heterogeneity of IVs in the MR analysis using gut microbiota as the exposure and immune cells as the outcome. Supplement 7: The scatterplots and leave-one-out plots in the MR analysis using gut microbiota as the exposure and immune cells as the outcome. Supplement 8: The results of the MR analysis using osteonecrosis as the exposure and gut microbiota and immune cells as the outcomes. [file 9323113.f1.zip › Supplement 3/GCST90027627/scatter.pdf]

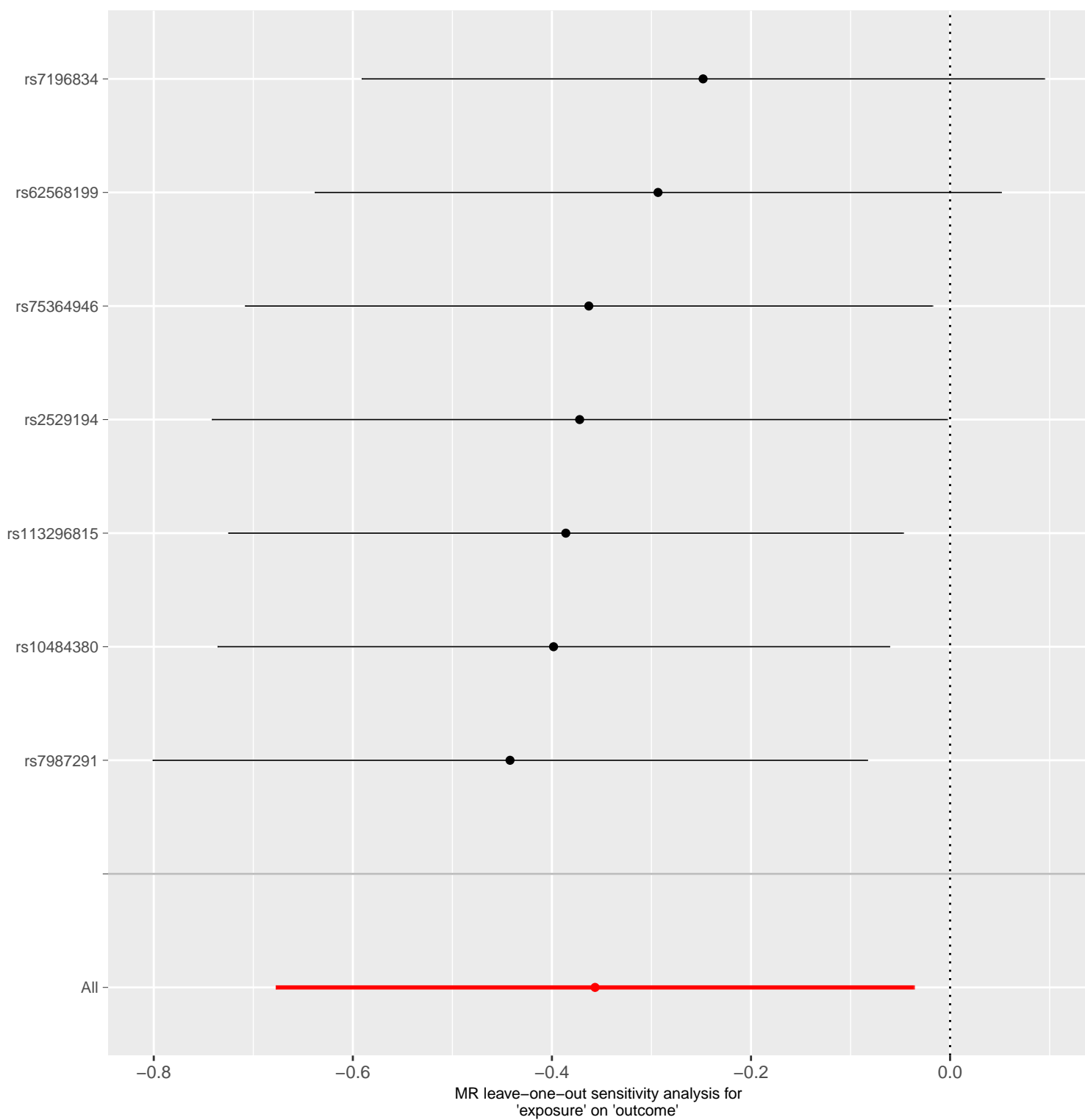

Supplement: Supporting Information — Supplement 1: The STROBE-MR checklist of recommended items to address in reports of Mendelian randomization studies. Supplement 2: The significant pleiotropy or heterogeneity of IVs in the MR analysis using gut microbiota as the exposure and osteonecrosis as the outcome. Supplement 3: The scatterplots and leave-one-out plots in the MR analysis using gut microbiota as the exposure and osteonecrosis as the outcome. Supplement 4: The significant pleiotropy or heterogeneity of IVs in the MR analysis using immune cells as the exposure and osteonecrosis as the outcome. Supplement 5: The scatterplots and leave-one-out plots in the MR analysis using immune cells as the exposure and osteonecrosis as the outcome. Supplement 6: The significant pleiotropy or heterogeneity of IVs in the MR analysis using gut microbiota as the exposure and immune cells as the outcome. Supplement 7: The scatterplots and leave-one-out plots in the MR analysis using gut microbiota as the exposure and immune cells as the outcome. Supplement 8: The results of the MR analysis using osteonecrosis as the exposure and gut microbiota and immune cells as the outcomes. [file 9323113.f1.zip › Supplement 3/GCST90027627/sensitivity-analysis.pdf]

# MR Test

- Inverse variance weighted
- MR Egger
- Simple mode
- Weighted median
- Weighted mode

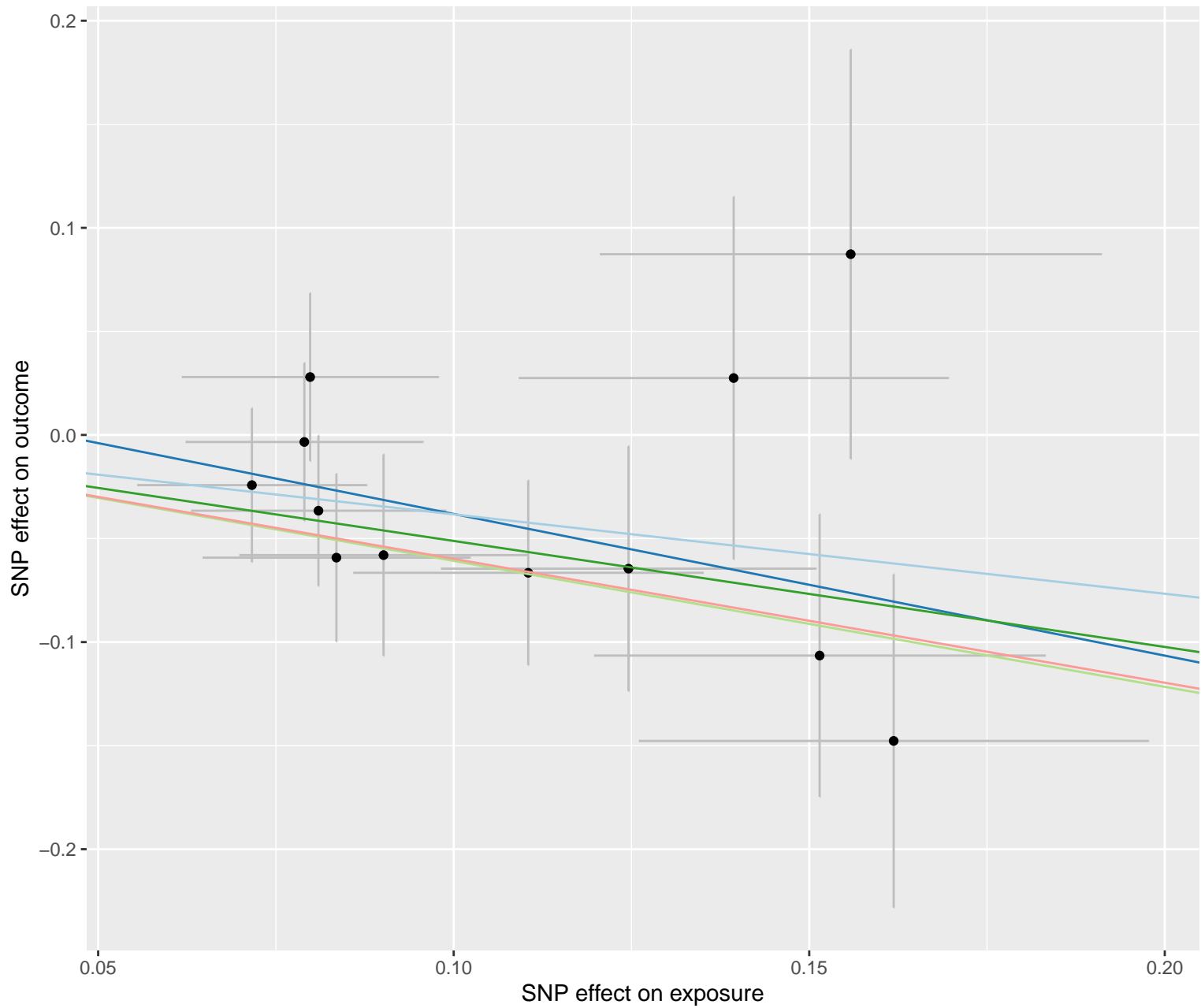

Supplement: Supporting Information — Supplement 1: The STROBE-MR checklist of recommended items to address in reports of Mendelian randomization studies. Supplement 2: The significant pleiotropy or heterogeneity of IVs in the MR analysis using gut microbiota as the exposure and osteonecrosis as the outcome. Supplement 3: The scatterplots and leave-one-out plots in the MR analysis using gut microbiota as the exposure and osteonecrosis as the outcome. Supplement 4: The significant pleiotropy or heterogeneity of IVs in the MR analysis using immune cells as the exposure and osteonecrosis as the outcome. Supplement 5: The scatterplots and leave-one-out plots in the MR analysis using immune cells as the exposure and osteonecrosis as the outcome. Supplement 6: The significant pleiotropy or heterogeneity of IVs in the MR analysis using gut microbiota as the exposure and immune cells as the outcome. Supplement 7: The scatterplots and leave-one-out plots in the MR analysis using gut microbiota as the exposure and immune cells as the outcome. Supplement 8: The results of the MR analysis using osteonecrosis as the exposure and gut microbiota and immune cells as the outcomes. [file 9323113.f1.zip › Supplement 3/GCST90027644/scatter.pdf]

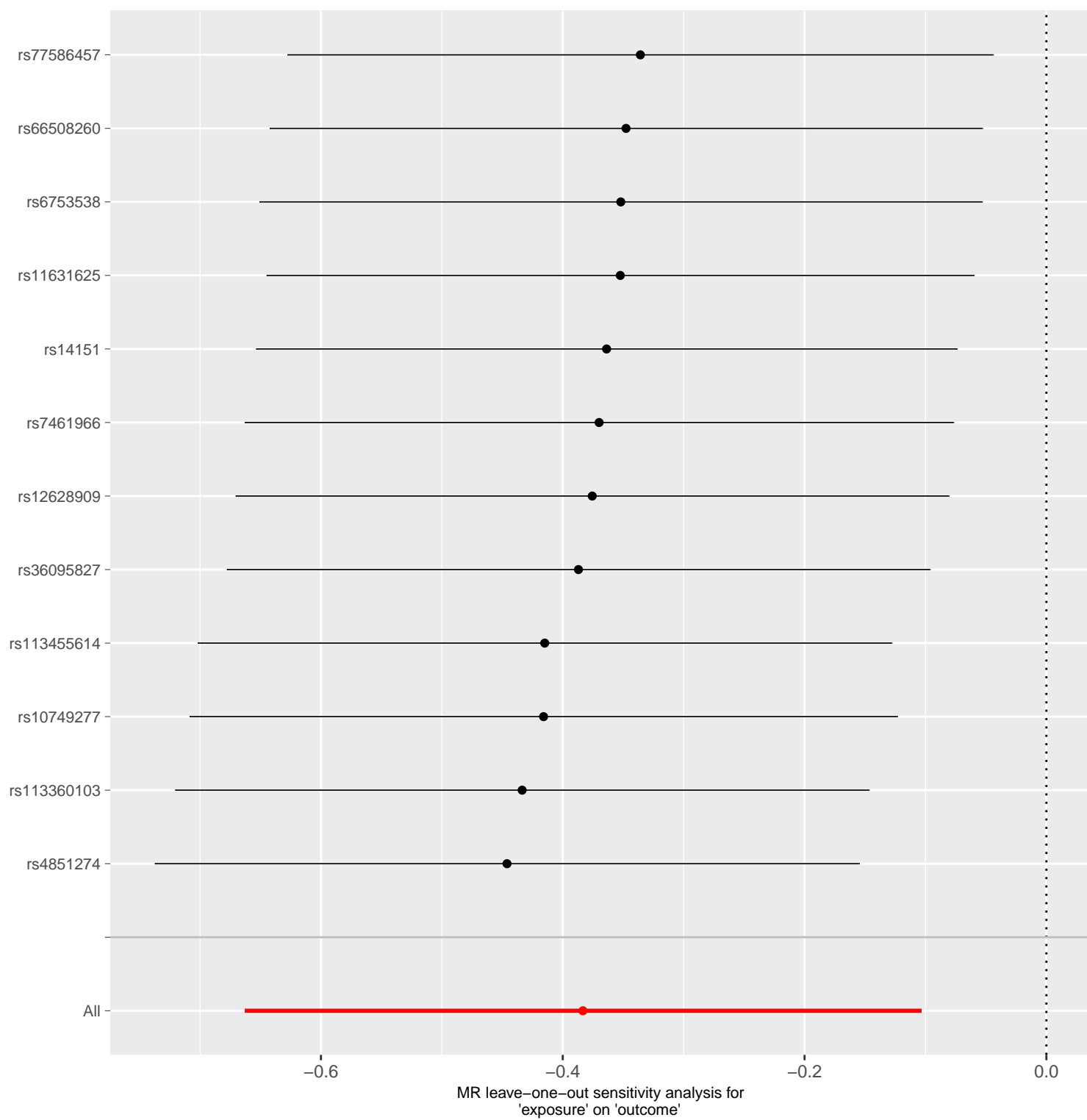

Supplement: Supporting Information — Supplement 1: The STROBE-MR checklist of recommended items to address in reports of Mendelian randomization studies. Supplement 2: The significant pleiotropy or heterogeneity of IVs in the MR analysis using gut microbiota as the exposure and osteonecrosis as the outcome. Supplement 3: The scatterplots and leave-one-out plots in the MR analysis using gut microbiota as the exposure and osteonecrosis as the outcome. Supplement 4: The significant pleiotropy or heterogeneity of IVs in the MR analysis using immune cells as the exposure and osteonecrosis as the outcome. Supplement 5: The scatterplots and leave-one-out plots in the MR analysis using immune cells as the exposure and osteonecrosis as the outcome. Supplement 6: The significant pleiotropy or heterogeneity of IVs in the MR analysis using gut microbiota as the exposure and immune cells as the outcome. Supplement 7: The scatterplots and leave-one-out plots in the MR analysis using gut microbiota as the exposure and immune cells as the outcome. Supplement 8: The results of the MR analysis using osteonecrosis as the exposure and gut microbiota and immune cells as the outcomes. [file 9323113.f1.zip › Supplement 3/GCST90027644/sensitivity-analysis.pdf]

# MR Test

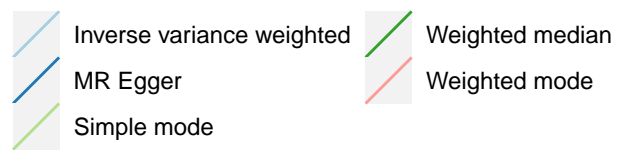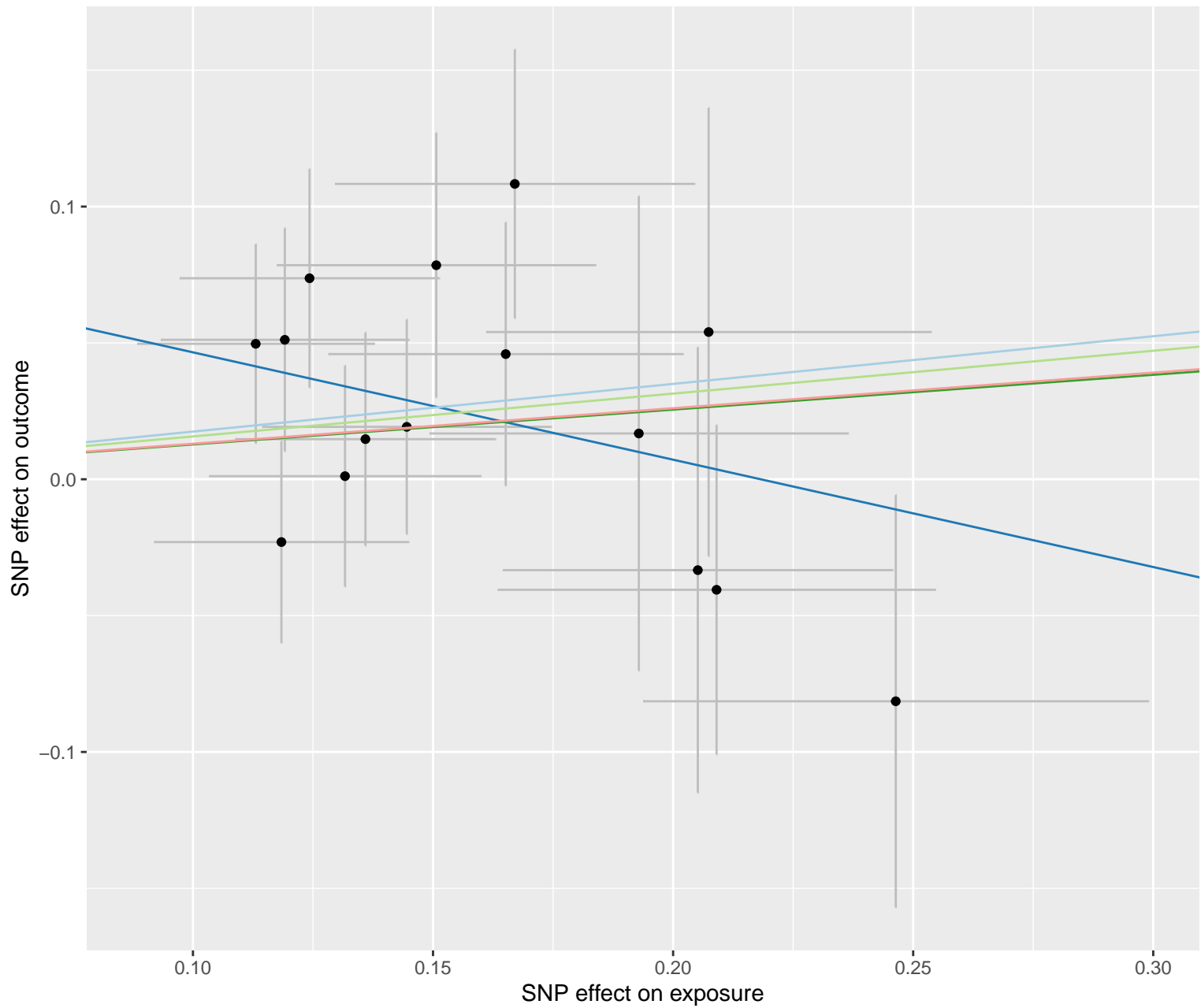

Supplement: Supporting Information — Supplement 1: The STROBE-MR checklist of recommended items to address in reports of Mendelian randomization studies. Supplement 2: The significant pleiotropy or heterogeneity of IVs in the MR analysis using gut microbiota as the exposure and osteonecrosis as the outcome. Supplement 3: The scatterplots and leave-one-out plots in the MR analysis using gut microbiota as the exposure and osteonecrosis as the outcome. Supplement 4: The significant pleiotropy or heterogeneity of IVs in the MR analysis using immune cells as the exposure and osteonecrosis as the outcome. Supplement 5: The scatterplots and leave-one-out plots in the MR analysis using immune cells as the exposure and osteonecrosis as the outcome. Supplement 6: The significant pleiotropy or heterogeneity of IVs in the MR analysis using gut microbiota as the exposure and immune cells as the outcome. Supplement 7: The scatterplots and leave-one-out plots in the MR analysis using gut microbiota as the exposure and immune cells as the outcome. Supplement 8: The results of the MR analysis using osteonecrosis as the exposure and gut microbiota and immune cells as the outcomes. [file 9323113.f1.zip › Supplement 3/GCST90027670/scatter.pdf]

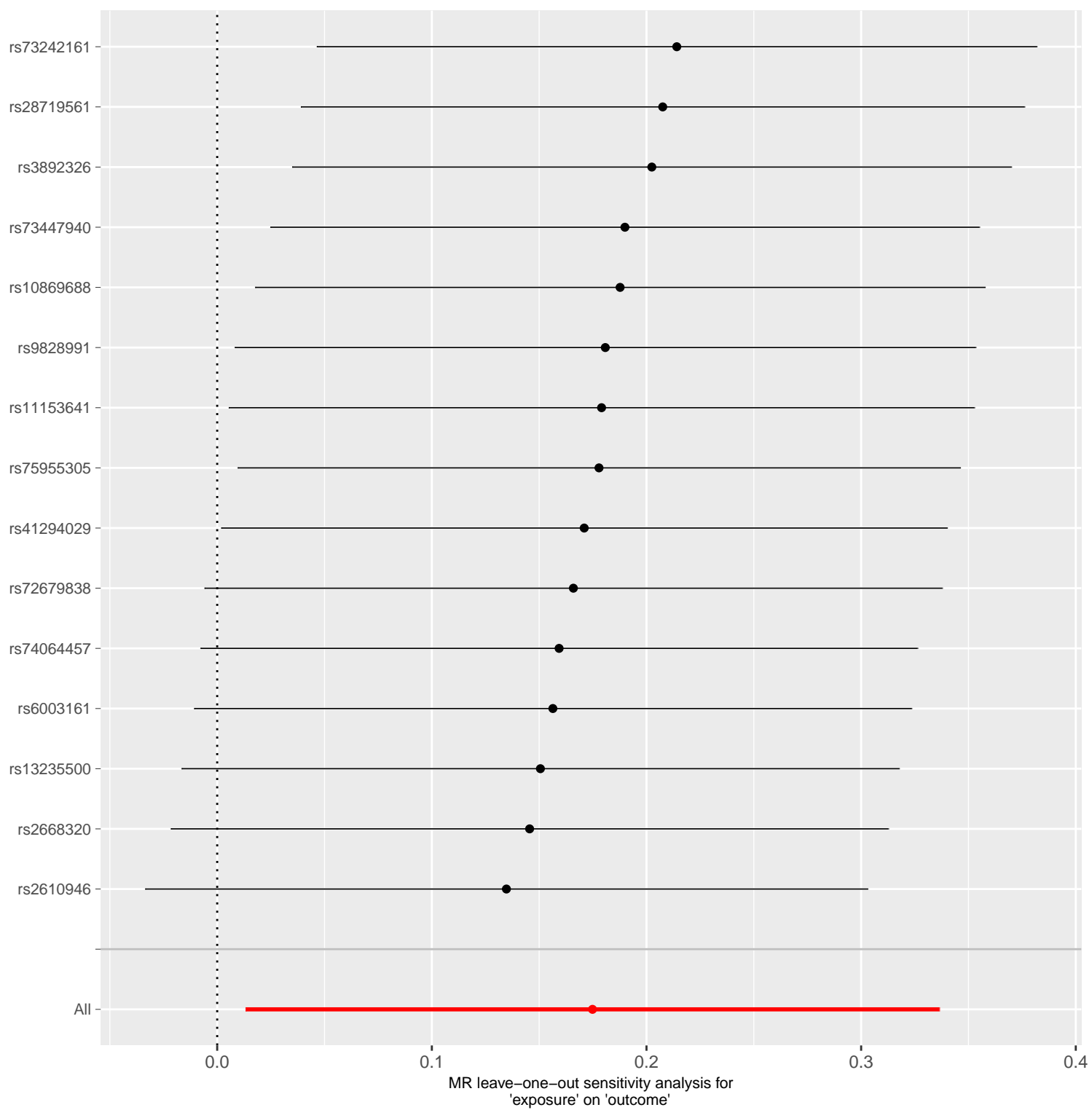

Supplement: Supporting Information — Supplement 1: The STROBE-MR checklist of recommended items to address in reports of Mendelian randomization studies. Supplement 2: The significant pleiotropy or heterogeneity of IVs in the MR analysis using gut microbiota as the exposure and osteonecrosis as the outcome. Supplement 3: The scatterplots and leave-one-out plots in the MR analysis using gut microbiota as the exposure and osteonecrosis as the outcome. Supplement 4: The significant pleiotropy or heterogeneity of IVs in the MR analysis using immune cells as the exposure and osteonecrosis as the outcome. Supplement 5: The scatterplots and leave-one-out plots in the MR analysis using immune cells as the exposure and osteonecrosis as the outcome. Supplement 6: The significant pleiotropy or heterogeneity of IVs in the MR analysis using gut microbiota as the exposure and immune cells as the outcome. Supplement 7: The scatterplots and leave-one-out plots in the MR analysis using gut microbiota as the exposure and immune cells as the outcome. Supplement 8: The results of the MR analysis using osteonecrosis as the exposure and gut microbiota and immune cells as the outcomes. [file 9323113.f1.zip › Supplement 3/GCST90027670/sensitivity-analysis.pdf]

# MR Test

- Inverse variance weighted
- MR Egger
- Simple mode
- Weighted median
- Weighted mode

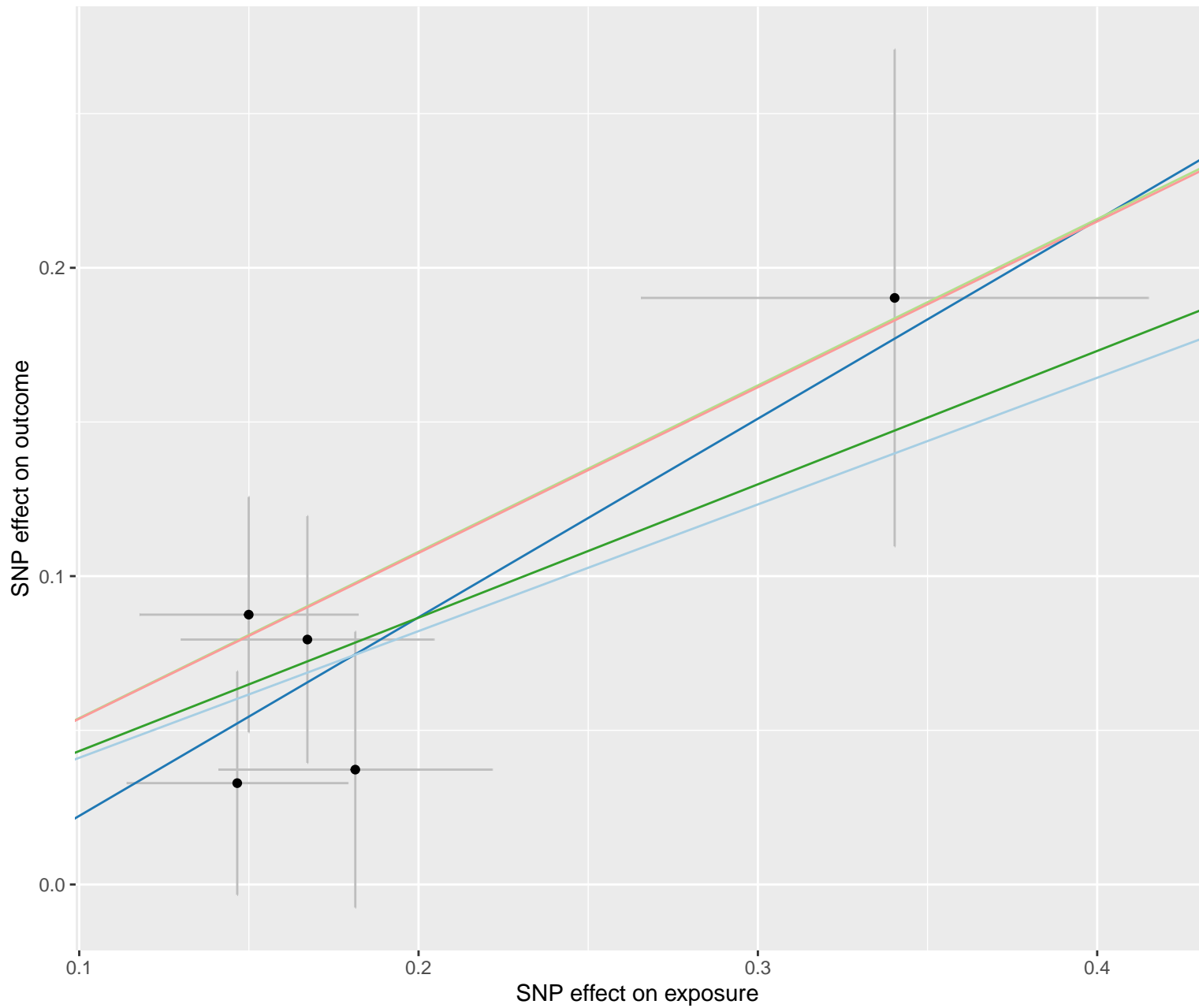

Supplement: Supporting Information — Supplement 1: The STROBE-MR checklist of recommended items to address in reports of Mendelian randomization studies. Supplement 2: The significant pleiotropy or heterogeneity of IVs in the MR analysis using gut microbiota as the exposure and osteonecrosis as the outcome. Supplement 3: The scatterplots and leave-one-out plots in the MR analysis using gut microbiota as the exposure and osteonecrosis as the outcome. Supplement 4: The significant pleiotropy or heterogeneity of IVs in the MR analysis using immune cells as the exposure and osteonecrosis as the outcome. Supplement 5: The scatterplots and leave-one-out plots in the MR analysis using immune cells as the exposure and osteonecrosis as the outcome. Supplement 6: The significant pleiotropy or heterogeneity of IVs in the MR analysis using gut microbiota as the exposure and immune cells as the outcome. Supplement 7: The scatterplots and leave-one-out plots in the MR analysis using gut microbiota as the exposure and immune cells as the outcome. Supplement 8: The results of the MR analysis using osteonecrosis as the exposure and gut microbiota and immune cells as the outcomes. [file 9323113.f1.zip › Supplement 3/GCST90027691/scatter.pdf]

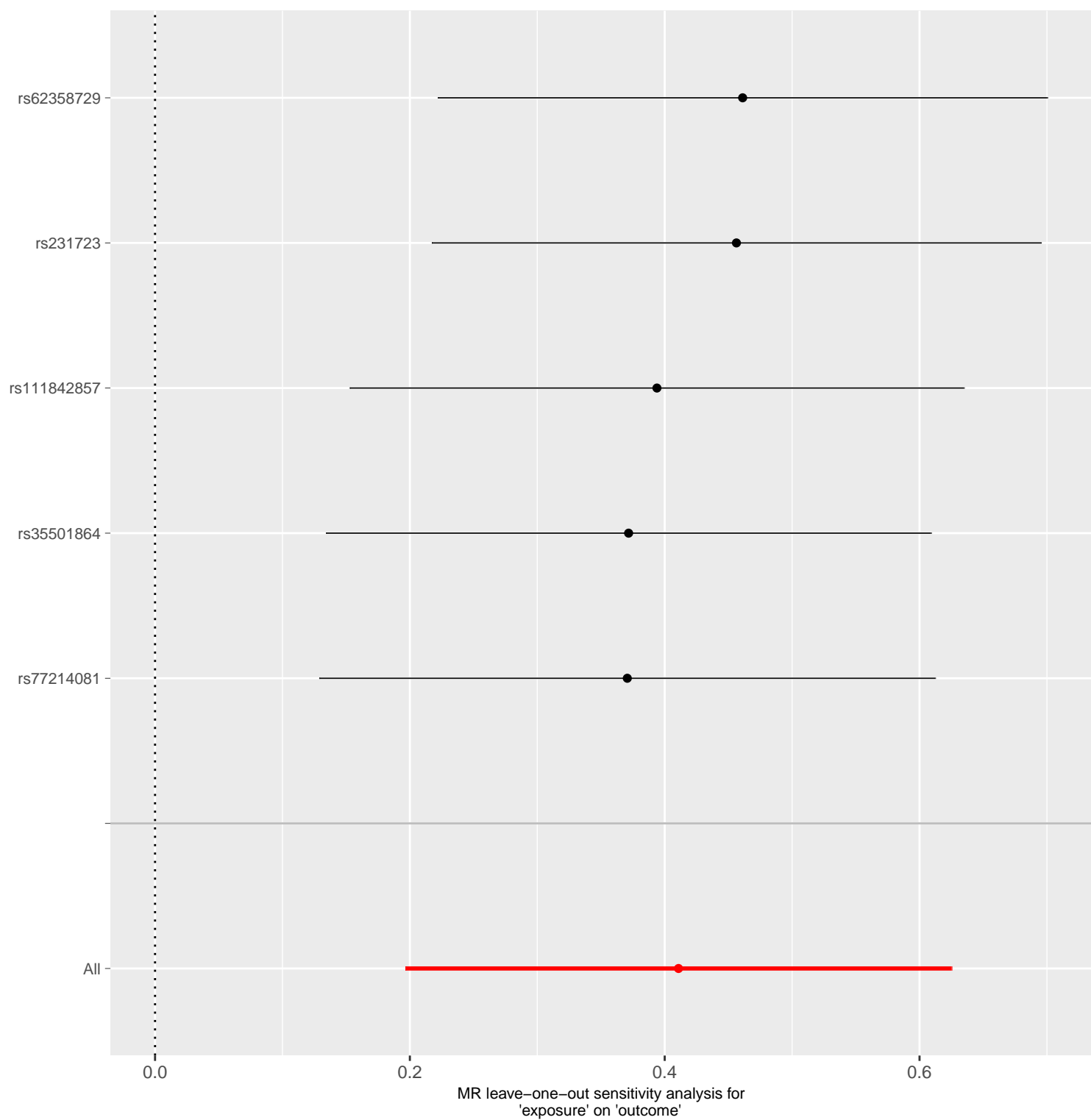

Supplement: Supporting Information — Supplement 1: The STROBE-MR checklist of recommended items to address in reports of Mendelian randomization studies. Supplement 2: The significant pleiotropy or heterogeneity of IVs in the MR analysis using gut microbiota as the exposure and osteonecrosis as the outcome. Supplement 3: The scatterplots and leave-one-out plots in the MR analysis using gut microbiota as the exposure and osteonecrosis as the outcome. Supplement 4: The significant pleiotropy or heterogeneity of IVs in the MR analysis using immune cells as the exposure and osteonecrosis as the outcome. Supplement 5: The scatterplots and leave-one-out plots in the MR analysis using immune cells as the exposure and osteonecrosis as the outcome. Supplement 6: The significant pleiotropy or heterogeneity of IVs in the MR analysis using gut microbiota as the exposure and immune cells as the outcome. Supplement 7: The scatterplots and leave-one-out plots in the MR analysis using gut microbiota as the exposure and immune cells as the outcome. Supplement 8: The results of the MR analysis using osteonecrosis as the exposure and gut microbiota and immune cells as the outcomes. [file 9323113.f1.zip › Supplement 3/GCST90027691/sensitivity-analysis.pdf]

# MR Test

- Inverse variance weighted
- MR Egger
- Simple mode
- Weighted median
- Weighted mode

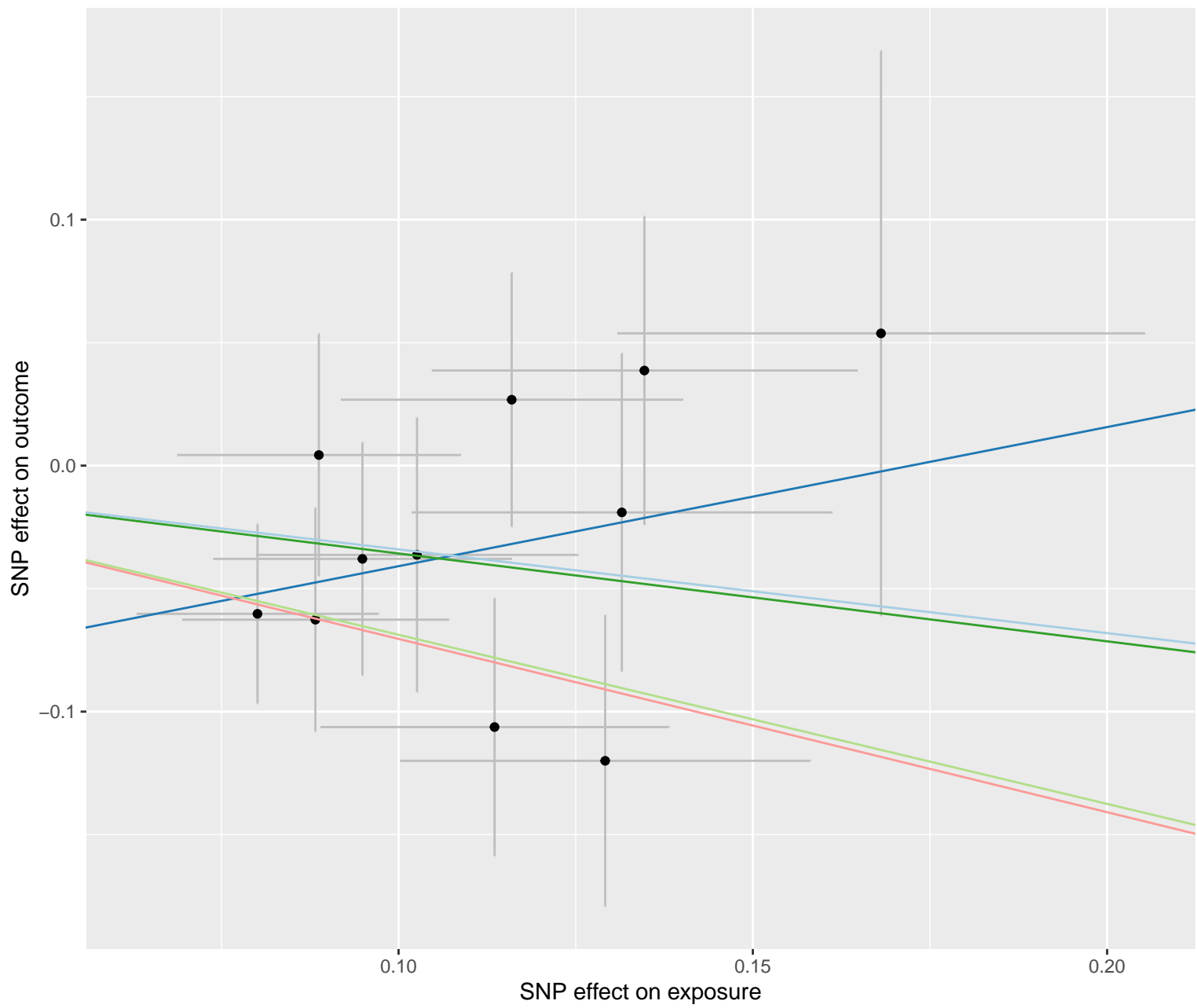

Supplement: Supporting Information — Supplement 1: The STROBE-MR checklist of recommended items to address in reports of Mendelian randomization studies. Supplement 2: The significant pleiotropy or heterogeneity of IVs in the MR analysis using gut microbiota as the exposure and osteonecrosis as the outcome. Supplement 3: The scatterplots and leave-one-out plots in the MR analysis using gut microbiota as the exposure and osteonecrosis as the outcome. Supplement 4: The significant pleiotropy or heterogeneity of IVs in the MR analysis using immune cells as the exposure and osteonecrosis as the outcome. Supplement 5: The scatterplots and leave-one-out plots in the MR analysis using immune cells as the exposure and osteonecrosis as the outcome. Supplement 6: The significant pleiotropy or heterogeneity of IVs in the MR analysis using gut microbiota as the exposure and immune cells as the outcome. Supplement 7: The scatterplots and leave-one-out plots in the MR analysis using gut microbiota as the exposure and immune cells as the outcome. Supplement 8: The results of the MR analysis using osteonecrosis as the exposure and gut microbiota and immune cells as the outcomes. [file 9323113.f1.zip › Supplement 3/GCST90027780/scatter.pdf]

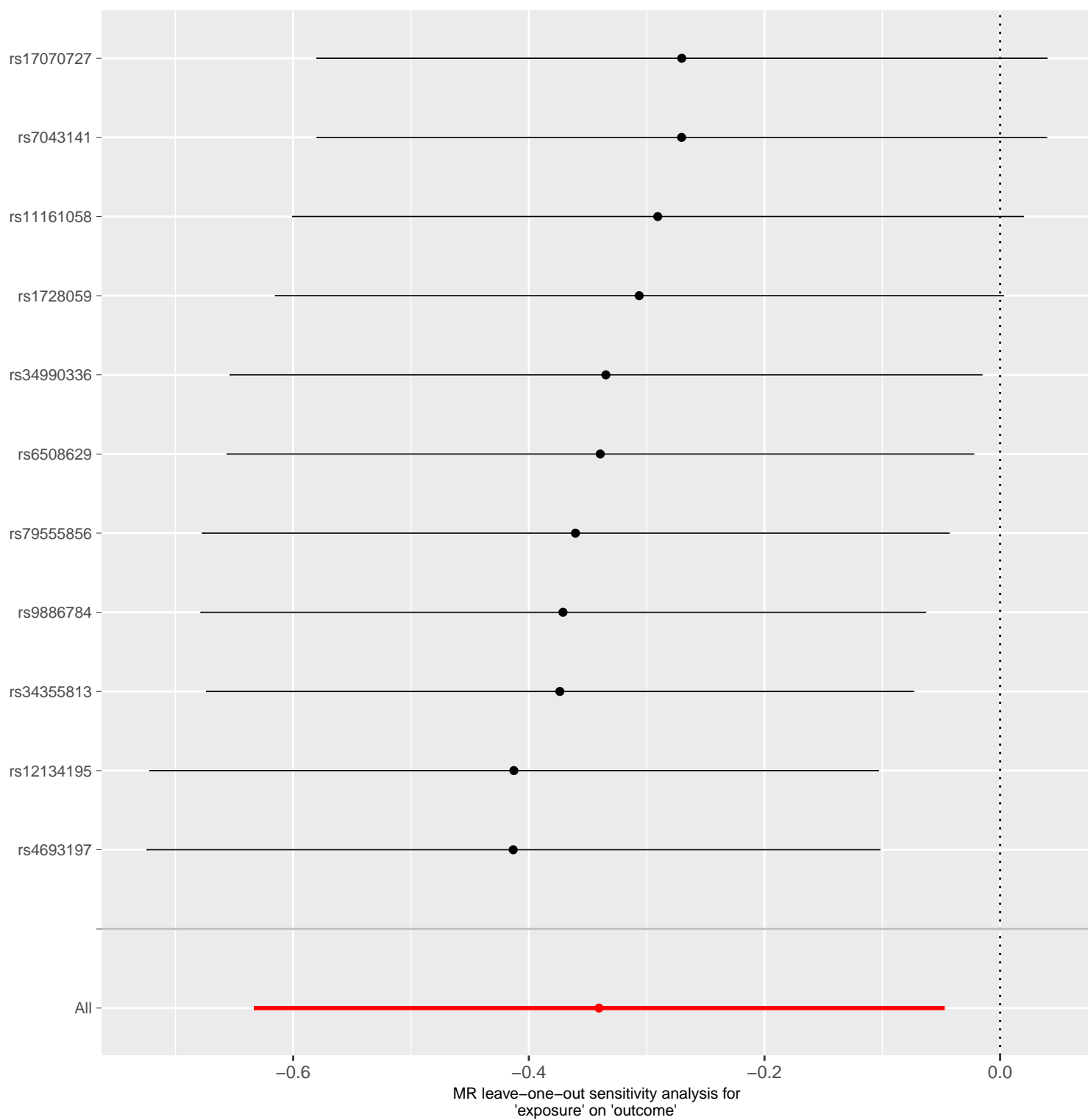

Supplement: Supporting Information — Supplement 1: The STROBE-MR checklist of recommended items to address in reports of Mendelian randomization studies. Supplement 2: The significant pleiotropy or heterogeneity of IVs in the MR analysis using gut microbiota as the exposure and osteonecrosis as the outcome. Supplement 3: The scatterplots and leave-one-out plots in the MR analysis using gut microbiota as the exposure and osteonecrosis as the outcome. Supplement 4: The significant pleiotropy or heterogeneity of IVs in the MR analysis using immune cells as the exposure and osteonecrosis as the outcome. Supplement 5: The scatterplots and leave-one-out plots in the MR analysis using immune cells as the exposure and osteonecrosis as the outcome. Supplement 6: The significant pleiotropy or heterogeneity of IVs in the MR analysis using gut microbiota as the exposure and immune cells as the outcome. Supplement 7: The scatterplots and leave-one-out plots in the MR analysis using gut microbiota as the exposure and immune cells as the outcome. Supplement 8: The results of the MR analysis using osteonecrosis as the exposure and gut microbiota and immune cells as the outcomes. [file 9323113.f1.zip › Supplement 3/GCST90027780/sensitivity-analysis.pdf]

# MR Test

- Inverse variance weighted
- MR Egger
- Simple mode
- Weighted median
- Weighted mode

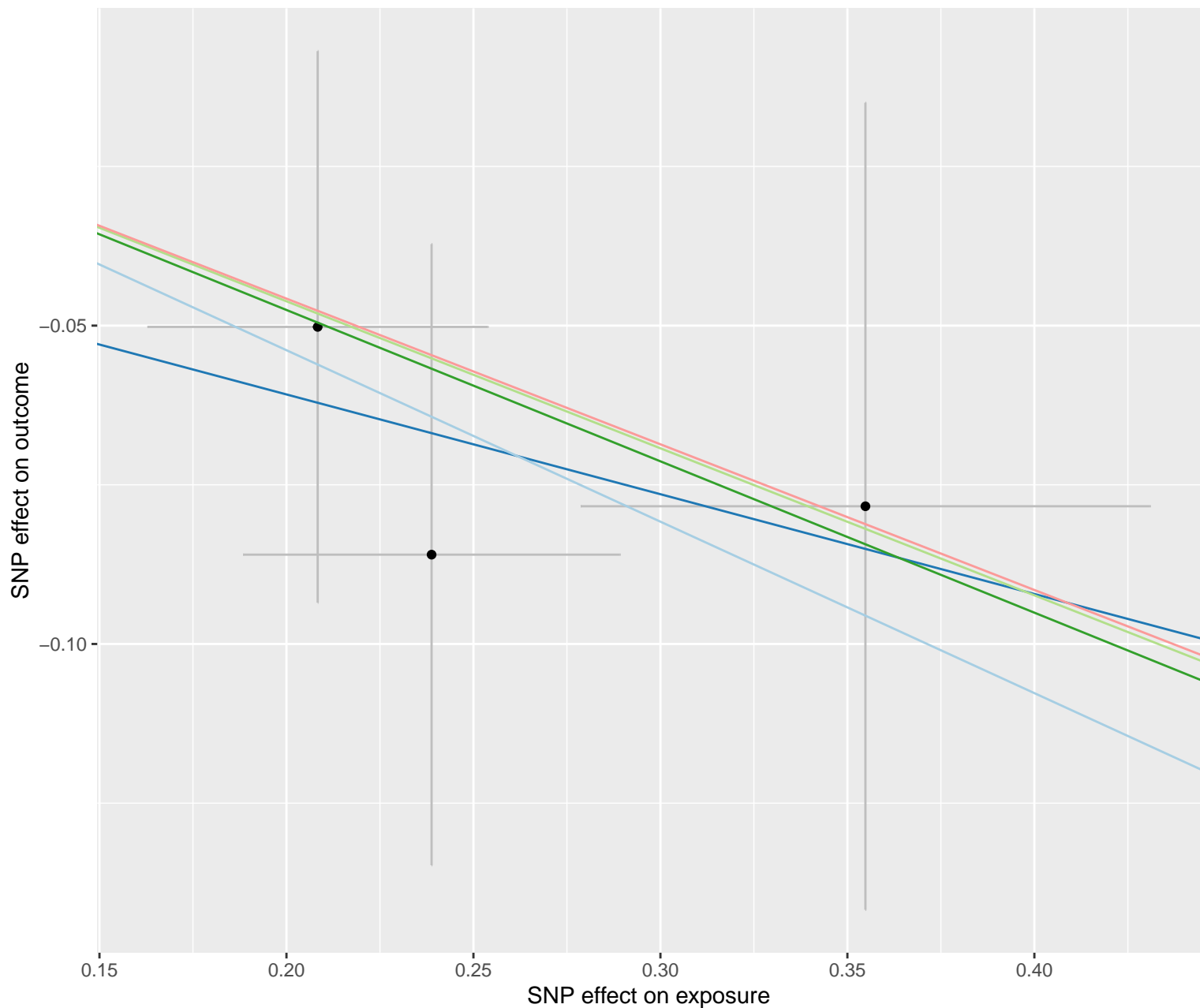

Supplement: Supporting Information — Supplement 1: The STROBE-MR checklist of recommended items to address in reports of Mendelian randomization studies. Supplement 2: The significant pleiotropy or heterogeneity of IVs in the MR analysis using gut microbiota as the exposure and osteonecrosis as the outcome. Supplement 3: The scatterplots and leave-one-out plots in the MR analysis using gut microbiota as the exposure and osteonecrosis as the outcome. Supplement 4: The significant pleiotropy or heterogeneity of IVs in the MR analysis using immune cells as the exposure and osteonecrosis as the outcome. Supplement 5: The scatterplots and leave-one-out plots in the MR analysis using immune cells as the exposure and osteonecrosis as the outcome. Supplement 6: The significant pleiotropy or heterogeneity of IVs in the MR analysis using gut microbiota as the exposure and immune cells as the outcome. Supplement 7: The scatterplots and leave-one-out plots in the MR analysis using gut microbiota as the exposure and immune cells as the outcome. Supplement 8: The results of the MR analysis using osteonecrosis as the exposure and gut microbiota and immune cells as the outcomes. [file 9323113.f1.zip › Supplement 3/GCST90027783/scatter.pdf]

rs6481791

rs1893286

rs117329402

All

-0.6

-0.4

-0.2

0.0

MR leave-one-out sensitivity analysis for  
'exposure' on 'outcome'

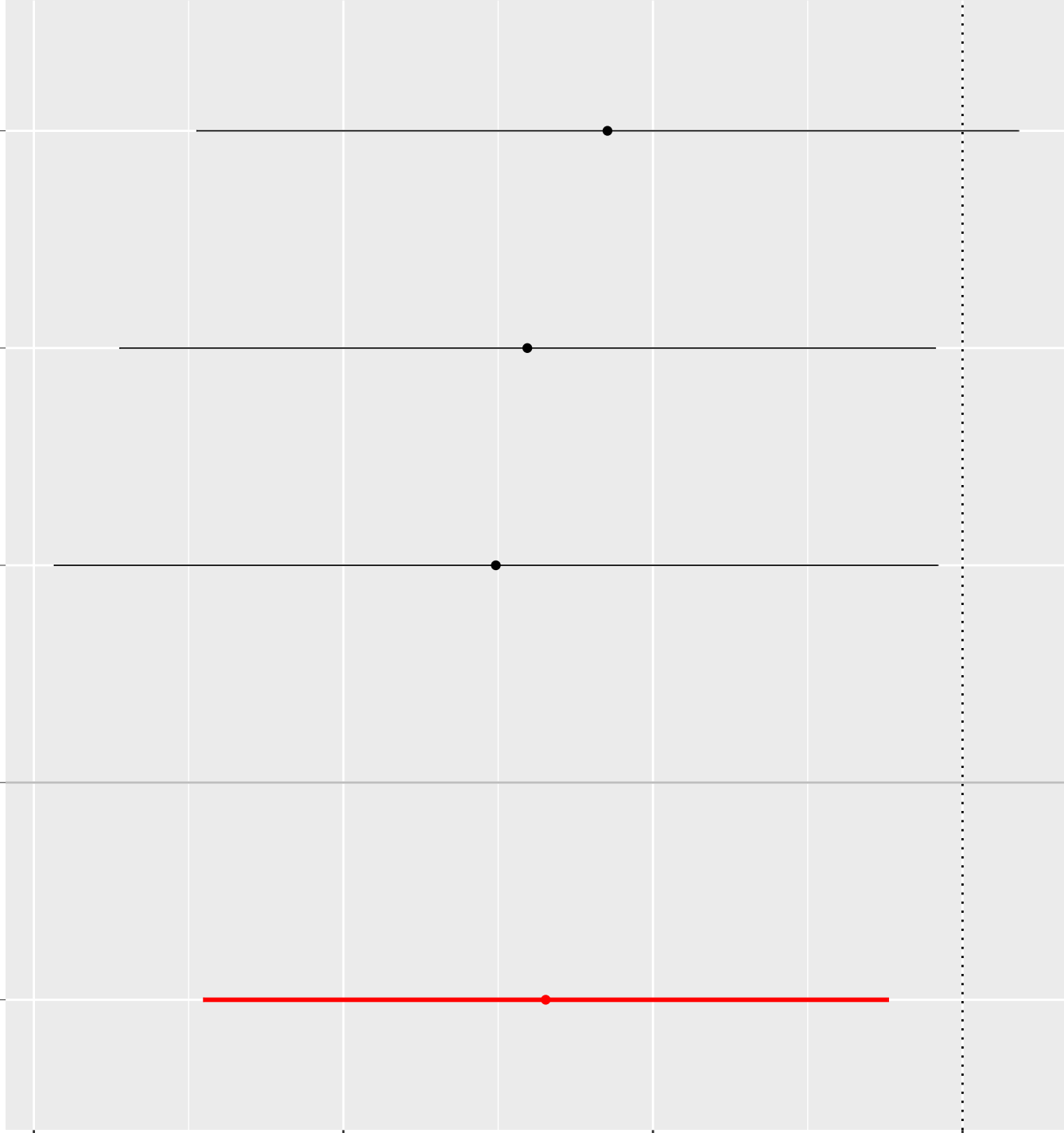

Supplement: Supporting Information — Supplement 1: The STROBE-MR checklist of recommended items to address in reports of Mendelian randomization studies. Supplement 2: The significant pleiotropy or heterogeneity of IVs in the MR analysis using gut microbiota as the exposure and osteonecrosis as the outcome. Supplement 3: The scatterplots and leave-one-out plots in the MR analysis using gut microbiota as the exposure and osteonecrosis as the outcome. Supplement 4: The significant pleiotropy or heterogeneity of IVs in the MR analysis using immune cells as the exposure and osteonecrosis as the outcome. Supplement 5: The scatterplots and leave-one-out plots in the MR analysis using immune cells as the exposure and osteonecrosis as the outcome. Supplement 6: The significant pleiotropy or heterogeneity of IVs in the MR analysis using gut microbiota as the exposure and immune cells as the outcome. Supplement 7: The scatterplots and leave-one-out plots in the MR analysis using gut microbiota as the exposure and immune cells as the outcome. Supplement 8: The results of the MR analysis using osteonecrosis as the exposure and gut microbiota and immune cells as the outcomes. [file 9323113.f1.zip › Supplement 3/GCST90027783/sensitivity-analysis.pdf]

# MR Test

- Inverse variance weighted
- MR Egger
- Simple mode
- Weighted median
- Weighted mode

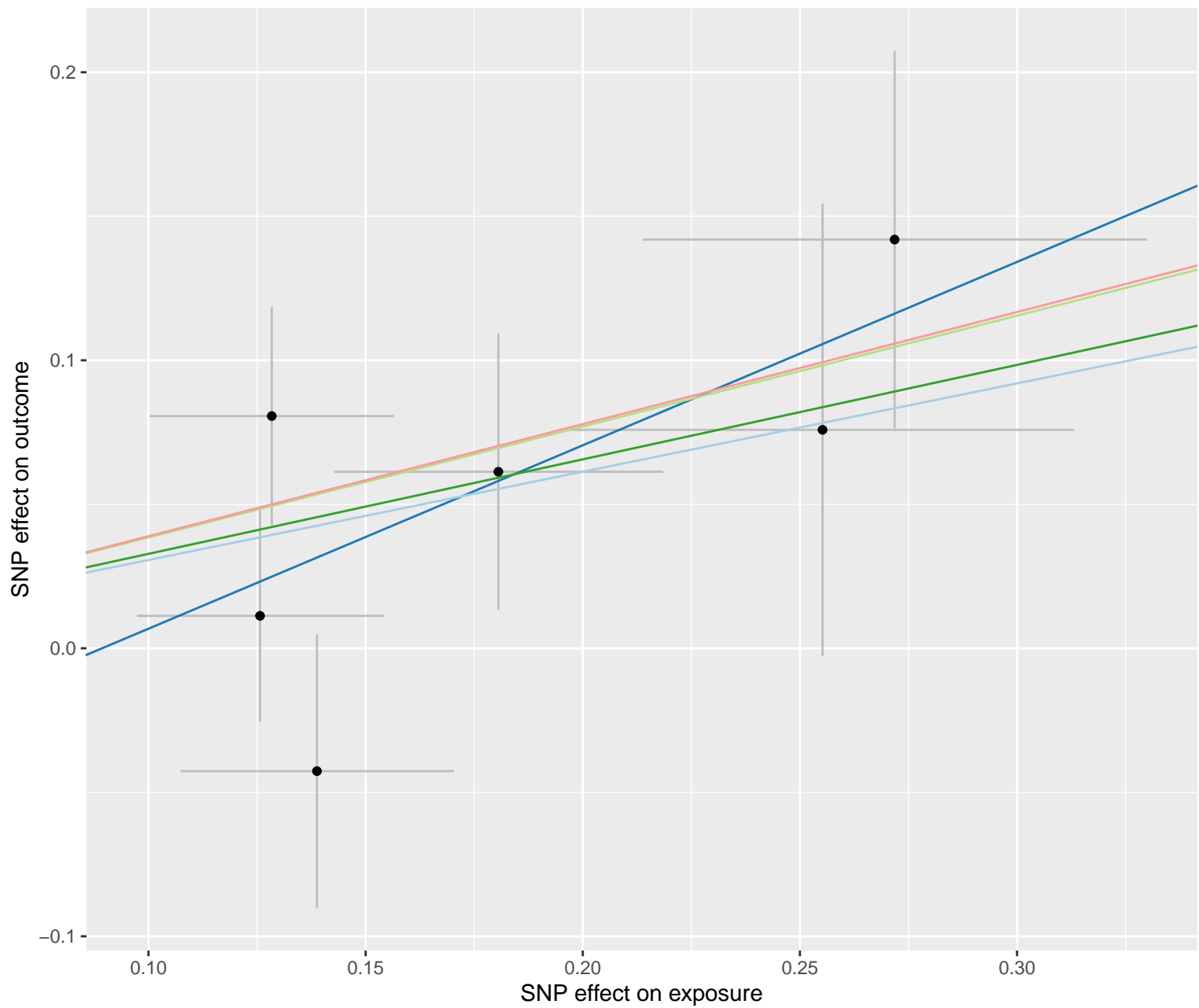

Supplement: Supporting Information — Supplement 1: The STROBE-MR checklist of recommended items to address in reports of Mendelian randomization studies. Supplement 2: The significant pleiotropy or heterogeneity of IVs in the MR analysis using gut microbiota as the exposure and osteonecrosis as the outcome. Supplement 3: The scatterplots and leave-one-out plots in the MR analysis using gut microbiota as the exposure and osteonecrosis as the outcome. Supplement 4: The significant pleiotropy or heterogeneity of IVs in the MR analysis using immune cells as the exposure and osteonecrosis as the outcome. Supplement 5: The scatterplots and leave-one-out plots in the MR analysis using immune cells as the exposure and osteonecrosis as the outcome. Supplement 6: The significant pleiotropy or heterogeneity of IVs in the MR analysis using gut microbiota as the exposure and immune cells as the outcome. Supplement 7: The scatterplots and leave-one-out plots in the MR analysis using gut microbiota as the exposure and immune cells as the outcome. Supplement 8: The results of the MR analysis using osteonecrosis as the exposure and gut microbiota and immune cells as the outcomes. [file 9323113.f1.zip › Supplement 3/GCST90027815/scatter.pdf]

rs75364852

rs196229

rs61818112

rs4854535

rs10030640

rs77902968

All

0.0

0.2

0.4

0.6

MR leave-one-out sensitivity analysis for  
'exposure' on 'outcome'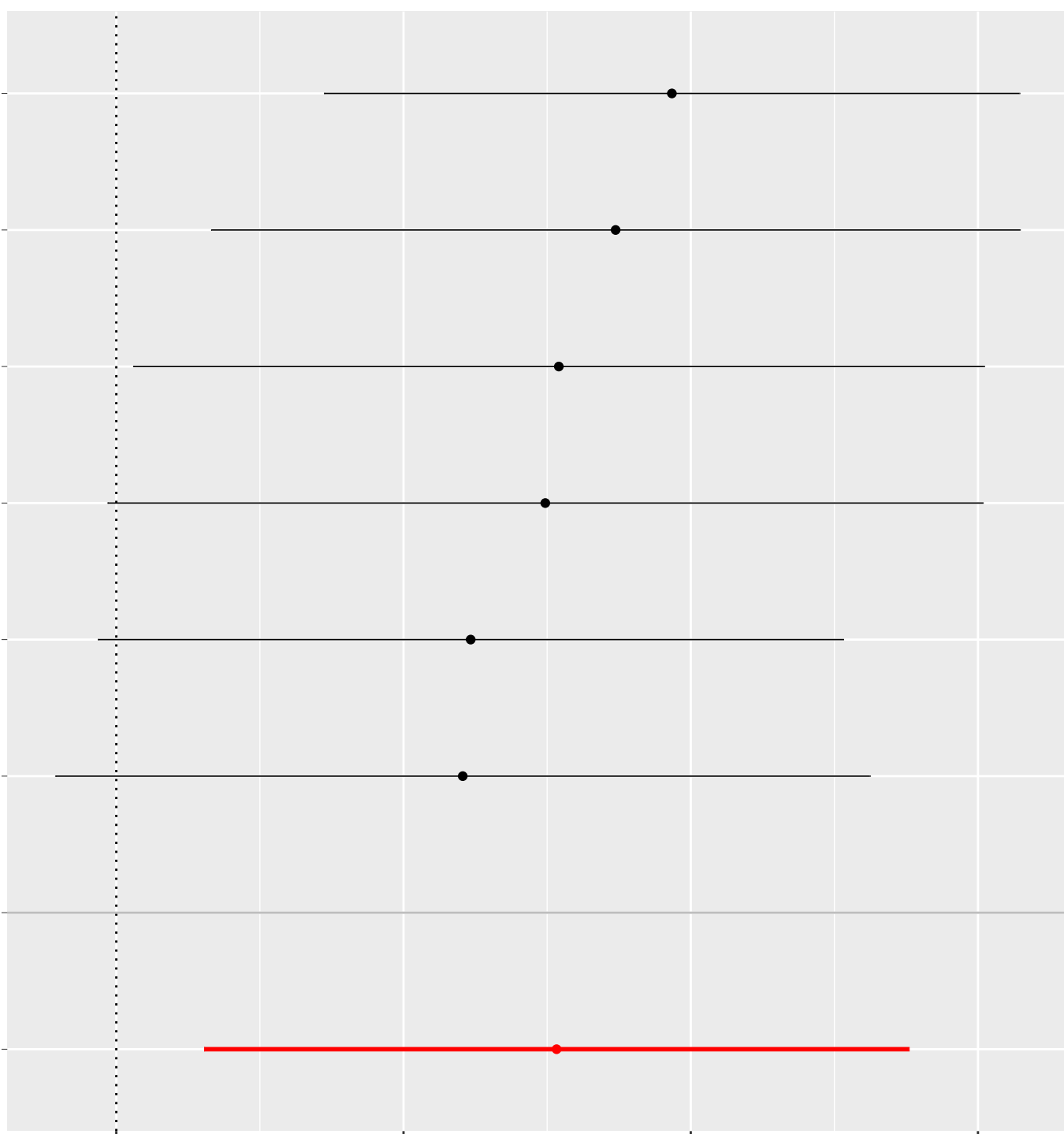

Supplement: Supporting Information — Supplement 1: The STROBE-MR checklist of recommended items to address in reports of Mendelian randomization studies. Supplement 2: The significant pleiotropy or heterogeneity of IVs in the MR analysis using gut microbiota as the exposure and osteonecrosis as the outcome. Supplement 3: The scatterplots and leave-one-out plots in the MR analysis using gut microbiota as the exposure and osteonecrosis as the outcome. Supplement 4: The significant pleiotropy or heterogeneity of IVs in the MR analysis using immune cells as the exposure and osteonecrosis as the outcome. Supplement 5: The scatterplots and leave-one-out plots in the MR analysis using immune cells as the exposure and osteonecrosis as the outcome. Supplement 6: The significant pleiotropy or heterogeneity of IVs in the MR analysis using gut microbiota as the exposure and immune cells as the outcome. Supplement 7: The scatterplots and leave-one-out plots in the MR analysis using gut microbiota as the exposure and immune cells as the outcome. Supplement 8: The results of the MR analysis using osteonecrosis as the exposure and gut microbiota and immune cells as the outcomes. [file 9323113.f1.zip › Supplement 3/GCST90027815/sensitivity-analysis.pdf]

# MR Test

- Inverse variance weighted
- MR Egger
- Simple mode
- Weighted median
- Weighted mode

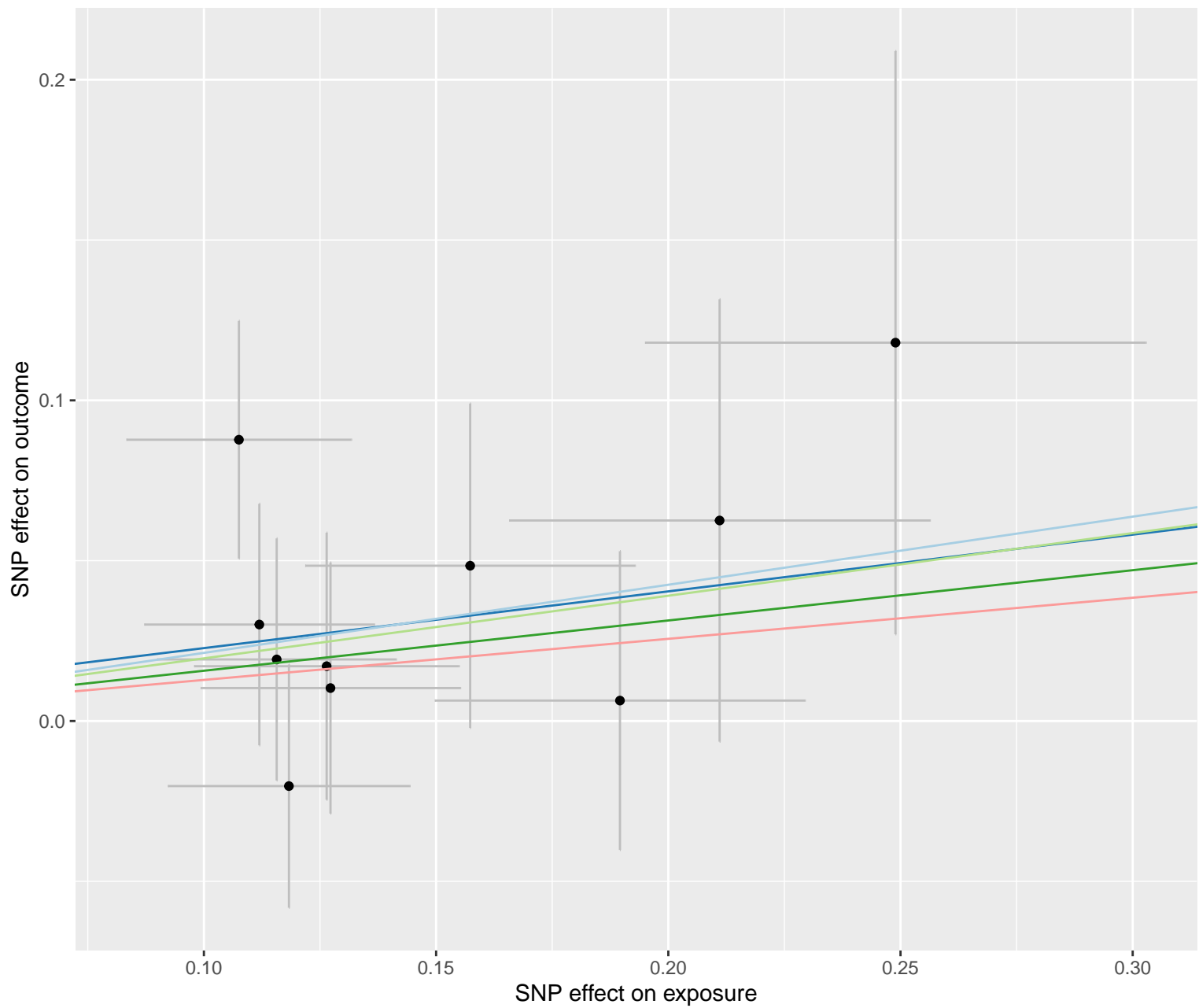

Supplement: Supporting Information — Supplement 1: The STROBE-MR checklist of recommended items to address in reports of Mendelian randomization studies. Supplement 2: The significant pleiotropy or heterogeneity of IVs in the MR analysis using gut microbiota as the exposure and osteonecrosis as the outcome. Supplement 3: The scatterplots and leave-one-out plots in the MR analysis using gut microbiota as the exposure and osteonecrosis as the outcome. Supplement 4: The significant pleiotropy or heterogeneity of IVs in the MR analysis using immune cells as the exposure and osteonecrosis as the outcome. Supplement 5: The scatterplots and leave-one-out plots in the MR analysis using immune cells as the exposure and osteonecrosis as the outcome. Supplement 6: The significant pleiotropy or heterogeneity of IVs in the MR analysis using gut microbiota as the exposure and immune cells as the outcome. Supplement 7: The scatterplots and leave-one-out plots in the MR analysis using gut microbiota as the exposure and immune cells as the outcome. Supplement 8: The results of the MR analysis using osteonecrosis as the exposure and gut microbiota and immune cells as the outcomes. [file 9323113.f1.zip › Supplement 3/GCST90027821/scatter.pdf]

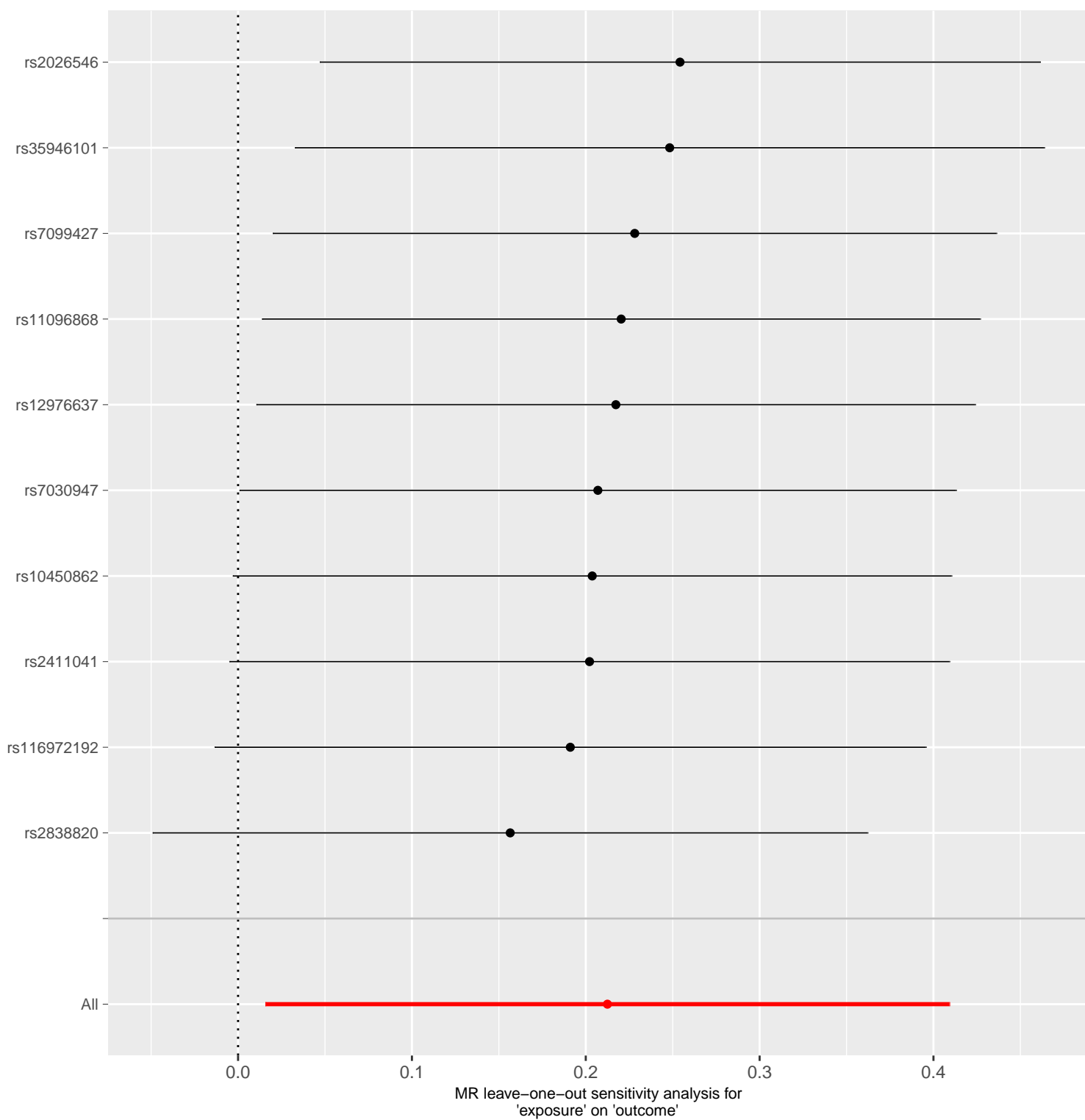

Supplement: Supporting Information — Supplement 1: The STROBE-MR checklist of recommended items to address in reports of Mendelian randomization studies. Supplement 2: The significant pleiotropy or heterogeneity of IVs in the MR analysis using gut microbiota as the exposure and osteonecrosis as the outcome. Supplement 3: The scatterplots and leave-one-out plots in the MR analysis using gut microbiota as the exposure and osteonecrosis as the outcome. Supplement 4: The significant pleiotropy or heterogeneity of IVs in the MR analysis using immune cells as the exposure and osteonecrosis as the outcome. Supplement 5: The scatterplots and leave-one-out plots in the MR analysis using immune cells as the exposure and osteonecrosis as the outcome. Supplement 6: The significant pleiotropy or heterogeneity of IVs in the MR analysis using gut microbiota as the exposure and immune cells as the outcome. Supplement 7: The scatterplots and leave-one-out plots in the MR analysis using gut microbiota as the exposure and immune cells as the outcome. Supplement 8: The results of the MR analysis using osteonecrosis as the exposure and gut microbiota and immune cells as the outcomes. [file 9323113.f1.zip › Supplement 3/GCST90027821/sensitivity-analysis.pdf]

# MR Test

- Inverse variance weighted
- MR Egger
- Simple mode
- Weighted median
- Weighted mode

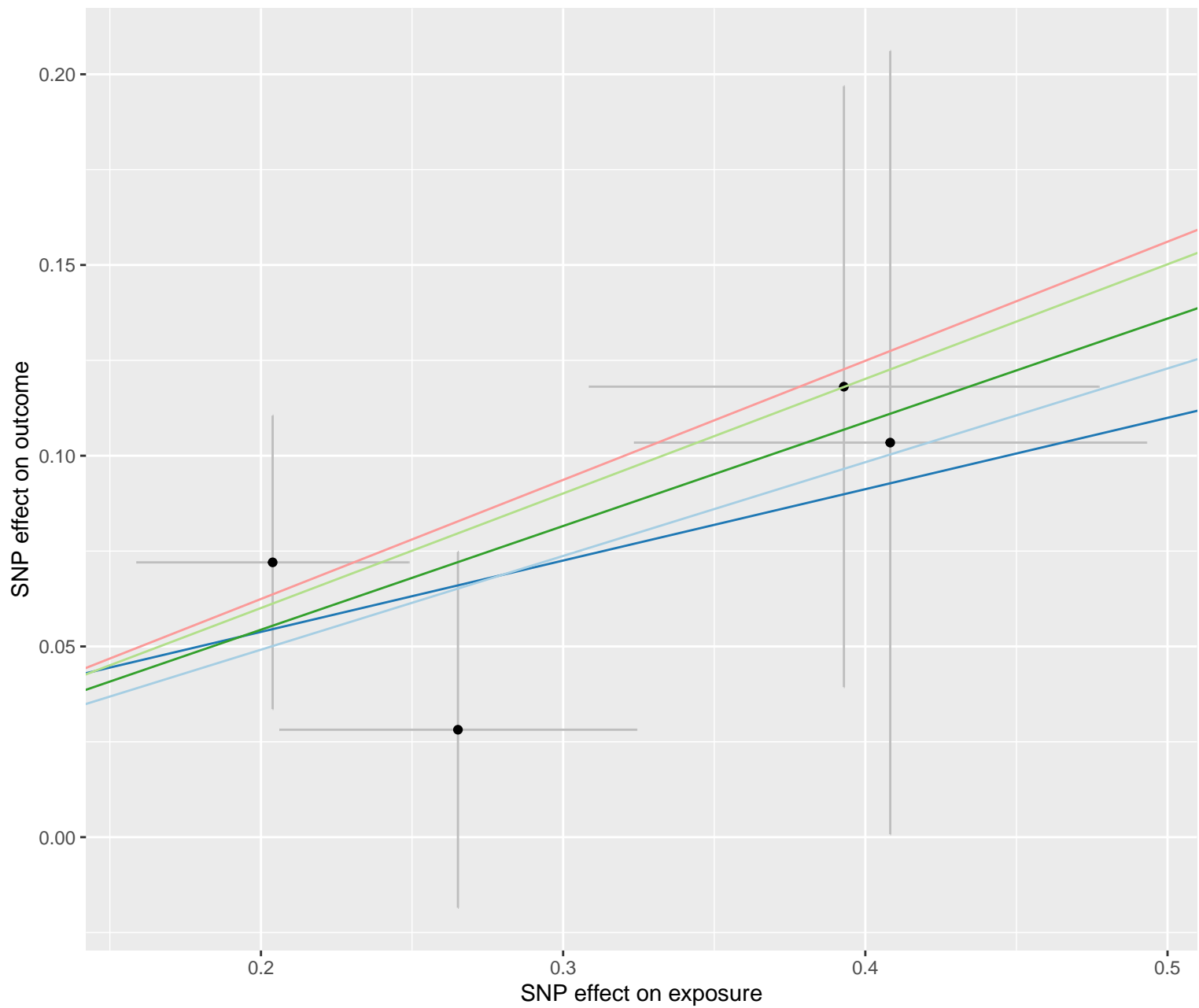

Supplement: Supporting Information — Supplement 1: The STROBE-MR checklist of recommended items to address in reports of Mendelian randomization studies. Supplement 2: The significant pleiotropy or heterogeneity of IVs in the MR analysis using gut microbiota as the exposure and osteonecrosis as the outcome. Supplement 3: The scatterplots and leave-one-out plots in the MR analysis using gut microbiota as the exposure and osteonecrosis as the outcome. Supplement 4: The significant pleiotropy or heterogeneity of IVs in the MR analysis using immune cells as the exposure and osteonecrosis as the outcome. Supplement 5: The scatterplots and leave-one-out plots in the MR analysis using immune cells as the exposure and osteonecrosis as the outcome. Supplement 6: The significant pleiotropy or heterogeneity of IVs in the MR analysis using gut microbiota as the exposure and immune cells as the outcome. Supplement 7: The scatterplots and leave-one-out plots in the MR analysis using gut microbiota as the exposure and immune cells as the outcome. Supplement 8: The results of the MR analysis using osteonecrosis as the exposure and gut microbiota and immune cells as the outcomes. [file 9323113.f1.zip › Supplement 3/GCST90027823/scatter.pdf]

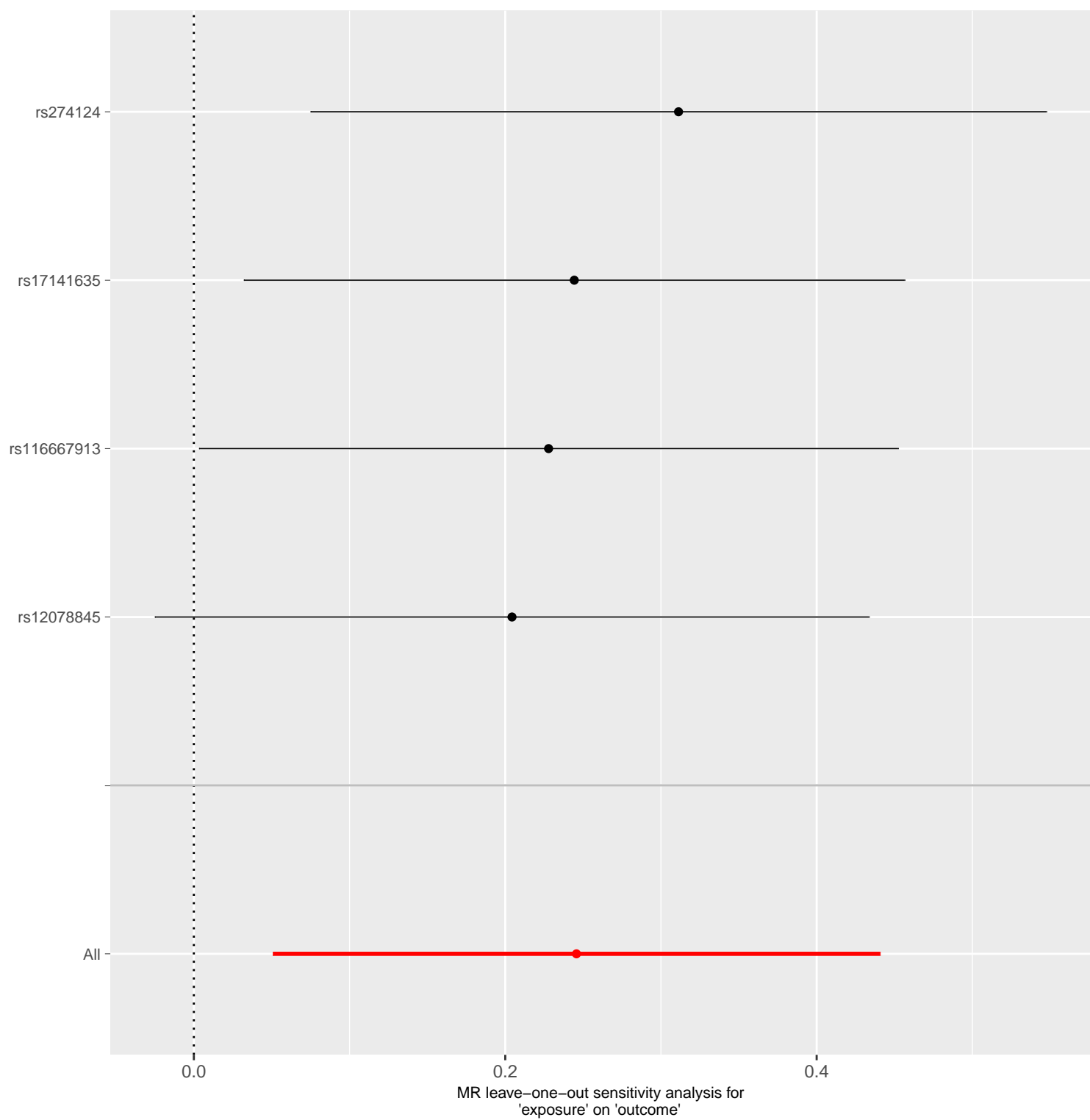

Supplement: Supporting Information — Supplement 1: The STROBE-MR checklist of recommended items to address in reports of Mendelian randomization studies. Supplement 2: The significant pleiotropy or heterogeneity of IVs in the MR analysis using gut microbiota as the exposure and osteonecrosis as the outcome. Supplement 3: The scatterplots and leave-one-out plots in the MR analysis using gut microbiota as the exposure and osteonecrosis as the outcome. Supplement 4: The significant pleiotropy or heterogeneity of IVs in the MR analysis using immune cells as the exposure and osteonecrosis as the outcome. Supplement 5: The scatterplots and leave-one-out plots in the MR analysis using immune cells as the exposure and osteonecrosis as the outcome. Supplement 6: The significant pleiotropy or heterogeneity of IVs in the MR analysis using gut microbiota as the exposure and immune cells as the outcome. Supplement 7: The scatterplots and leave-one-out plots in the MR analysis using gut microbiota as the exposure and immune cells as the outcome. Supplement 8: The results of the MR analysis using osteonecrosis as the exposure and gut microbiota and immune cells as the outcomes. [file 9323113.f1.zip › Supplement 3/GCST90027823/sensitivity-analysis.pdf]

# MR Test

- Inverse variance weighted
- MR Egger
- Simple mode
- Weighted median
- Weighted mode

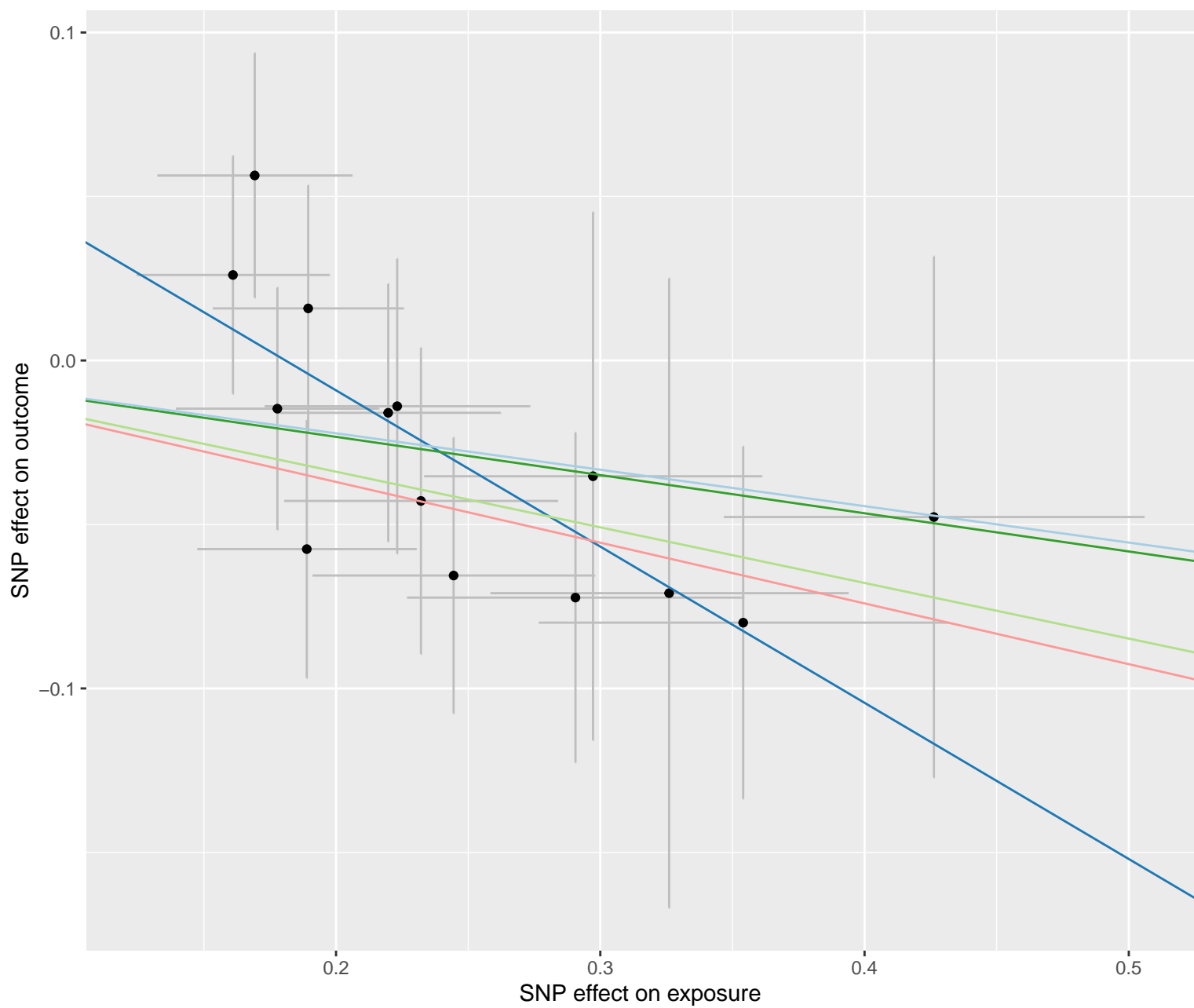

Supplement: Supporting Information — Supplement 1: The STROBE-MR checklist of recommended items to address in reports of Mendelian randomization studies. Supplement 2: The significant pleiotropy or heterogeneity of IVs in the MR analysis using gut microbiota as the exposure and osteonecrosis as the outcome. Supplement 3: The scatterplots and leave-one-out plots in the MR analysis using gut microbiota as the exposure and osteonecrosis as the outcome. Supplement 4: The significant pleiotropy or heterogeneity of IVs in the MR analysis using immune cells as the exposure and osteonecrosis as the outcome. Supplement 5: The scatterplots and leave-one-out plots in the MR analysis using immune cells as the exposure and osteonecrosis as the outcome. Supplement 6: The significant pleiotropy or heterogeneity of IVs in the MR analysis using gut microbiota as the exposure and immune cells as the outcome. Supplement 7: The scatterplots and leave-one-out plots in the MR analysis using gut microbiota as the exposure and immune cells as the outcome. Supplement 8: The results of the MR analysis using osteonecrosis as the exposure and gut microbiota and immune cells as the outcomes. [file 9323113.f1.zip › Supplement 3/GCST90027826/scatter.pdf]

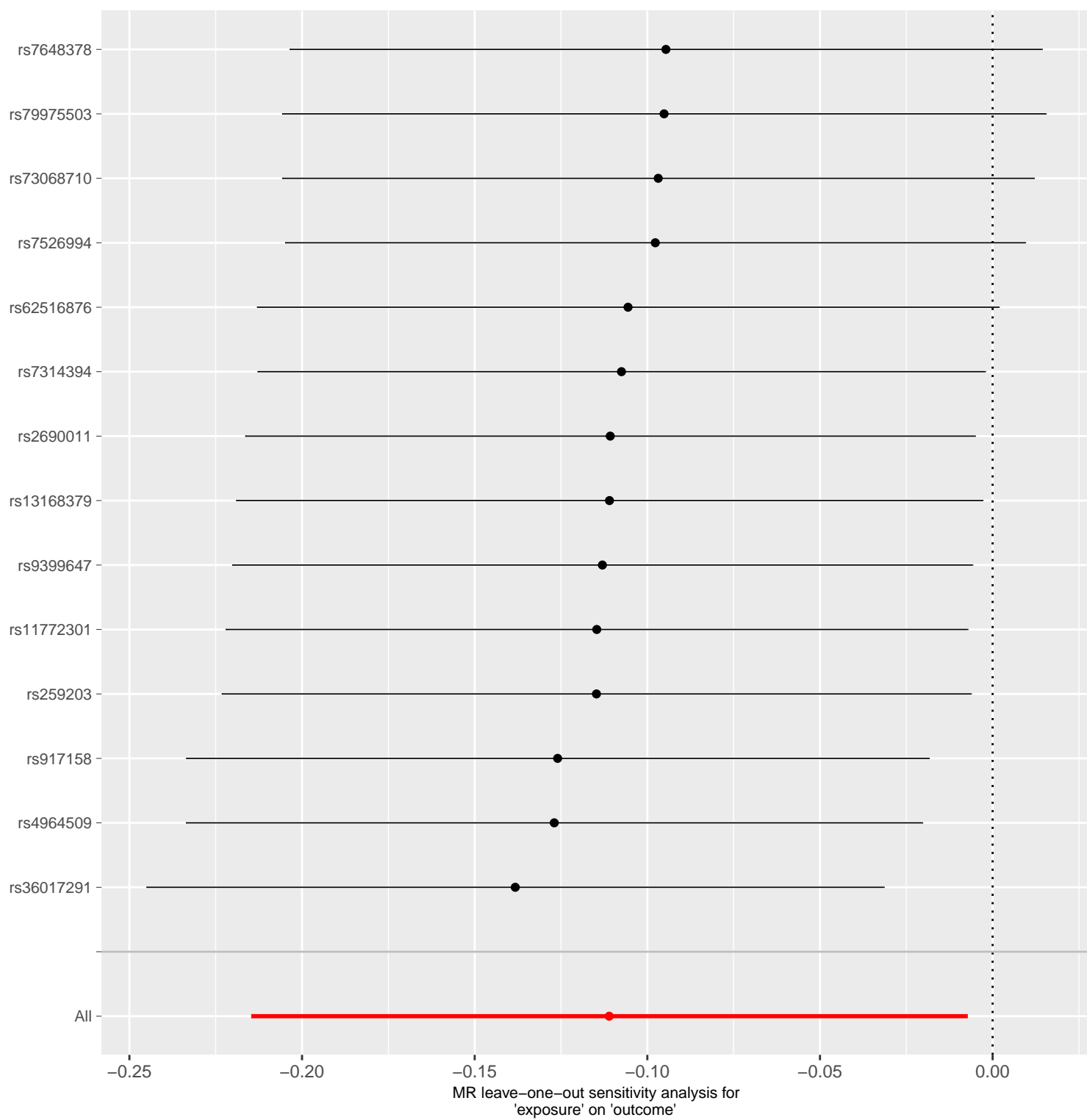

Supplement: Supporting Information — Supplement 1: The STROBE-MR checklist of recommended items to address in reports of Mendelian randomization studies. Supplement 2: The significant pleiotropy or heterogeneity of IVs in the MR analysis using gut microbiota as the exposure and osteonecrosis as the outcome. Supplement 3: The scatterplots and leave-one-out plots in the MR analysis using gut microbiota as the exposure and osteonecrosis as the outcome. Supplement 4: The significant pleiotropy or heterogeneity of IVs in the MR analysis using immune cells as the exposure and osteonecrosis as the outcome. Supplement 5: The scatterplots and leave-one-out plots in the MR analysis using immune cells as the exposure and osteonecrosis as the outcome. Supplement 6: The significant pleiotropy or heterogeneity of IVs in the MR analysis using gut microbiota as the exposure and immune cells as the outcome. Supplement 7: The scatterplots and leave-one-out plots in the MR analysis using gut microbiota as the exposure and immune cells as the outcome. Supplement 8: The results of the MR analysis using osteonecrosis as the exposure and gut microbiota and immune cells as the outcomes. [file 9323113.f1.zip › Supplement 3/GCST90027826/sensitivity-analysis.pdf]

# MR Test

- Inverse variance weighted
- MR Egger
- Simple mode
- Weighted median
- Weighted mode

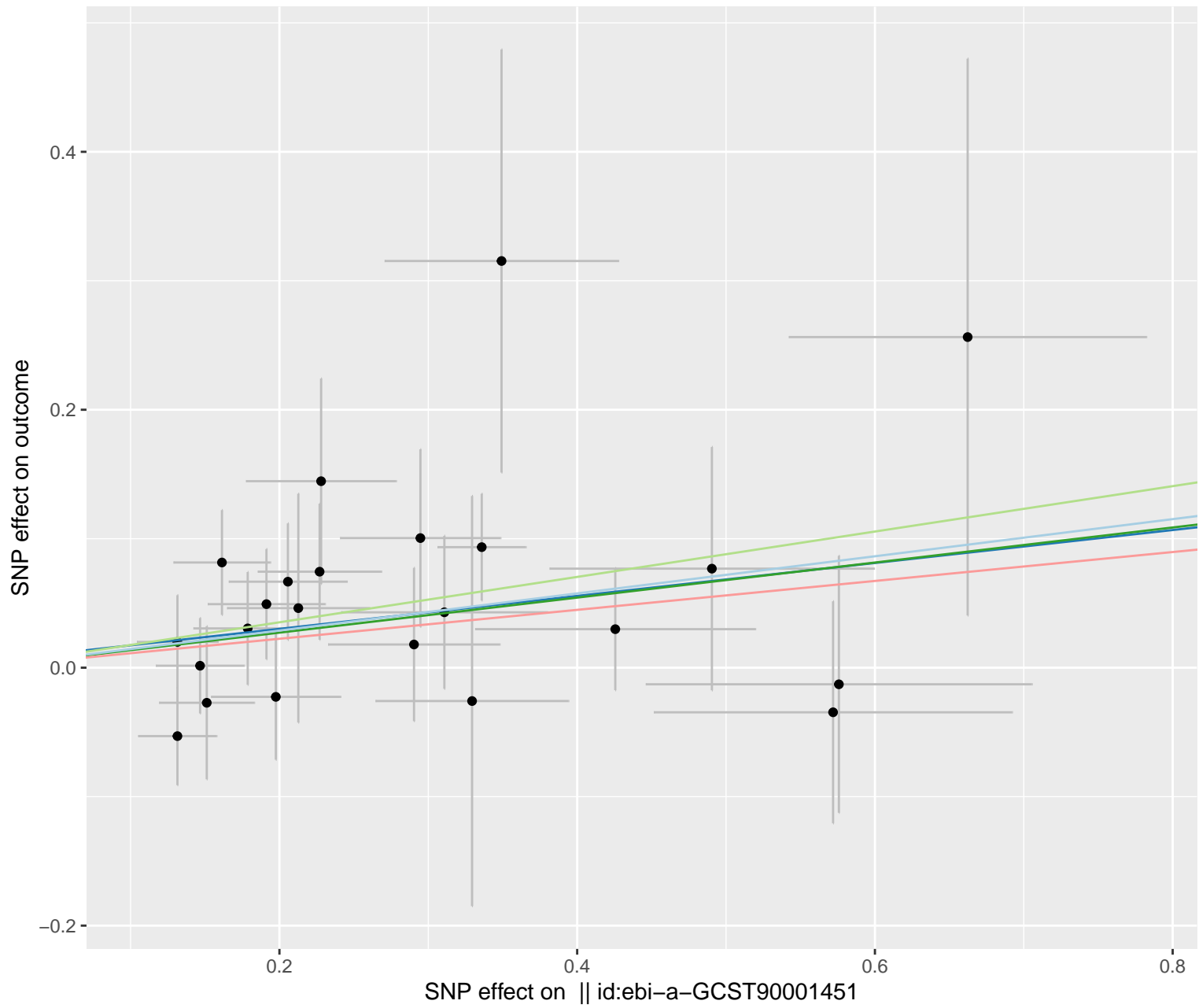

Supplement: Supporting Information — Supplement 1: The STROBE-MR checklist of recommended items to address in reports of Mendelian randomization studies. Supplement 2: The significant pleiotropy or heterogeneity of IVs in the MR analysis using gut microbiota as the exposure and osteonecrosis as the outcome. Supplement 3: The scatterplots and leave-one-out plots in the MR analysis using gut microbiota as the exposure and osteonecrosis as the outcome. Supplement 4: The significant pleiotropy or heterogeneity of IVs in the MR analysis using immune cells as the exposure and osteonecrosis as the outcome. Supplement 5: The scatterplots and leave-one-out plots in the MR analysis using immune cells as the exposure and osteonecrosis as the outcome. Supplement 6: The significant pleiotropy or heterogeneity of IVs in the MR analysis using gut microbiota as the exposure and immune cells as the outcome. Supplement 7: The scatterplots and leave-one-out plots in the MR analysis using gut microbiota as the exposure and immune cells as the outcome. Supplement 8: The results of the MR analysis using osteonecrosis as the exposure and gut microbiota and immune cells as the outcomes. [file 9323113.f1.zip › Supplement 5/ebi-a-GCST90001451/scatter.pdf]

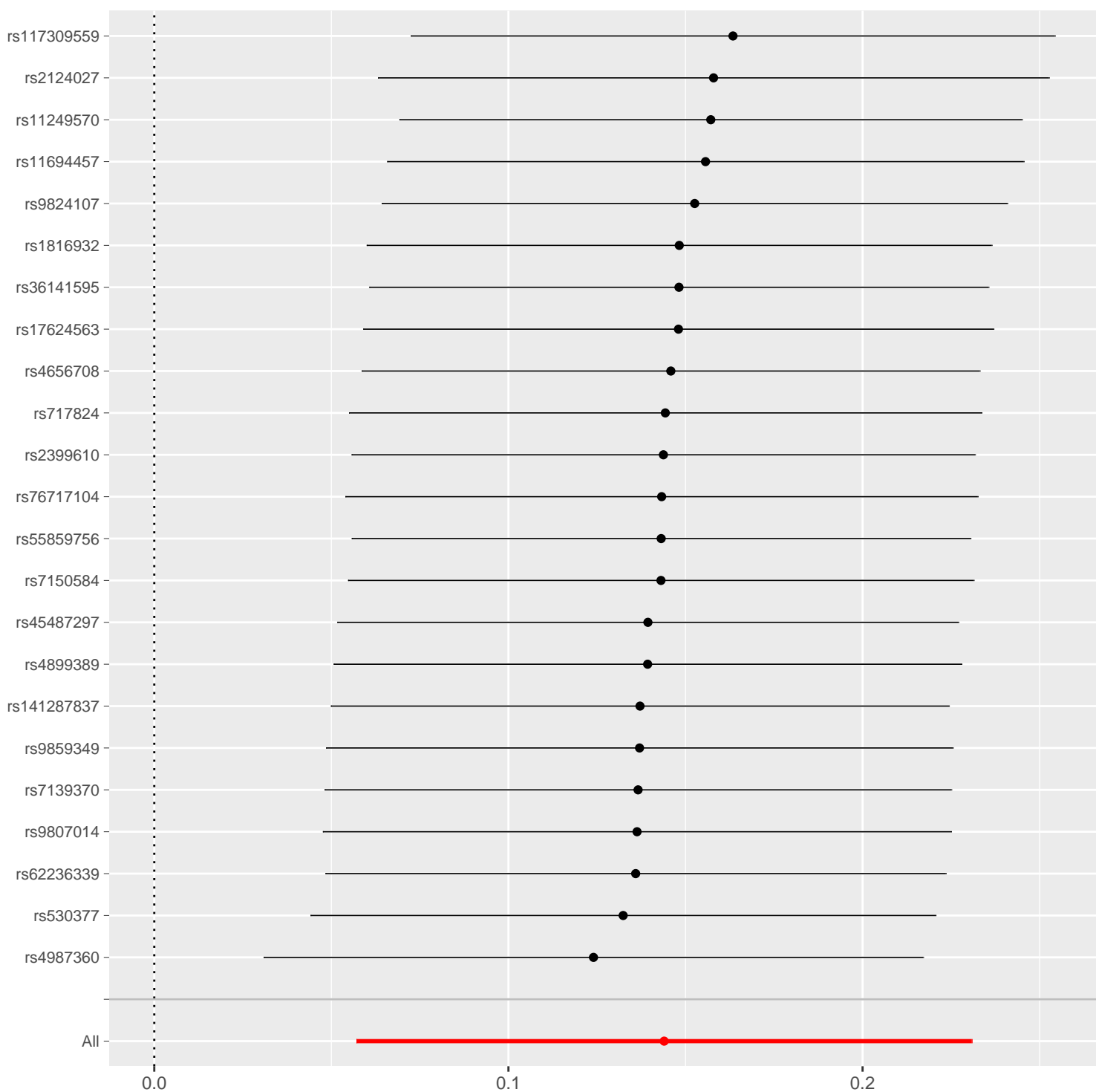

MR leave-one-out sensitivity analysis for  
' || id:ebi-a-GCST90001451' on 'outcome'

Supplement: Supporting Information — Supplement 1: The STROBE-MR checklist of recommended items to address in reports of Mendelian randomization studies. Supplement 2: The significant pleiotropy or heterogeneity of IVs in the MR analysis using gut microbiota as the exposure and osteonecrosis as the outcome. Supplement 3: The scatterplots and leave-one-out plots in the MR analysis using gut microbiota as the exposure and osteonecrosis as the outcome. Supplement 4: The significant pleiotropy or heterogeneity of IVs in the MR analysis using immune cells as the exposure and osteonecrosis as the outcome. Supplement 5: The scatterplots and leave-one-out plots in the MR analysis using immune cells as the exposure and osteonecrosis as the outcome. Supplement 6: The significant pleiotropy or heterogeneity of IVs in the MR analysis using gut microbiota as the exposure and immune cells as the outcome. Supplement 7: The scatterplots and leave-one-out plots in the MR analysis using gut microbiota as the exposure and immune cells as the outcome. Supplement 8: The results of the MR analysis using osteonecrosis as the exposure and gut microbiota and immune cells as the outcomes. [file 9323113.f1.zip › Supplement 5/ebi-a-GCST90001451/sensitivity-analysis.pdf]

# MR Test

- Inverse variance weighted
- MR Egger
- Simple mode
- Weighted median
- Weighted mode

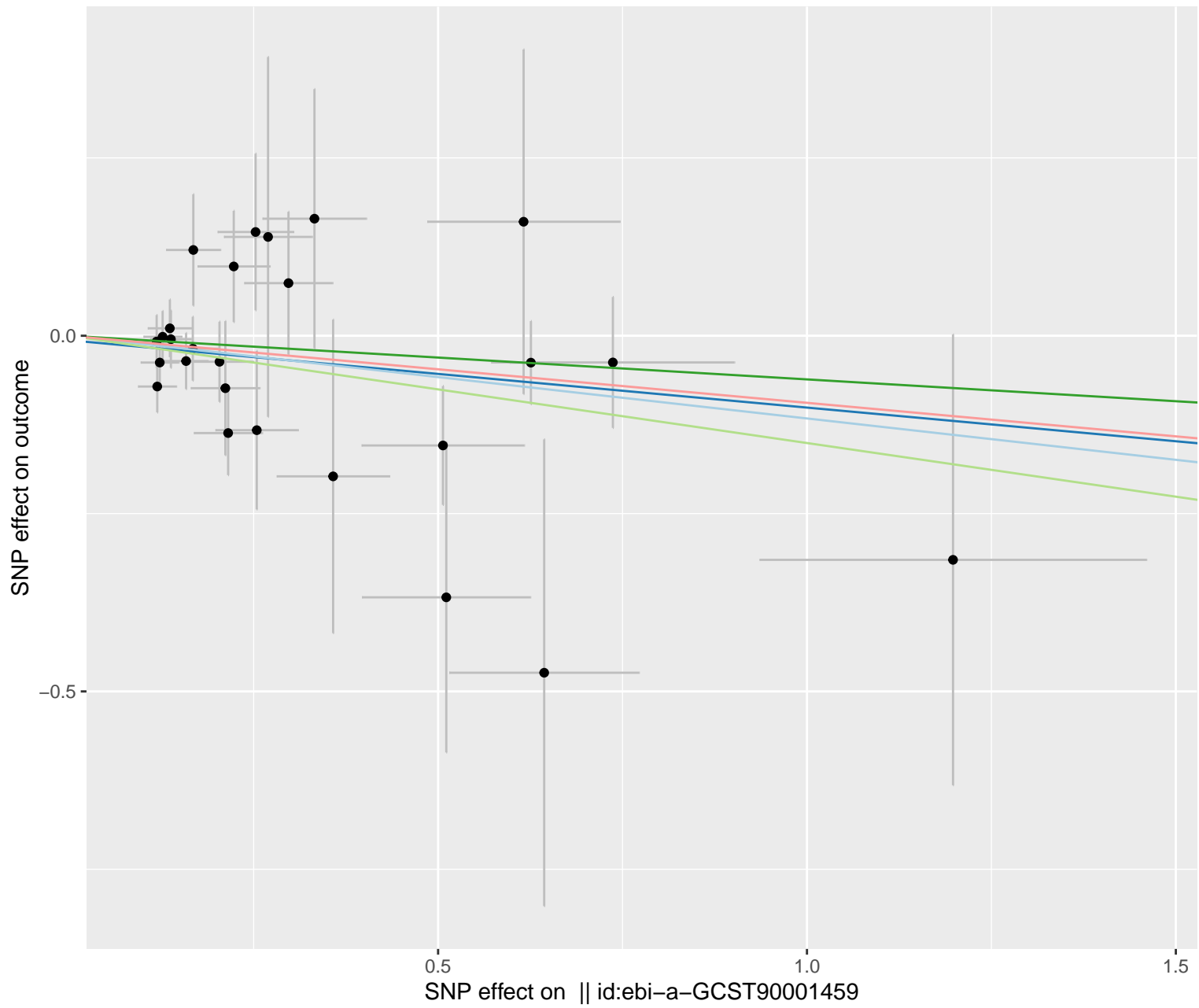

Supplement: Supporting Information — Supplement 1: The STROBE-MR checklist of recommended items to address in reports of Mendelian randomization studies. Supplement 2: The significant pleiotropy or heterogeneity of IVs in the MR analysis using gut microbiota as the exposure and osteonecrosis as the outcome. Supplement 3: The scatterplots and leave-one-out plots in the MR analysis using gut microbiota as the exposure and osteonecrosis as the outcome. Supplement 4: The significant pleiotropy or heterogeneity of IVs in the MR analysis using immune cells as the exposure and osteonecrosis as the outcome. Supplement 5: The scatterplots and leave-one-out plots in the MR analysis using immune cells as the exposure and osteonecrosis as the outcome. Supplement 6: The significant pleiotropy or heterogeneity of IVs in the MR analysis using gut microbiota as the exposure and immune cells as the outcome. Supplement 7: The scatterplots and leave-one-out plots in the MR analysis using gut microbiota as the exposure and immune cells as the outcome. Supplement 8: The results of the MR analysis using osteonecrosis as the exposure and gut microbiota and immune cells as the outcomes. [file 9323113.f1.zip › Supplement 5/ebi-a-GCST90001459/scatter.pdf]

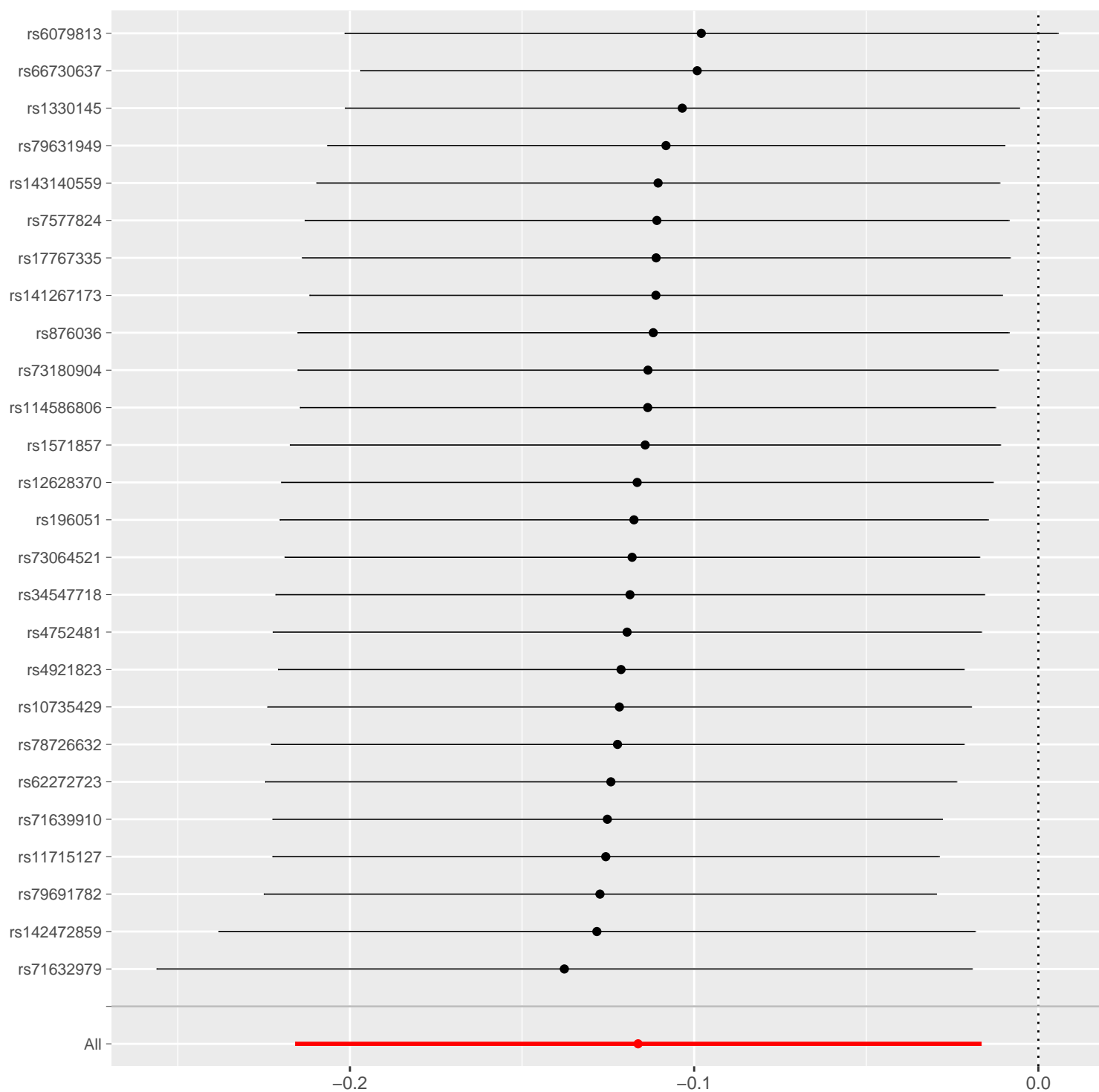

Supplement: Supporting Information — Supplement 1: The STROBE-MR checklist of recommended items to address in reports of Mendelian randomization studies. Supplement 2: The significant pleiotropy or heterogeneity of IVs in the MR analysis using gut microbiota as the exposure and osteonecrosis as the outcome. Supplement 3: The scatterplots and leave-one-out plots in the MR analysis using gut microbiota as the exposure and osteonecrosis as the outcome. Supplement 4: The significant pleiotropy or heterogeneity of IVs in the MR analysis using immune cells as the exposure and osteonecrosis as the outcome. Supplement 5: The scatterplots and leave-one-out plots in the MR analysis using immune cells as the exposure and osteonecrosis as the outcome. Supplement 6: The significant pleiotropy or heterogeneity of IVs in the MR analysis using gut microbiota as the exposure and immune cells as the outcome. Supplement 7: The scatterplots and leave-one-out plots in the MR analysis using gut microbiota as the exposure and immune cells as the outcome. Supplement 8: The results of the MR analysis using osteonecrosis as the exposure and gut microbiota and immune cells as the outcomes. [file 9323113.f1.zip › Supplement 5/ebi-a-GCST90001459/sensitivity-analysis.pdf]

# MR Test

- Inverse variance weighted
- MR Egger
- Simple mode
- Weighted median
- Weighted mode

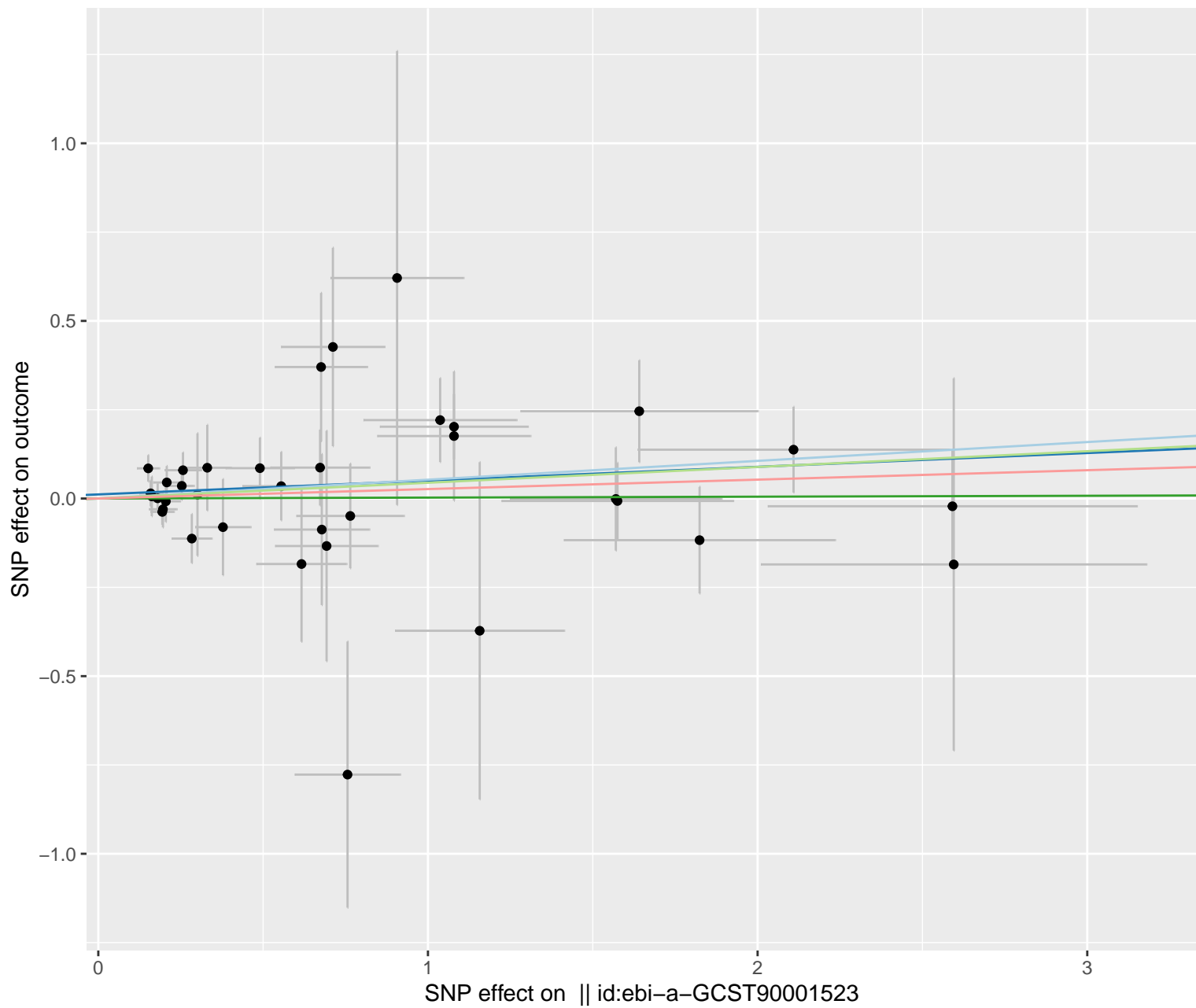

Supplement: Supporting Information — Supplement 1: The STROBE-MR checklist of recommended items to address in reports of Mendelian randomization studies. Supplement 2: The significant pleiotropy or heterogeneity of IVs in the MR analysis using gut microbiota as the exposure and osteonecrosis as the outcome. Supplement 3: The scatterplots and leave-one-out plots in the MR analysis using gut microbiota as the exposure and osteonecrosis as the outcome. Supplement 4: The significant pleiotropy or heterogeneity of IVs in the MR analysis using immune cells as the exposure and osteonecrosis as the outcome. Supplement 5: The scatterplots and leave-one-out plots in the MR analysis using immune cells as the exposure and osteonecrosis as the outcome. Supplement 6: The significant pleiotropy or heterogeneity of IVs in the MR analysis using gut microbiota as the exposure and immune cells as the outcome. Supplement 7: The scatterplots and leave-one-out plots in the MR analysis using gut microbiota as the exposure and immune cells as the outcome. Supplement 8: The results of the MR analysis using osteonecrosis as the exposure and gut microbiota and immune cells as the outcomes. [file 9323113.f1.zip › Supplement 5/ebi-a-GCST90001523/scatter.pdf]

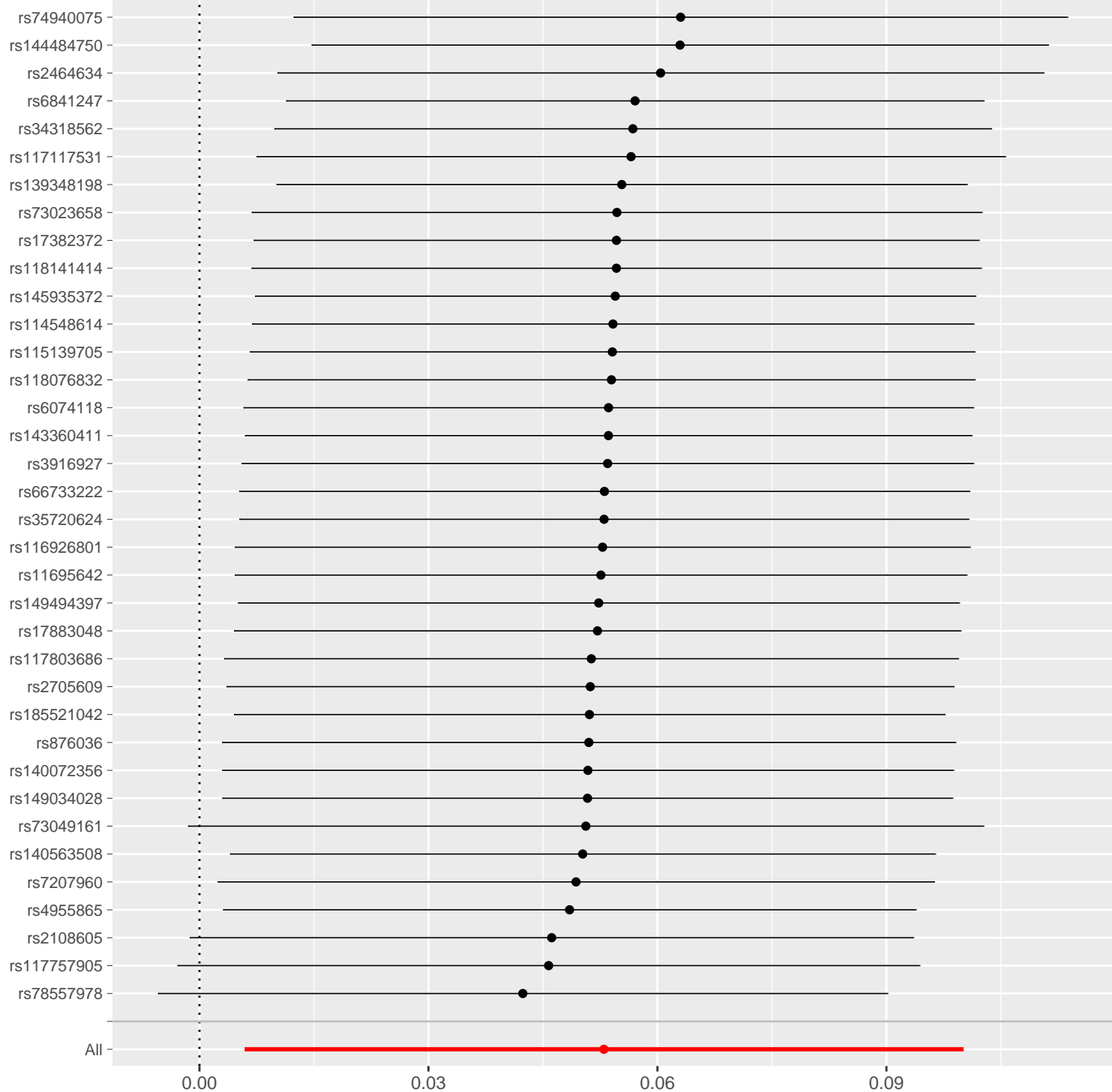

Supplement: Supporting Information — Supplement 1: The STROBE-MR checklist of recommended items to address in reports of Mendelian randomization studies. Supplement 2: The significant pleiotropy or heterogeneity of IVs in the MR analysis using gut microbiota as the exposure and osteonecrosis as the outcome. Supplement 3: The scatterplots and leave-one-out plots in the MR analysis using gut microbiota as the exposure and osteonecrosis as the outcome. Supplement 4: The significant pleiotropy or heterogeneity of IVs in the MR analysis using immune cells as the exposure and osteonecrosis as the outcome. Supplement 5: The scatterplots and leave-one-out plots in the MR analysis using immune cells as the exposure and osteonecrosis as the outcome. Supplement 6: The significant pleiotropy or heterogeneity of IVs in the MR analysis using gut microbiota as the exposure and immune cells as the outcome. Supplement 7: The scatterplots and leave-one-out plots in the MR analysis using gut microbiota as the exposure and immune cells as the outcome. Supplement 8: The results of the MR analysis using osteonecrosis as the exposure and gut microbiota and immune cells as the outcomes. [file 9323113.f1.zip › Supplement 5/ebi-a-GCST90001523/sensitivity-analysis.pdf]

# MR Test

- Inverse variance weighted
- MR Egger
- Simple mode
- Weighted median
- Weighted mode

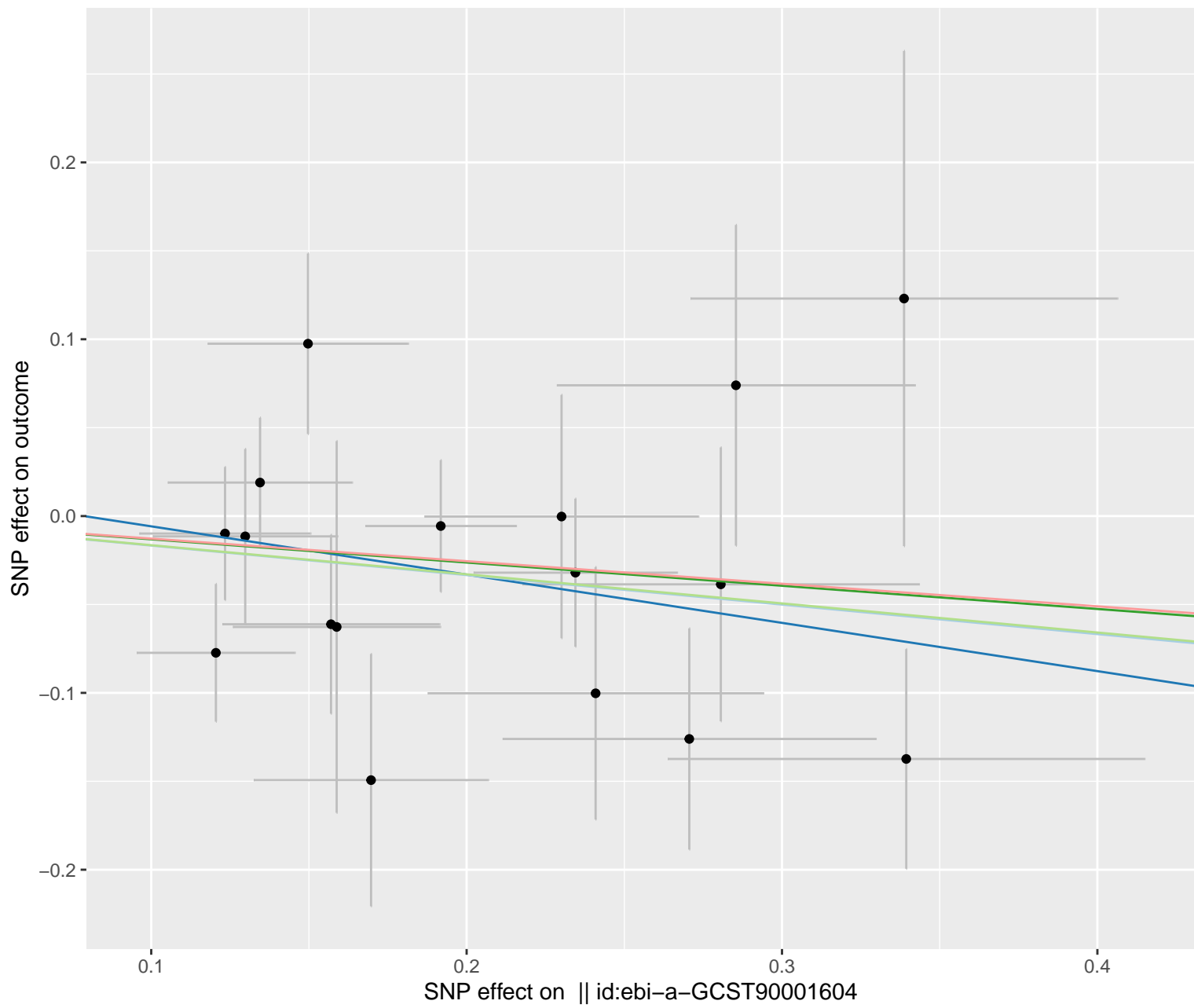

Supplement: Supporting Information — Supplement 1: The STROBE-MR checklist of recommended items to address in reports of Mendelian randomization studies. Supplement 2: The significant pleiotropy or heterogeneity of IVs in the MR analysis using gut microbiota as the exposure and osteonecrosis as the outcome. Supplement 3: The scatterplots and leave-one-out plots in the MR analysis using gut microbiota as the exposure and osteonecrosis as the outcome. Supplement 4: The significant pleiotropy or heterogeneity of IVs in the MR analysis using immune cells as the exposure and osteonecrosis as the outcome. Supplement 5: The scatterplots and leave-one-out plots in the MR analysis using immune cells as the exposure and osteonecrosis as the outcome. Supplement 6: The significant pleiotropy or heterogeneity of IVs in the MR analysis using gut microbiota as the exposure and immune cells as the outcome. Supplement 7: The scatterplots and leave-one-out plots in the MR analysis using gut microbiota as the exposure and immune cells as the outcome. Supplement 8: The results of the MR analysis using osteonecrosis as the exposure and gut microbiota and immune cells as the outcomes. [file 9323113.f1.zip › Supplement 5/ebi-a-GCST90001604/scatter.pdf]

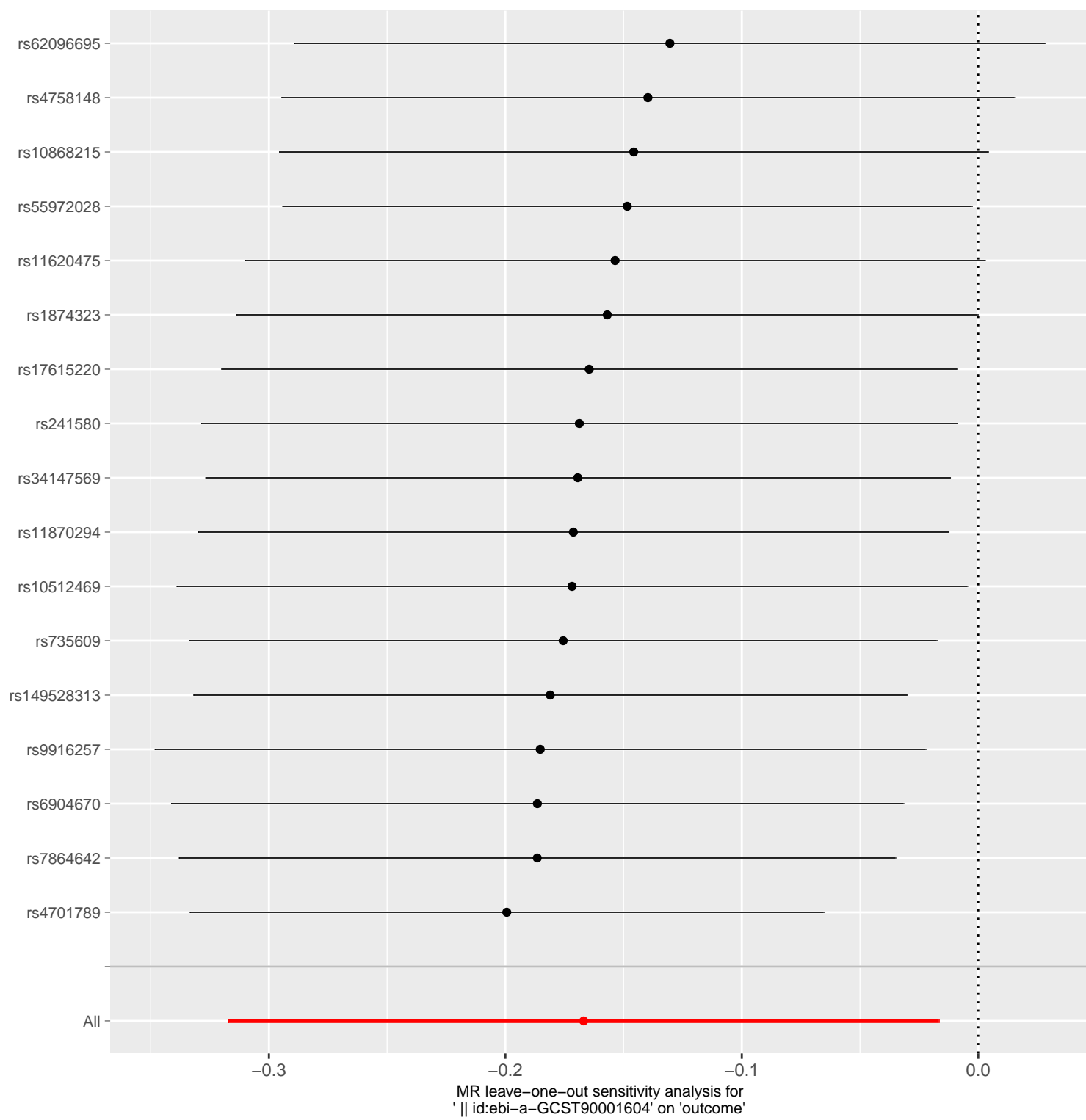

Supplement: Supporting Information — Supplement 1: The STROBE-MR checklist of recommended items to address in reports of Mendelian randomization studies. Supplement 2: The significant pleiotropy or heterogeneity of IVs in the MR analysis using gut microbiota as the exposure and osteonecrosis as the outcome. Supplement 3: The scatterplots and leave-one-out plots in the MR analysis using gut microbiota as the exposure and osteonecrosis as the outcome. Supplement 4: The significant pleiotropy or heterogeneity of IVs in the MR analysis using immune cells as the exposure and osteonecrosis as the outcome. Supplement 5: The scatterplots and leave-one-out plots in the MR analysis using immune cells as the exposure and osteonecrosis as the outcome. Supplement 6: The significant pleiotropy or heterogeneity of IVs in the MR analysis using gut microbiota as the exposure and immune cells as the outcome. Supplement 7: The scatterplots and leave-one-out plots in the MR analysis using gut microbiota as the exposure and immune cells as the outcome. Supplement 8: The results of the MR analysis using osteonecrosis as the exposure and gut microbiota and immune cells as the outcomes. [file 9323113.f1.zip › Supplement 5/ebi-a-GCST90001604/sensitivity-analysis.pdf]

# MR Test

- Inverse variance weighted
- MR Egger
- Simple mode
- Weighted median
- Weighted mode

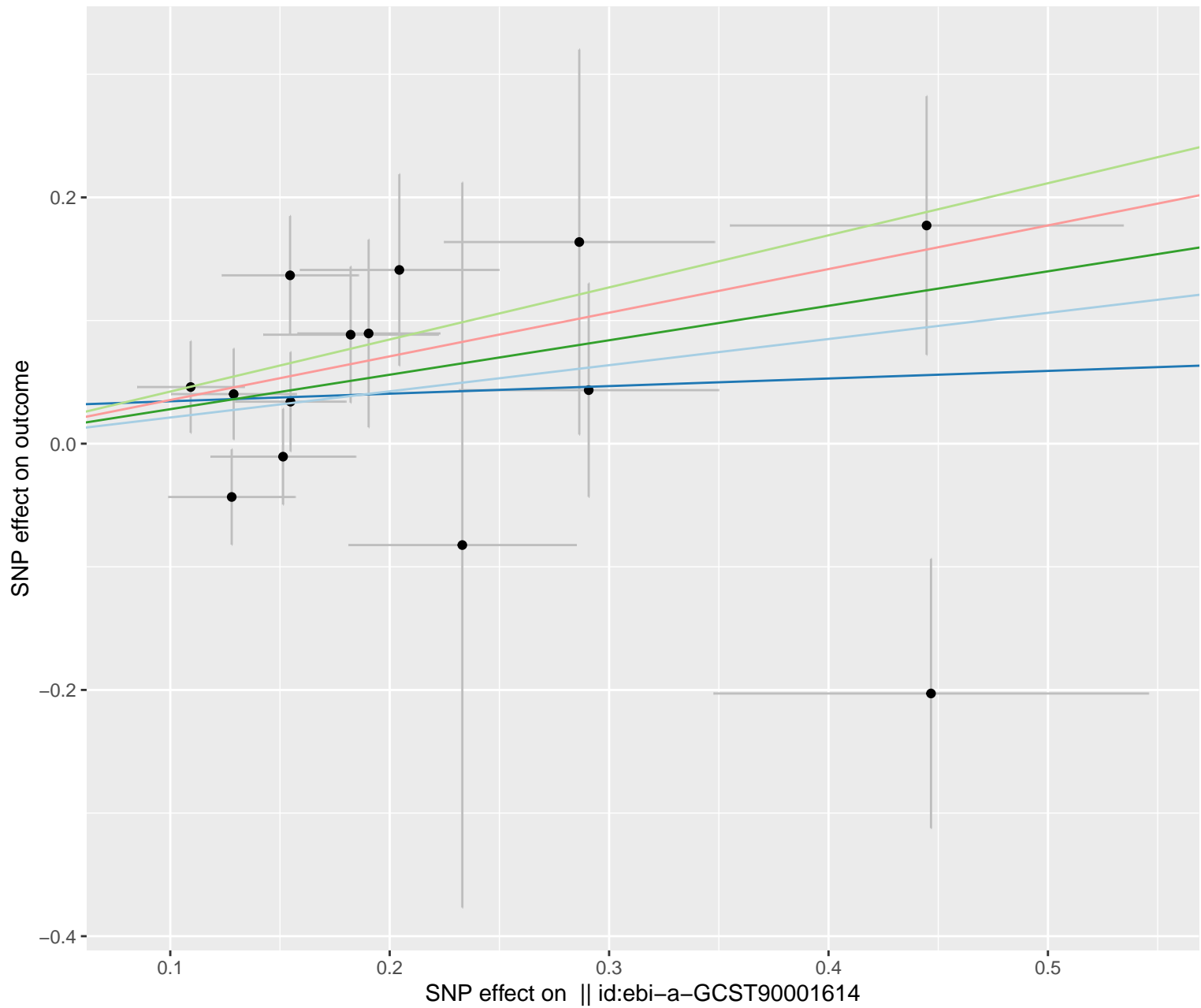

Supplement: Supporting Information — Supplement 1: The STROBE-MR checklist of recommended items to address in reports of Mendelian randomization studies. Supplement 2: The significant pleiotropy or heterogeneity of IVs in the MR analysis using gut microbiota as the exposure and osteonecrosis as the outcome. Supplement 3: The scatterplots and leave-one-out plots in the MR analysis using gut microbiota as the exposure and osteonecrosis as the outcome. Supplement 4: The significant pleiotropy or heterogeneity of IVs in the MR analysis using immune cells as the exposure and osteonecrosis as the outcome. Supplement 5: The scatterplots and leave-one-out plots in the MR analysis using immune cells as the exposure and osteonecrosis as the outcome. Supplement 6: The significant pleiotropy or heterogeneity of IVs in the MR analysis using gut microbiota as the exposure and immune cells as the outcome. Supplement 7: The scatterplots and leave-one-out plots in the MR analysis using gut microbiota as the exposure and immune cells as the outcome. Supplement 8: The results of the MR analysis using osteonecrosis as the exposure and gut microbiota and immune cells as the outcomes. [file 9323113.f1.zip › Supplement 5/ebi-a-GCST90001614/scatter.pdf]

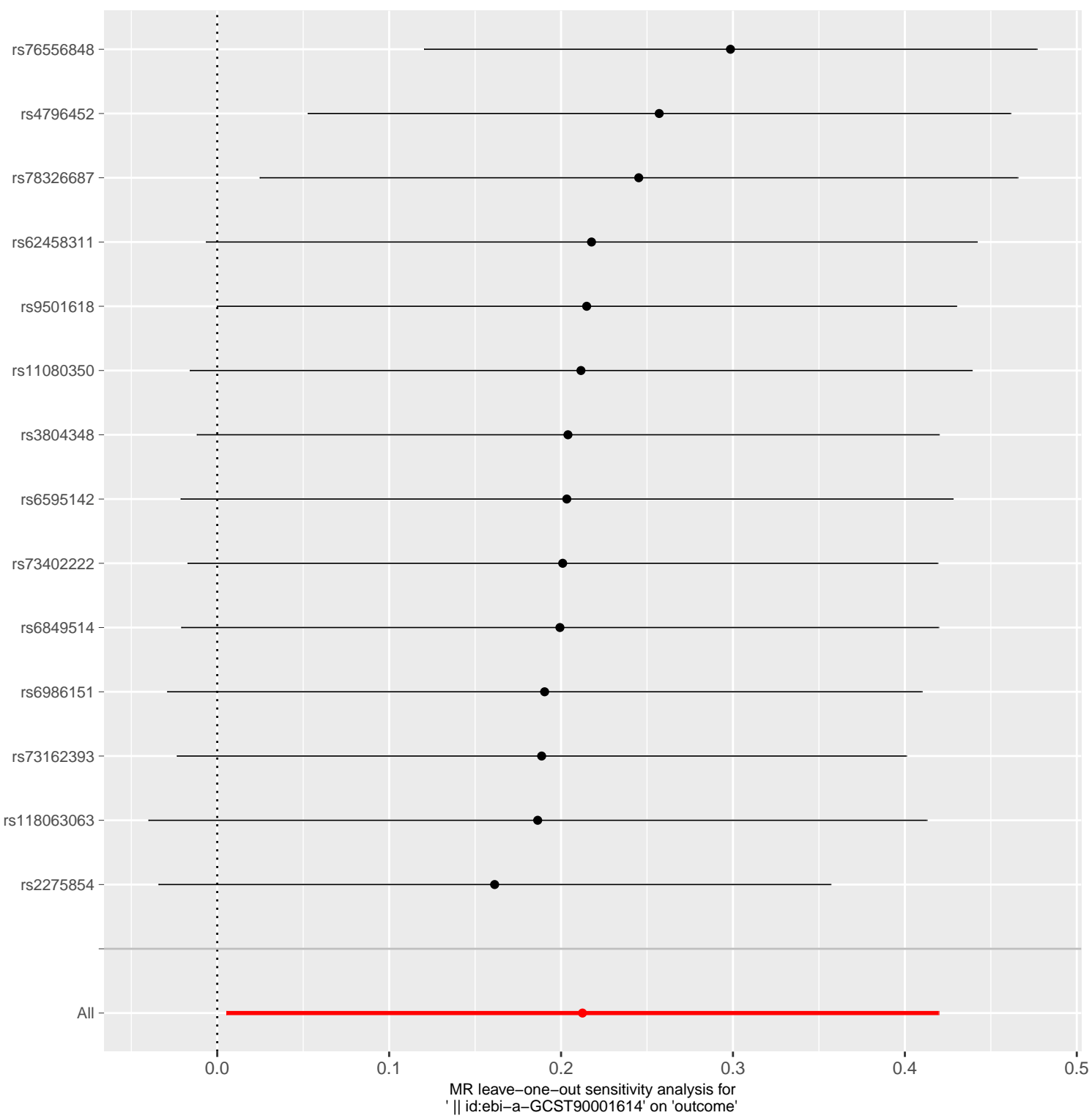

Supplement: Supporting Information — Supplement 1: The STROBE-MR checklist of recommended items to address in reports of Mendelian randomization studies. Supplement 2: The significant pleiotropy or heterogeneity of IVs in the MR analysis using gut microbiota as the exposure and osteonecrosis as the outcome. Supplement 3: The scatterplots and leave-one-out plots in the MR analysis using gut microbiota as the exposure and osteonecrosis as the outcome. Supplement 4: The significant pleiotropy or heterogeneity of IVs in the MR analysis using immune cells as the exposure and osteonecrosis as the outcome. Supplement 5: The scatterplots and leave-one-out plots in the MR analysis using immune cells as the exposure and osteonecrosis as the outcome. Supplement 6: The significant pleiotropy or heterogeneity of IVs in the MR analysis using gut microbiota as the exposure and immune cells as the outcome. Supplement 7: The scatterplots and leave-one-out plots in the MR analysis using gut microbiota as the exposure and immune cells as the outcome. Supplement 8: The results of the MR analysis using osteonecrosis as the exposure and gut microbiota and immune cells as the outcomes. [file 9323113.f1.zip › Supplement 5/ebi-a-GCST90001614/sensitivity-analysis.pdf]

# MR Test

- Inverse variance weighted
- MR Egger
- Simple mode
- Weighted median
- Weighted mode

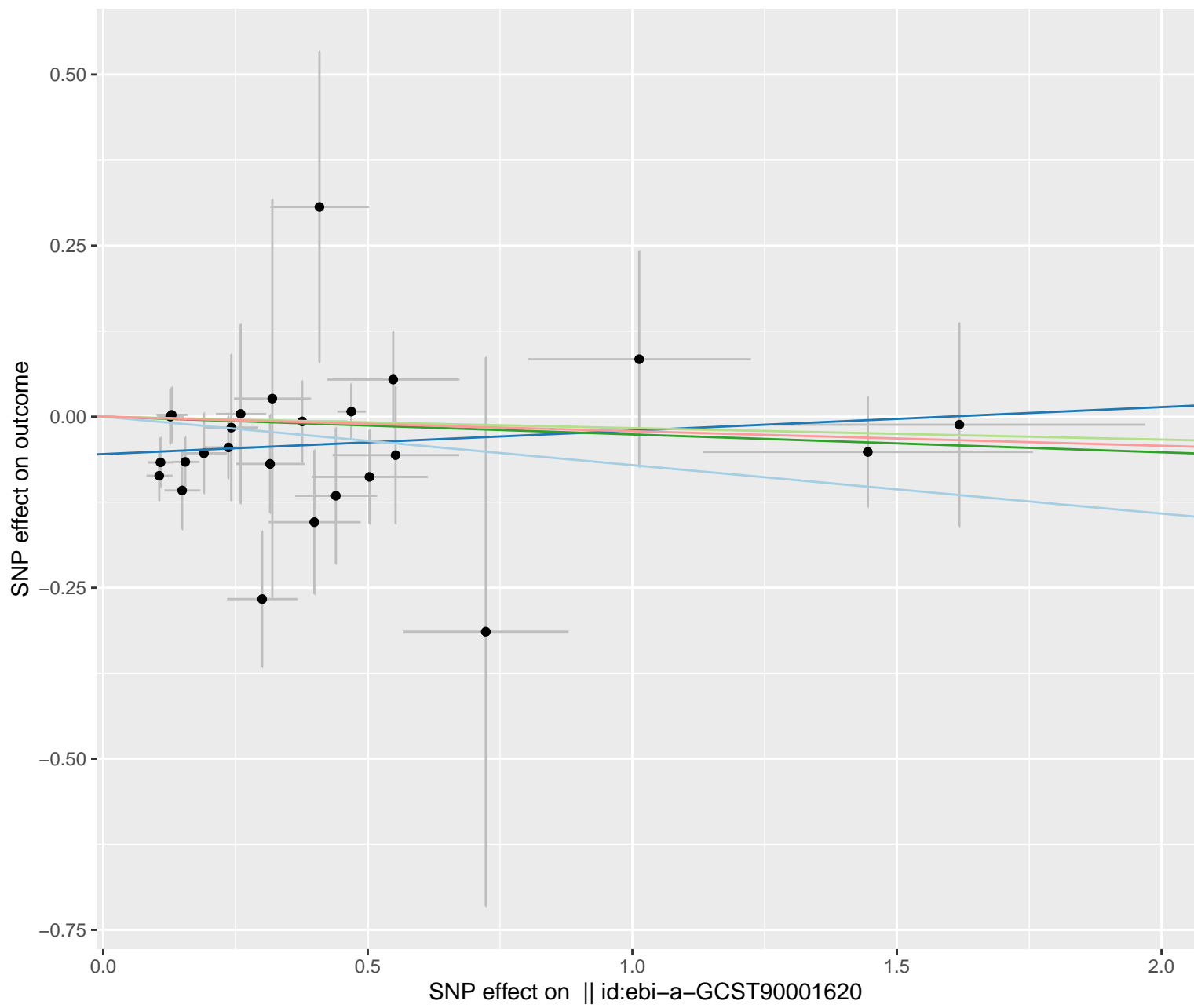

Supplement: Supporting Information — Supplement 1: The STROBE-MR checklist of recommended items to address in reports of Mendelian randomization studies. Supplement 2: The significant pleiotropy or heterogeneity of IVs in the MR analysis using gut microbiota as the exposure and osteonecrosis as the outcome. Supplement 3: The scatterplots and leave-one-out plots in the MR analysis using gut microbiota as the exposure and osteonecrosis as the outcome. Supplement 4: The significant pleiotropy or heterogeneity of IVs in the MR analysis using immune cells as the exposure and osteonecrosis as the outcome. Supplement 5: The scatterplots and leave-one-out plots in the MR analysis using immune cells as the exposure and osteonecrosis as the outcome. Supplement 6: The significant pleiotropy or heterogeneity of IVs in the MR analysis using gut microbiota as the exposure and immune cells as the outcome. Supplement 7: The scatterplots and leave-one-out plots in the MR analysis using gut microbiota as the exposure and immune cells as the outcome. Supplement 8: The results of the MR analysis using osteonecrosis as the exposure and gut microbiota and immune cells as the outcomes. [file 9323113.f1.zip › Supplement 5/ebi-a-GCST90001620/scatter.pdf]

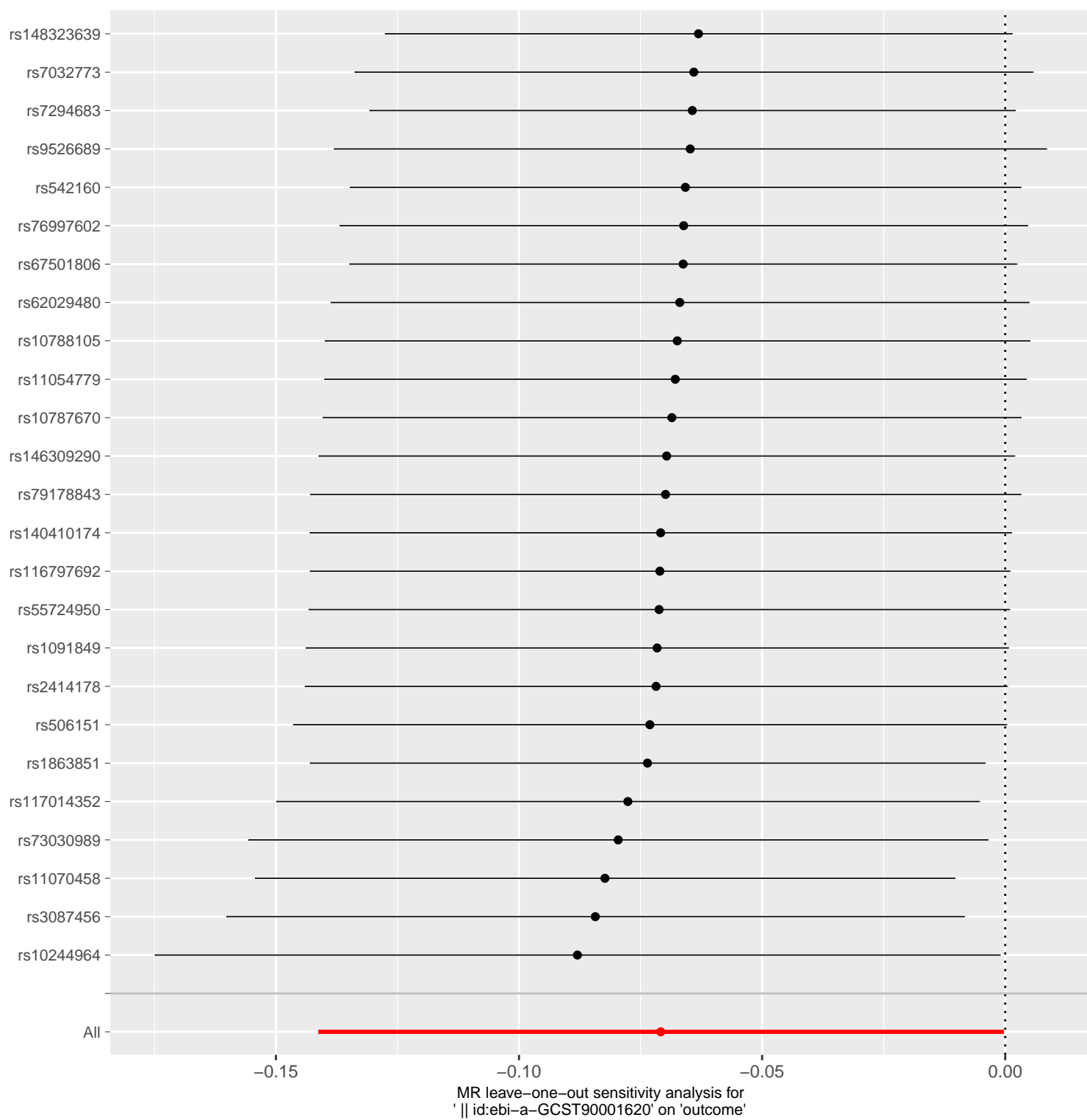

Supplement: Supporting Information — Supplement 1: The STROBE-MR checklist of recommended items to address in reports of Mendelian randomization studies. Supplement 2: The significant pleiotropy or heterogeneity of IVs in the MR analysis using gut microbiota as the exposure and osteonecrosis as the outcome. Supplement 3: The scatterplots and leave-one-out plots in the MR analysis using gut microbiota as the exposure and osteonecrosis as the outcome. Supplement 4: The significant pleiotropy or heterogeneity of IVs in the MR analysis using immune cells as the exposure and osteonecrosis as the outcome. Supplement 5: The scatterplots and leave-one-out plots in the MR analysis using immune cells as the exposure and osteonecrosis as the outcome. Supplement 6: The significant pleiotropy or heterogeneity of IVs in the MR analysis using gut microbiota as the exposure and immune cells as the outcome. Supplement 7: The scatterplots and leave-one-out plots in the MR analysis using gut microbiota as the exposure and immune cells as the outcome. Supplement 8: The results of the MR analysis using osteonecrosis as the exposure and gut microbiota and immune cells as the outcomes. [file 9323113.f1.zip › Supplement 5/ebi-a-GCST90001620/sensitivity-analysis.pdf]

# MR Test

- Inverse variance weighted
- MR Egger
- Simple mode
- Weighted median
- Weighted mode

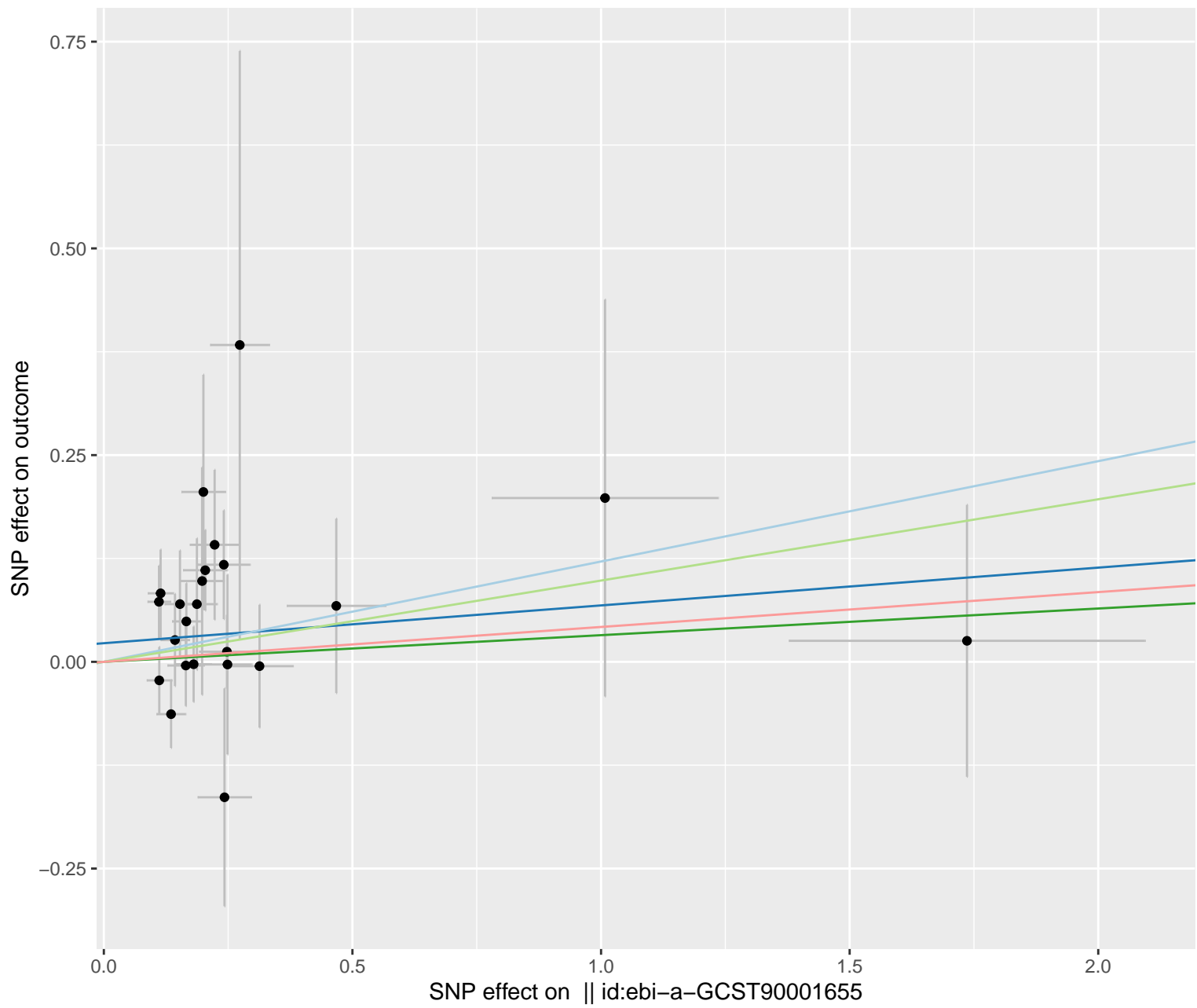

Supplement: Supporting Information — Supplement 1: The STROBE-MR checklist of recommended items to address in reports of Mendelian randomization studies. Supplement 2: The significant pleiotropy or heterogeneity of IVs in the MR analysis using gut microbiota as the exposure and osteonecrosis as the outcome. Supplement 3: The scatterplots and leave-one-out plots in the MR analysis using gut microbiota as the exposure and osteonecrosis as the outcome. Supplement 4: The significant pleiotropy or heterogeneity of IVs in the MR analysis using immune cells as the exposure and osteonecrosis as the outcome. Supplement 5: The scatterplots and leave-one-out plots in the MR analysis using immune cells as the exposure and osteonecrosis as the outcome. Supplement 6: The significant pleiotropy or heterogeneity of IVs in the MR analysis using gut microbiota as the exposure and immune cells as the outcome. Supplement 7: The scatterplots and leave-one-out plots in the MR analysis using gut microbiota as the exposure and immune cells as the outcome. Supplement 8: The results of the MR analysis using osteonecrosis as the exposure and gut microbiota and immune cells as the outcomes. [file 9323113.f1.zip › Supplement 5/ebi-a-GCST90001655/scatter.pdf]

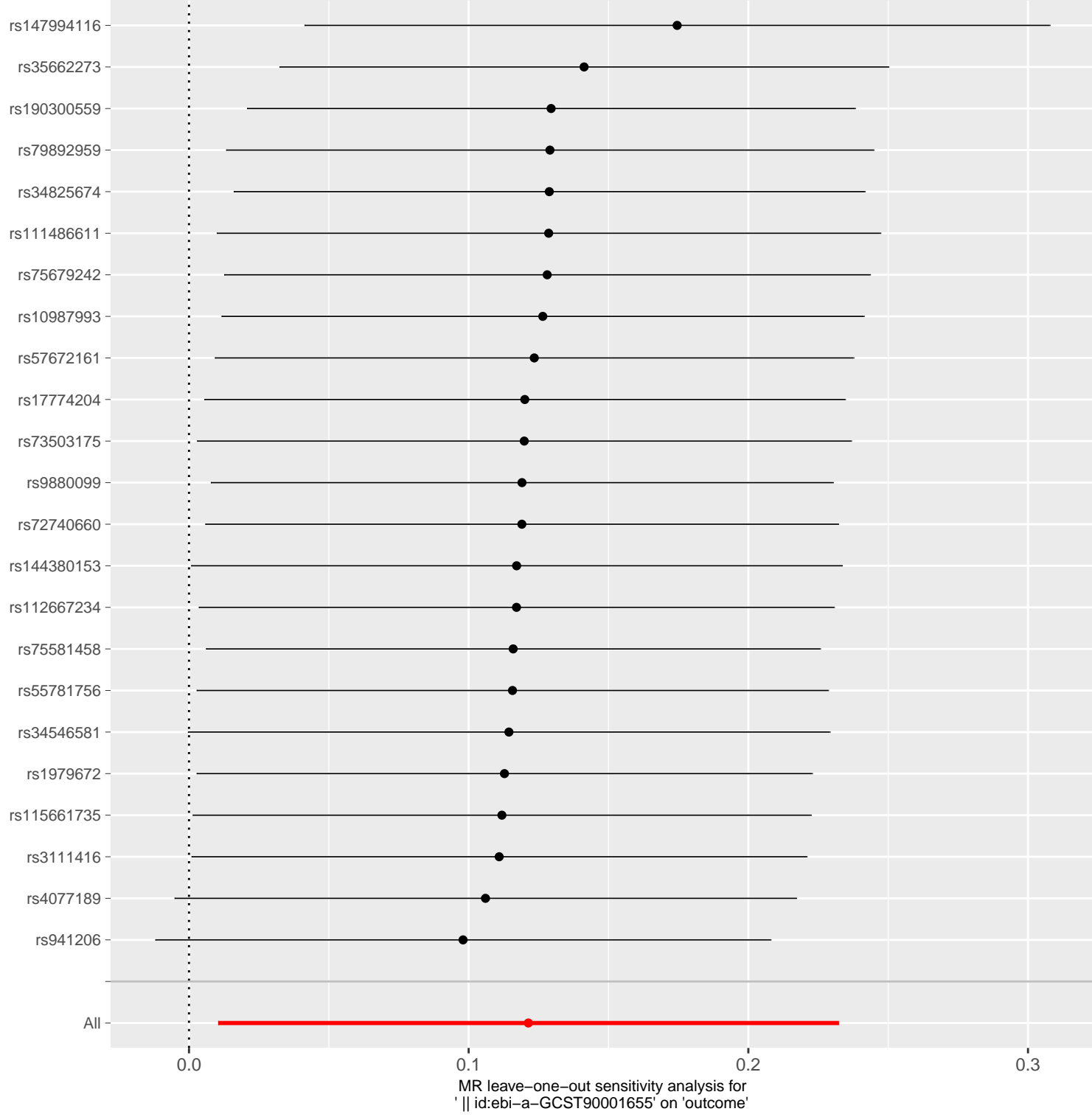

Supplement: Supporting Information — Supplement 1: The STROBE-MR checklist of recommended items to address in reports of Mendelian randomization studies. Supplement 2: The significant pleiotropy or heterogeneity of IVs in the MR analysis using gut microbiota as the exposure and osteonecrosis as the outcome. Supplement 3: The scatterplots and leave-one-out plots in the MR analysis using gut microbiota as the exposure and osteonecrosis as the outcome. Supplement 4: The significant pleiotropy or heterogeneity of IVs in the MR analysis using immune cells as the exposure and osteonecrosis as the outcome. Supplement 5: The scatterplots and leave-one-out plots in the MR analysis using immune cells as the exposure and osteonecrosis as the outcome. Supplement 6: The significant pleiotropy or heterogeneity of IVs in the MR analysis using gut microbiota as the exposure and immune cells as the outcome. Supplement 7: The scatterplots and leave-one-out plots in the MR analysis using gut microbiota as the exposure and immune cells as the outcome. Supplement 8: The results of the MR analysis using osteonecrosis as the exposure and gut microbiota and immune cells as the outcomes. [file 9323113.f1.zip › Supplement 5/ebi-a-GCST90001655/sensitivity-analysis.pdf]

# MR Test

- Inverse variance weighted
- MR Egger
- Simple mode
- Weighted median
- Weighted mode

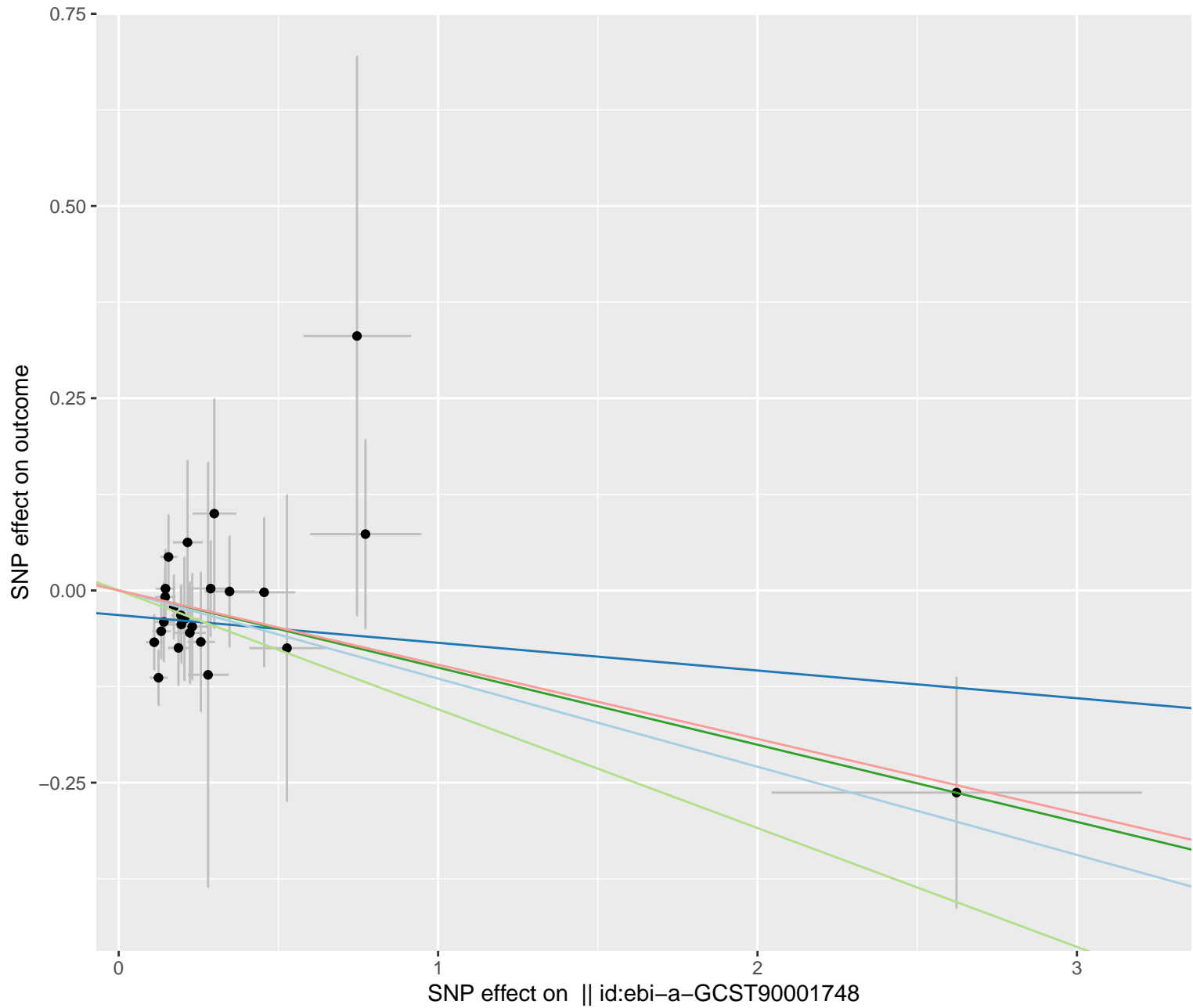

Supplement: Supporting Information — Supplement 1: The STROBE-MR checklist of recommended items to address in reports of Mendelian randomization studies. Supplement 2: The significant pleiotropy or heterogeneity of IVs in the MR analysis using gut microbiota as the exposure and osteonecrosis as the outcome. Supplement 3: The scatterplots and leave-one-out plots in the MR analysis using gut microbiota as the exposure and osteonecrosis as the outcome. Supplement 4: The significant pleiotropy or heterogeneity of IVs in the MR analysis using immune cells as the exposure and osteonecrosis as the outcome. Supplement 5: The scatterplots and leave-one-out plots in the MR analysis using immune cells as the exposure and osteonecrosis as the outcome. Supplement 6: The significant pleiotropy or heterogeneity of IVs in the MR analysis using gut microbiota as the exposure and immune cells as the outcome. Supplement 7: The scatterplots and leave-one-out plots in the MR analysis using gut microbiota as the exposure and immune cells as the outcome. Supplement 8: The results of the MR analysis using osteonecrosis as the exposure and gut microbiota and immune cells as the outcomes. [file 9323113.f1.zip › Supplement 5/ebi-a-GCST90001748/scatter.pdf]

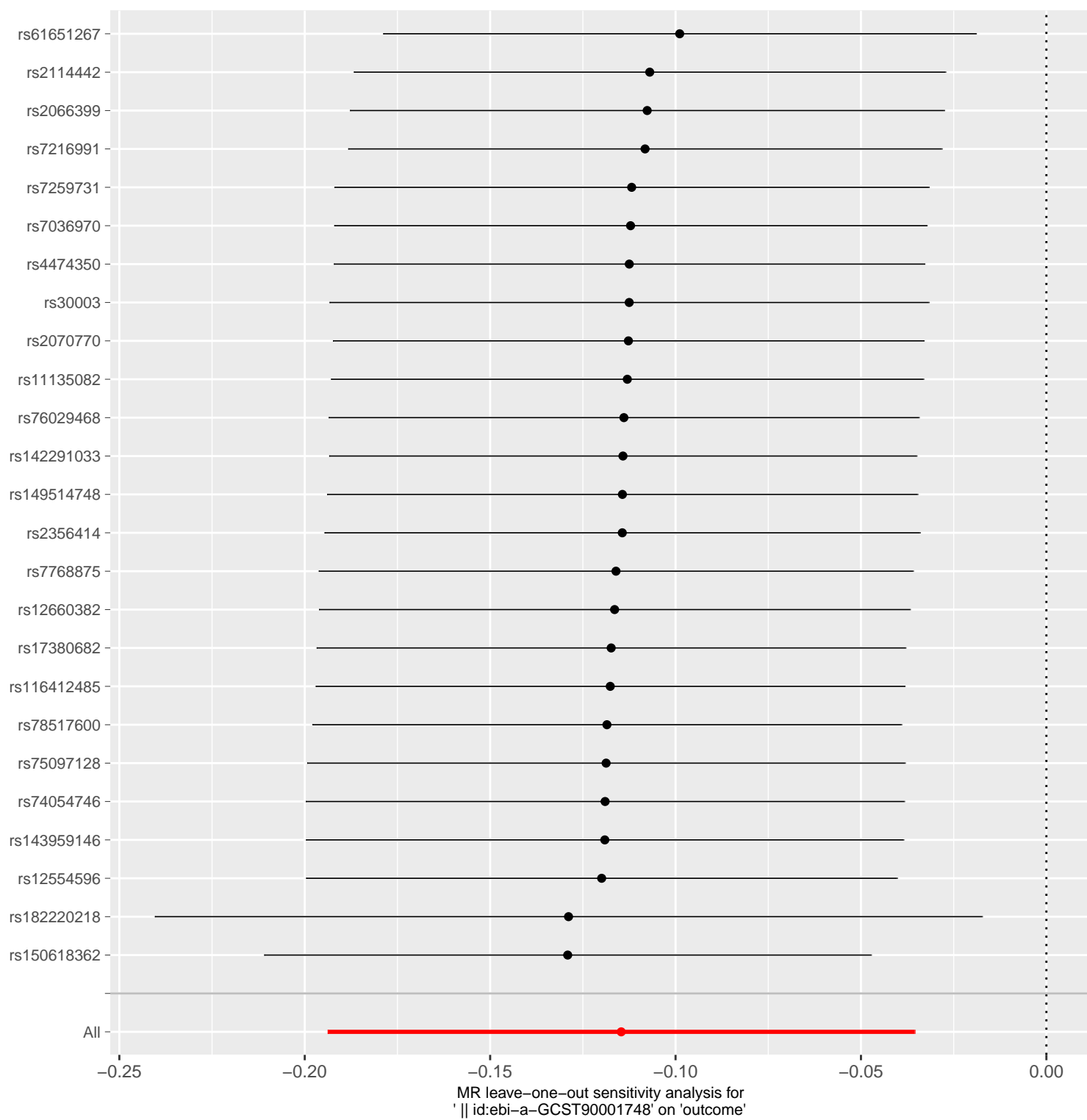

Supplement: Supporting Information — Supplement 1: The STROBE-MR checklist of recommended items to address in reports of Mendelian randomization studies. Supplement 2: The significant pleiotropy or heterogeneity of IVs in the MR analysis using gut microbiota as the exposure and osteonecrosis as the outcome. Supplement 3: The scatterplots and leave-one-out plots in the MR analysis using gut microbiota as the exposure and osteonecrosis as the outcome. Supplement 4: The significant pleiotropy or heterogeneity of IVs in the MR analysis using immune cells as the exposure and osteonecrosis as the outcome. Supplement 5: The scatterplots and leave-one-out plots in the MR analysis using immune cells as the exposure and osteonecrosis as the outcome. Supplement 6: The significant pleiotropy or heterogeneity of IVs in the MR analysis using gut microbiota as the exposure and immune cells as the outcome. Supplement 7: The scatterplots and leave-one-out plots in the MR analysis using gut microbiota as the exposure and immune cells as the outcome. Supplement 8: The results of the MR analysis using osteonecrosis as the exposure and gut microbiota and immune cells as the outcomes. [file 9323113.f1.zip › Supplement 5/ebi-a-GCST90001748/sensitivity-analysis.pdf]

# MR Test

- Inverse variance weighted
- MR Egger
- Simple mode
- Weighted median
- Weighted mode

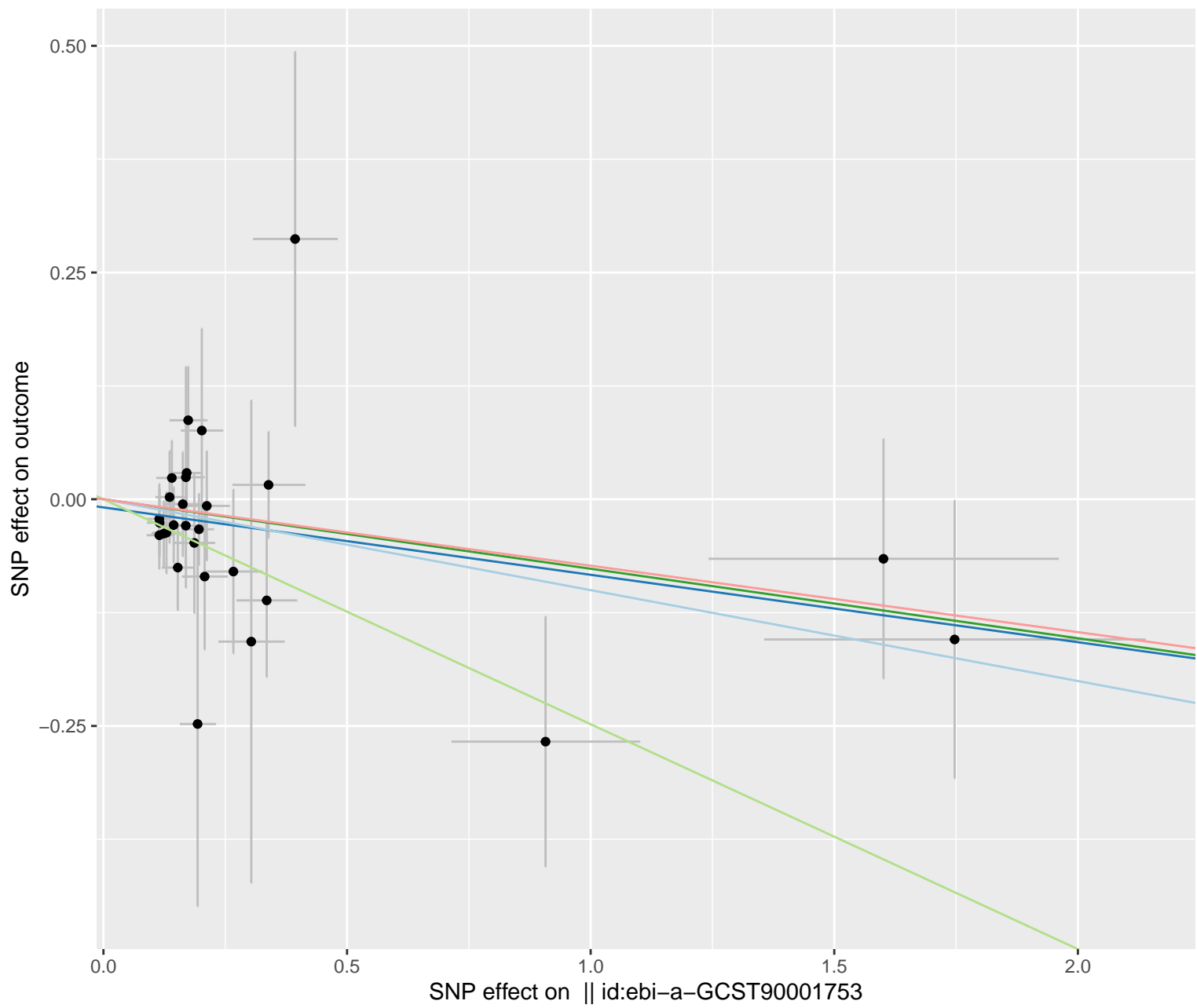

Supplement: Supporting Information — Supplement 1: The STROBE-MR checklist of recommended items to address in reports of Mendelian randomization studies. Supplement 2: The significant pleiotropy or heterogeneity of IVs in the MR analysis using gut microbiota as the exposure and osteonecrosis as the outcome. Supplement 3: The scatterplots and leave-one-out plots in the MR analysis using gut microbiota as the exposure and osteonecrosis as the outcome. Supplement 4: The significant pleiotropy or heterogeneity of IVs in the MR analysis using immune cells as the exposure and osteonecrosis as the outcome. Supplement 5: The scatterplots and leave-one-out plots in the MR analysis using immune cells as the exposure and osteonecrosis as the outcome. Supplement 6: The significant pleiotropy or heterogeneity of IVs in the MR analysis using gut microbiota as the exposure and immune cells as the outcome. Supplement 7: The scatterplots and leave-one-out plots in the MR analysis using gut microbiota as the exposure and immune cells as the outcome. Supplement 8: The results of the MR analysis using osteonecrosis as the exposure and gut microbiota and immune cells as the outcomes. [file 9323113.f1.zip › Supplement 5/ebi-a-GCST90001753/scatter.pdf]

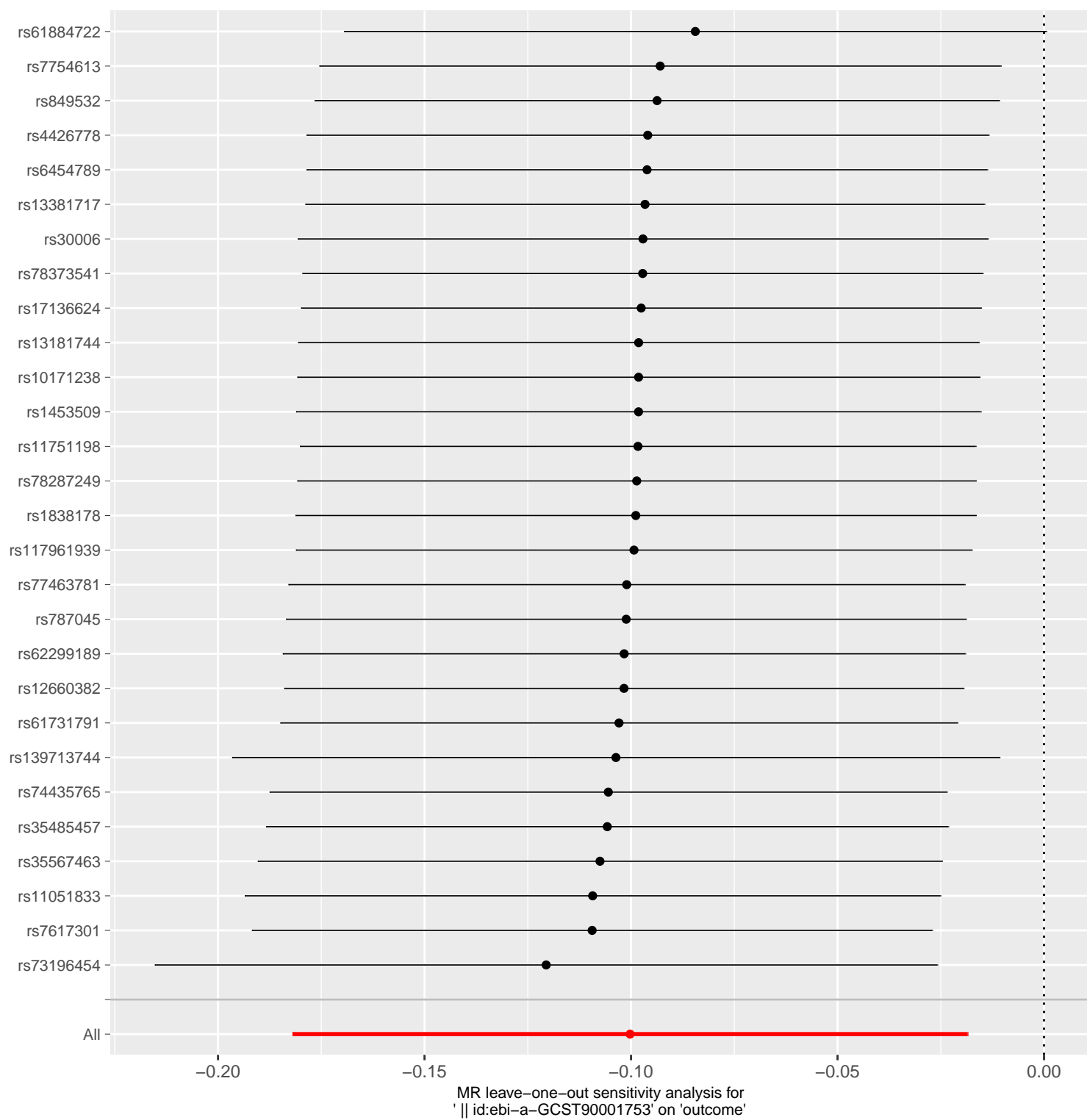

Supplement: Supporting Information — Supplement 1: The STROBE-MR checklist of recommended items to address in reports of Mendelian randomization studies. Supplement 2: The significant pleiotropy or heterogeneity of IVs in the MR analysis using gut microbiota as the exposure and osteonecrosis as the outcome. Supplement 3: The scatterplots and leave-one-out plots in the MR analysis using gut microbiota as the exposure and osteonecrosis as the outcome. Supplement 4: The significant pleiotropy or heterogeneity of IVs in the MR analysis using immune cells as the exposure and osteonecrosis as the outcome. Supplement 5: The scatterplots and leave-one-out plots in the MR analysis using immune cells as the exposure and osteonecrosis as the outcome. Supplement 6: The significant pleiotropy or heterogeneity of IVs in the MR analysis using gut microbiota as the exposure and immune cells as the outcome. Supplement 7: The scatterplots and leave-one-out plots in the MR analysis using gut microbiota as the exposure and immune cells as the outcome. Supplement 8: The results of the MR analysis using osteonecrosis as the exposure and gut microbiota and immune cells as the outcomes. [file 9323113.f1.zip › Supplement 5/ebi-a-GCST90001753/sensitivity-analysis.pdf]

# MR Test

- Inverse variance weighted
- MR Egger
- Simple mode
- Weighted median
- Weighted mode

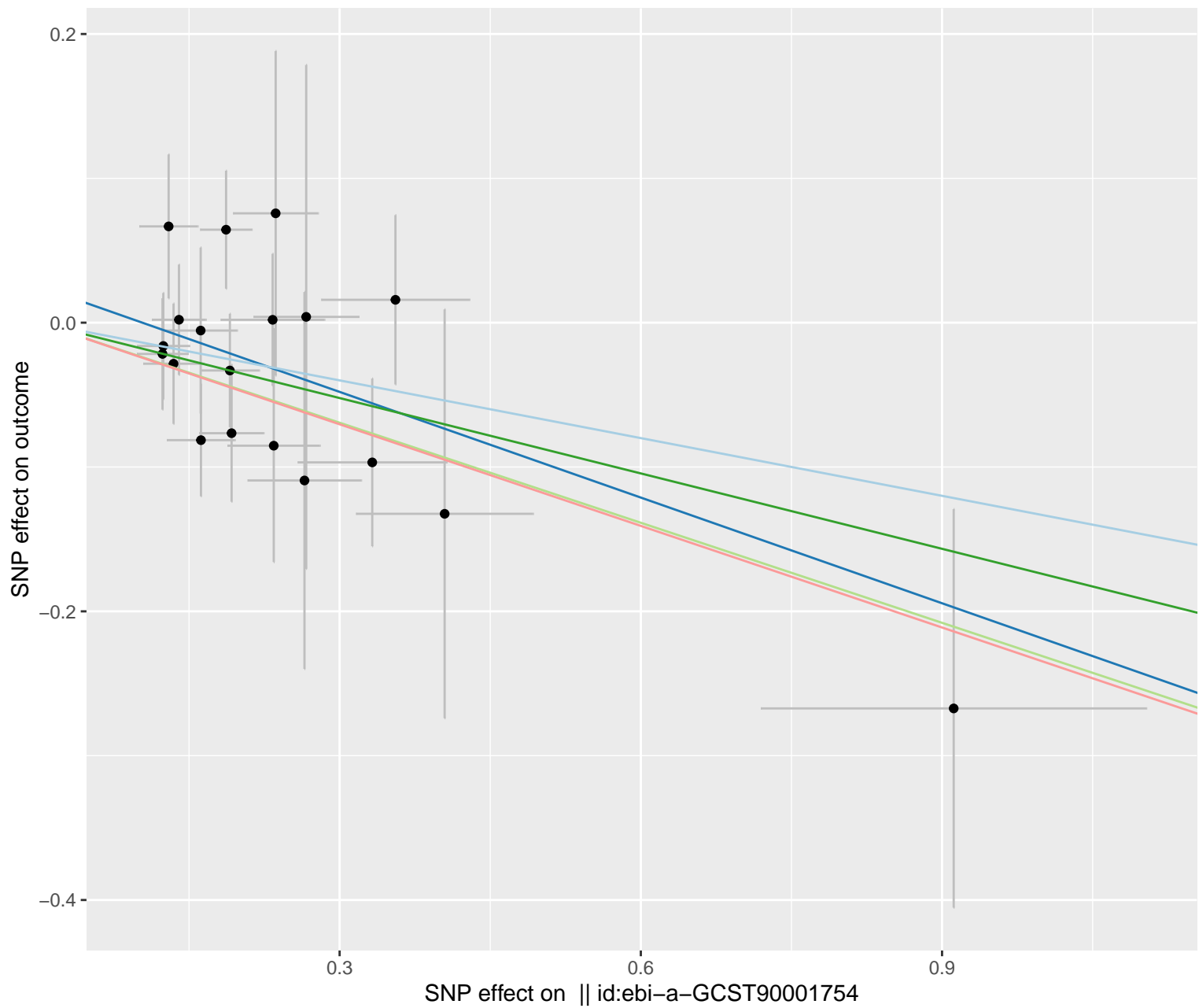

Supplement: Supporting Information — Supplement 1: The STROBE-MR checklist of recommended items to address in reports of Mendelian randomization studies. Supplement 2: The significant pleiotropy or heterogeneity of IVs in the MR analysis using gut microbiota as the exposure and osteonecrosis as the outcome. Supplement 3: The scatterplots and leave-one-out plots in the MR analysis using gut microbiota as the exposure and osteonecrosis as the outcome. Supplement 4: The significant pleiotropy or heterogeneity of IVs in the MR analysis using immune cells as the exposure and osteonecrosis as the outcome. Supplement 5: The scatterplots and leave-one-out plots in the MR analysis using immune cells as the exposure and osteonecrosis as the outcome. Supplement 6: The significant pleiotropy or heterogeneity of IVs in the MR analysis using gut microbiota as the exposure and immune cells as the outcome. Supplement 7: The scatterplots and leave-one-out plots in the MR analysis using gut microbiota as the exposure and immune cells as the outcome. Supplement 8: The results of the MR analysis using osteonecrosis as the exposure and gut microbiota and immune cells as the outcomes. [file 9323113.f1.zip › Supplement 5/ebi-a-GCST90001754/scatter.pdf]

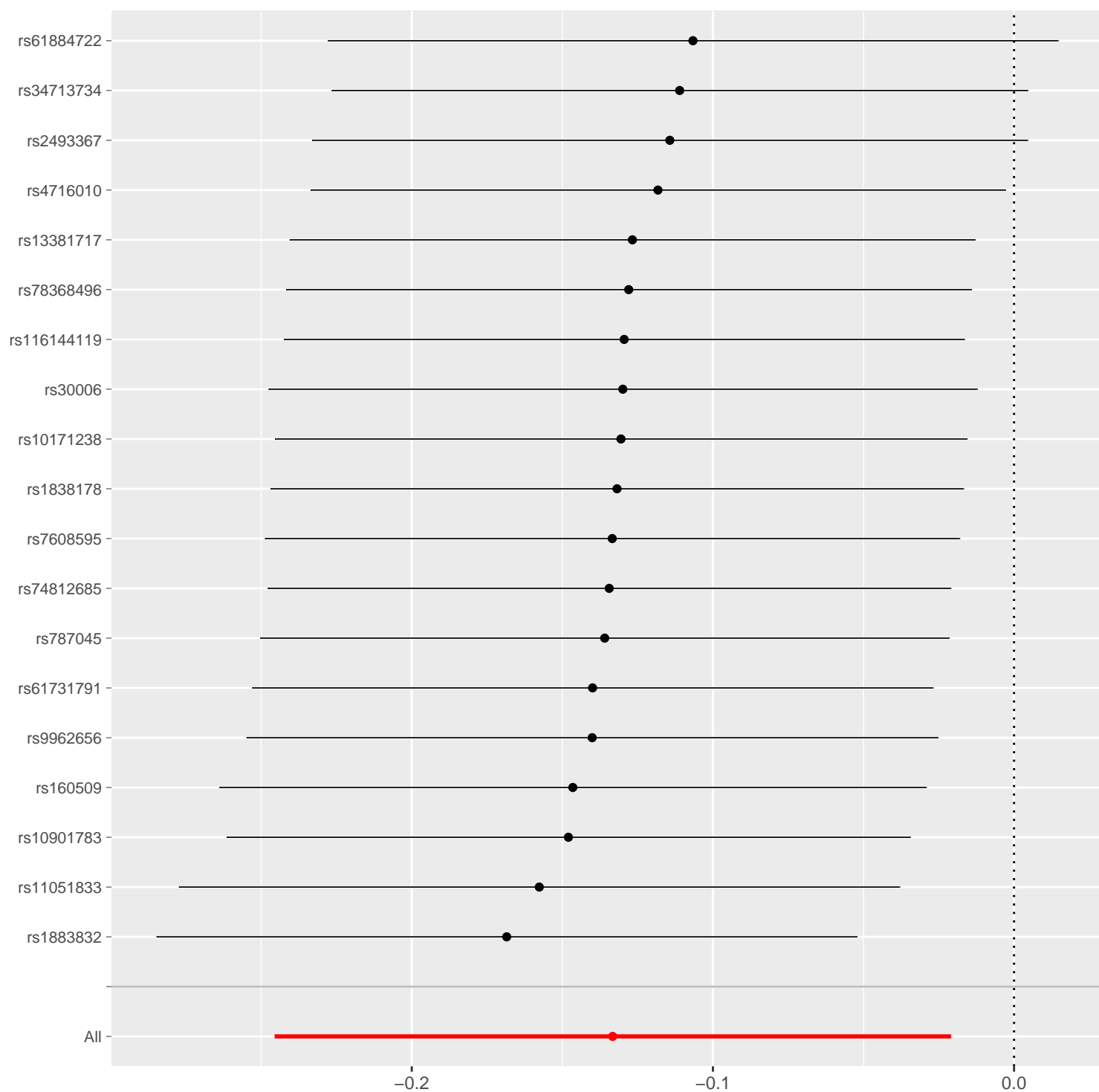

Supplement: Supporting Information — Supplement 1: The STROBE-MR checklist of recommended items to address in reports of Mendelian randomization studies. Supplement 2: The significant pleiotropy or heterogeneity of IVs in the MR analysis using gut microbiota as the exposure and osteonecrosis as the outcome. Supplement 3: The scatterplots and leave-one-out plots in the MR analysis using gut microbiota as the exposure and osteonecrosis as the outcome. Supplement 4: The significant pleiotropy or heterogeneity of IVs in the MR analysis using immune cells as the exposure and osteonecrosis as the outcome. Supplement 5: The scatterplots and leave-one-out plots in the MR analysis using immune cells as the exposure and osteonecrosis as the outcome. Supplement 6: The significant pleiotropy or heterogeneity of IVs in the MR analysis using gut microbiota as the exposure and immune cells as the outcome. Supplement 7: The scatterplots and leave-one-out plots in the MR analysis using gut microbiota as the exposure and immune cells as the outcome. Supplement 8: The results of the MR analysis using osteonecrosis as the exposure and gut microbiota and immune cells as the outcomes. [file 9323113.f1.zip › Supplement 5/ebi-a-GCST90001754/sensitivity-analysis.pdf]

# MR Test

- Inverse variance weighted
- MR Egger
- Simple mode
- Weighted median
- Weighted mode

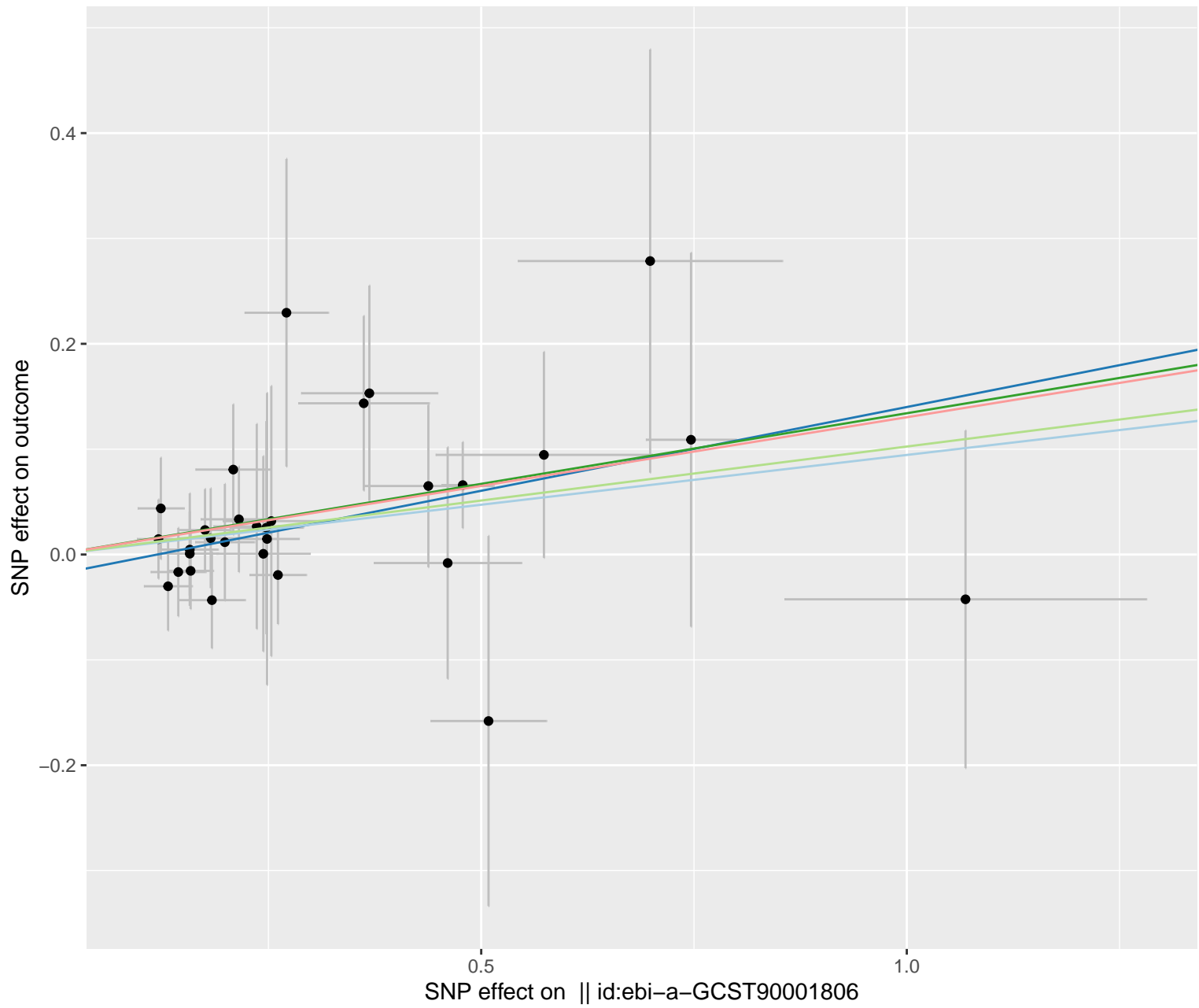

Supplement: Supporting Information — Supplement 1: The STROBE-MR checklist of recommended items to address in reports of Mendelian randomization studies. Supplement 2: The significant pleiotropy or heterogeneity of IVs in the MR analysis using gut microbiota as the exposure and osteonecrosis as the outcome. Supplement 3: The scatterplots and leave-one-out plots in the MR analysis using gut microbiota as the exposure and osteonecrosis as the outcome. Supplement 4: The significant pleiotropy or heterogeneity of IVs in the MR analysis using immune cells as the exposure and osteonecrosis as the outcome. Supplement 5: The scatterplots and leave-one-out plots in the MR analysis using immune cells as the exposure and osteonecrosis as the outcome. Supplement 6: The significant pleiotropy or heterogeneity of IVs in the MR analysis using gut microbiota as the exposure and immune cells as the outcome. Supplement 7: The scatterplots and leave-one-out plots in the MR analysis using gut microbiota as the exposure and immune cells as the outcome. Supplement 8: The results of the MR analysis using osteonecrosis as the exposure and gut microbiota and immune cells as the outcomes. [file 9323113.f1.zip › Supplement 5/ebi-a-GCST90001806/scatter.pdf]

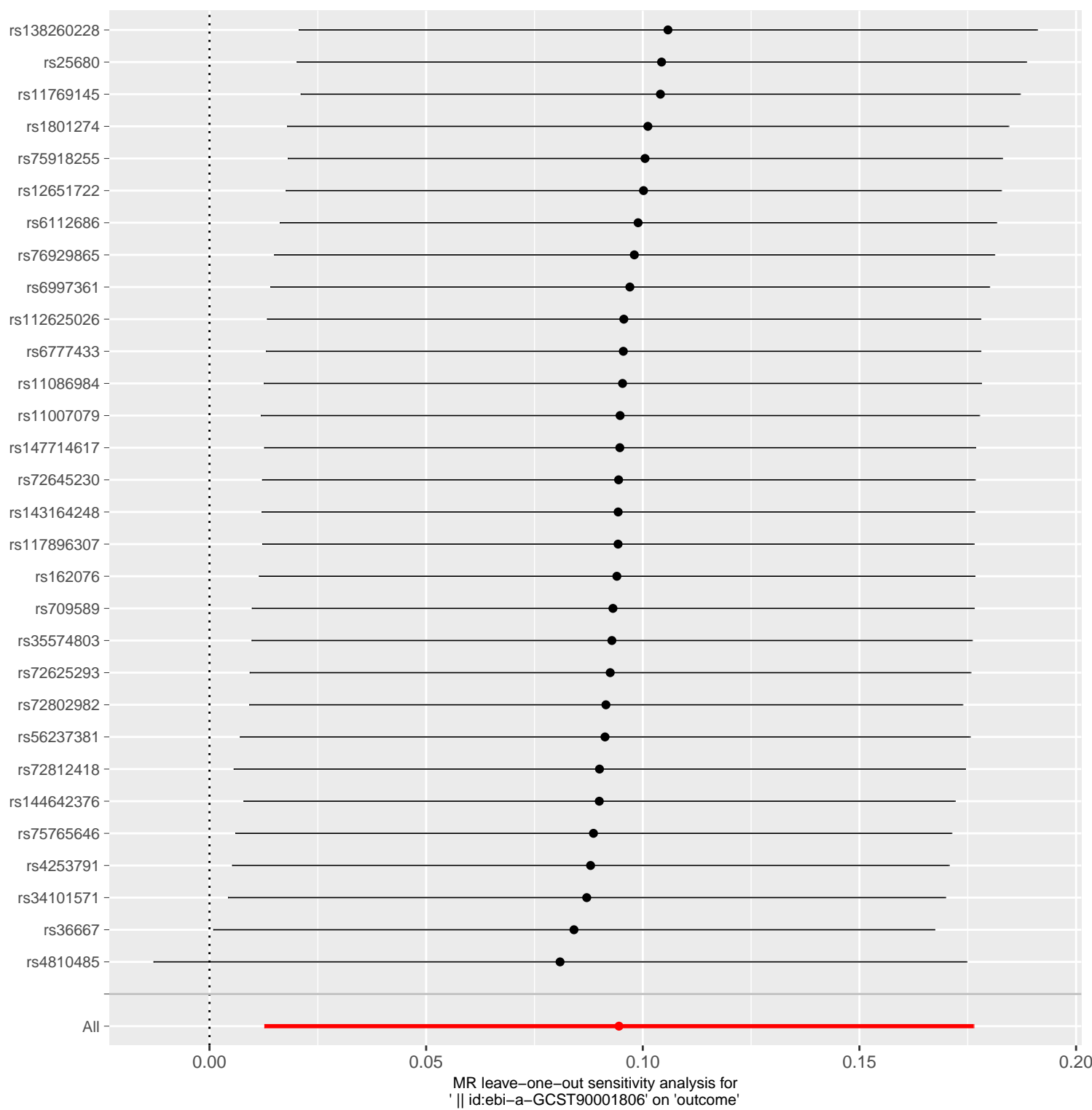

Supplement: Supporting Information — Supplement 1: The STROBE-MR checklist of recommended items to address in reports of Mendelian randomization studies. Supplement 2: The significant pleiotropy or heterogeneity of IVs in the MR analysis using gut microbiota as the exposure and osteonecrosis as the outcome. Supplement 3: The scatterplots and leave-one-out plots in the MR analysis using gut microbiota as the exposure and osteonecrosis as the outcome. Supplement 4: The significant pleiotropy or heterogeneity of IVs in the MR analysis using immune cells as the exposure and osteonecrosis as the outcome. Supplement 5: The scatterplots and leave-one-out plots in the MR analysis using immune cells as the exposure and osteonecrosis as the outcome. Supplement 6: The significant pleiotropy or heterogeneity of IVs in the MR analysis using gut microbiota as the exposure and immune cells as the outcome. Supplement 7: The scatterplots and leave-one-out plots in the MR analysis using gut microbiota as the exposure and immune cells as the outcome. Supplement 8: The results of the MR analysis using osteonecrosis as the exposure and gut microbiota and immune cells as the outcomes. [file 9323113.f1.zip › Supplement 5/ebi-a-GCST90001806/sensitivity-analysis.pdf]

# MR Test

- Inverse variance weighted
- MR Egger
- Simple mode
- Weighted median
- Weighted mode

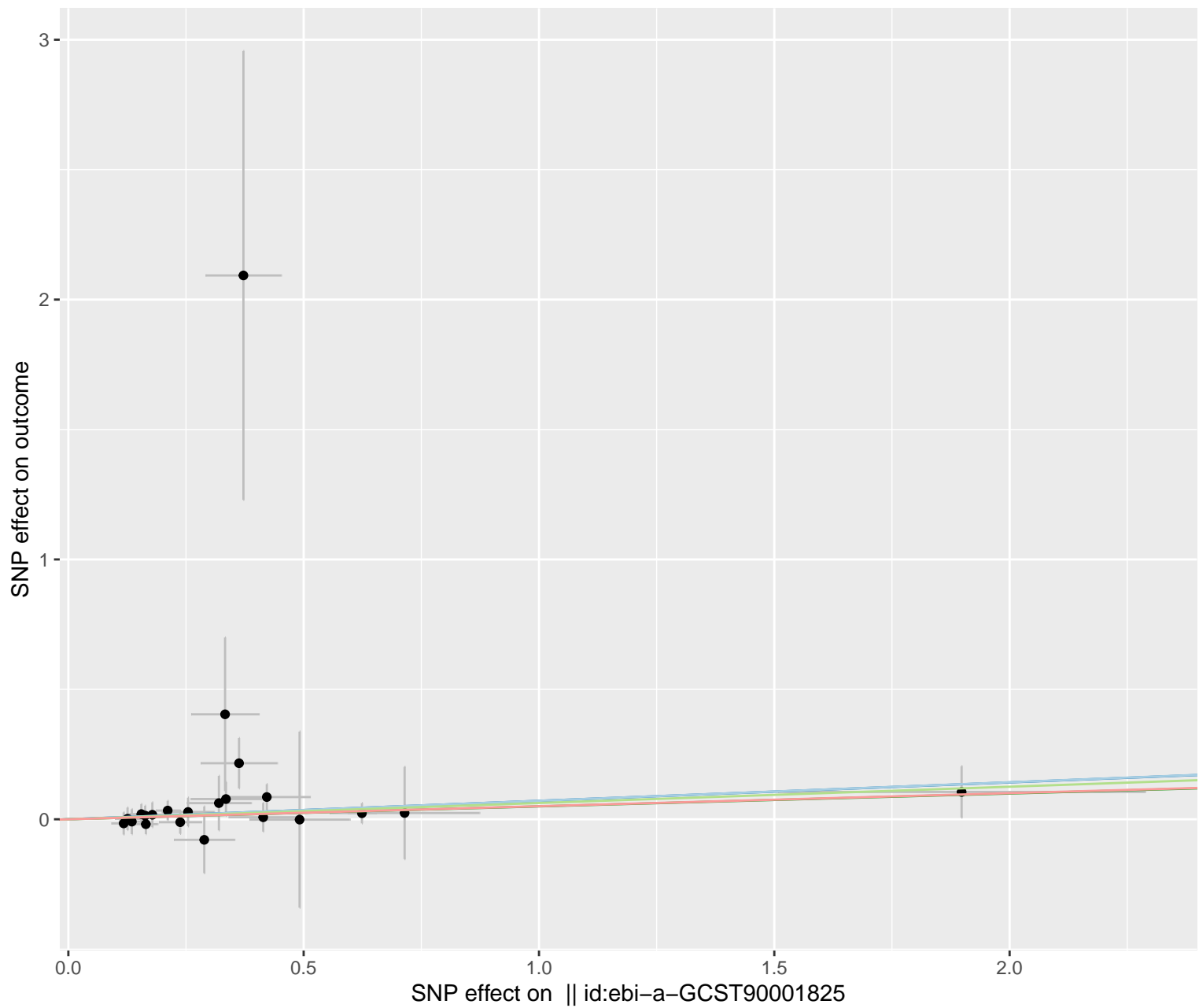

Supplement: Supporting Information — Supplement 1: The STROBE-MR checklist of recommended items to address in reports of Mendelian randomization studies. Supplement 2: The significant pleiotropy or heterogeneity of IVs in the MR analysis using gut microbiota as the exposure and osteonecrosis as the outcome. Supplement 3: The scatterplots and leave-one-out plots in the MR analysis using gut microbiota as the exposure and osteonecrosis as the outcome. Supplement 4: The significant pleiotropy or heterogeneity of IVs in the MR analysis using immune cells as the exposure and osteonecrosis as the outcome. Supplement 5: The scatterplots and leave-one-out plots in the MR analysis using immune cells as the exposure and osteonecrosis as the outcome. Supplement 6: The significant pleiotropy or heterogeneity of IVs in the MR analysis using gut microbiota as the exposure and immune cells as the outcome. Supplement 7: The scatterplots and leave-one-out plots in the MR analysis using gut microbiota as the exposure and immune cells as the outcome. Supplement 8: The results of the MR analysis using osteonecrosis as the exposure and gut microbiota and immune cells as the outcomes. [file 9323113.f1.zip › Supplement 5/ebi-a-GCST90001825/scatter.pdf]

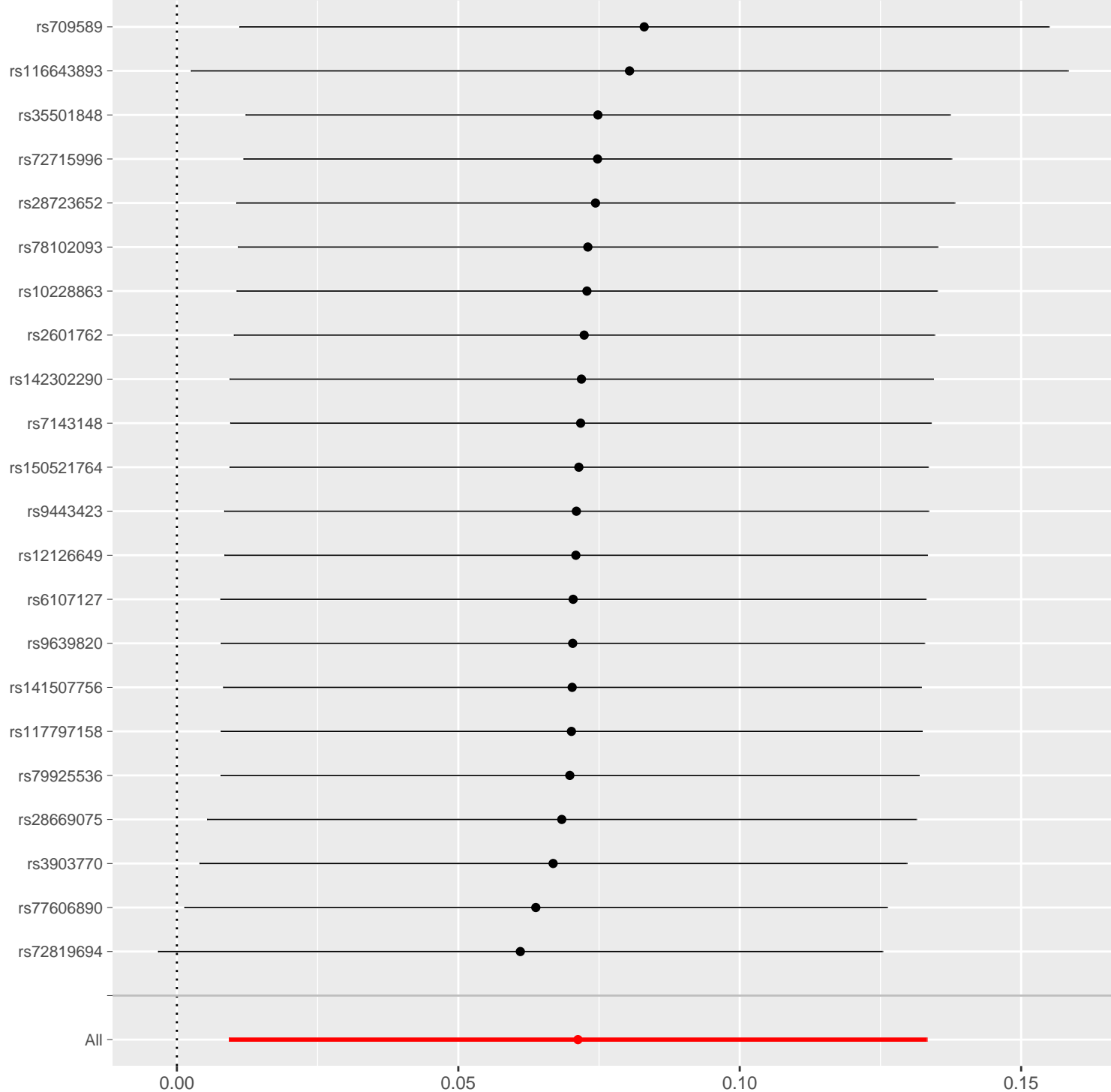

Supplement: Supporting Information — Supplement 1: The STROBE-MR checklist of recommended items to address in reports of Mendelian randomization studies. Supplement 2: The significant pleiotropy or heterogeneity of IVs in the MR analysis using gut microbiota as the exposure and osteonecrosis as the outcome. Supplement 3: The scatterplots and leave-one-out plots in the MR analysis using gut microbiota as the exposure and osteonecrosis as the outcome. Supplement 4: The significant pleiotropy or heterogeneity of IVs in the MR analysis using immune cells as the exposure and osteonecrosis as the outcome. Supplement 5: The scatterplots and leave-one-out plots in the MR analysis using immune cells as the exposure and osteonecrosis as the outcome. Supplement 6: The significant pleiotropy or heterogeneity of IVs in the MR analysis using gut microbiota as the exposure and immune cells as the outcome. Supplement 7: The scatterplots and leave-one-out plots in the MR analysis using gut microbiota as the exposure and immune cells as the outcome. Supplement 8: The results of the MR analysis using osteonecrosis as the exposure and gut microbiota and immune cells as the outcomes. [file 9323113.f1.zip › Supplement 5/ebi-a-GCST90001825/sensitivity-analysis.pdf]

# MR Test

- Inverse variance weighted
- MR Egger
- Simple mode
- Weighted median
- Weighted mode

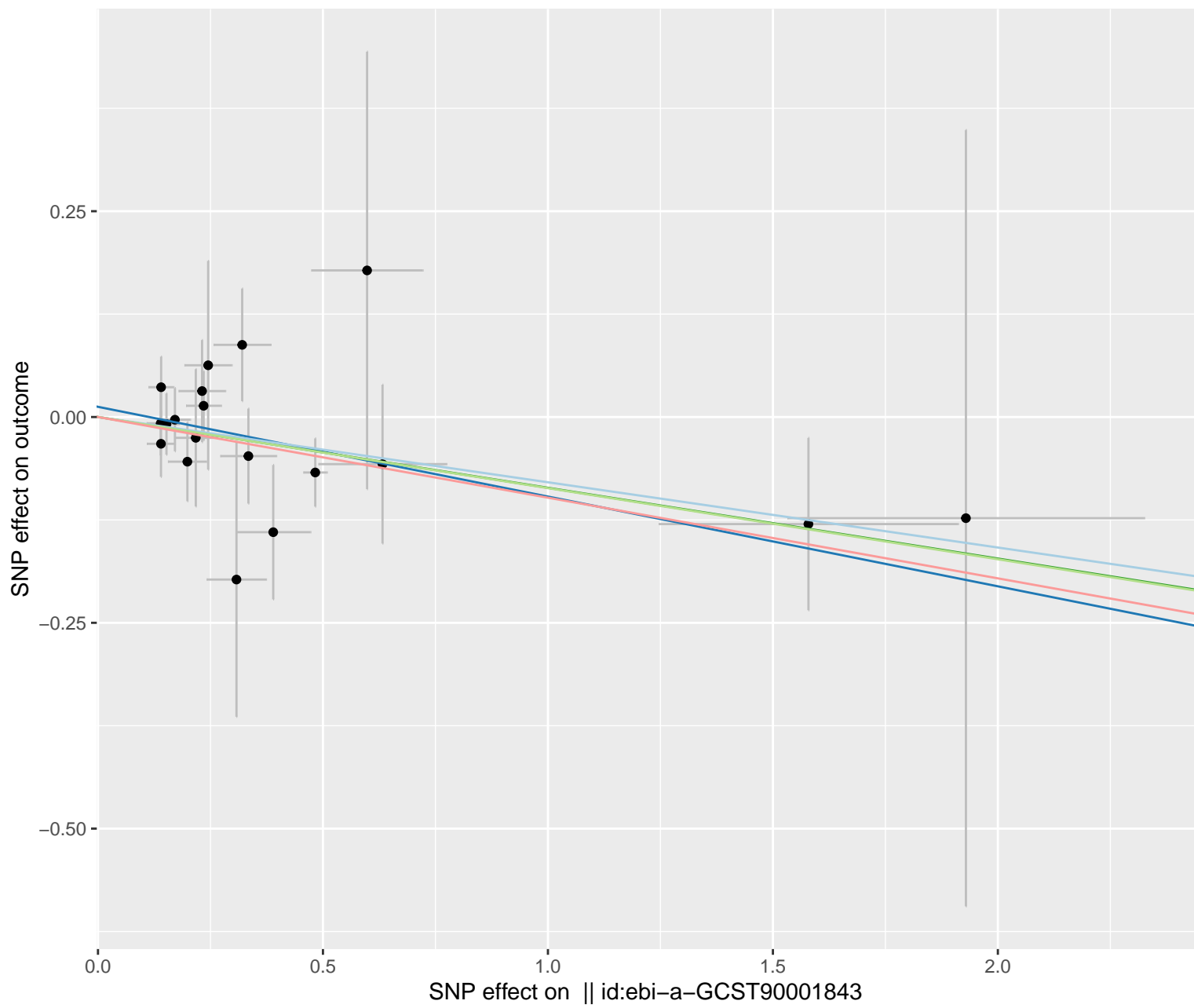

Supplement: Supporting Information — Supplement 1: The STROBE-MR checklist of recommended items to address in reports of Mendelian randomization studies. Supplement 2: The significant pleiotropy or heterogeneity of IVs in the MR analysis using gut microbiota as the exposure and osteonecrosis as the outcome. Supplement 3: The scatterplots and leave-one-out plots in the MR analysis using gut microbiota as the exposure and osteonecrosis as the outcome. Supplement 4: The significant pleiotropy or heterogeneity of IVs in the MR analysis using immune cells as the exposure and osteonecrosis as the outcome. Supplement 5: The scatterplots and leave-one-out plots in the MR analysis using immune cells as the exposure and osteonecrosis as the outcome. Supplement 6: The significant pleiotropy or heterogeneity of IVs in the MR analysis using gut microbiota as the exposure and immune cells as the outcome. Supplement 7: The scatterplots and leave-one-out plots in the MR analysis using gut microbiota as the exposure and immune cells as the outcome. Supplement 8: The results of the MR analysis using osteonecrosis as the exposure and gut microbiota and immune cells as the outcomes. [file 9323113.f1.zip › Supplement 5/ebi-a-GCST90001843/scatter.pdf]

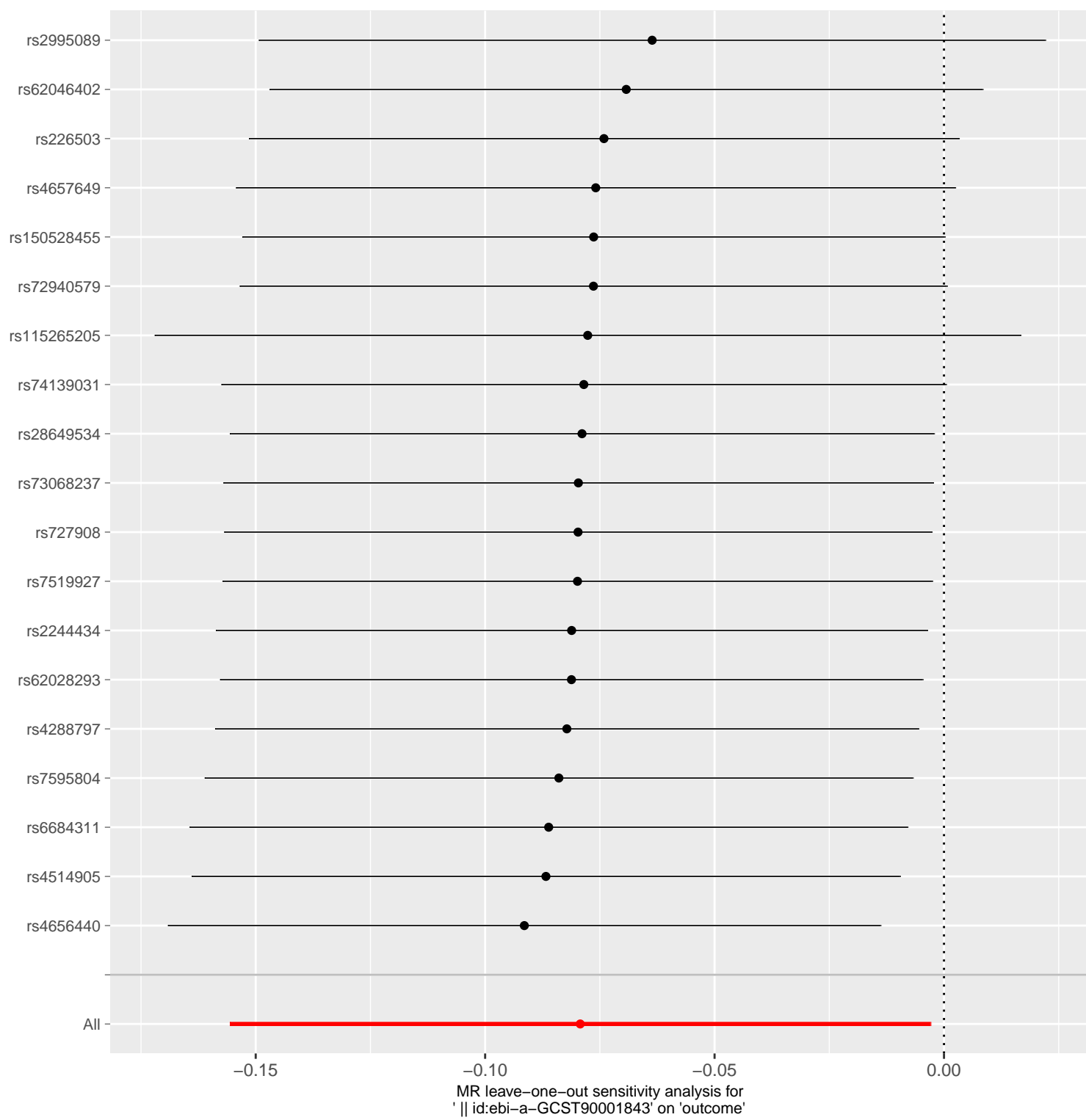

Supplement: Supporting Information — Supplement 1: The STROBE-MR checklist of recommended items to address in reports of Mendelian randomization studies. Supplement 2: The significant pleiotropy or heterogeneity of IVs in the MR analysis using gut microbiota as the exposure and osteonecrosis as the outcome. Supplement 3: The scatterplots and leave-one-out plots in the MR analysis using gut microbiota as the exposure and osteonecrosis as the outcome. Supplement 4: The significant pleiotropy or heterogeneity of IVs in the MR analysis using immune cells as the exposure and osteonecrosis as the outcome. Supplement 5: The scatterplots and leave-one-out plots in the MR analysis using immune cells as the exposure and osteonecrosis as the outcome. Supplement 6: The significant pleiotropy or heterogeneity of IVs in the MR analysis using gut microbiota as the exposure and immune cells as the outcome. Supplement 7: The scatterplots and leave-one-out plots in the MR analysis using gut microbiota as the exposure and immune cells as the outcome. Supplement 8: The results of the MR analysis using osteonecrosis as the exposure and gut microbiota and immune cells as the outcomes. [file 9323113.f1.zip › Supplement 5/ebi-a-GCST90001843/sensitivity-analysis.pdf]

# MR Test

- Inverse variance weighted
- MR Egger
- Simple mode
- Weighted median
- Weighted mode

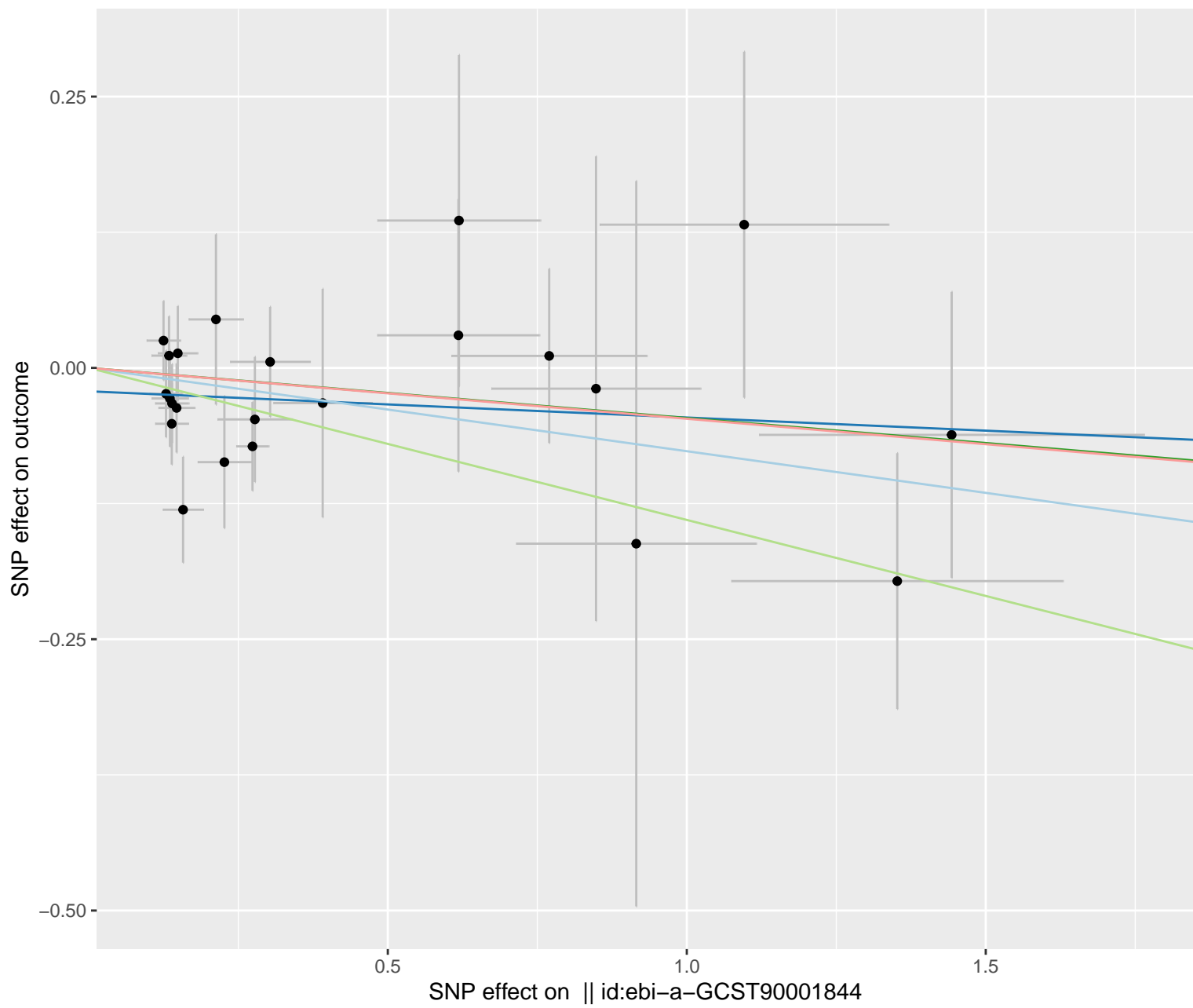

Supplement: Supporting Information — Supplement 1: The STROBE-MR checklist of recommended items to address in reports of Mendelian randomization studies. Supplement 2: The significant pleiotropy or heterogeneity of IVs in the MR analysis using gut microbiota as the exposure and osteonecrosis as the outcome. Supplement 3: The scatterplots and leave-one-out plots in the MR analysis using gut microbiota as the exposure and osteonecrosis as the outcome. Supplement 4: The significant pleiotropy or heterogeneity of IVs in the MR analysis using immune cells as the exposure and osteonecrosis as the outcome. Supplement 5: The scatterplots and leave-one-out plots in the MR analysis using immune cells as the exposure and osteonecrosis as the outcome. Supplement 6: The significant pleiotropy or heterogeneity of IVs in the MR analysis using gut microbiota as the exposure and immune cells as the outcome. Supplement 7: The scatterplots and leave-one-out plots in the MR analysis using gut microbiota as the exposure and immune cells as the outcome. Supplement 8: The results of the MR analysis using osteonecrosis as the exposure and gut microbiota and immune cells as the outcomes. [file 9323113.f1.zip › Supplement 5/ebi-a-GCST90001844/scatter.pdf]

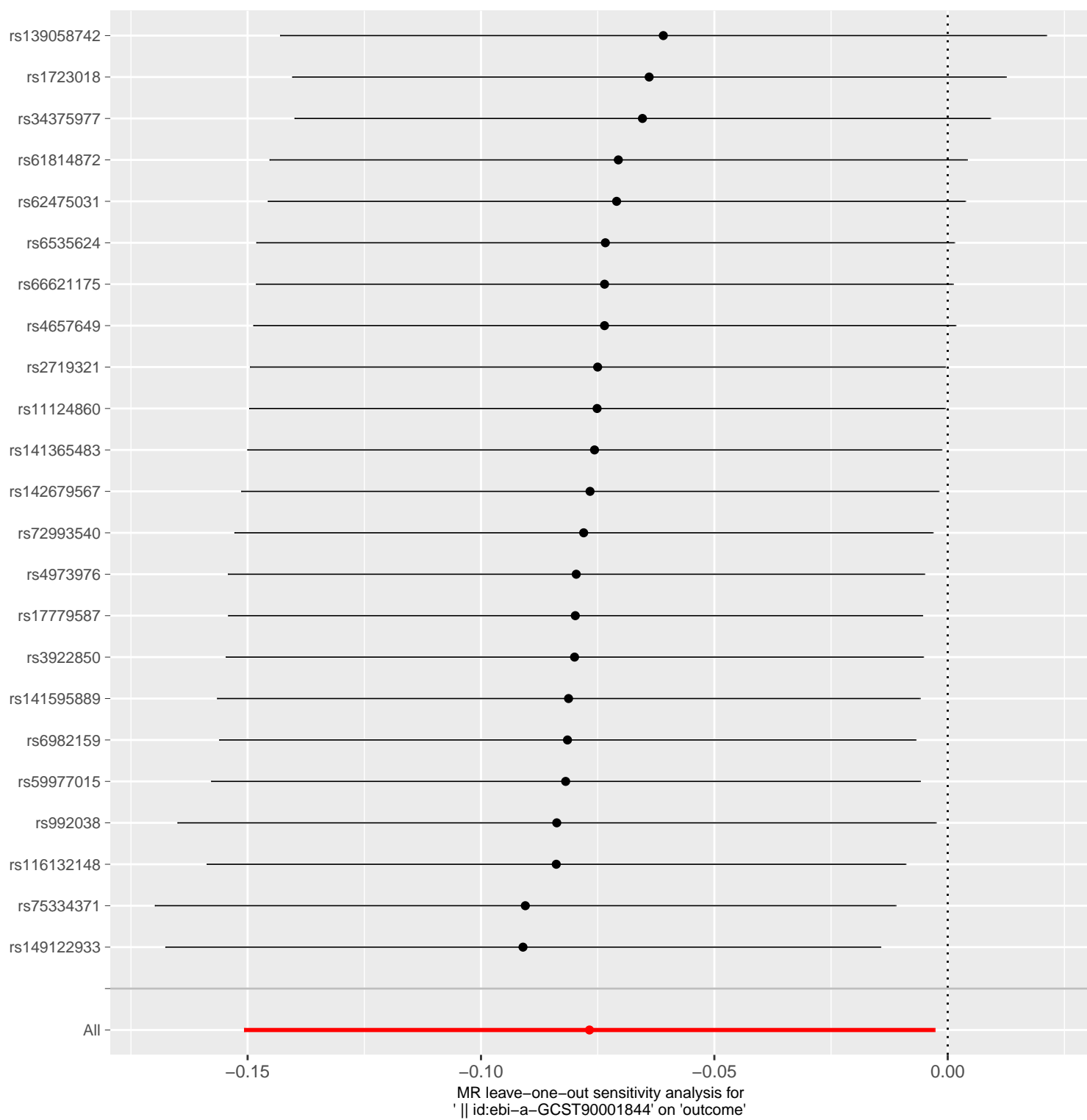

Supplement: Supporting Information — Supplement 1: The STROBE-MR checklist of recommended items to address in reports of Mendelian randomization studies. Supplement 2: The significant pleiotropy or heterogeneity of IVs in the MR analysis using gut microbiota as the exposure and osteonecrosis as the outcome. Supplement 3: The scatterplots and leave-one-out plots in the MR analysis using gut microbiota as the exposure and osteonecrosis as the outcome. Supplement 4: The significant pleiotropy or heterogeneity of IVs in the MR analysis using immune cells as the exposure and osteonecrosis as the outcome. Supplement 5: The scatterplots and leave-one-out plots in the MR analysis using immune cells as the exposure and osteonecrosis as the outcome. Supplement 6: The significant pleiotropy or heterogeneity of IVs in the MR analysis using gut microbiota as the exposure and immune cells as the outcome. Supplement 7: The scatterplots and leave-one-out plots in the MR analysis using gut microbiota as the exposure and immune cells as the outcome. Supplement 8: The results of the MR analysis using osteonecrosis as the exposure and gut microbiota and immune cells as the outcomes. [file 9323113.f1.zip › Supplement 5/ebi-a-GCST90001844/sensitivity-analysis.pdf]

# MR Test

- Inverse variance weighted
- MR Egger
- Simple mode
- Weighted median
- Weighted mode

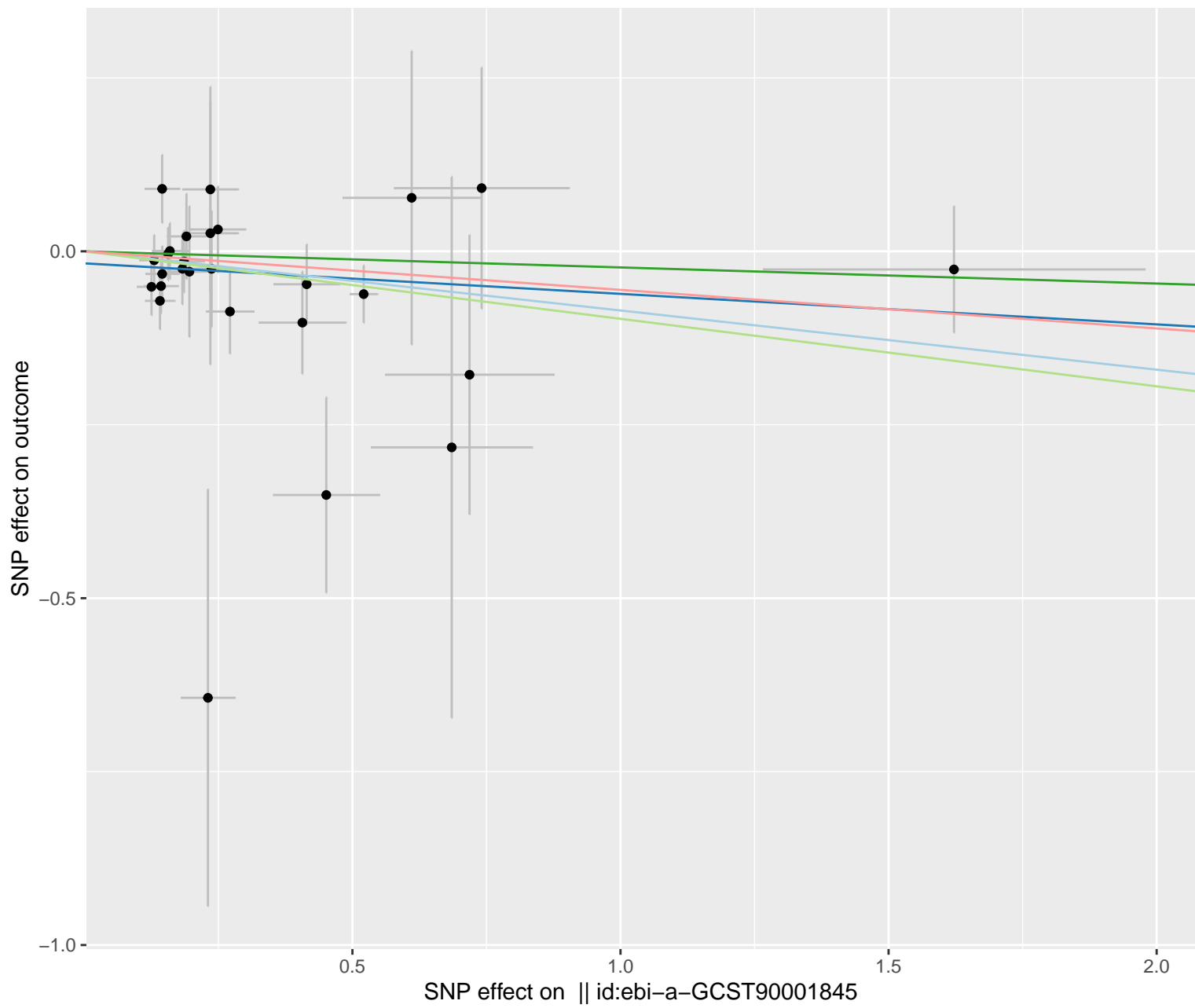

Supplement: Supporting Information — Supplement 1: The STROBE-MR checklist of recommended items to address in reports of Mendelian randomization studies. Supplement 2: The significant pleiotropy or heterogeneity of IVs in the MR analysis using gut microbiota as the exposure and osteonecrosis as the outcome. Supplement 3: The scatterplots and leave-one-out plots in the MR analysis using gut microbiota as the exposure and osteonecrosis as the outcome. Supplement 4: The significant pleiotropy or heterogeneity of IVs in the MR analysis using immune cells as the exposure and osteonecrosis as the outcome. Supplement 5: The scatterplots and leave-one-out plots in the MR analysis using immune cells as the exposure and osteonecrosis as the outcome. Supplement 6: The significant pleiotropy or heterogeneity of IVs in the MR analysis using gut microbiota as the exposure and immune cells as the outcome. Supplement 7: The scatterplots and leave-one-out plots in the MR analysis using gut microbiota as the exposure and immune cells as the outcome. Supplement 8: The results of the MR analysis using osteonecrosis as the exposure and gut microbiota and immune cells as the outcomes. [file 9323113.f1.zip › Supplement 5/ebi-a-GCST90001845/scatter.pdf]

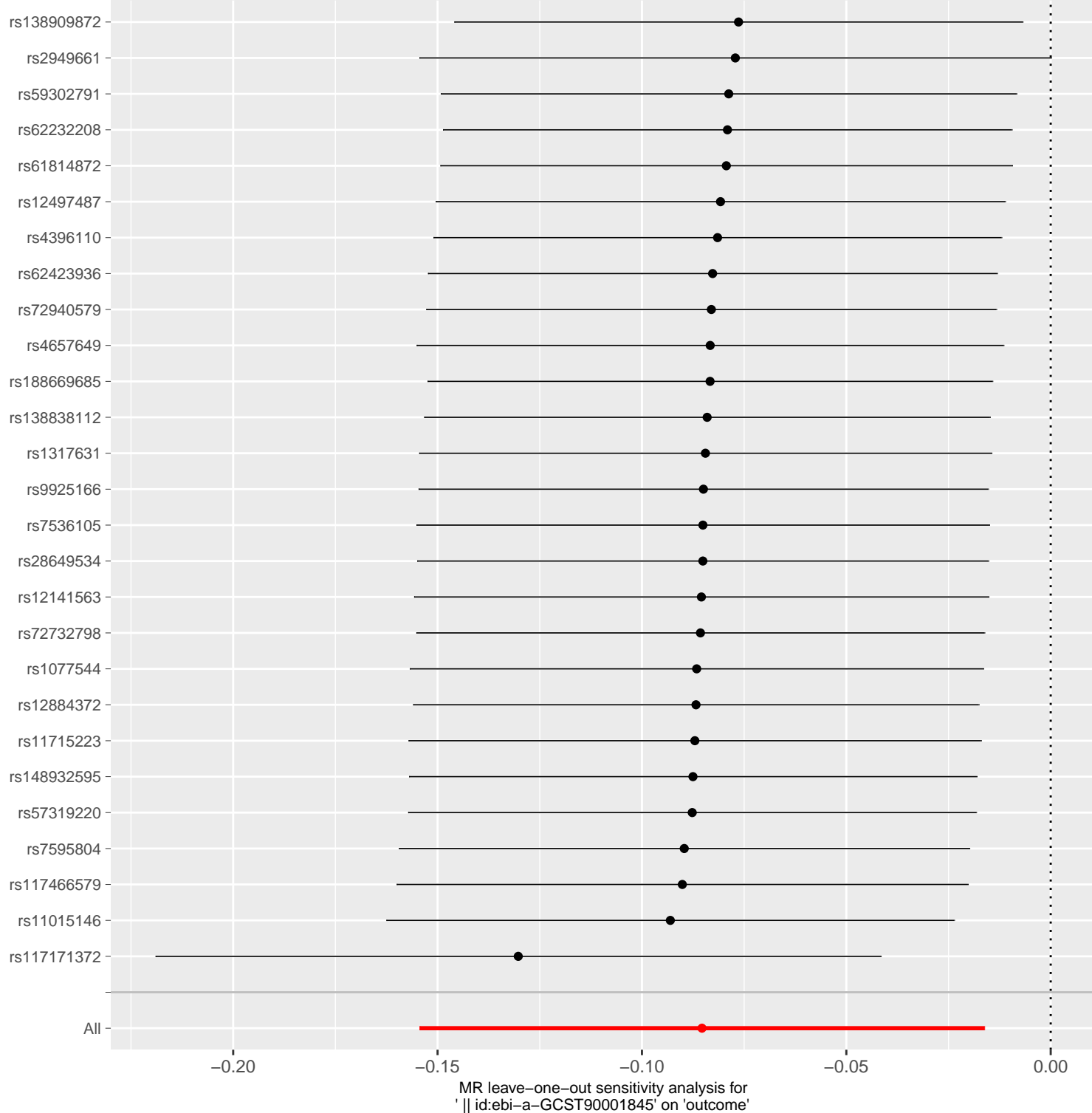

Supplement: Supporting Information — Supplement 1: The STROBE-MR checklist of recommended items to address in reports of Mendelian randomization studies. Supplement 2: The significant pleiotropy or heterogeneity of IVs in the MR analysis using gut microbiota as the exposure and osteonecrosis as the outcome. Supplement 3: The scatterplots and leave-one-out plots in the MR analysis using gut microbiota as the exposure and osteonecrosis as the outcome. Supplement 4: The significant pleiotropy or heterogeneity of IVs in the MR analysis using immune cells as the exposure and osteonecrosis as the outcome. Supplement 5: The scatterplots and leave-one-out plots in the MR analysis using immune cells as the exposure and osteonecrosis as the outcome. Supplement 6: The significant pleiotropy or heterogeneity of IVs in the MR analysis using gut microbiota as the exposure and immune cells as the outcome. Supplement 7: The scatterplots and leave-one-out plots in the MR analysis using gut microbiota as the exposure and immune cells as the outcome. Supplement 8: The results of the MR analysis using osteonecrosis as the exposure and gut microbiota and immune cells as the outcomes. [file 9323113.f1.zip › Supplement 5/ebi-a-GCST90001845/sensitivity-analysis.pdf]

# MR Test

- Inverse variance weighted
- MR Egger
- Simple mode
- Weighted median
- Weighted mode

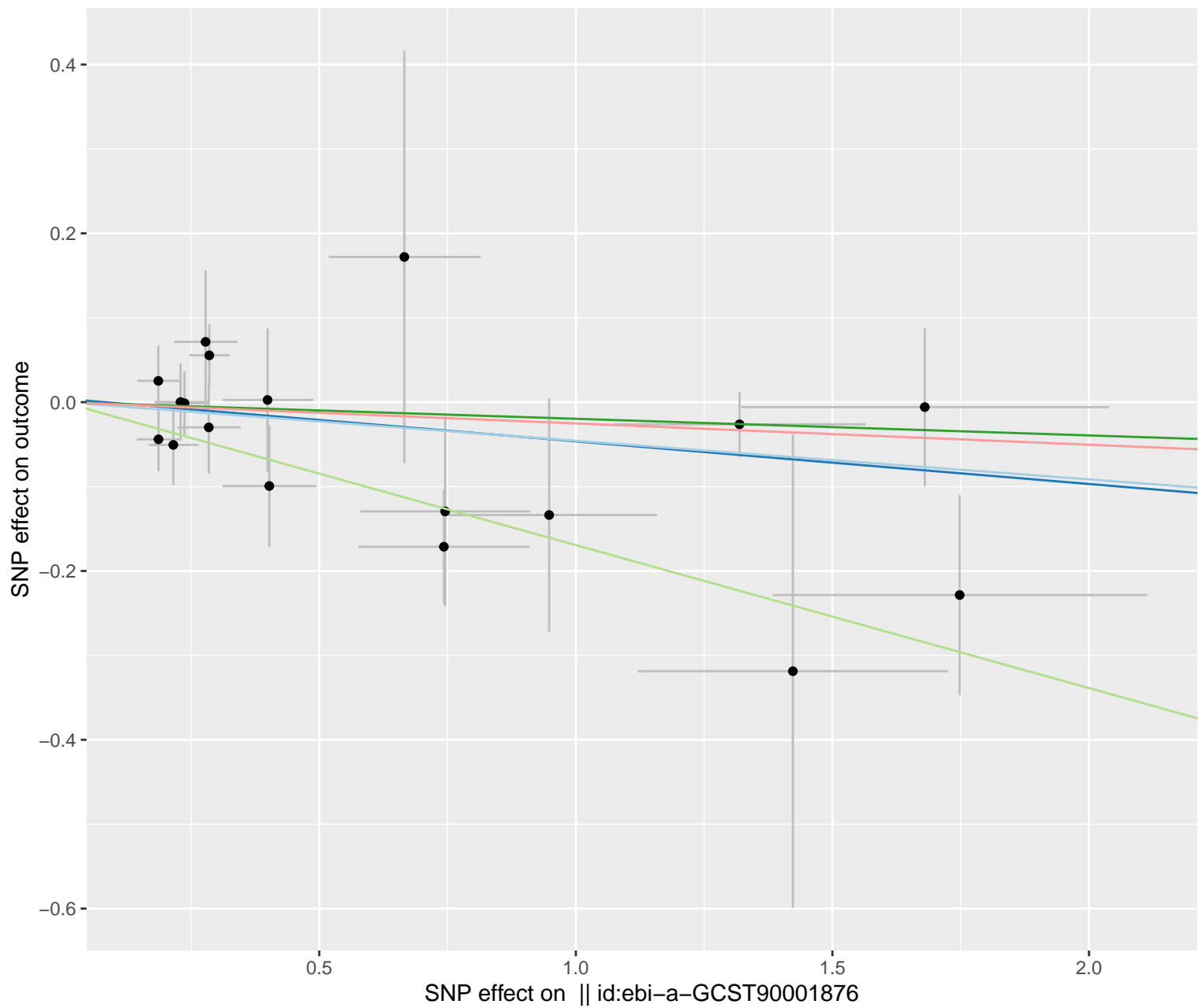

Supplement: Supporting Information — Supplement 1: The STROBE-MR checklist of recommended items to address in reports of Mendelian randomization studies. Supplement 2: The significant pleiotropy or heterogeneity of IVs in the MR analysis using gut microbiota as the exposure and osteonecrosis as the outcome. Supplement 3: The scatterplots and leave-one-out plots in the MR analysis using gut microbiota as the exposure and osteonecrosis as the outcome. Supplement 4: The significant pleiotropy or heterogeneity of IVs in the MR analysis using immune cells as the exposure and osteonecrosis as the outcome. Supplement 5: The scatterplots and leave-one-out plots in the MR analysis using immune cells as the exposure and osteonecrosis as the outcome. Supplement 6: The significant pleiotropy or heterogeneity of IVs in the MR analysis using gut microbiota as the exposure and immune cells as the outcome. Supplement 7: The scatterplots and leave-one-out plots in the MR analysis using gut microbiota as the exposure and immune cells as the outcome. Supplement 8: The results of the MR analysis using osteonecrosis as the exposure and gut microbiota and immune cells as the outcomes. [file 9323113.f1.zip › Supplement 5/ebi-a-GCST90001876/scatter.pdf]

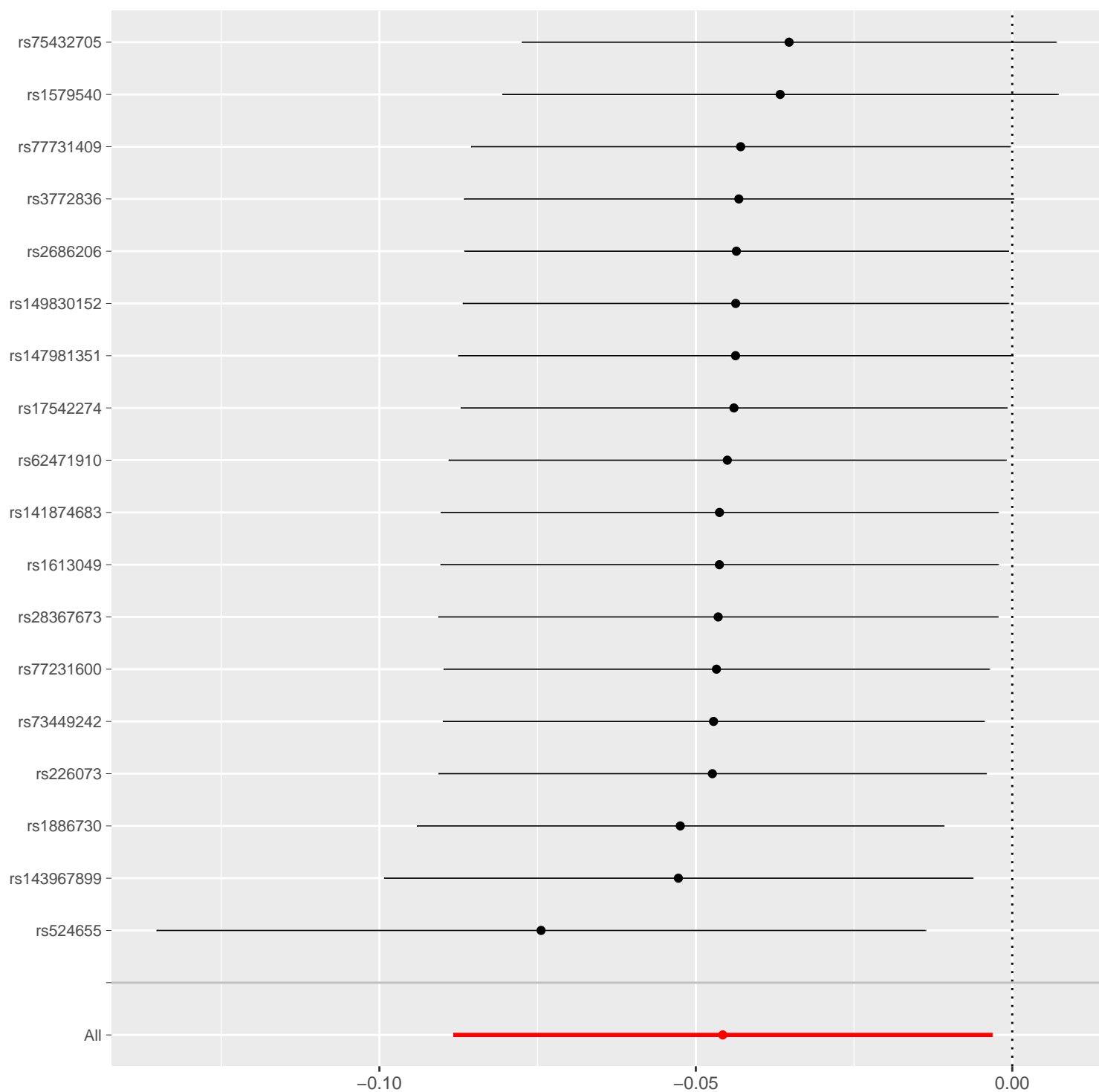

Supplement: Supporting Information — Supplement 1: The STROBE-MR checklist of recommended items to address in reports of Mendelian randomization studies. Supplement 2: The significant pleiotropy or heterogeneity of IVs in the MR analysis using gut microbiota as the exposure and osteonecrosis as the outcome. Supplement 3: The scatterplots and leave-one-out plots in the MR analysis using gut microbiota as the exposure and osteonecrosis as the outcome. Supplement 4: The significant pleiotropy or heterogeneity of IVs in the MR analysis using immune cells as the exposure and osteonecrosis as the outcome. Supplement 5: The scatterplots and leave-one-out plots in the MR analysis using immune cells as the exposure and osteonecrosis as the outcome. Supplement 6: The significant pleiotropy or heterogeneity of IVs in the MR analysis using gut microbiota as the exposure and immune cells as the outcome. Supplement 7: The scatterplots and leave-one-out plots in the MR analysis using gut microbiota as the exposure and immune cells as the outcome. Supplement 8: The results of the MR analysis using osteonecrosis as the exposure and gut microbiota and immune cells as the outcomes. [file 9323113.f1.zip › Supplement 5/ebi-a-GCST90001876/sensitivity-analysis.pdf]

# MR Test

- Inverse variance weighted
- MR Egger
- Simple mode
- Weighted median
- Weighted mode

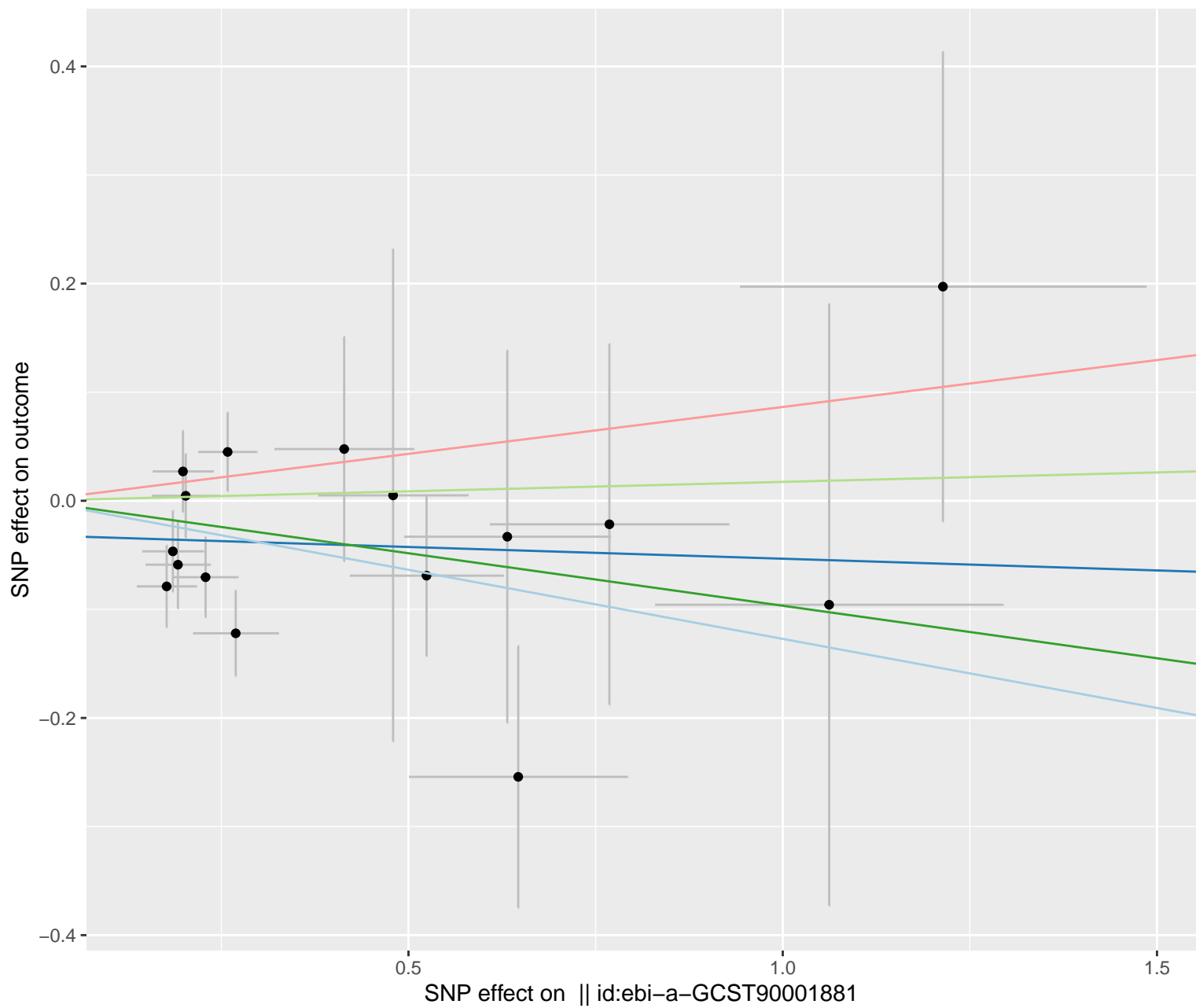

Supplement: Supporting Information — Supplement 1: The STROBE-MR checklist of recommended items to address in reports of Mendelian randomization studies. Supplement 2: The significant pleiotropy or heterogeneity of IVs in the MR analysis using gut microbiota as the exposure and osteonecrosis as the outcome. Supplement 3: The scatterplots and leave-one-out plots in the MR analysis using gut microbiota as the exposure and osteonecrosis as the outcome. Supplement 4: The significant pleiotropy or heterogeneity of IVs in the MR analysis using immune cells as the exposure and osteonecrosis as the outcome. Supplement 5: The scatterplots and leave-one-out plots in the MR analysis using immune cells as the exposure and osteonecrosis as the outcome. Supplement 6: The significant pleiotropy or heterogeneity of IVs in the MR analysis using gut microbiota as the exposure and immune cells as the outcome. Supplement 7: The scatterplots and leave-one-out plots in the MR analysis using gut microbiota as the exposure and immune cells as the outcome. Supplement 8: The results of the MR analysis using osteonecrosis as the exposure and gut microbiota and immune cells as the outcomes. [file 9323113.f1.zip › Supplement 5/ebi-a-GCST90001881/scatter.pdf]

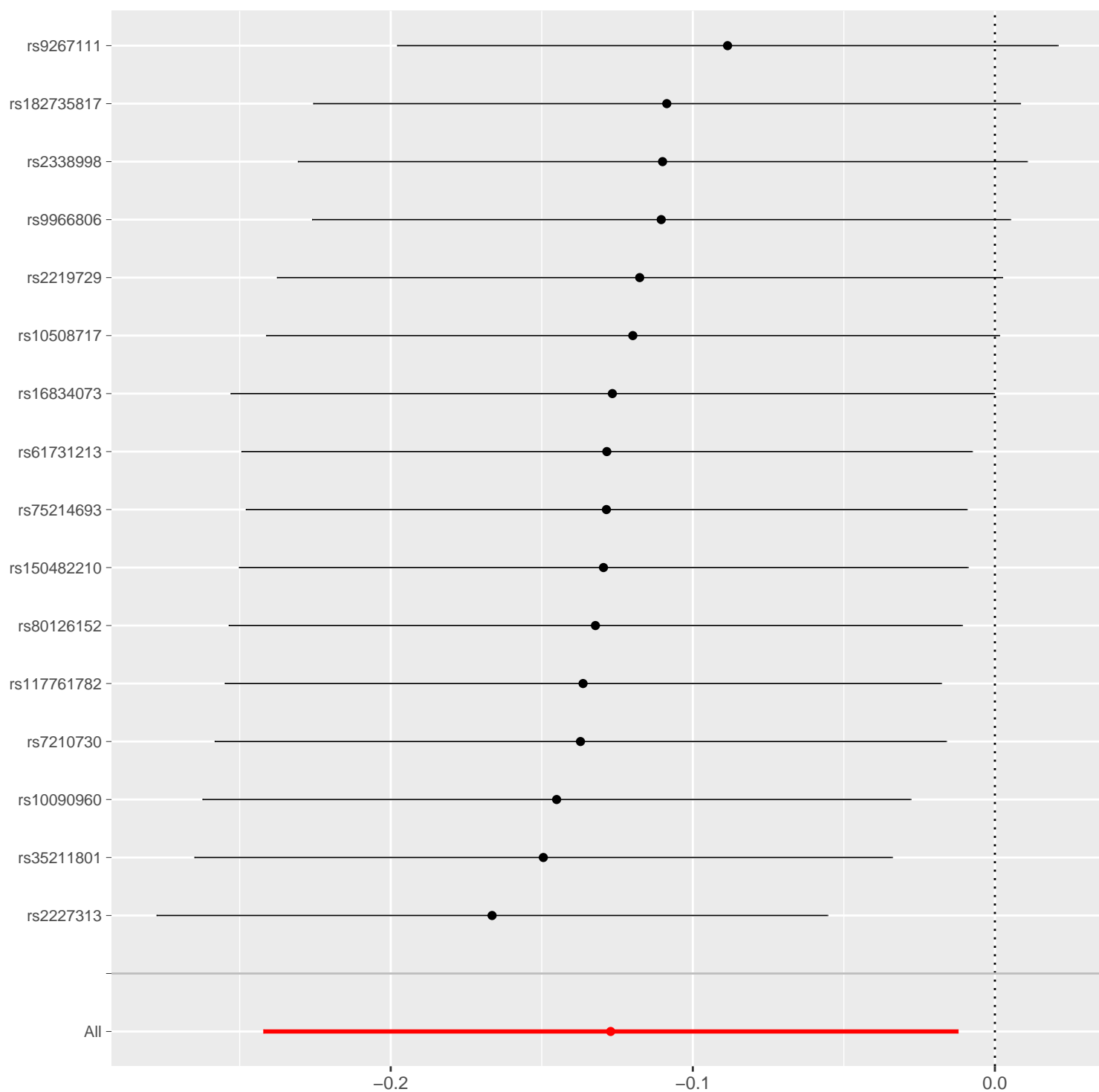

Supplement: Supporting Information — Supplement 1: The STROBE-MR checklist of recommended items to address in reports of Mendelian randomization studies. Supplement 2: The significant pleiotropy or heterogeneity of IVs in the MR analysis using gut microbiota as the exposure and osteonecrosis as the outcome. Supplement 3: The scatterplots and leave-one-out plots in the MR analysis using gut microbiota as the exposure and osteonecrosis as the outcome. Supplement 4: The significant pleiotropy or heterogeneity of IVs in the MR analysis using immune cells as the exposure and osteonecrosis as the outcome. Supplement 5: The scatterplots and leave-one-out plots in the MR analysis using immune cells as the exposure and osteonecrosis as the outcome. Supplement 6: The significant pleiotropy or heterogeneity of IVs in the MR analysis using gut microbiota as the exposure and immune cells as the outcome. Supplement 7: The scatterplots and leave-one-out plots in the MR analysis using gut microbiota as the exposure and immune cells as the outcome. Supplement 8: The results of the MR analysis using osteonecrosis as the exposure and gut microbiota and immune cells as the outcomes. [file 9323113.f1.zip › Supplement 5/ebi-a-GCST90001881/sensitivity-analysis.pdf]

# MR Test

- Inverse variance weighted
- MR Egger
- Simple mode
- Weighted median
- Weighted mode

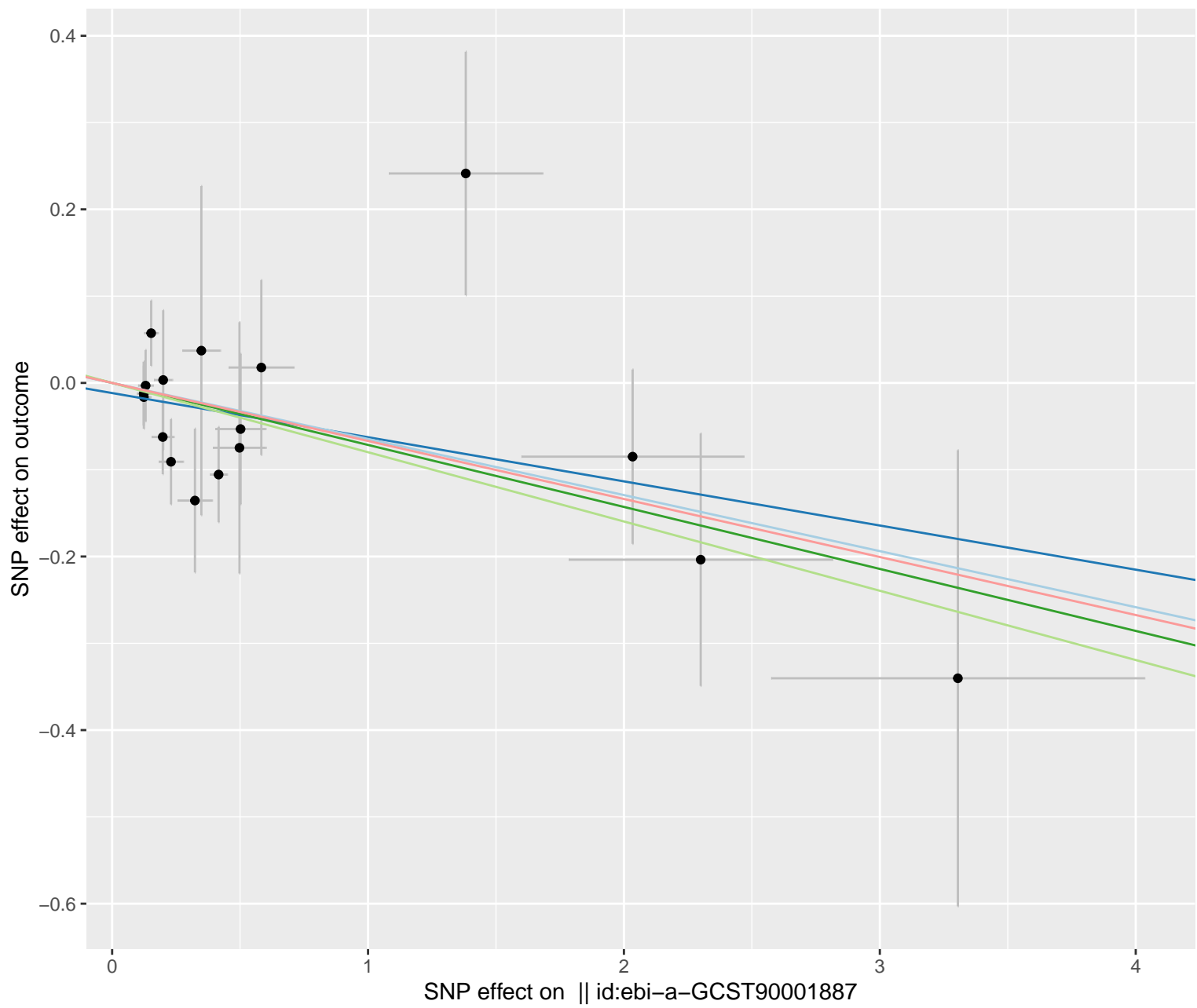

Supplement: Supporting Information — Supplement 1: The STROBE-MR checklist of recommended items to address in reports of Mendelian randomization studies. Supplement 2: The significant pleiotropy or heterogeneity of IVs in the MR analysis using gut microbiota as the exposure and osteonecrosis as the outcome. Supplement 3: The scatterplots and leave-one-out plots in the MR analysis using gut microbiota as the exposure and osteonecrosis as the outcome. Supplement 4: The significant pleiotropy or heterogeneity of IVs in the MR analysis using immune cells as the exposure and osteonecrosis as the outcome. Supplement 5: The scatterplots and leave-one-out plots in the MR analysis using immune cells as the exposure and osteonecrosis as the outcome. Supplement 6: The significant pleiotropy or heterogeneity of IVs in the MR analysis using gut microbiota as the exposure and immune cells as the outcome. Supplement 7: The scatterplots and leave-one-out plots in the MR analysis using gut microbiota as the exposure and immune cells as the outcome. Supplement 8: The results of the MR analysis using osteonecrosis as the exposure and gut microbiota and immune cells as the outcomes. [file 9323113.f1.zip › Supplement 5/ebi-a-GCST90001887/scatter.pdf]

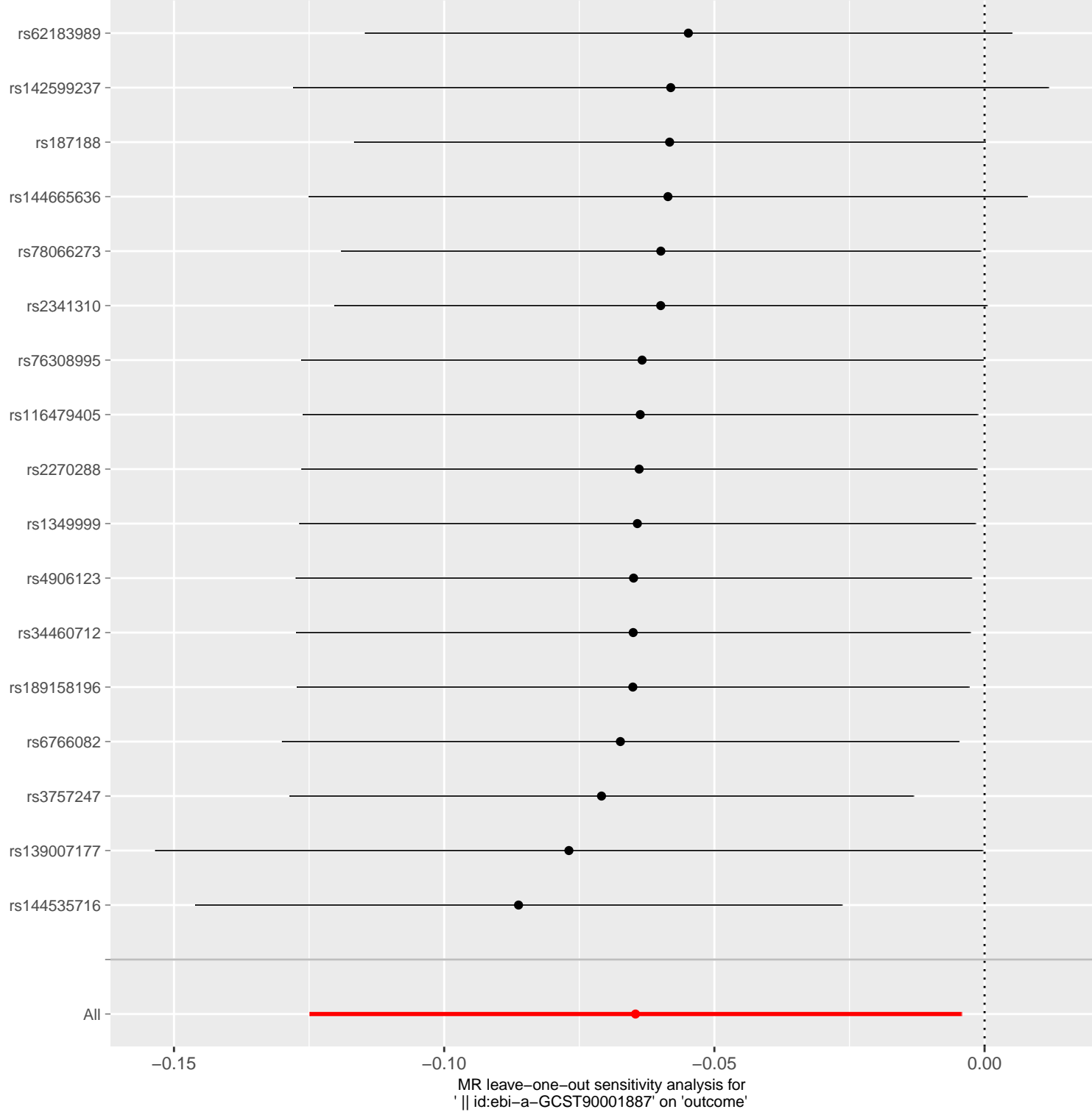

Supplement: Supporting Information — Supplement 1: The STROBE-MR checklist of recommended items to address in reports of Mendelian randomization studies. Supplement 2: The significant pleiotropy or heterogeneity of IVs in the MR analysis using gut microbiota as the exposure and osteonecrosis as the outcome. Supplement 3: The scatterplots and leave-one-out plots in the MR analysis using gut microbiota as the exposure and osteonecrosis as the outcome. Supplement 4: The significant pleiotropy or heterogeneity of IVs in the MR analysis using immune cells as the exposure and osteonecrosis as the outcome. Supplement 5: The scatterplots and leave-one-out plots in the MR analysis using immune cells as the exposure and osteonecrosis as the outcome. Supplement 6: The significant pleiotropy or heterogeneity of IVs in the MR analysis using gut microbiota as the exposure and immune cells as the outcome. Supplement 7: The scatterplots and leave-one-out plots in the MR analysis using gut microbiota as the exposure and immune cells as the outcome. Supplement 8: The results of the MR analysis using osteonecrosis as the exposure and gut microbiota and immune cells as the outcomes. [file 9323113.f1.zip › Supplement 5/ebi-a-GCST90001887/sensitivity-analysis.pdf]

# MR Test

- Inverse variance weighted
- MR Egger
- Simple mode
- Weighted median
- Weighted mode

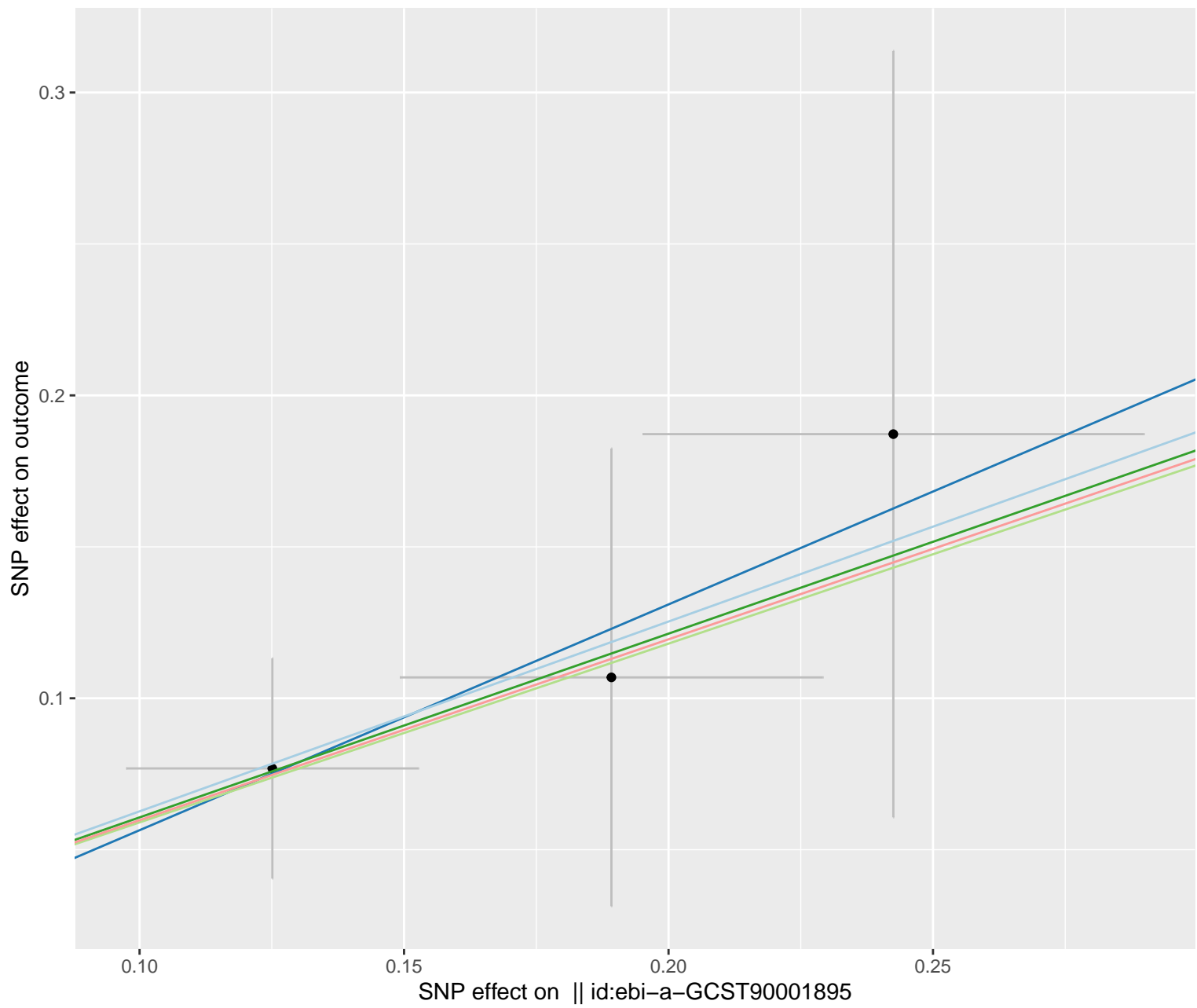

Supplement: Supporting Information — Supplement 1: The STROBE-MR checklist of recommended items to address in reports of Mendelian randomization studies. Supplement 2: The significant pleiotropy or heterogeneity of IVs in the MR analysis using gut microbiota as the exposure and osteonecrosis as the outcome. Supplement 3: The scatterplots and leave-one-out plots in the MR analysis using gut microbiota as the exposure and osteonecrosis as the outcome. Supplement 4: The significant pleiotropy or heterogeneity of IVs in the MR analysis using immune cells as the exposure and osteonecrosis as the outcome. Supplement 5: The scatterplots and leave-one-out plots in the MR analysis using immune cells as the exposure and osteonecrosis as the outcome. Supplement 6: The significant pleiotropy or heterogeneity of IVs in the MR analysis using gut microbiota as the exposure and immune cells as the outcome. Supplement 7: The scatterplots and leave-one-out plots in the MR analysis using gut microbiota as the exposure and immune cells as the outcome. Supplement 8: The results of the MR analysis using osteonecrosis as the exposure and gut microbiota and immune cells as the outcomes. [file 9323113.f1.zip › Supplement 5/ebi-a-GCST90001895/scatter.pdf]

rs79457820

rs2992756

rs873041

All

0.0

0.5

1.0

MR leave-one-out sensitivity analysis for  
' || id:ebi-a-GCST90001895' on 'outcome'

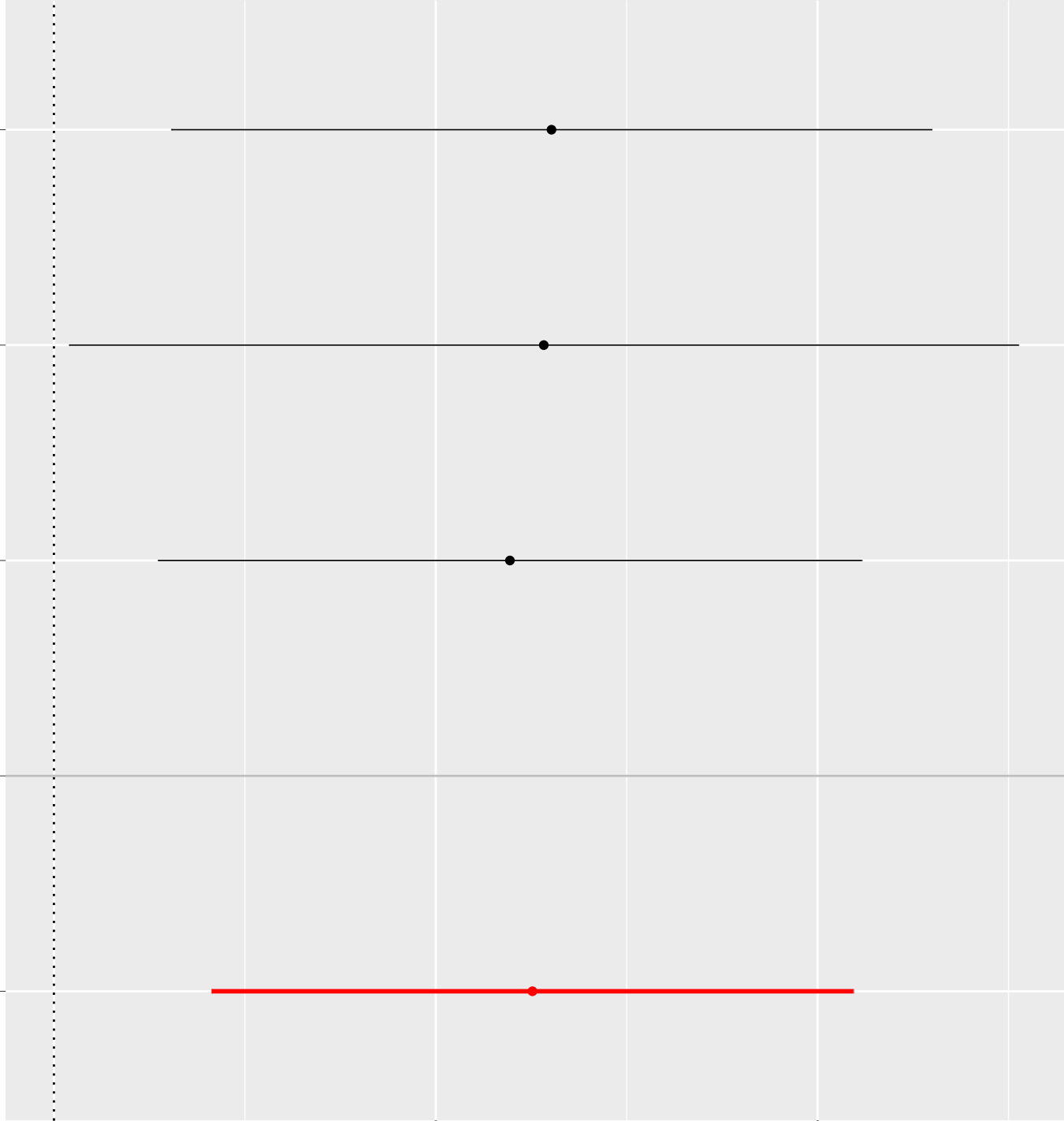

Supplement: Supporting Information — Supplement 1: The STROBE-MR checklist of recommended items to address in reports of Mendelian randomization studies. Supplement 2: The significant pleiotropy or heterogeneity of IVs in the MR analysis using gut microbiota as the exposure and osteonecrosis as the outcome. Supplement 3: The scatterplots and leave-one-out plots in the MR analysis using gut microbiota as the exposure and osteonecrosis as the outcome. Supplement 4: The significant pleiotropy or heterogeneity of IVs in the MR analysis using immune cells as the exposure and osteonecrosis as the outcome. Supplement 5: The scatterplots and leave-one-out plots in the MR analysis using immune cells as the exposure and osteonecrosis as the outcome. Supplement 6: The significant pleiotropy or heterogeneity of IVs in the MR analysis using gut microbiota as the exposure and immune cells as the outcome. Supplement 7: The scatterplots and leave-one-out plots in the MR analysis using gut microbiota as the exposure and immune cells as the outcome. Supplement 8: The results of the MR analysis using osteonecrosis as the exposure and gut microbiota and immune cells as the outcomes. [file 9323113.f1.zip › Supplement 5/ebi-a-GCST90001895/sensitivity-analysis.pdf]

# MR Test

- Inverse variance weighted
- MR Egger
- Simple mode
- Weighted median
- Weighted mode

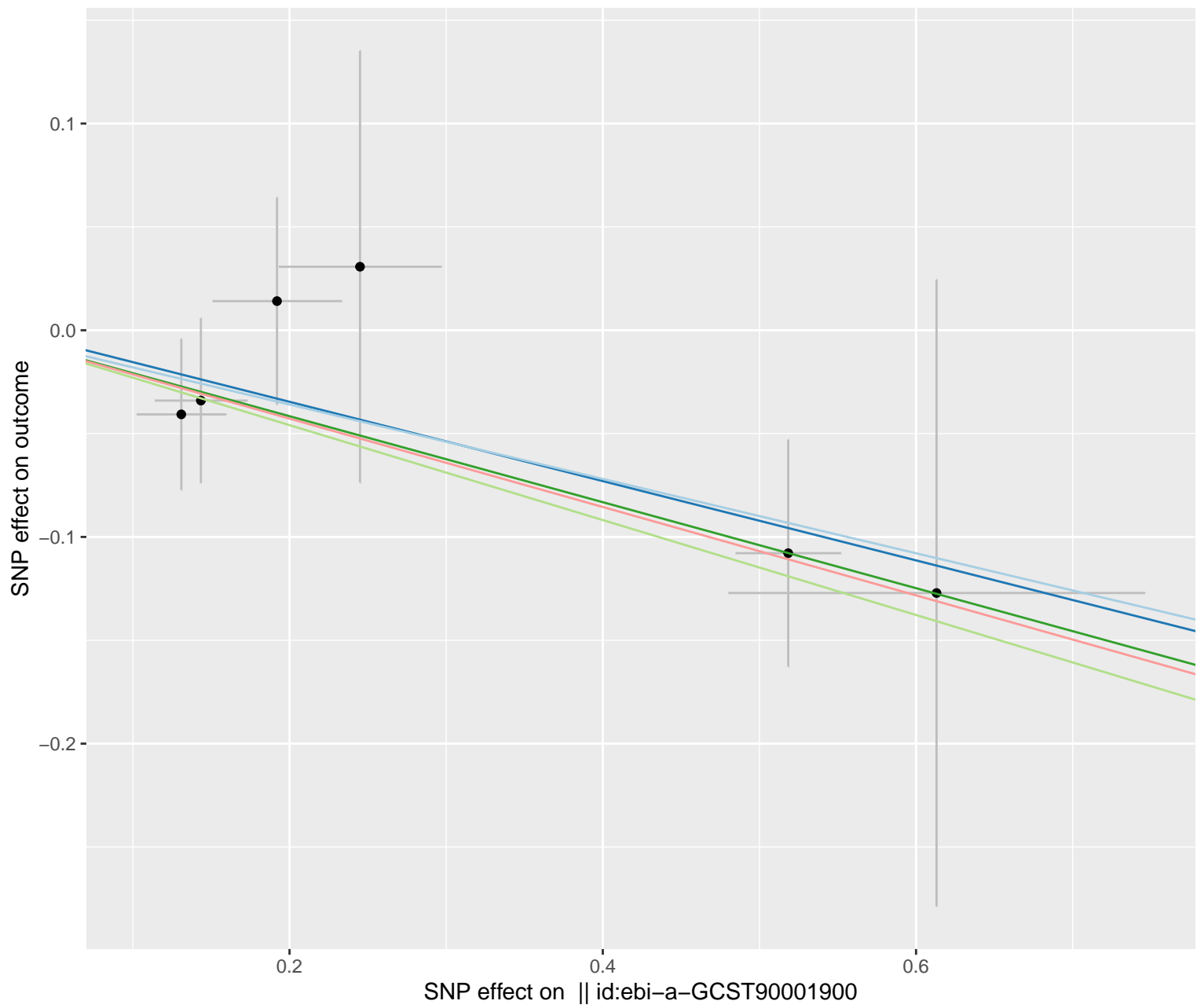

Supplement: Supporting Information — Supplement 1: The STROBE-MR checklist of recommended items to address in reports of Mendelian randomization studies. Supplement 2: The significant pleiotropy or heterogeneity of IVs in the MR analysis using gut microbiota as the exposure and osteonecrosis as the outcome. Supplement 3: The scatterplots and leave-one-out plots in the MR analysis using gut microbiota as the exposure and osteonecrosis as the outcome. Supplement 4: The significant pleiotropy or heterogeneity of IVs in the MR analysis using immune cells as the exposure and osteonecrosis as the outcome. Supplement 5: The scatterplots and leave-one-out plots in the MR analysis using immune cells as the exposure and osteonecrosis as the outcome. Supplement 6: The significant pleiotropy or heterogeneity of IVs in the MR analysis using gut microbiota as the exposure and immune cells as the outcome. Supplement 7: The scatterplots and leave-one-out plots in the MR analysis using gut microbiota as the exposure and immune cells as the outcome. Supplement 8: The results of the MR analysis using osteonecrosis as the exposure and gut microbiota and immune cells as the outcomes. [file 9323113.f1.zip › Supplement 5/ebi-a-GCST90001900/scatter.pdf]

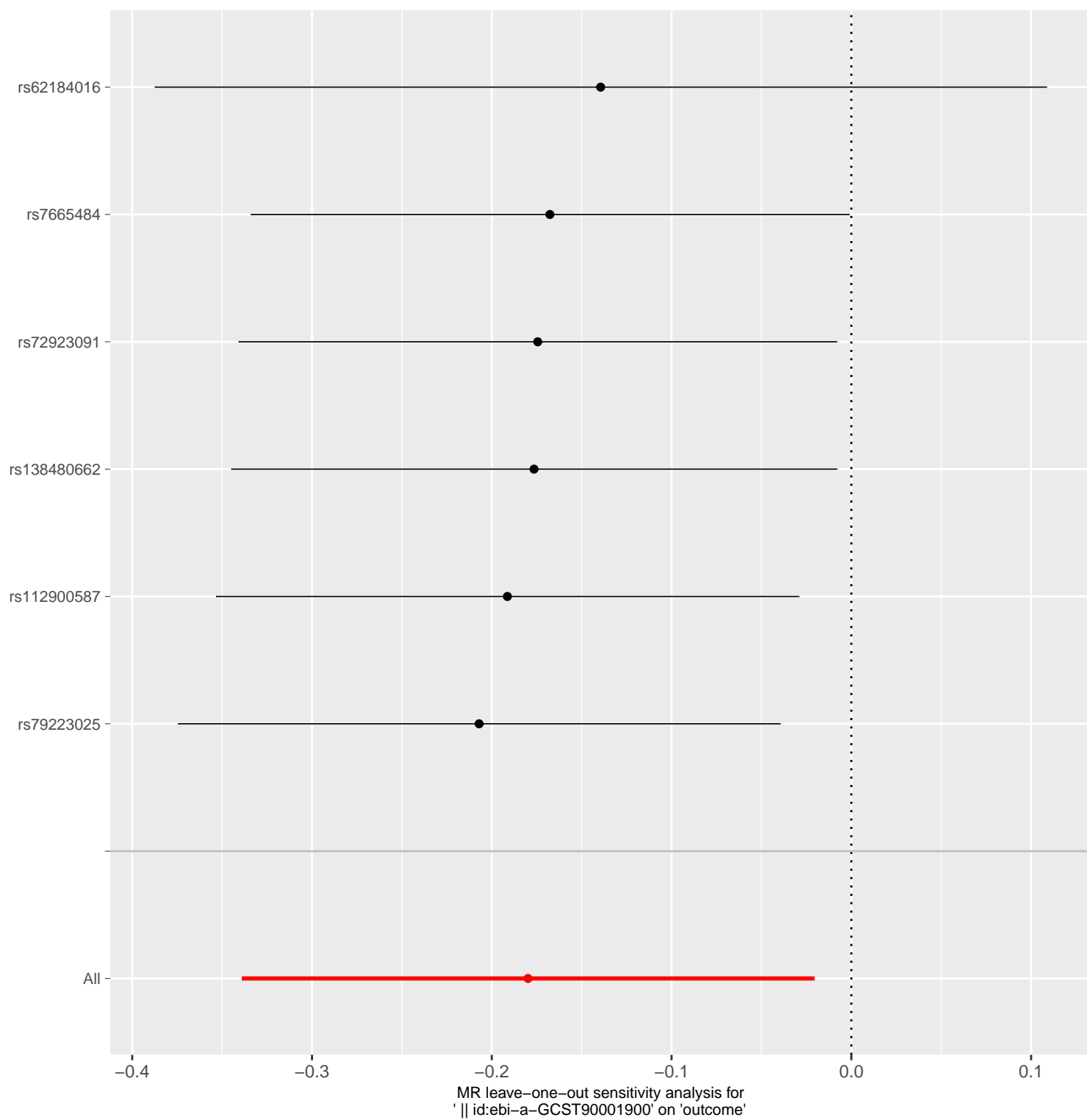

Supplement: Supporting Information — Supplement 1: The STROBE-MR checklist of recommended items to address in reports of Mendelian randomization studies. Supplement 2: The significant pleiotropy or heterogeneity of IVs in the MR analysis using gut microbiota as the exposure and osteonecrosis as the outcome. Supplement 3: The scatterplots and leave-one-out plots in the MR analysis using gut microbiota as the exposure and osteonecrosis as the outcome. Supplement 4: The significant pleiotropy or heterogeneity of IVs in the MR analysis using immune cells as the exposure and osteonecrosis as the outcome. Supplement 5: The scatterplots and leave-one-out plots in the MR analysis using immune cells as the exposure and osteonecrosis as the outcome. Supplement 6: The significant pleiotropy or heterogeneity of IVs in the MR analysis using gut microbiota as the exposure and immune cells as the outcome. Supplement 7: The scatterplots and leave-one-out plots in the MR analysis using gut microbiota as the exposure and immune cells as the outcome. Supplement 8: The results of the MR analysis using osteonecrosis as the exposure and gut microbiota and immune cells as the outcomes. [file 9323113.f1.zip › Supplement 5/ebi-a-GCST90001900/sensitivity-analysis.pdf]

# MR Test

- Inverse variance weighted
- MR Egger
- Simple mode
- Weighted median
- Weighted mode

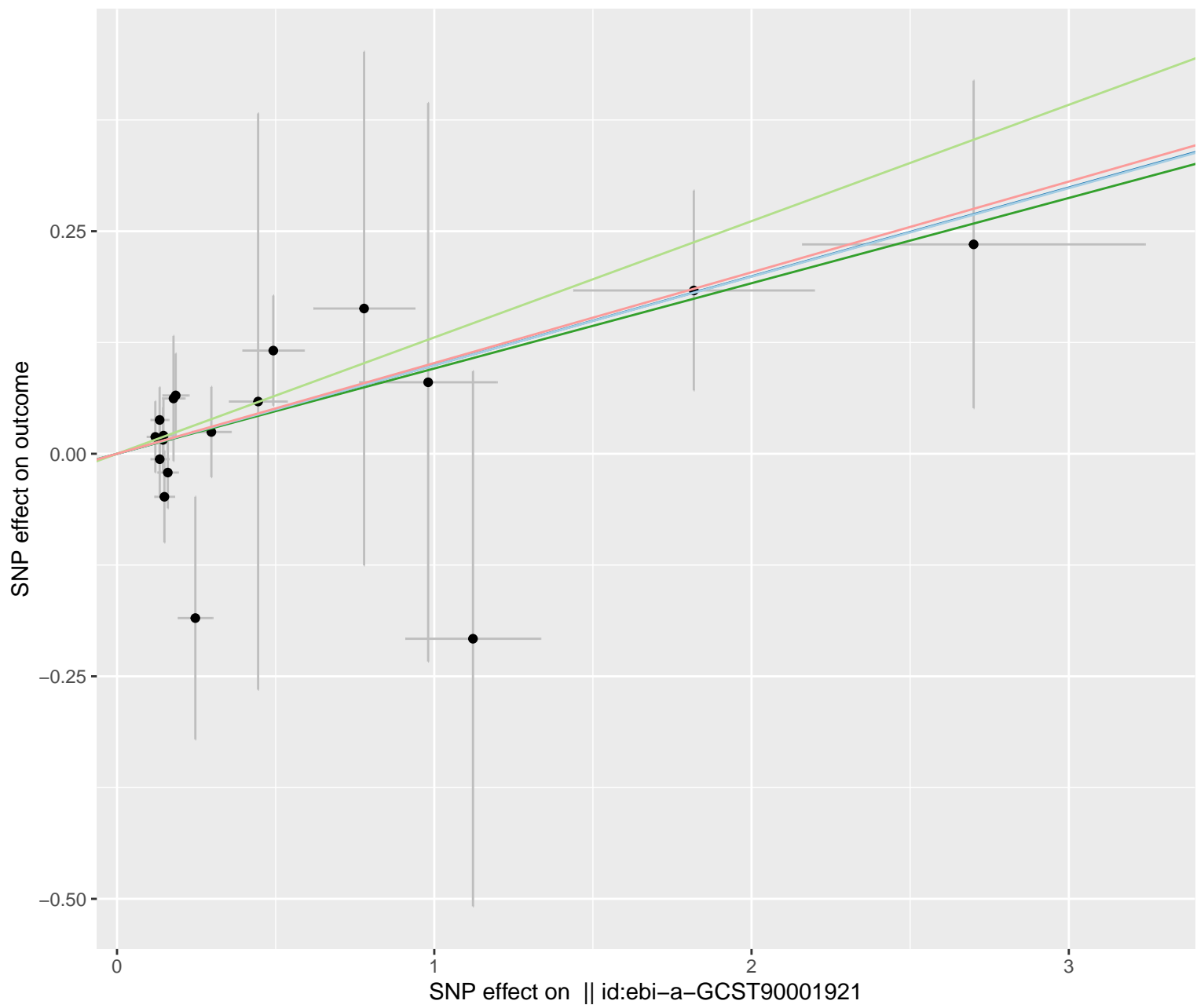

Supplement: Supporting Information — Supplement 1: The STROBE-MR checklist of recommended items to address in reports of Mendelian randomization studies. Supplement 2: The significant pleiotropy or heterogeneity of IVs in the MR analysis using gut microbiota as the exposure and osteonecrosis as the outcome. Supplement 3: The scatterplots and leave-one-out plots in the MR analysis using gut microbiota as the exposure and osteonecrosis as the outcome. Supplement 4: The significant pleiotropy or heterogeneity of IVs in the MR analysis using immune cells as the exposure and osteonecrosis as the outcome. Supplement 5: The scatterplots and leave-one-out plots in the MR analysis using immune cells as the exposure and osteonecrosis as the outcome. Supplement 6: The significant pleiotropy or heterogeneity of IVs in the MR analysis using gut microbiota as the exposure and immune cells as the outcome. Supplement 7: The scatterplots and leave-one-out plots in the MR analysis using gut microbiota as the exposure and immune cells as the outcome. Supplement 8: The results of the MR analysis using osteonecrosis as the exposure and gut microbiota and immune cells as the outcomes. [file 9323113.f1.zip › Supplement 5/ebi-a-GCST90001921/scatter.pdf]

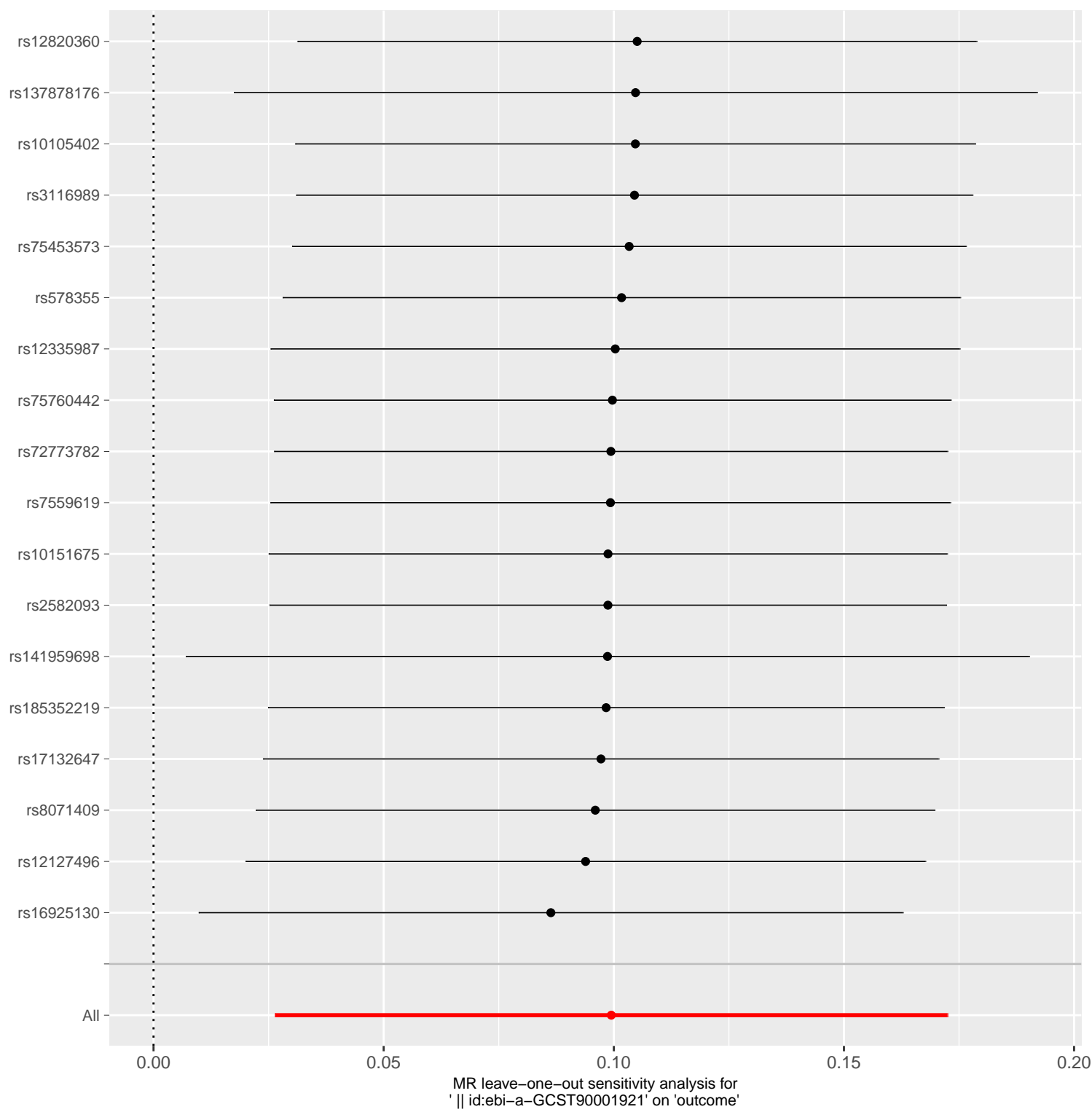

Supplement: Supporting Information — Supplement 1: The STROBE-MR checklist of recommended items to address in reports of Mendelian randomization studies. Supplement 2: The significant pleiotropy or heterogeneity of IVs in the MR analysis using gut microbiota as the exposure and osteonecrosis as the outcome. Supplement 3: The scatterplots and leave-one-out plots in the MR analysis using gut microbiota as the exposure and osteonecrosis as the outcome. Supplement 4: The significant pleiotropy or heterogeneity of IVs in the MR analysis using immune cells as the exposure and osteonecrosis as the outcome. Supplement 5: The scatterplots and leave-one-out plots in the MR analysis using immune cells as the exposure and osteonecrosis as the outcome. Supplement 6: The significant pleiotropy or heterogeneity of IVs in the MR analysis using gut microbiota as the exposure and immune cells as the outcome. Supplement 7: The scatterplots and leave-one-out plots in the MR analysis using gut microbiota as the exposure and immune cells as the outcome. Supplement 8: The results of the MR analysis using osteonecrosis as the exposure and gut microbiota and immune cells as the outcomes. [file 9323113.f1.zip › Supplement 5/ebi-a-GCST90001921/sensitivity-analysis.pdf]

# MR Test

- Inverse variance weighted
- MR Egger
- Simple mode
- Weighted median
- Weighted mode

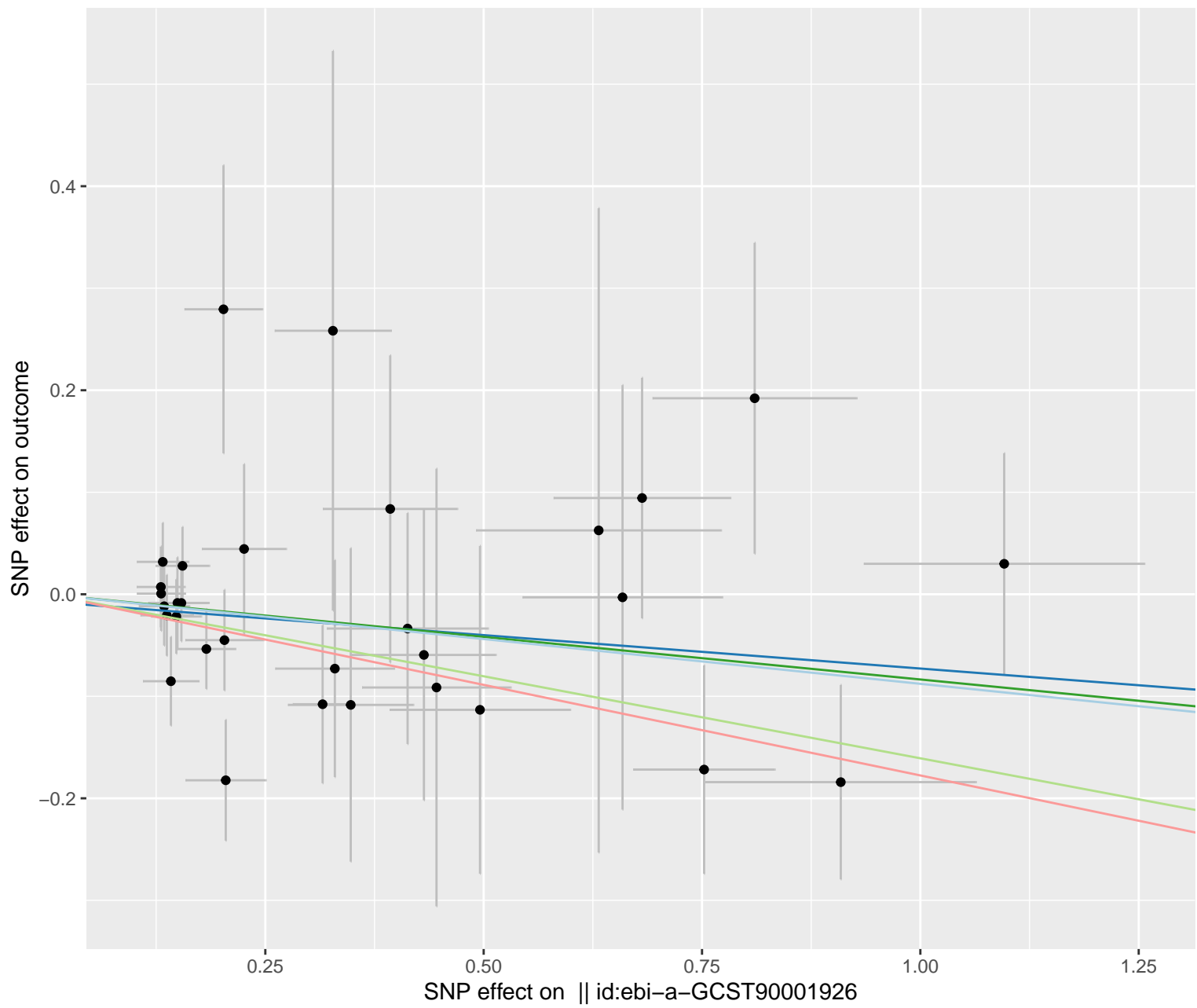

Supplement: Supporting Information — Supplement 1: The STROBE-MR checklist of recommended items to address in reports of Mendelian randomization studies. Supplement 2: The significant pleiotropy or heterogeneity of IVs in the MR analysis using gut microbiota as the exposure and osteonecrosis as the outcome. Supplement 3: The scatterplots and leave-one-out plots in the MR analysis using gut microbiota as the exposure and osteonecrosis as the outcome. Supplement 4: The significant pleiotropy or heterogeneity of IVs in the MR analysis using immune cells as the exposure and osteonecrosis as the outcome. Supplement 5: The scatterplots and leave-one-out plots in the MR analysis using immune cells as the exposure and osteonecrosis as the outcome. Supplement 6: The significant pleiotropy or heterogeneity of IVs in the MR analysis using gut microbiota as the exposure and immune cells as the outcome. Supplement 7: The scatterplots and leave-one-out plots in the MR analysis using gut microbiota as the exposure and immune cells as the outcome. Supplement 8: The results of the MR analysis using osteonecrosis as the exposure and gut microbiota and immune cells as the outcomes. [file 9323113.f1.zip › Supplement 5/ebi-a-GCST90001926/scatter.pdf]

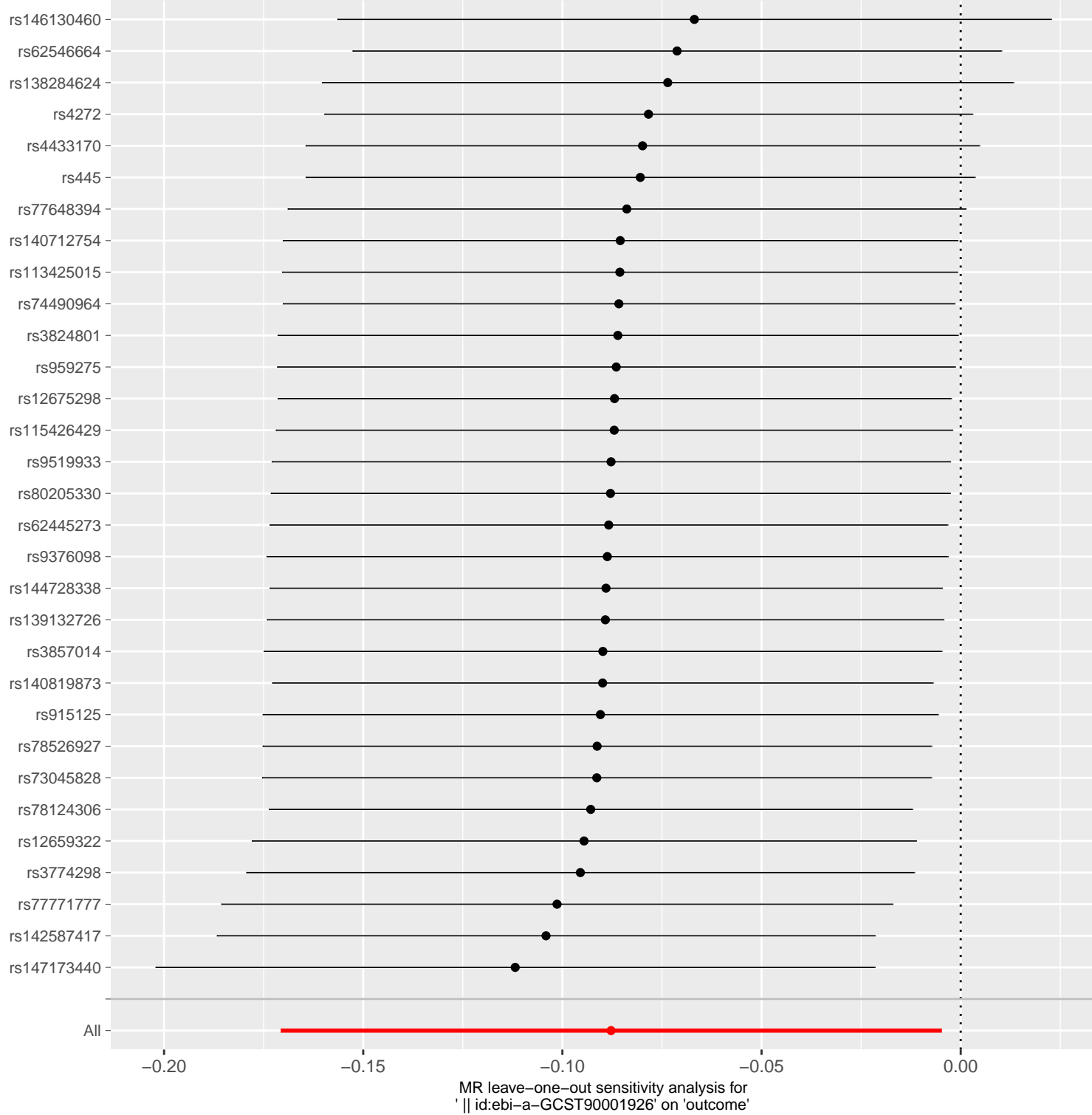

Supplement: Supporting Information — Supplement 1: The STROBE-MR checklist of recommended items to address in reports of Mendelian randomization studies. Supplement 2: The significant pleiotropy or heterogeneity of IVs in the MR analysis using gut microbiota as the exposure and osteonecrosis as the outcome. Supplement 3: The scatterplots and leave-one-out plots in the MR analysis using gut microbiota as the exposure and osteonecrosis as the outcome. Supplement 4: The significant pleiotropy or heterogeneity of IVs in the MR analysis using immune cells as the exposure and osteonecrosis as the outcome. Supplement 5: The scatterplots and leave-one-out plots in the MR analysis using immune cells as the exposure and osteonecrosis as the outcome. Supplement 6: The significant pleiotropy or heterogeneity of IVs in the MR analysis using gut microbiota as the exposure and immune cells as the outcome. Supplement 7: The scatterplots and leave-one-out plots in the MR analysis using gut microbiota as the exposure and immune cells as the outcome. Supplement 8: The results of the MR analysis using osteonecrosis as the exposure and gut microbiota and immune cells as the outcomes. [file 9323113.f1.zip › Supplement 5/ebi-a-GCST90001926/sensitivity-analysis.pdf]

# MR Test

- Inverse variance weighted
- MR Egger
- Simple mode
- Weighted median
- Weighted mode

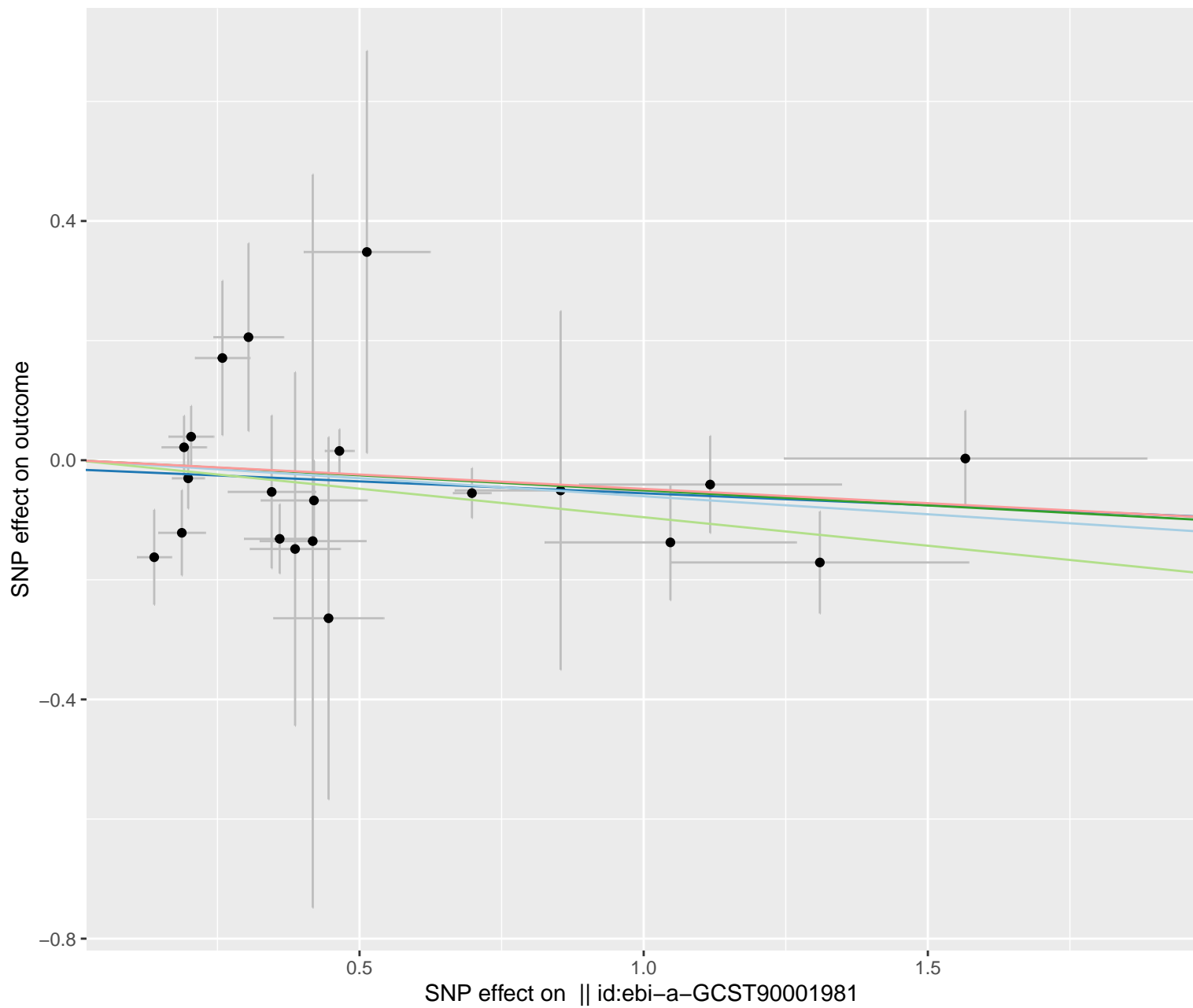

Supplement: Supporting Information — Supplement 1: The STROBE-MR checklist of recommended items to address in reports of Mendelian randomization studies. Supplement 2: The significant pleiotropy or heterogeneity of IVs in the MR analysis using gut microbiota as the exposure and osteonecrosis as the outcome. Supplement 3: The scatterplots and leave-one-out plots in the MR analysis using gut microbiota as the exposure and osteonecrosis as the outcome. Supplement 4: The significant pleiotropy or heterogeneity of IVs in the MR analysis using immune cells as the exposure and osteonecrosis as the outcome. Supplement 5: The scatterplots and leave-one-out plots in the MR analysis using immune cells as the exposure and osteonecrosis as the outcome. Supplement 6: The significant pleiotropy or heterogeneity of IVs in the MR analysis using gut microbiota as the exposure and immune cells as the outcome. Supplement 7: The scatterplots and leave-one-out plots in the MR analysis using gut microbiota as the exposure and immune cells as the outcome. Supplement 8: The results of the MR analysis using osteonecrosis as the exposure and gut microbiota and immune cells as the outcomes. [file 9323113.f1.zip › Supplement 5/ebi-a-GCST90001981/scatter.pdf]

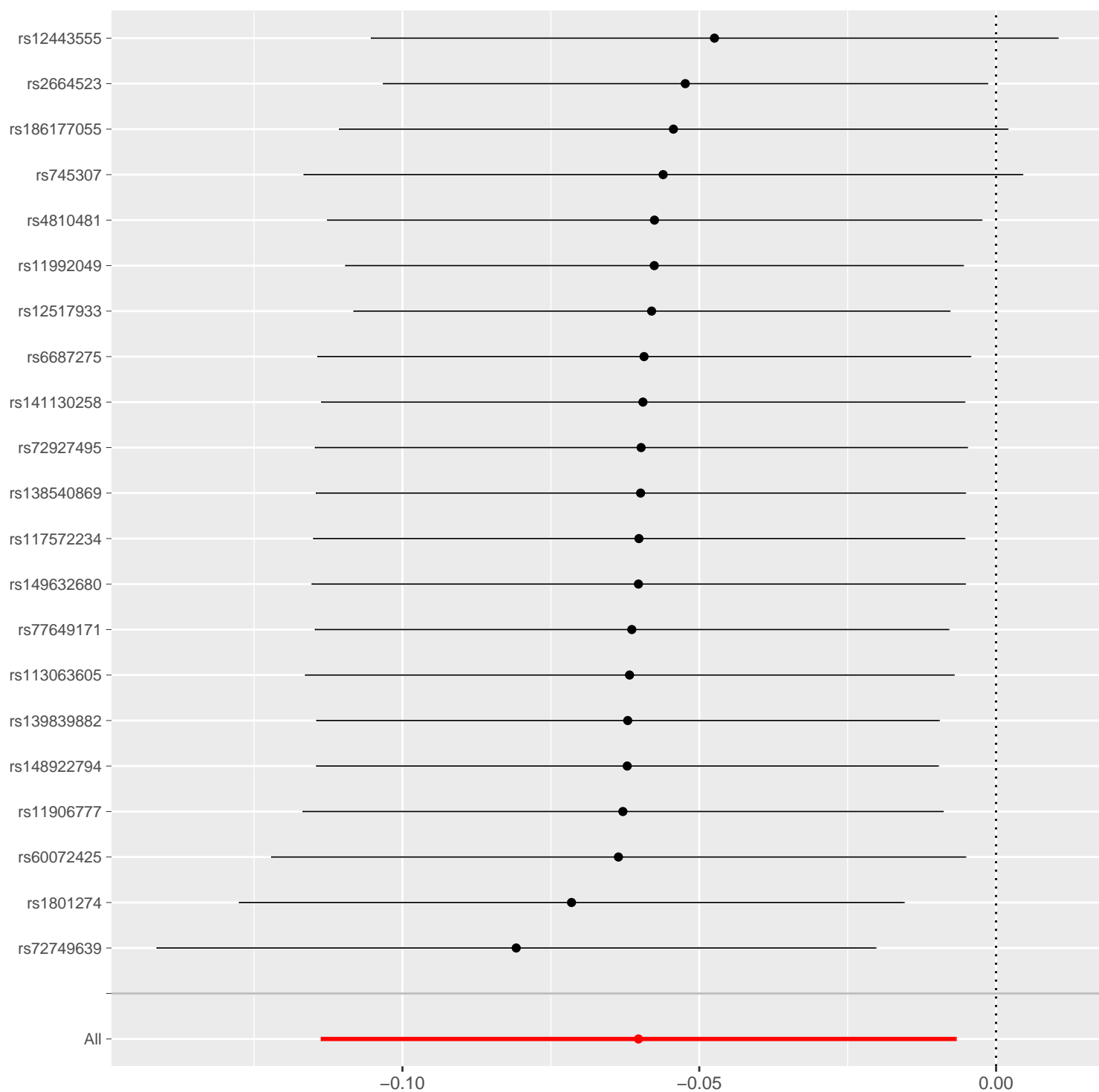

Supplement: Supporting Information — Supplement 1: The STROBE-MR checklist of recommended items to address in reports of Mendelian randomization studies. Supplement 2: The significant pleiotropy or heterogeneity of IVs in the MR analysis using gut microbiota as the exposure and osteonecrosis as the outcome. Supplement 3: The scatterplots and leave-one-out plots in the MR analysis using gut microbiota as the exposure and osteonecrosis as the outcome. Supplement 4: The significant pleiotropy or heterogeneity of IVs in the MR analysis using immune cells as the exposure and osteonecrosis as the outcome. Supplement 5: The scatterplots and leave-one-out plots in the MR analysis using immune cells as the exposure and osteonecrosis as the outcome. Supplement 6: The significant pleiotropy or heterogeneity of IVs in the MR analysis using gut microbiota as the exposure and immune cells as the outcome. Supplement 7: The scatterplots and leave-one-out plots in the MR analysis using gut microbiota as the exposure and immune cells as the outcome. Supplement 8: The results of the MR analysis using osteonecrosis as the exposure and gut microbiota and immune cells as the outcomes. [file 9323113.f1.zip › Supplement 5/ebi-a-GCST90001981/sensitivity-analysis.pdf]

# MR Test

- Inverse variance weighted
- MR Egger
- Simple mode
- Weighted median
- Weighted mode

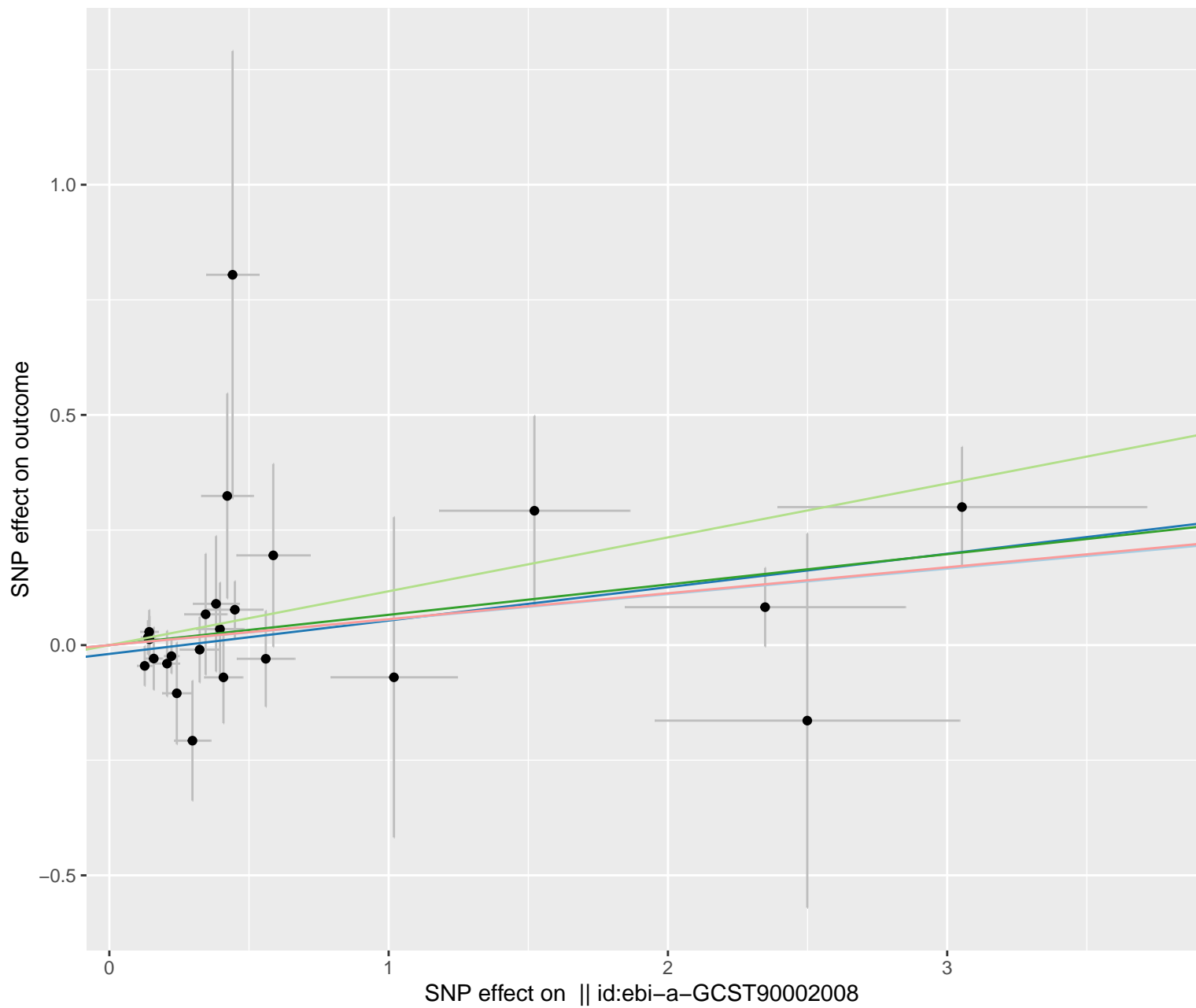

Supplement: Supporting Information — Supplement 1: The STROBE-MR checklist of recommended items to address in reports of Mendelian randomization studies. Supplement 2: The significant pleiotropy or heterogeneity of IVs in the MR analysis using gut microbiota as the exposure and osteonecrosis as the outcome. Supplement 3: The scatterplots and leave-one-out plots in the MR analysis using gut microbiota as the exposure and osteonecrosis as the outcome. Supplement 4: The significant pleiotropy or heterogeneity of IVs in the MR analysis using immune cells as the exposure and osteonecrosis as the outcome. Supplement 5: The scatterplots and leave-one-out plots in the MR analysis using immune cells as the exposure and osteonecrosis as the outcome. Supplement 6: The significant pleiotropy or heterogeneity of IVs in the MR analysis using gut microbiota as the exposure and immune cells as the outcome. Supplement 7: The scatterplots and leave-one-out plots in the MR analysis using gut microbiota as the exposure and immune cells as the outcome. Supplement 8: The results of the MR analysis using osteonecrosis as the exposure and gut microbiota and immune cells as the outcomes. [file 9323113.f1.zip › Supplement 5/ebi-a-GCST90002008/scatter.pdf]

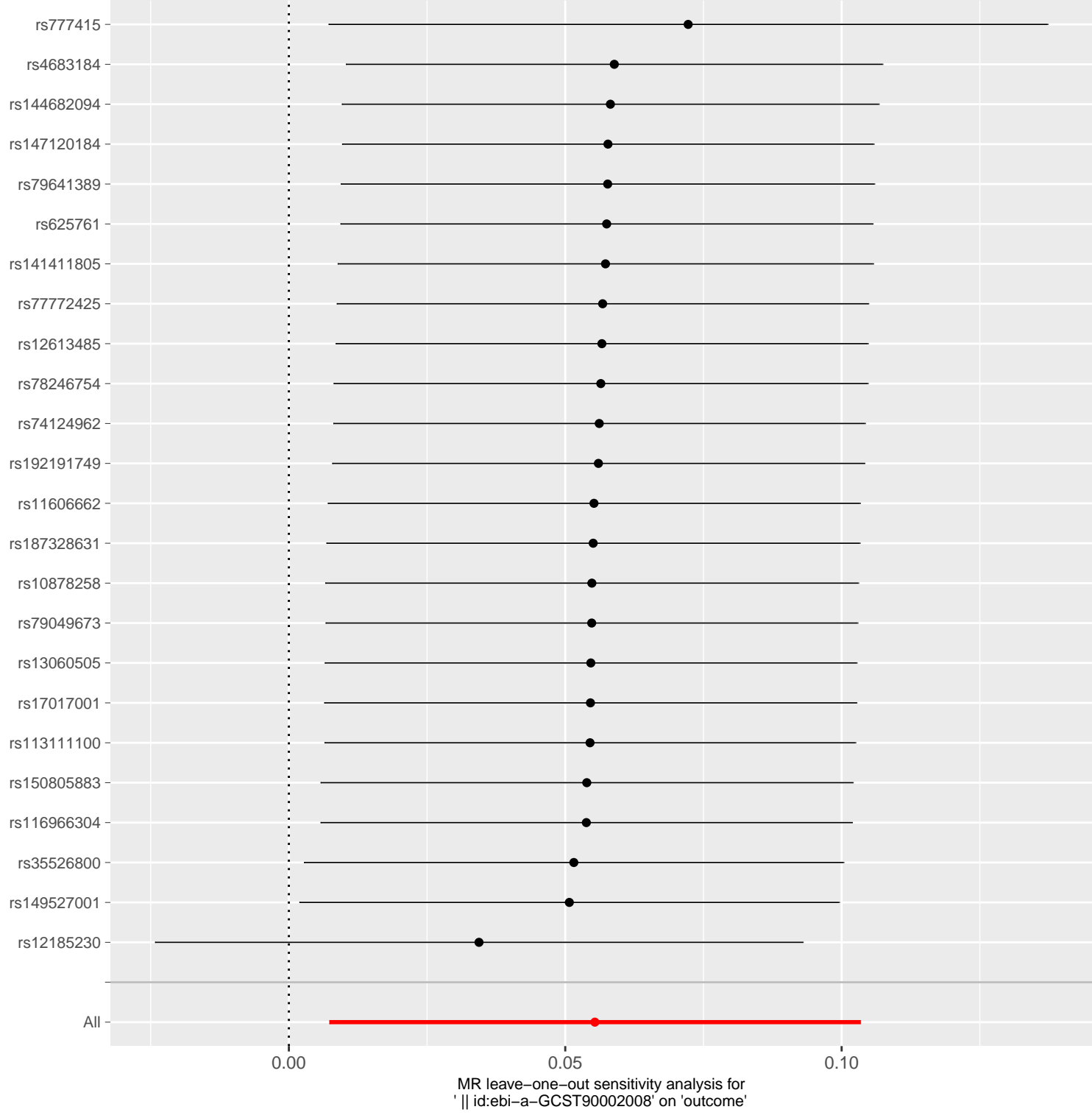

Supplement: Supporting Information — Supplement 1: The STROBE-MR checklist of recommended items to address in reports of Mendelian randomization studies. Supplement 2: The significant pleiotropy or heterogeneity of IVs in the MR analysis using gut microbiota as the exposure and osteonecrosis as the outcome. Supplement 3: The scatterplots and leave-one-out plots in the MR analysis using gut microbiota as the exposure and osteonecrosis as the outcome. Supplement 4: The significant pleiotropy or heterogeneity of IVs in the MR analysis using immune cells as the exposure and osteonecrosis as the outcome. Supplement 5: The scatterplots and leave-one-out plots in the MR analysis using immune cells as the exposure and osteonecrosis as the outcome. Supplement 6: The significant pleiotropy or heterogeneity of IVs in the MR analysis using gut microbiota as the exposure and immune cells as the outcome. Supplement 7: The scatterplots and leave-one-out plots in the MR analysis using gut microbiota as the exposure and immune cells as the outcome. Supplement 8: The results of the MR analysis using osteonecrosis as the exposure and gut microbiota and immune cells as the outcomes. [file 9323113.f1.zip › Supplement 5/ebi-a-GCST90002008/sensitivity-analysis.pdf]

# MR Test

- Inverse variance weighted
- MR Egger
- Simple mode
- Weighted median
- Weighted mode

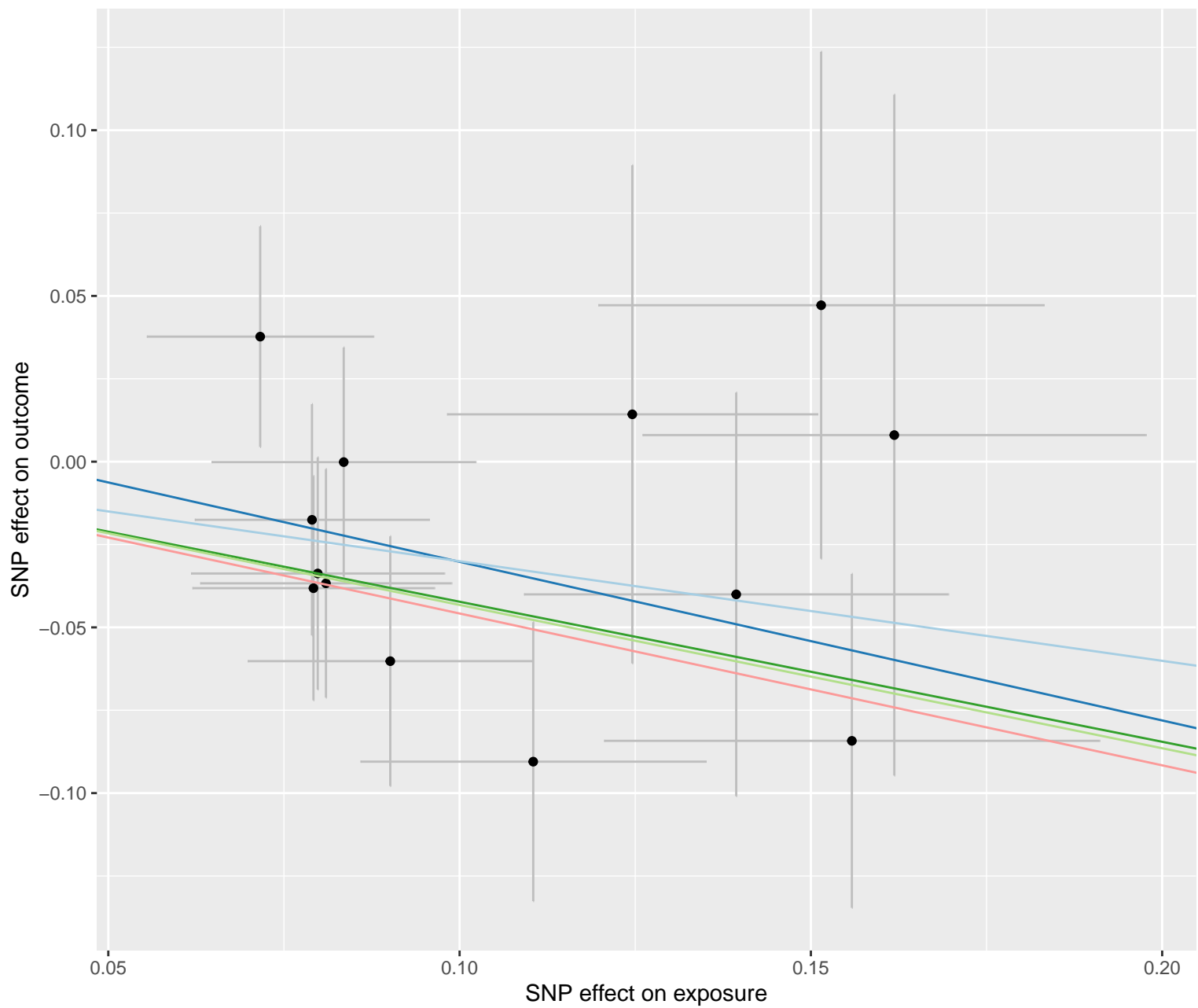

Supplement: Supporting Information — Supplement 1: The STROBE-MR checklist of recommended items to address in reports of Mendelian randomization studies. Supplement 2: The significant pleiotropy or heterogeneity of IVs in the MR analysis using gut microbiota as the exposure and osteonecrosis as the outcome. Supplement 3: The scatterplots and leave-one-out plots in the MR analysis using gut microbiota as the exposure and osteonecrosis as the outcome. Supplement 4: The significant pleiotropy or heterogeneity of IVs in the MR analysis using immune cells as the exposure and osteonecrosis as the outcome. Supplement 5: The scatterplots and leave-one-out plots in the MR analysis using immune cells as the exposure and osteonecrosis as the outcome. Supplement 6: The significant pleiotropy or heterogeneity of IVs in the MR analysis using gut microbiota as the exposure and immune cells as the outcome. Supplement 7: The scatterplots and leave-one-out plots in the MR analysis using gut microbiota as the exposure and immune cells as the outcome. Supplement 8: The results of the MR analysis using osteonecrosis as the exposure and gut microbiota and immune cells as the outcomes. [file 9323113.f1.zip › Supplement 7/ebi-a-GCST90001523/scatter.pdf]

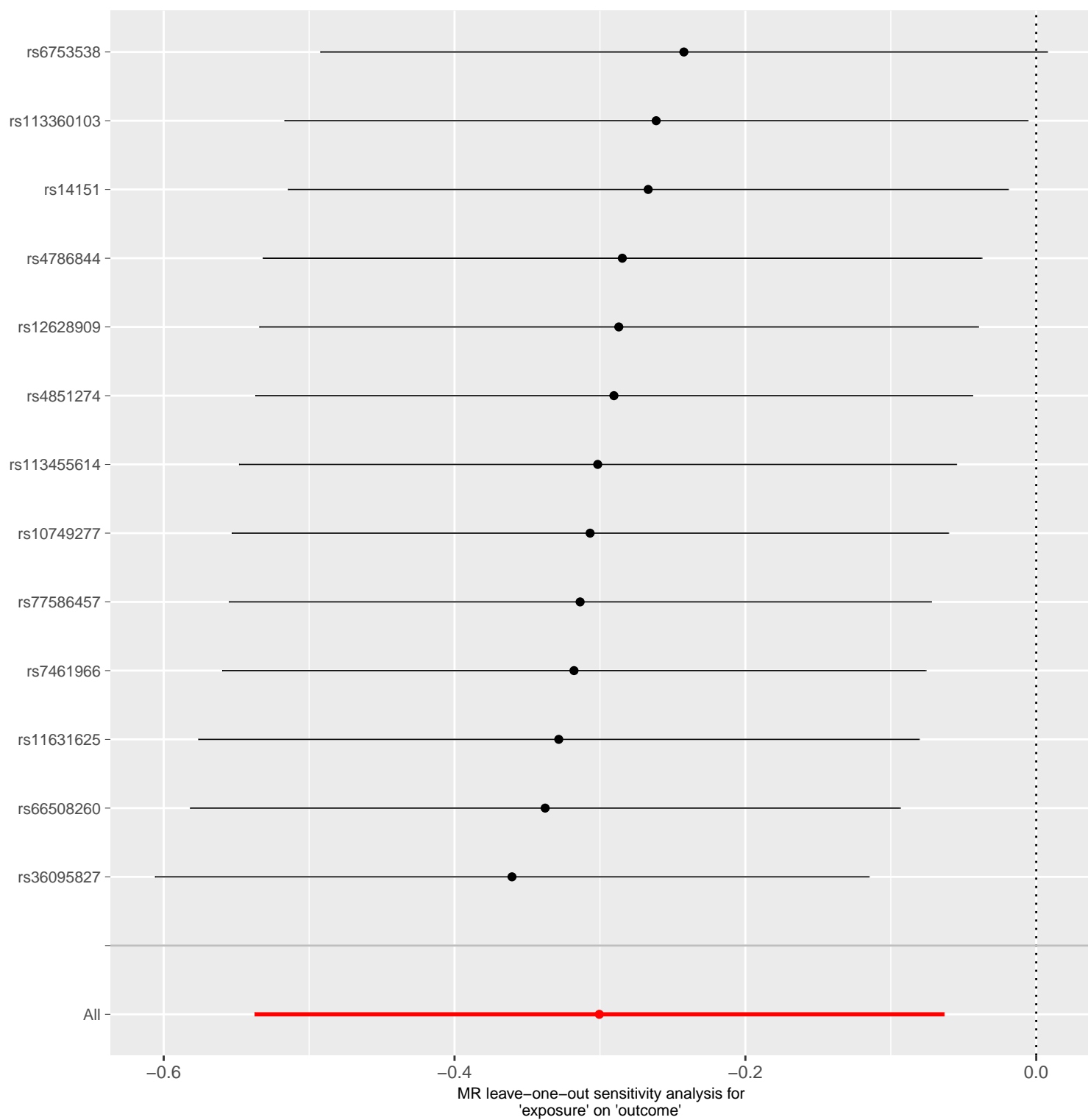

Supplement: Supporting Information — Supplement 1: The STROBE-MR checklist of recommended items to address in reports of Mendelian randomization studies. Supplement 2: The significant pleiotropy or heterogeneity of IVs in the MR analysis using gut microbiota as the exposure and osteonecrosis as the outcome. Supplement 3: The scatterplots and leave-one-out plots in the MR analysis using gut microbiota as the exposure and osteonecrosis as the outcome. Supplement 4: The significant pleiotropy or heterogeneity of IVs in the MR analysis using immune cells as the exposure and osteonecrosis as the outcome. Supplement 5: The scatterplots and leave-one-out plots in the MR analysis using immune cells as the exposure and osteonecrosis as the outcome. Supplement 6: The significant pleiotropy or heterogeneity of IVs in the MR analysis using gut microbiota as the exposure and immune cells as the outcome. Supplement 7: The scatterplots and leave-one-out plots in the MR analysis using gut microbiota as the exposure and immune cells as the outcome. Supplement 8: The results of the MR analysis using osteonecrosis as the exposure and gut microbiota and immune cells as the outcomes. [file 9323113.f1.zip › Supplement 7/ebi-a-GCST90001523/sensitivity-analysis.pdf]

# MR Test

- Inverse variance weighted
- MR Egger
- Simple mode
- Weighted median
- Weighted mode

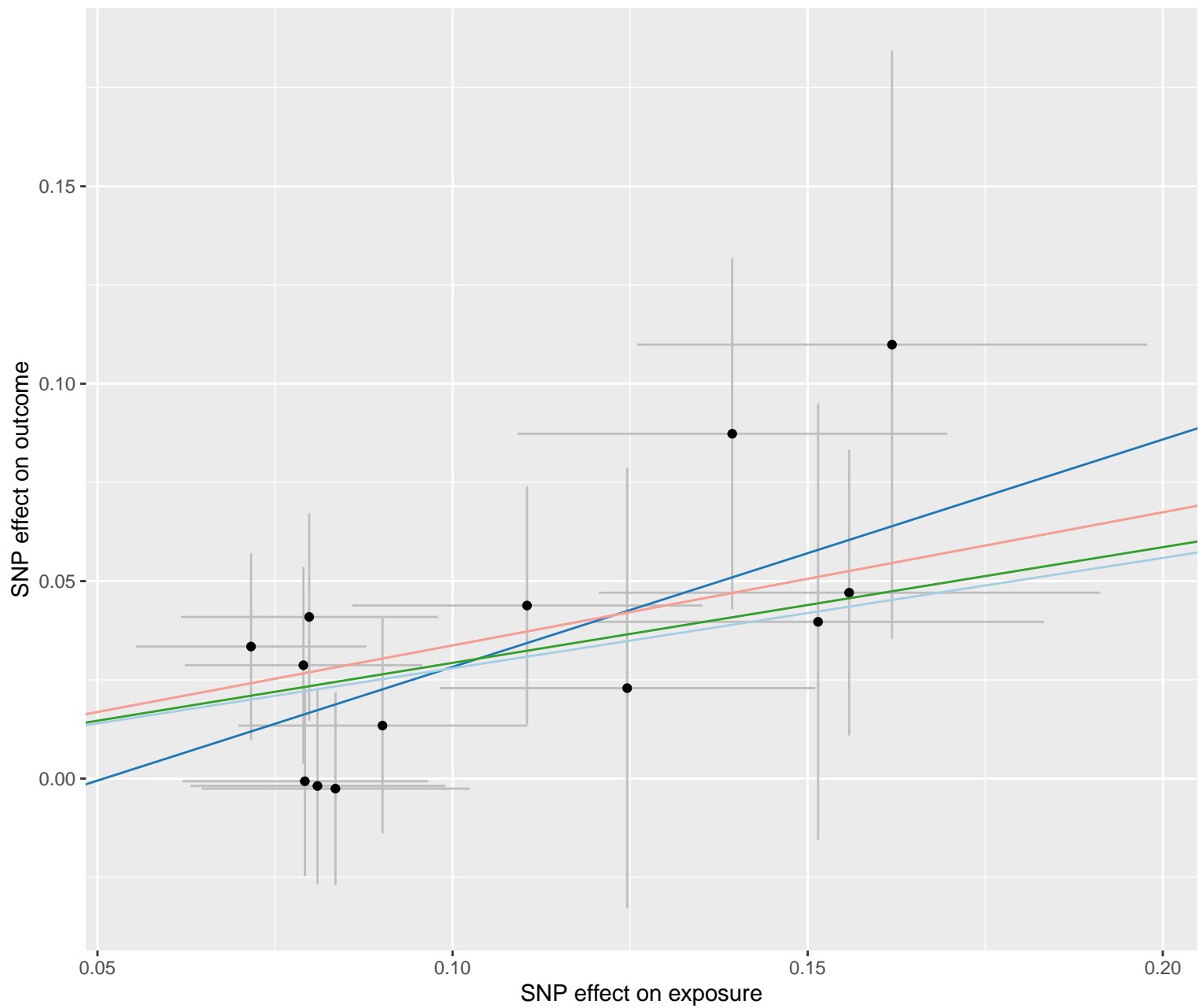

Supplement: Supporting Information — Supplement 1: The STROBE-MR checklist of recommended items to address in reports of Mendelian randomization studies. Supplement 2: The significant pleiotropy or heterogeneity of IVs in the MR analysis using gut microbiota as the exposure and osteonecrosis as the outcome. Supplement 3: The scatterplots and leave-one-out plots in the MR analysis using gut microbiota as the exposure and osteonecrosis as the outcome. Supplement 4: The significant pleiotropy or heterogeneity of IVs in the MR analysis using immune cells as the exposure and osteonecrosis as the outcome. Supplement 5: The scatterplots and leave-one-out plots in the MR analysis using immune cells as the exposure and osteonecrosis as the outcome. Supplement 6: The significant pleiotropy or heterogeneity of IVs in the MR analysis using gut microbiota as the exposure and immune cells as the outcome. Supplement 7: The scatterplots and leave-one-out plots in the MR analysis using gut microbiota as the exposure and immune cells as the outcome. Supplement 8: The results of the MR analysis using osteonecrosis as the exposure and gut microbiota and immune cells as the outcomes. [file 9323113.f1.zip › Supplement 7/ebi-a-GCST90001604/scatter.pdf]

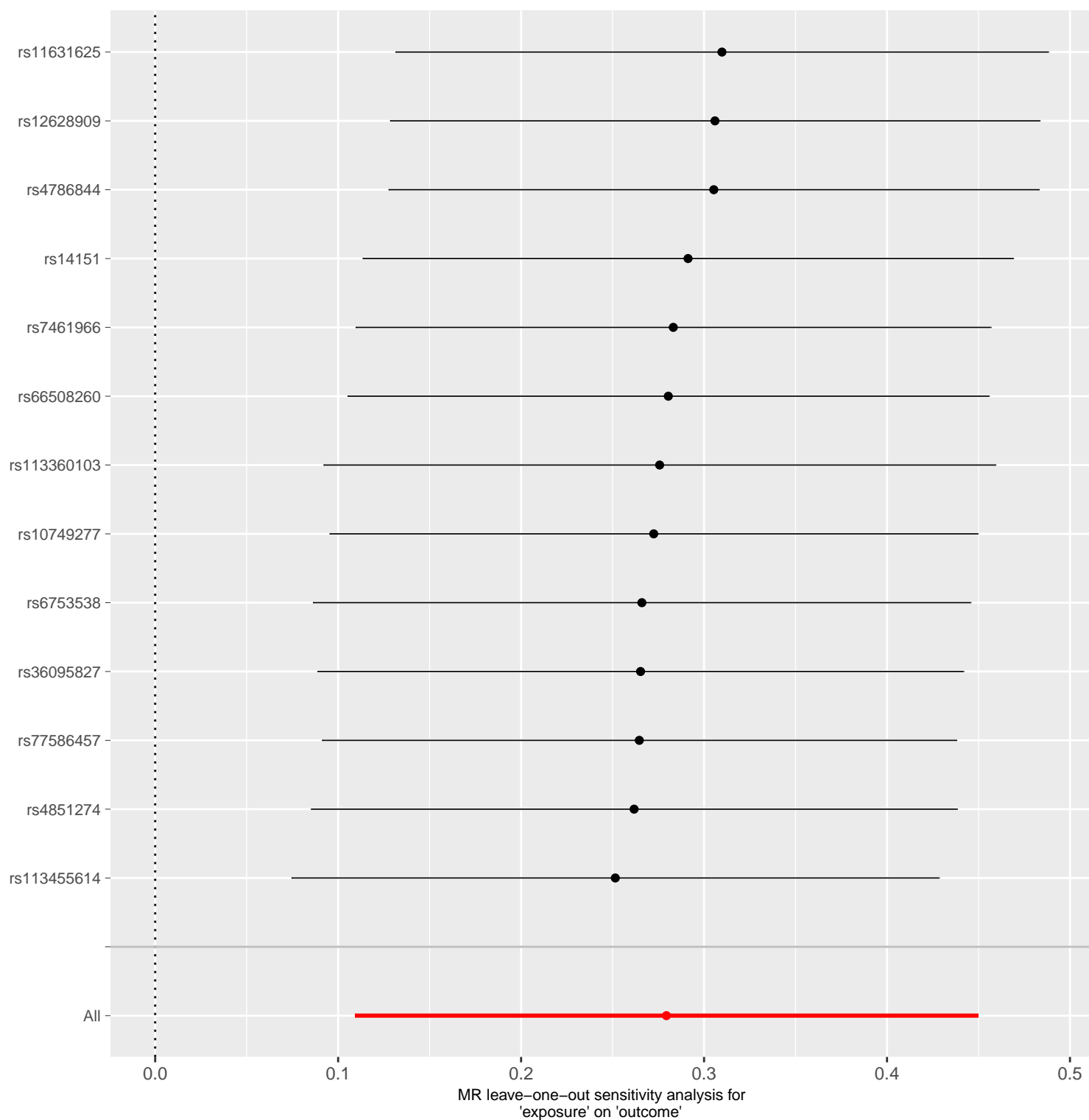

Supplement: Supporting Information — Supplement 1: The STROBE-MR checklist of recommended items to address in reports of Mendelian randomization studies. Supplement 2: The significant pleiotropy or heterogeneity of IVs in the MR analysis using gut microbiota as the exposure and osteonecrosis as the outcome. Supplement 3: The scatterplots and leave-one-out plots in the MR analysis using gut microbiota as the exposure and osteonecrosis as the outcome. Supplement 4: The significant pleiotropy or heterogeneity of IVs in the MR analysis using immune cells as the exposure and osteonecrosis as the outcome. Supplement 5: The scatterplots and leave-one-out plots in the MR analysis using immune cells as the exposure and osteonecrosis as the outcome. Supplement 6: The significant pleiotropy or heterogeneity of IVs in the MR analysis using gut microbiota as the exposure and immune cells as the outcome. Supplement 7: The scatterplots and leave-one-out plots in the MR analysis using gut microbiota as the exposure and immune cells as the outcome. Supplement 8: The results of the MR analysis using osteonecrosis as the exposure and gut microbiota and immune cells as the outcomes. [file 9323113.f1.zip › Supplement 7/ebi-a-GCST90001604/sensitivity-analysis.pdf]
